# Building up Pt^II^−Thiosemicarbazone−Lysine−sC18 Conjugates

**DOI:** 10.1002/cbic.202000564

**Published:** 2020-11-06

**Authors:** Alexander Haseloer, Tamara Lützenburg, Joss Pepe Strache, Jörg Neudörfl, Ines Neundorf, Axel Klein

**Affiliations:** ^1^ Universität zu Köln, Department für Chemie Institut für Anorganische Chemie Greinstraße 6 50939 Köln Germany; ^2^ Universität zu Köln Department für Chemie, Institut für Biochemie Zülpicher Strasse 47a 50674 Köln Germany; ^3^ Universität zu Köln Department für Chemie, Institut für Organische Chemie Greinstraße 4 50939 Köln Germany

**Keywords:** Antiproliferative, cell-penetrating peptides, conjugates, platinum, thiosemicarbazone

## Abstract

Three chiral tridentate N^N^S coordinating pyridine‐carbaldehyde (*S*)‐*N*4‐(α‐methylbenzyl)thiosemicarbazones (HTSCmB) were synthesised along with lysine‐modified derivatives. One of them was selected and covalently conjugated to the cell‐penetrating peptide sC18 by solid‐phase peptide synthesis. The HTSCmB model ligands, the HTSCLp derivatives and the peptide conjugate rapidly and quantitatively form very stable Pt^II^ chlorido complexes [Pt(TSC)Cl] when treated with K_2_PtCl_4_ in solution. The Pt(CN) derivatives were obtained from one TSCmB model complex and the peptide conjugate complex through Cl^−^→CN^−^ exchange. Ligands and complexes were characterised by NMR, IR spectroscopy, HR‐ESI‐MS and single‐crystal XRD. Intriguingly, no decrease in cell viability was observed when testing the biological activity of the lysine‐tagged HdpyTSCLp, its sC18 conjugate HdpyTSCL‐sC18 or the PtCl and Pt(CN) conjugate complexes in three different cell lines. Thus, given the facile and effective preparation of such Pt‐TSC‐peptide conjugates, these systems might pave the way for future use in late‐stage labelling with Pt radionuclides and application in nuclear medicine.

## Graphical abstract

**Late‐stage labelling**: Chiral pyridine‐carbaldehyde (*S*)‐*N*4‐(α‐methylbenzyl)thiosemicarbazones were lysine modified (TSCLp) and covalently conjugated to the cell‐penetrating peptide sC18 by SPPS. Very stable Pt^II^ complexes [Pt(TSC)X] (X=Cl or CN) were synthesised, and the biological activity of the TSCLp, conjugate TSCL‐sC18, or Pt conjugate complexes was studied.

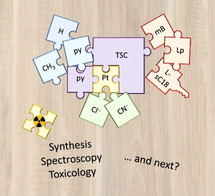

## Introduction

Thiosemicarbazones (TSC) have been studied in the last 70 years with regard to their use as versatile ligands in coordination chemistry[^1^, ^2^, ^3^, ^4^, ^5^, ^6^, ^7^, ^8^, ^9^, ^10^, ^11^, ^12^, ^13^, ^14^, ^15^, ^16^, ^17^] with applications in transition metal catalysis,[^1^, ^4^, ^5^, ^6^] and as materials for biomedical imaging and treatment.[^5^, ^6^, ^7^, ^8^, ^9^, ^10^, ^11^, ^12^, ^13^, ^14^, ^15^, ^16^, ^17^] TSCs of heterocycles, especially pyridine, form a vast group within the class of TSC. They can act as tridentate N^N^Sx chelate ligands forming stable complexes with many metals[^1^, ^2^, ^3^] and many studies have shown their cytotoxic behaviour with and without a coordinated metal ion.[^14^, ^15^, ^16^, ^17^, ^18^, ^19^, ^20^]

Many Pt^II^ complexes, the most prominent being cisplatin, exhibit high anti‐proliferative activity.[^21^, ^22^, ^23^] This holds also true for TSC and related Pt^II^ complexes,[^10^, ^11^, ^12^, ^13^, ^23^, ^24^, ^25^, ^26^, ^27^, ^28^, ^29^] which affect even cisplatin resistant cells. It is assumed that they act with high activity by a different mode of action than cisplatin.[^12^, ^24^, ^25^]

However, although promising, metal complexes are generally only less developed as pharmaceutics owing to their poor water solubility and limited cellular uptake.^[30]^ One way to overcome these issues is the combination of metal complexes with bioactive peptides, which has gained considerable attention during the last decade.[^31^, ^32^, ^33^, ^34^, ^35^, ^36^, ^37^, ^38^, ^39^, ^40^, ^41^] In this context, so‐called cell‐penetrating peptides (CPPs) are promising tools to deliver metal complexes into various types of cells.[^41^, ^42^, ^43^, ^44^, ^45^, ^46^, ^47^, ^48^, ^49^] For instance, the Pt^IV^ analogue of oxaliplatin was successfully conjugated to the CPP Tat(48–58) protein providing increased water solubility and bioavailability.[^23^, ^39^, ^40^]

**Scheme 1:**
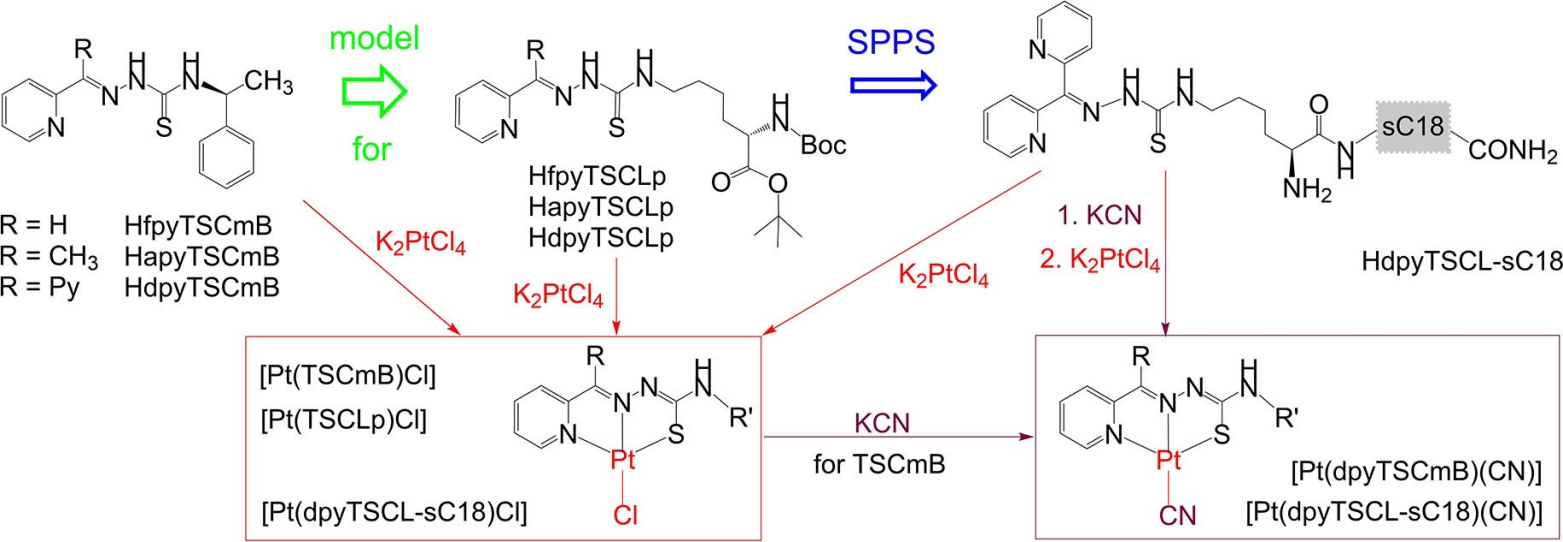
Compounds in this study and their nomenclature.

Herein, we present the synthesis of three chiral (*S*)‐*N*4‐(α‐methylbenzyl) thiosemicarbazone (HTSCmB) protoligands (ligand precursors prior to deprotonation upon coordination) and their lysine derivatives (HTSCL and HTSCLp). Moreover, the lysine‐tagged di‐2‐pyridylketone TSC (HdpyTSCL) was successfully coupled to the recently described cell‐penetrating peptide sC18 (Gly−Leu−Arg−Lys−Arg−Leu−Arg−Lys−Phe−Arg−Asn−Lys−Ile−Lys−Glu−Lys−NH_2_),[^40^, ^41^, ^42^, ^43^, ^44^, ^45^, ^46^, ^47^, ^48^] by solid‐phase peptide synthesis (HdpyTSCL‐SC18; Scheme 1). We discuss the complex formation with Pt^II^ and their detailed characterisation using NMR, MS, UV/Vis absorption spectroscopy, electrochemical methods and DFT calculations. Finally, for selected compounds we present details about their stability in biological conditions, and their anti‐proliferative activity towards human cancer and noncancerous cells.

## Results and Discussion

### Syntheses of the HTSCmB protoligands, lysine derivatives HTSCLp, and the conjugate HdpyTSCL‐SC18

**Scheme 2:**
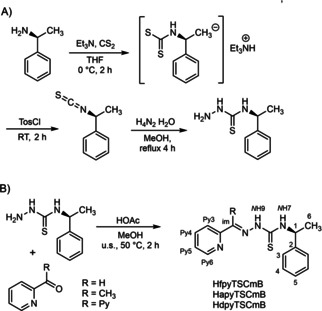
Multistep synthesis of the (*S*)‐*N*4‐(α‐methylbenzyl)thiosemicarbazone ligands (HTSCmB) with NMR nomenclature.

**Scheme 3:**
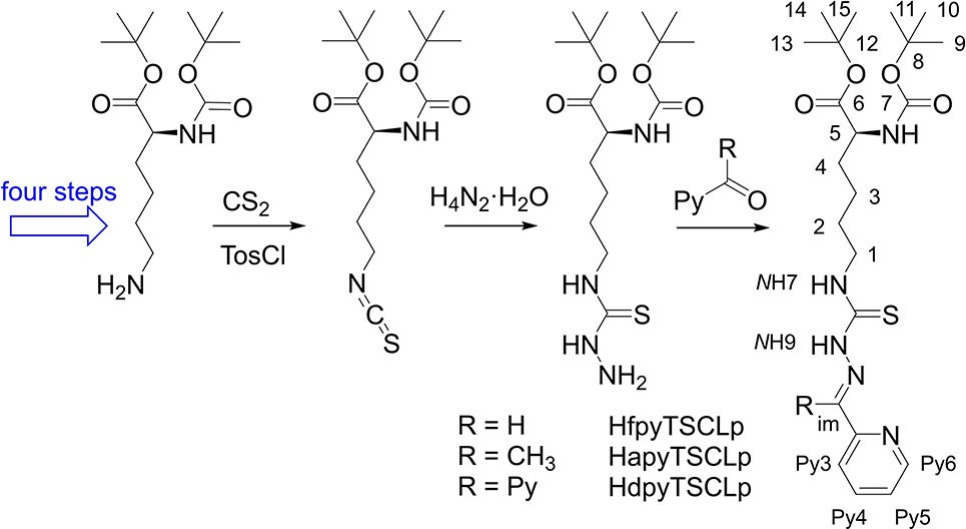
Synthesis of the protected lysine HTSCLp ligands (with NMR nomenclature).

*S*‐Benzyl methylamine isothiocyanate was synthesised by reacting the amine with CS_2_ followed by a tosylchloride mediated decomposition derived from a method reported by Wong et al.^[50]^ (Scheme 2A, details in the Experimental Section in the Supporting Information). Upon reaction with hydrazine, the thiosemicarbazide was obtained in excellent yields (99 %). The thiosemicarbazide was then reacted with three different N‐heterocyclic carbonyls (Scheme 2B) to yield the three HTSCmB derivatives (analytical details and characterisation including single‐crystal XRD in the Supporting Information). The HTSCmB molecules were soluble in solvents like THF, MeCN and MeOH but not in H_2_O.

l‐Lysine was converted into *tert*‐butyl‐(*tert*‐butoxycarbonyl)‐l‐lysine (Scheme 3) in four‐steps in an overall yield of 52 % using using standard procedures (Scheme 3; details see the Supporting Information). The amino function of the boc‐protected lysine was converted into the isothiocyanate which was then reacted with hydrazine hydrate yielding the corresponding thiosemicarbazide. Condensation with the pyridine carbonyls yielded the HTSCLp protoligands (Lp=protected lysine) in overall yields of 23, 36, and 37 %, respectively (Scheme 3).

**Scheme 4:**
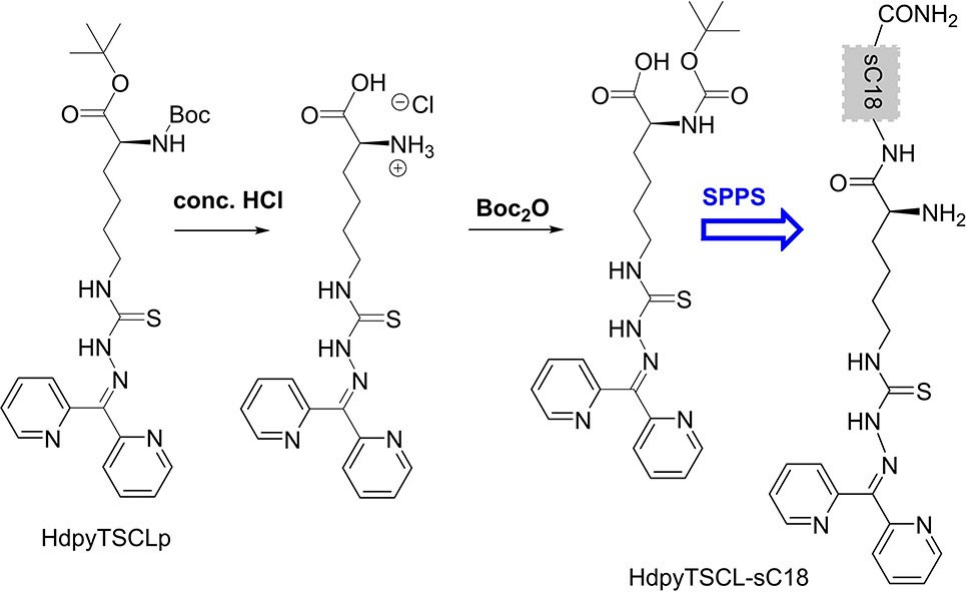
Synthesis of the HdpyTSCL‐sC18 peptide conjugate from HdpyTSCLp (SPPS: solid‐phase peptide synthesis).

Then, the Boc‐protected 2‐(2‐pyridyl)thiosemicarbazone‐lysine derivative HdpyTSCLp was prepared for peptide coupling using a sequence of de‐protection and re‐protection steps (Scheme 4, details in the Supporting Information). Meanwhile, sC18 was synthesised by solid‐phase peptide synthesis (SPPS) as previously described.^[51]^ Subsequently, HdpyTSCL was coupled to the *N* terminus of sC18 using a mixture of HATU/DIPEA (O‐(7‐azabenzotriazol‐1‐yl)‐*N,N,N′,N′*‐tetramethyluronium‐hexafluorphosphate/*N,N*‐diisopropyl‐ethyl‐amine) as activating reagents. Finally, the resulting conjugate was cleaved from the resin with concentrated trifluoroacetic acid and purified, yielding HdpyTSCL‐sC18 in high purity of >95 % (Scheme 4). HdpyTSCL‐sC18 was characterised using HPLC and ESI‐MS(+) (Figure S1 in the Supporting Information).

### Synthesis and characterisation of the Pt^II^ complexes

In a next set of reactions, we let react all of the different protoligands HTSCmB and HTSCLp with K_2_PtCl_4_ using either organic solvents or aqueous solutions, respectively. Notably, the complexes [Pt(TSC)Cl] formed in very short reaction times and excellent yields.

#### Syntheses and characterisation of the “model” complexes [Pt(fpyTSCmB)Cl], [Pt(apyTSCmB)Cl], [Pt(dpyTSCmB)Cl] and [Pt(dpyTSCmB)(CN)]

**Figure 1:**
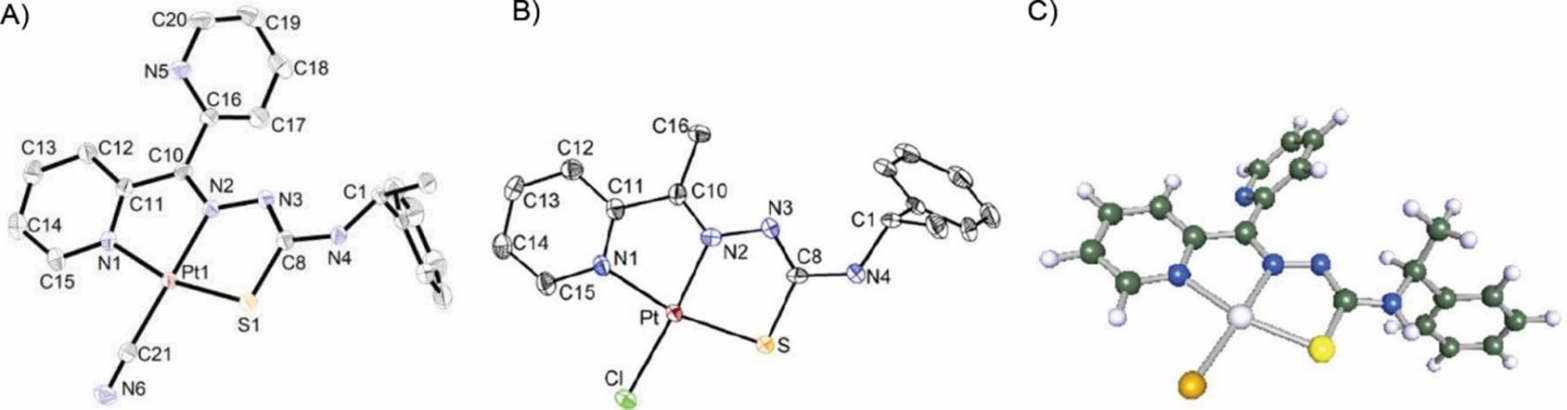
Molecular structures of A) [Pt(dpyTSCmB)(CN)] (one of two independent molecules) and B) [Pt(apyTSCmB)Cl] from single‐crystal XRD; atoms shown with 50 % probability and H atoms omitted for clarity. C) DFT‐calculated molecular structure of [Pt(dpyTSCmB)Cl] at the B3LYP def2‐TZVP level (C,H,N,S,Cl) and the B3LYP LANL2DZ level with ecp60 Hay & Wadt (Pt).[^52^, ^53^]

The three [Pt(TSCmB)Cl] complexes (Schemes 1 and 2) were obtained from MeCN reaction mixtures in about 30 min with yields ranging from 93 to 97 %. The CN complex was synthesised in 81 % yield after workup through mixing a MeCN solution of the Cl derivative with an aqueous solution of KCN. NMR data and the crystal structure of the complexes [Pt(apyTSCmB)Cl] and [Pt(dpyTSCmB)(CN)] (two independent molecules in the unit cell) (Figure 1) proved a square planar coordination of Pt^II^ through the deprotonated TSC ligand (N^N^S_thiolate_) and a chlorido or cyanido coligand, respectively. The two experimental structures show C−S bond lengths in the range of a single bond, whereas the C8−N3 lengths lie in the typical range of a double bond (Table 1) in line with already reported data and related structures.[^10^, ^24^, ^25^, ^27^, ^29^] A closer look at the angles around Pt^II^ revealed distorted planar coordination with almost perfect Cl/C21−Pt−N2 angles of about 175 and 176°. However, the N1−Pt−N2 and N2−Pt−S bite angles of the five‐membered chelates, which are markedly smaller than 90°, resulted in S−Pt−N1 angles of 166 and 165°. Furthermore, trans influence of the stronger CN^−^ co‐ligand compared with Cl^−^ might be the reason for the elongated Pt−N2 bond length of about 2.00 Å in [Pt(dpyTSCmB)(CN)] (Table S20) compared with 1.966(5) found for the complex [Pt(apyTSCmB)Cl] (Table 1).

**Table 1:** Selected experimental and DFT‐calculated bond lengths and angles of [Pt(apyTSCmB)Cl].^[a]^

	Bond lengths [Å]			Angles [°]	
	XRD^[a]^	DFT^[b]^		XRD^[a]^	DFT^[b]^
Pt−Cl	2.3173(2)	2.34	N1−Pt−N2	80.2(2)	80.69
Pt−N1	2.055(6)	2.07	N2−Pt−S	85.75(2)	84.79
Pt−N2	1.966(5)	1.99	S−Pt−Cl	96.75(6)	98.52
Pt−S	2.2623(2)	2.33	Cl−Pt−N1	97.35(2)	96.01
C10−N2	1.293(9)	1.34	Cl−Pt−N2	175.23(1)	176.88
N2−N3	1.391(8)	1.34	N1−Pt−S	166.05(1)	165.32
N3−C8	1.317(1)	1.34			
C8−S	1.775(8)	1.77	Σ of angles Pt	360.05(3)	360.01

[a] From single‐crystal XRD data measured at 100(2) K, solved in orthorhombic *P*2_1_2_1_2_1_; full crystallographic and metric data are given in the Supporting Information. [b] DFT calculations at the B3LYP def2‐TZVP level (for C,H,N,S,Cl) and B3LYP LANL2DZ level with ecp60 Hay & Wadt (for Pt).[^51^, ^52^]

The experimental and DFT‐calculated metrical parameters of [Pt(apyTSCmB)Cl] were essentially the same (Table 1 and Supporting Information). Only the distances in the ligand backbone vary slightly between the experimental and theoretical values. This might be the result of overestimation of the conjugation of the TSC system. On the other hand the DFT optimised structure represents the geometry in the gas phase, while the XRD data has been obtained from the crystalline solid in which bond lengths and angles might be modified by crystal packing. From this we concluded that suitable basis‐sets to model the Pt complexes were found.

**Figure 2:**
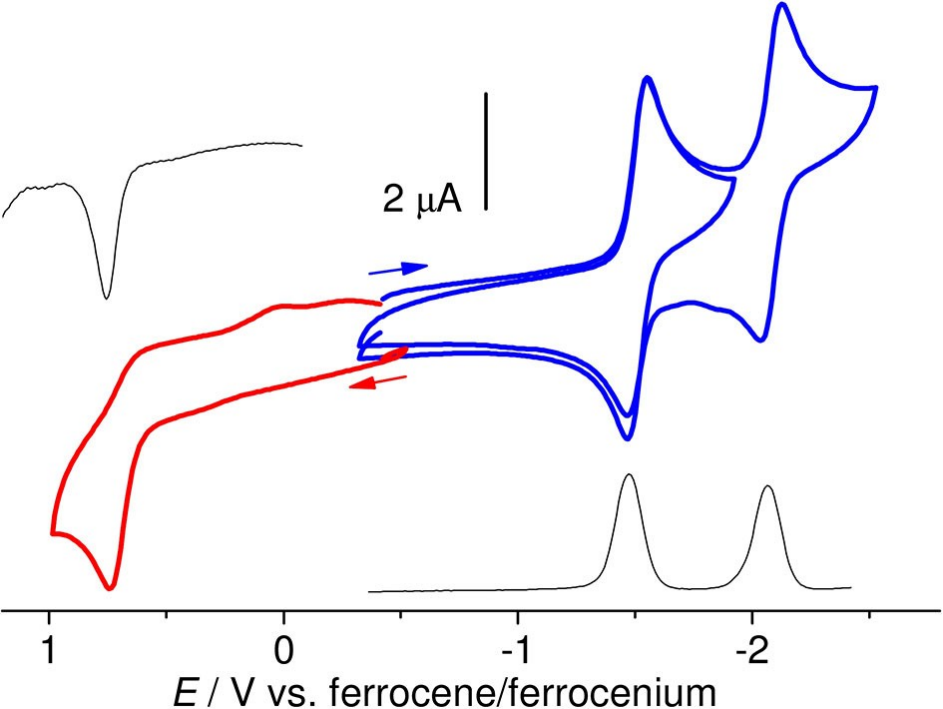
Cyclic voltammetry of [Pt(dpyTSCmB)Cl] in 0.1 m *n*Bu_4_NPF_6_/MeCN solution at a 100 mV/s scan rate. Square‐wave voltammograms in black, cathodic CV waves in blue, anodic waves in red.

Cyclic voltammograms (CV) of [Pt(dpyTSCmB)Cl] recorded in MeCN (Figure 2) showed two fully reversible one‐electron reduction waves at *E*_1/2_=−1.6 and −2.25 V, and a further irreversible wave at *E*_pc_=−2.9 V (not shown). On the anodic scan an irreversible oxidation is observed at *E*_pa_=0.74 V. For the apy and fpy derivatives very similar values were observed (Figure S16–S18 and Table S24). For the [Pt(dpyTSCmB)(CN)] complex the oxidation is markedly anodically shifted (+0.23 V) compared with the Cl derivative, while the reduction potential is only slightly shifted (+0.1 V; Figure S18 and Table S24). When taking a conversion factor of 0.62 V,^[54]^ the two dpyTSCmB complexes are stable within a range from 0.1 (0.33 for CN) to approx. −2.1 V vs. NHE. In conclusion, the complexes were stable in a range of more than 2 V, giving hope for adequate redox stability in water and other biological conditions.

**Figure 3:**
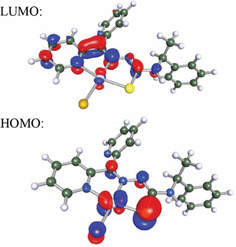
DFT‐calculated composition of LUMO and HOMO for [Pt(dpyTSCmB)Cl] at the B3LYP def2‐TZVP level (C,H,N,S,Cl) and B3LYP LANL2DZ level with ecp60 Hay & Wadt (Pt). Iso‐surface level at 0.06.

We have seen that for [Pt(dpyTSCmB)Cl] the DFT‐calculated highest occupied molecular orbital (HOMO) was located on the Pt^II^ atom (d_*xy*_ orbital) and on the thiolate S atom and also obtained a minor contribution from the chlorido co‐ligand (Figure 3). The electrochemical oxidation at 0.74 V can thus be attributed to a Pt^II^/Pt^III^ redox pair with contributions from a thiolate(S^−^)/thiyl‐radical(S^•^) pair. This is completely consistent with the observed anodic shift when changing the weak Cl^−^ co‐ligand for the strong CN^−^. The lowest unoccupied molecular orbital (LUMO) had essentially the character of the lowest pyridine π* orbital extending into the TSC backbone (Figures 3 and S19). Thus, the first electrochemical reduction was directed into this orbital (ligand‐centred).

**Figure 4:**
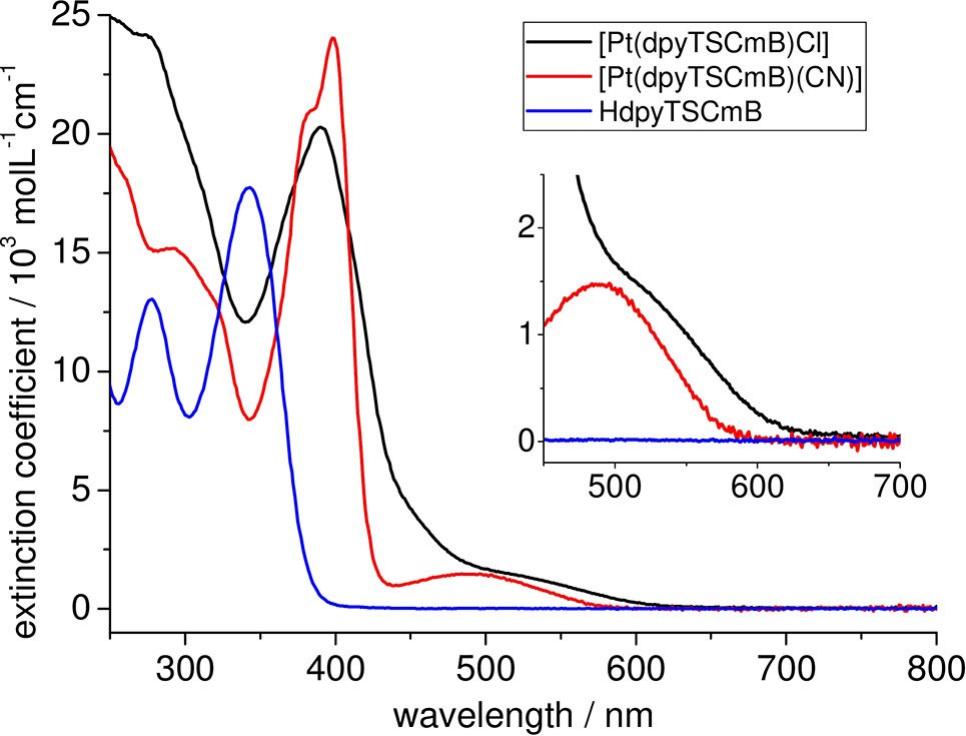
UV/Vis absorption spectra of HdpyTSCmB (blue), [Pt(dpyTSCmB)Cl] (black), and [Pt(dpyTSCmB)(CN)] (red) in MeCN.

The UV/Vis absorption spectra of the four Pt^II^ TSCmB complexes are all quite similar (Figures 4, S13 and S14, data in Table S23). We assign the long‐wavelength absorption to metal(d)‐to‐ligand(π*) (MLCT) excited states based on our DFT calculations, while the UV bands were of π‐π* type and were also observed in the protoligands HTSCmB (Figures 4, S13 and S14, Table S23).

When the comparing the complex [Pt(dpyTSCmB)(CN)] with the Cl derivative, we recognised for the CN complex a slight blue‐shift for the long‐wavelength MLCT band and the absorptions at around 400 and 320 nm received changes in intensity and resolution (Figure 4). We assigned these changes to the stronger σ‐donating and π‐accepting CN ligand stabilising the metal‐centred HOMO. Notably, this data was consistent with the observed markedly higher oxidation and slightly higher reduction potentials, as well as the DFT results.

**Figure 5:**
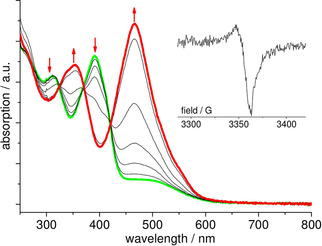
UV/Vis absorption spectra recorded during the first reduction of [Pt(dpyTSCmB)Cl] in 0.1 m *n*Bu_4_NPF_6_/MeCN solution (0.05 V increments). Inset: X‐band EPR spectrum recorded during the first reduction of [Pt(dpyTSCmB)Cl] (cathodic reduction in 0.1 m *n*Bu_4_NPF_6_/MeCN solution at 298 K, measured at 110 K).

Upon cathodic reduction of [Pt(dpyTSCmB)Cl] in 0.1 m *n*‐Bu_4_NPF_6_/MeCN solution, a narrow unstructured EPR signal of approximate axial symmetry was observed in glassy frozen solution at 110 K (Figure 5, inset). The missing hyperfine splitting to the ^195^Pt isotope (*I*=1/2 , 33.8 % nat. abundance) pointed to a negligible contribution of Pt orbitals to the singly occupied molecular orbital (SOMO). The *g*_⊥_ and *g*_*‖*_ values at 2.0066 and 2.0079 supported the ligand centred character.[^54^, ^55^, ^56^, ^57^, ^58^] At ambient *T* no signal was observed. Efforts to get EPR spectra of oxidised species were not successful.

Spectro‐electrochemical UV/Vis absorption measurements on cathodic scans showed the growth of a strong and broad absorption band centred at 490 nm for the first reduction step obscuring the long‐wavelength MLCT band (Figure 5). Furthermore, the two π–π* absorption bands at 400 and 330 nm moved to 380 and 260 nm, respectively. Importantly, this process is fully reversible; the spectrum of the parent compound (green line in Figure 5) can be fully recovered upon anodic back‐scan. The spectroscopic changes are strongly indicative for a singly reduced pyridine moiety indicating a ligand‐centred reduction, and were thus, fully in line with our DFT calculations and related examples.[^55^, ^56^, ^57^, ^58^, ^59^, ^60^]

The second reduction process shows the formation of new long‐wavelength bands between 550 and 800 nm as well as a hypochromic shift of the bands at 490 and 340 nm (Figure S20) and turns out to be not reversible on the timescale of this experiment (5–20 min).

#### Syntheses and characterisation of the complexes [Pt(fpyTSCLp)Cl], [Pt(apyTSCLp)Cl], and [Pt(dpyTSCLp)Cl] containing protected lysine

**Figure 6:**
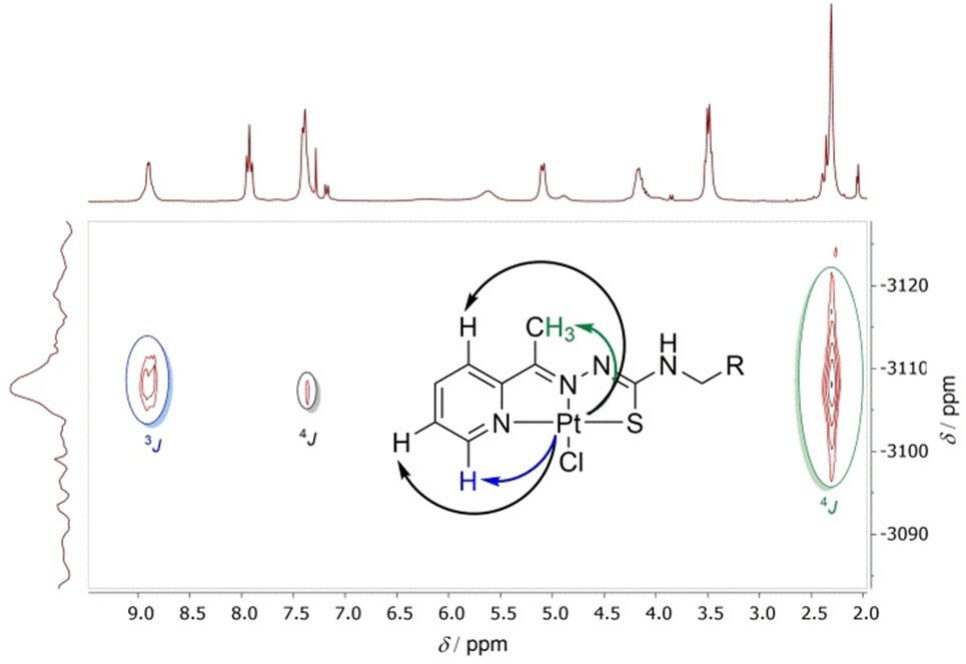
300 MHz ^195^Pt, ^1^H HMBC NMR spectra of [Pt(apyTSCLp)Cl] in [D_6_]DMSO.

The reaction of the Boc‐protected HTSCLp protoligands and K_2_PtCl_4_ was carried out in a mixture of MeCN and water. After 2 h of stirring at ambient temperature and column chromatographic purification the three complexes were obtained in 75–87 % yield. Good solubility of [Pt(apyTSCLp)Cl] in organic solvents allowed ^195^Pt,^1^H HMBC NMR experiments showing several ^x^J_Pt‐H_ couplings between Pt and the TSC ligand backbone (Figure 6, Table S22). The complexes were not soluble in water.

As the UV/Vis absorption spectra (Figure S13, Table S23) and the redox behaviour (Figure S18, Table S24) of the [Pt(TSCLp)Cl] complexes are very similar to those of the α‐methyl benzyl (TSCmB) derivatives, we supposed the same complex entity [Pt(N^N^S)Cl]. Moreover, we did not observe any influence on the photophysical and electrochemical properties of the complexes when changing the side residue from α‐methyl benzyl to protected lysine.

#### Syntheses and characterisation of the Pt conjugate complexes [Pt(dpyTSCL‐sC18)Cl] and [Pt(dpyTSCL‐sC18)(CN)]

**Figure 7:**
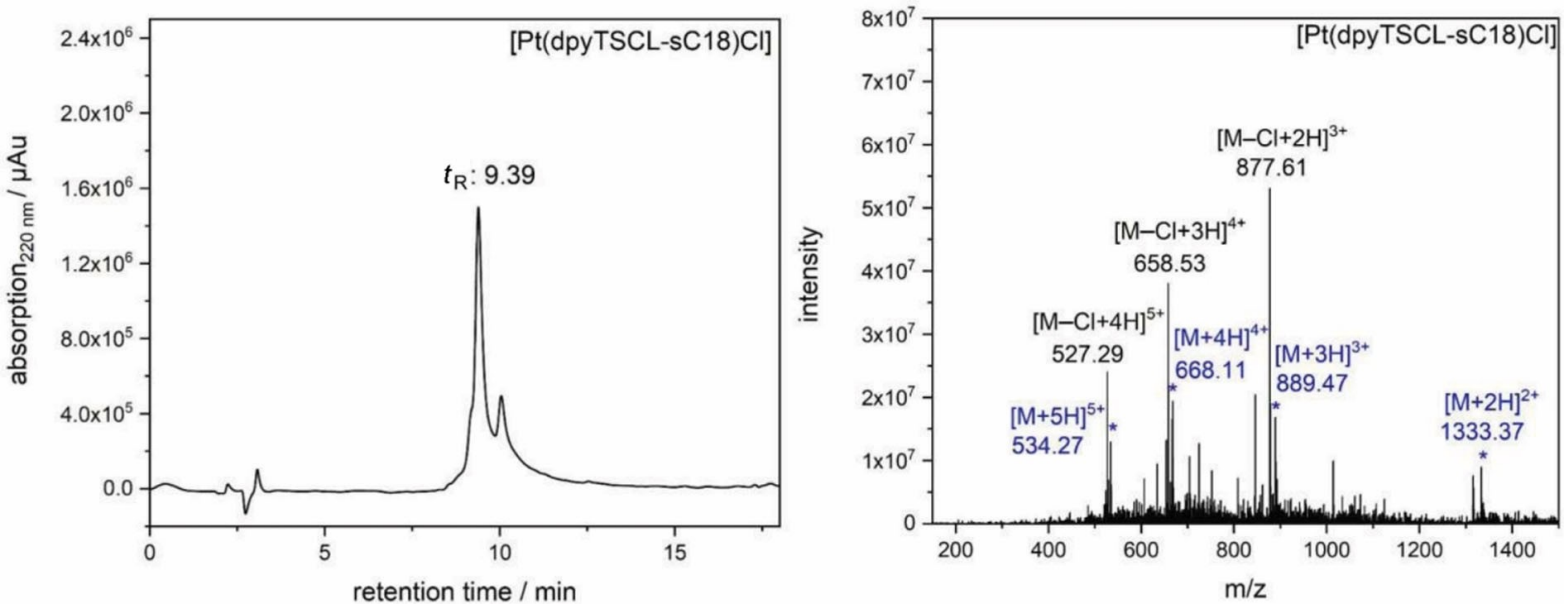
HPLC analysis (left) and ESI‐MS(+) analysis (right) of [Pt(dpyTSCL‐sC18)Cl] after desalting. Detected masses for [*M*+*n*H]^*n*+^ are labelled in blue, the calculated mass for [Pt(dpyTSCL‐sC18)Cl] is 2666.40 (found: 2666.26). Detected masses for [*M*−Cl+*n*H]^(*n*+1)+^ are labelled in black representing the complex [Pt(dpyTSCL‐sC18)]^+^ without coordinated chloride. For [*M*−Cl]^+^ 2631.43 was calculated, 2630.47 was found.

The conjugate protoligand HdpyTSCL‐sC18 was treated with K_2_PtCl_4_ in H_2_O for 2 h at ambient T forming the complex [Pt(dpyTSCL‐sC18)Cl]. The crude complex was desalted using a C18ec cartridge (Chromafix C18ec, Macherey‐Nagel), evaporated from solvent and lyophilised. HPLC analysis and ESI‐MS confirmed quantitative product formation, while unreacted HdpyTSCL‐sC18 was not observed (Figure 7).

The ESI‐MS analysis showed the target complex [Pt(dpyTSCL‐sC18)Cl] alongside with the species [Pt(dpyTSCL‐sC18)]^+^ having lost the chloride co‐ligand. The UV/Vis absorption spectrum of the product proved the quantitative formation of a [Pt(N^N^S)Cl] complex as shown before for the other TSC ligand derivatives (Figures S13–S15, S21). Interestingly, changing from organic solvents to water did not lead to alterations of the long‐wavelength MLCT band energy. The observed energy fitted well into the solvatochromic behaviour recorded for the [Pt(dpyTSCLp)Cl] complexes which is in good correlation to the Reichardt E_T_(30) solvent parameters^[61]^ (Figure S22).

**Figure 8:**
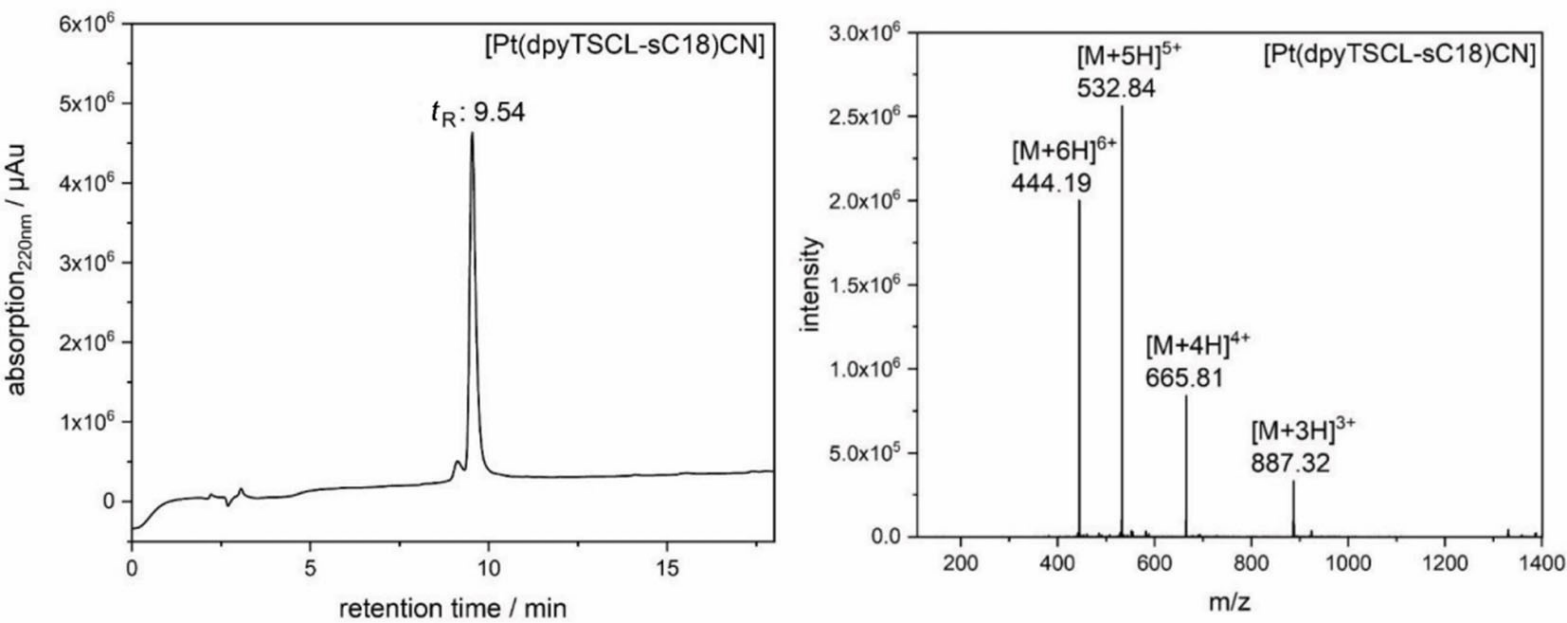
HPLC analysis and ESI‐MS(+) analysis of [Pt(dpyTSCL‐sC18)(CN)] conjugate after desalting. The sample was recorded using a linear gradient from 10–60 % MeCN in H_2_O (incl. 0.1 % trifluoroacetic acid) within 15 min. The identified molecular ions agree well with the calculated molecular weight (calcd: 2657.44 g/mol; exp.: 2659.13 g/mol).

Complex [Pt(dpyTSCL‐sC18)(CN)] was synthesised in 2 h from KCN, HdpyTSCL‐sC18 and K_2_PtCl_4_ in quantitative yield. HPLC analysis and ESI‐MS(+) of the purified complex showed exclusively the product (Figure 8). In this case, the species [Pt(dpyTSCL‐sC18)]^+^ having lost the anionic co‐ligand was not observed. This observation underlined the stronger binding of the CN^−^ co‐ligand to Pt compared to Cl^−^.

The stability of [Pt(dpyTSCL‐sC18)Cl] and [Pt(dpyTSCL‐sC18)(CN)] was tested for 72 h in foetal bovine serum (FBS) containing medium using UV/Vis absorption spectroscopy (Figure S23). The long‐wavelength MLCT band of the complexes was used as an indicator for structural integrity. During the first hours, a decay of about 2 % of the [Pt(dpyTSCL‐sC18)Cl] complex was observed, but no further changes were detected (Figure S23). Compared to this, a loss of 19 % signal intensity was observed for [Pt(dpyTSCL‐sC18)(CN)] (Figure S23).

### Anti‐proliferative activity

**Figure 9:**
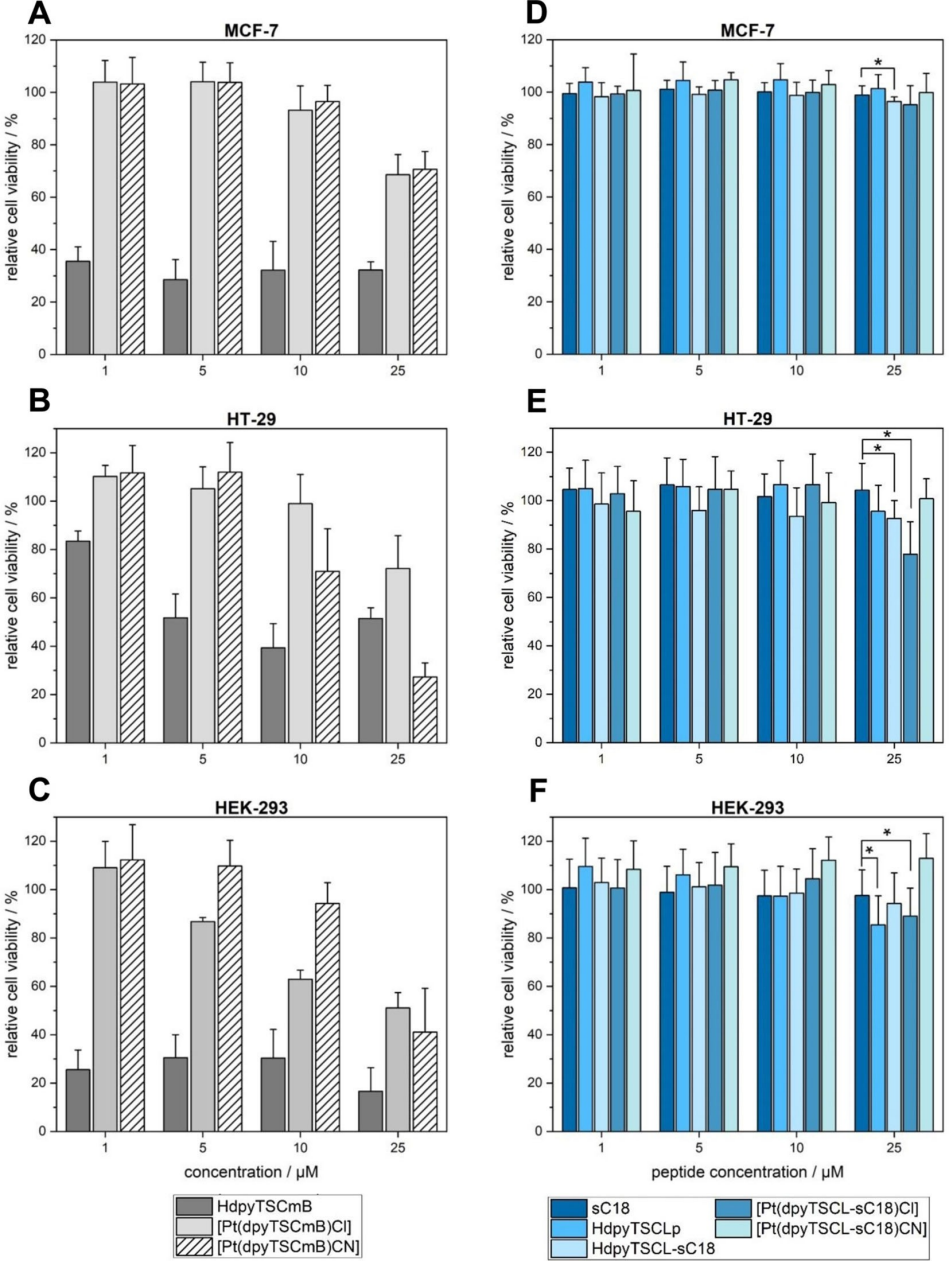
Anti‐proliferative activity of HdpyTSCmB, [Pt(dpyTSCmB)Cl] and [Pt(dpyTSCmB)(CN)] against A) MCF‐7, B) HT‐29 and C) HEK‐293 cells after 72 h of incubation with different concentrations of the compounds. Anti‐proliferative activity of sC18, HdpyTSCLp, HdpyTSCL‐sC18, [Pt(dpyTSCL‐sC18)Cl], and [Pt(dpyTSCL‐sC18)(CN)] against D) MCF‐7, E) HT‐29 and F) HEK‐293. Cells treated with 70 % EtOH served as a positive control. Data were normalised to untreated cells (100 % viability). Shown are mean ± SD values of three independent experiments, each performed in triplicate.

Next, we investigated the anti‐proliferative activity of several of our TSC ligands, as well as their Pt^II^ complexes (Figure 9). As control we included Dp44mT (di‐2‐pyridylketone‐4,4,–dimethyl‐3‐thiosemicarbazone, Figure S24), which is structurally very similar to HdpyTSCmB.[^19^, ^20^, ^62^, ^63^, ^64^, ^65^] We chose two different cancer cell lines, namely breast adenocarcinoma MCF‐7 (Michigan Cancer Foundation 7) and colorectal carcinoma HT‐29 cells, since those have been already used in former experiments, thus allowing for comparing the activity of our novel compounds with recent results from our own studies and literature.[^44^, ^45^, ^46^, ^66^, ^67^, ^68^, ^69^, ^70^] HEK‐293 (human embryonic kidney) cells were used as non‐cancerous control cell line (Figure 9).

HdpyTSCmB exhibited quite strong anti‐proliferative activity against HT‐29 cells, while MCF‐7 cells were less harmed (Figure 9A, B). However, also HEK‐293 cells were strongly affected (Figure 9C) letting assume only less selectivity of this compound. Overall, the activity was in the range of Dp44mT[^72^, ^73^, ^74^, ^75^] and DpC (di‐2‐pyridylketone‐4‐cyclohexyl‐4‐methyl‐3‐thiosemicarbazone).[^18^, ^19^, ^20^] Notably, the Pt complexes [Pt(dpyTSCmB)Cl] and [Pt(dpyTSCmB)(CN)] exhibited reduced activity compared with the ligand alone. Only at high concentrations of 10–25 μM markedly reduced cell viability was observed. Thereby it seemed that the CN complex was more active against HT‐29 cells, while the Cl derivative demonstrated higher activity against HEK cells. Interestingly, there was no difference detectable when incubating the two complexes with MCF‐7 cells. Recently, it was hypothesised that TSC molecules coordinate in the cell to redox‐active metals such as Fe or Cu, and that these formed complexes would lead to the production of reactive oxygen species, damaging the cell.[^8^, ^15^, ^20^, ^63^, ^64^, ^65^, ^71^, ^72^, ^73^, ^74^, ^75^] In fact, this would explain also our observation of higher activity of the free ligands compared with the Pt complexes. Obviously, Pt^II^ is only a poor substitute to these redox‐active metals that are known to quickly react with H_2_O_2_ forming hydroxyl radicals in Fenton‐type reactions.[^63^, ^64^, ^65^, ^71^, ^72^, ^73^, ^74^, ^75^] Considering that we have measured high electrochemical stability for both Pt model complexes [Pt(dpyTSCmB)Cl] and [Pt(dpyTSCmB)(CN)], particularly against reduction, further substantiates our assumption.

Interestingly, no or only minor decrease in cell viability was determined after incubating all other compounds for 72 h with the three cell lines (Figure 9D–F). In fact, the noncomplexed HdpyTSCL‐sC18 showed a significant anti‐proliferative activity compared to the parent peptide sC18 when applied at 25 μm to MCF‐7 (*p*=0.0449) or HT‐29 (*p*=0.0226) cells. Surprisingly, the ligand HdpyTSCLp alone only exhibited significant toxicity towards HEK‐293 cells (*p*=0.0058), also when at the highest concentration studied. For the corresponding platinum complexes, exclusively [Pt(dpyTSCL‐sC18)Cl] was determined to significantly reduce the cell viability of HT‐29 (*p*=0.0200) and HEK‐293 (*p*=0.0409) cells when added at 25 μm. That sC18 did not display cytotoxic effects agrees to already published results and demonstrates again that sC18 is well tolerated.[^43^, ^47^, ^51^] For the other tested substances including the HdpyTSCL‐sC18 ligand and the complex [Pt(dpyTSCL‐sC18)Cl], we found this more surprising. However, since we have already demonstrated that sC18 is a highly efficient carrier for various cargos, including also metal complexes,[^42^, ^43^, ^44^, ^45^, ^46^, ^47^, ^48^, ^51^] we were very confident that the whole conjugate entered the cells. The results let rather conclude that there might be a dramatic influence of the linker structure that bridges the TSC with the carrier molecule (in this case sC18). This hypothesis is probably proven by the observation that we did also not detect any activity of HdpyTSCLp being structurally highly divers to recently reported toxic binuclear Pt^II^Cl complexes containing triazole‐bridged bisthiosemicarbazone ligands.^[10]^ In addition, we have already seen similar effects when coupling Rh^III^ polypyridyl complexes to sC18.^[44]^ Generally, the potency of such metal complexes is often dependent on specific properties like DNA intercalation. For sC18 conjugates it is already known that they are mainly taken up by endocytotic pathways, restricting efficient cytosolic release, and thus, transfer of the cargo to its final target.[^43^, ^47^] One way to overcome this limitation is to include specific proteolytic cleavage sites within the ligand to peptide structure, for example, for cathepsin B. This protease is abundant in the lysosomes and may induce enhanced endosomal release.^[42]^

On the other side, the good tolerability of the novel Pt^II^ conjugates paves the way for future studies, in which we will use these and related Pt‐TSC‐peptide conjugates for late‐stage radiolabelling with Pt radionuclides, such as ^189^Pt, ^191^Pt, ^193m^Pt, and ^195m^Pt that are very interesting candidates for Auger‐electron radionuclide therapy.[^76^, ^77^, ^78^, ^79^] The advantage of this method is the emission of low energy electrons which are able to ionise material in a very confined space, thus reducing radiation damage to healthy cells.[^80^, ^81^] Tagged by a ligand containing biological information, the radionuclide can be transported to a desired location or cell, thus focussing the decay and cell damage in a certain area (targeted radiotherapy). The facile, rapid, and stable binding of Pt^II^ from a simple source such as K_2_PtCl_4_ together with the observed high stability and virtual non‐toxicity make our systems very suitable for late‐stage labelling.

## Conclusions

l‐Lysine‐based thiosemicarbazone (TSC) ligands were successfully conjugated to the cell‐penetrating peptide sC18, using standard solid‐phase peptide synthesis. The overall synthesis yield for the peptide conjugate is 10 % over 10 steps. The tridentate N^N^S binding site in of the pyridine‐based TSC bound rapidly and quantitatively to Pt^II^ forming Pt chlorido complexes of the type [Pt(TSC)Cl]. This was shown for dpyTSCL‐sC18 as well as for model compounds with *N*4‐(α‐methylbenzyl) termination (TSCmB) or protected l‐lysinate (TSCLp). Increasing the terminating chain from dpyTSCmB to dpyTSCL‐sC18 drastically enhanced their solubility. The sC18‐conjugated Pt complexes [Pt(dpyTSCL‐sC18)X] with X=Cl or CN were soluble in solvents ranging from rather non‐polar CH_2_Cl_2_ to water. The uncoordinated HdpyTSCmB showed antiproliferative activity towards the human cancer cell lines MCF‐7 and HT‐29 as well as the noncancer cell line HEK‐293. For the two non‐conjugated Pt complexes containing this ligand [Pt(dpyTSCmB)X] (X=Cl or CN) the activity was markedly reduced and only for the HEK cells effective doses lower than 25 μm were found. Interestingly, none of the sC18‐conjugated compounds as well as HdpyTSCLp exhibited significant anti‐proliferative activities at lower concentrations. Future studies will be directed to get unequivocal proof that the conjugates have entered the cells.

In summary, we highlighted these nontoxic and biologically very stable Pt^II^ complexes as very interesting candidates for late‐stage labelling with the radioisotopes ^189^Pt, ^191^Pt, ^193m^Pt, or ^195m^Pt.

## Experimental Section

**Materials and syntheses**: The syntheses of the three HTSCmB protoligands and the Boc‐protected lysine derivatives HTSCLp are outlined in detail in the Supporting Information.

**Synthesis of the Pt complexes [Pt(TSCmB)Cl]. General description**: The protoligands HTSCmB and K_2_PtCl_4_ were dissolved in MeCN and stirred for 2 h at ambient temperature. The colour changes from yellow to dark red. Upon evaporation of the solvent a red solid forms, which was isolated by filtration.

**[Pt(fpyTSCmB)Cl]**: Yield: 119.9 mg (0.23 mmol, 97 %). C_15_H_15_ClN_4_PtS (513.90) C 35.06, H 2.94, N 10.90, S 6.24; found C 35.03, H 3.02, N 10.90, S 6.29 %. ^1^H NMR: (300 MHz, [D_6_]DMSO): *δ*=8.87 (d, 1H, *J*=8.0 Hz, *N*H), 8.70 (d, 1H, *J*=5.6 Hz, ^3^ *J*_PtH_=27 Hz, HPy6), 8.35 (s, 1H, ^3^ *J*_PtH_=12 Hz H_im_), 8.13 (t, 1H, *J*=7.7 Hz, HPy4), 7.70 (t, 1H, *J*=7.9 Hz, HPy5), 7.33 (d, 5H, *J*=5.9 Hz, H3,4,5), 7.25 (dd, 1H, *J*=6.1, 2.1 Hz, HPy3), 5.06 (quint, 1H, *J*=7.4 Hz, H1), 1.42 (d, 3H, *J*=7.1 Hz, H6) ppm (nomenclature as in Scheme 2) EI‐MS(+) m/z=514 [*M*]^+^, 478 [*M*−Cl]^+^.

**[Pt(apyTSCmB)Cl]**: Yield: 0.121 g (0.23 mmol, 96 %). Anal. calc. for C_16_H_17_ClN_4_PtS (527.93) C 36.40, H 3.25, N 10.61, S 6.07; found C 36.39, H 3.24, N 10.60, S 6.08 %. ^1^H NMR: (300 MHz, [D_6_]DMSO): *δ*=8.76 (m, 2H, *N*H7, ^3^ *J*_PtH_=16 Hz, HPy6), 8.16 (t, 1H, *J*=7.9 Hz, HPy4), 7.74 (d, 1H, *J*=8.1 Hz, HPy5), 7.68 (d, 1H, *J*=6.8 Hz, HPy3), 7.41‐7.29 (m, 4H, H3,4), 7.24 (m, 1H, H5), 5.13‐4.93 (m, 1H, H1), 2.26 (d, 3H, *J*=6.9 Hz, CH_3im_), 1.43 (d, 3H, *J*=7.0 Hz, H6) ppm. EI‐MS(+) m/z=528 [*M*]^+^, 492 [*M*−Cl]^+^.

**[Pt(dpyTSCmB)Cl]**: Yield: 0.131 g (0.22 mmol, 93 %). Anal. calc. for C_20_H_18_ClN_5_PtS (590.99) C 40.65, H 3.07, N 11.85, S 5.42; found C 40.66, H 3.08, N 11.88, S 5.41 %. ^1^H NMR: (300 MHz, [D_6_]DMSO): *δ*=9.02 (d, 1H, *J*=7.2 Hz, *N*H7), 8.84 (d, 1H, *J*_HH_=5.0 Hz, ^3^ *J*_PtH_=27 Hz, HPy6), 8.72 (d, 1H, *J*=4.9 Hz HPy’6), 8.06 (d, 1H, *J*=5.0 Hz, HPy4), 7.97 (t, 1H, *J*=4.9 Hz, HPy’4), 7.70 (t, 1H, *J*=7.4 Hz, HPy5), 7.60 (t, 1H, *J*=7.5 Hz, HPy’5), 7.45 (d, 1H, *J*=7.9 Hz, HPy3), 7.40‐7.15 (m, 5H, H3, H4, H5), 7.36 (d, 1H, *J*=7.9 Hz, HPy’3), 4.65 (quint, 1H, *J*=6.9 Hz, H1), 1.32 (d, 3H, *J*=7.0 Hz, 3H, H6) ppm. EI‐MS(+) *m*/z=591 [*M*]^+^, 555 [*M*−Cl]^+^.

**Synthesis of [Pt(dpyTSCmB)(CN)]**: 65 mg (0.11 mmol) of [Pt(dpyTSCmB)Cl] were dissolved in 5 mL MeCN. 20 mg (0.3 mmol) KCN were dissolved in 1 mL H_2_O of which 0.5 mL were added to the complex solution. The mixture rapidly changed colour from a dark red to bright red. After 2 h at room temperature, the solvent was removed and the crude mixture was purified by silica column chromatography (100 % EtOAc). A bright red/pinkish band was isolated containing the desired complex. Yield: 46 mg (0.081 mmol, 81 %). C_21_H_18_N_6_PtS (581.55). ^1^H NMR (300 MHz, [D_6_]DMSO): *δ*=9.19 (d, 1H, *J*=7.1 Hz, *N*H7), 8.85 (d, 1H, *J*=5.4 Hz, ^3^ *J*_PtH_=35 Hz, HPy6), 8.77 (d, 1H, *J*=4.9 Hz, HPy’6), 8.10 (t, 1H, *J*=8.0 Hz, HPy4), 7.99 (t, 1H, *J*=7.2 Hz, HPy’4), 7.66 (t, 1H, *J*=7.7, 1.8 Hz, HPy5’), 7.59 (t, 1H, *J*_HH_=7.8 Hz, HPy5), 7.49 (m, 1H, HPy’3), 7.44 (m, 1H, HPy3), 7.35‐7.19 (m, 3H, H4,5), 7.04 (d, 2H, *J*=8.1 Hz, H3), 4.77‐4.62 (m, 1H, H1), 1.34 (d, 3H, *J*=7.0 Hz, H6) ppm. EI‐MS(+) *m*/*z*=581 [*M*]^+^.

**Synthesis of the Pt complexes [Pt(TSCLp)Cl]. General description**: 1 equiv. of the ligand was dissolved in MeCN and added to a solution of 1 equiv. K_2_[PtCl_4_]. The colour changes from yellow to dark red. After 2 h the solvent was evaporated under reduced pressure and the residue was purified by column chromatography.

**[Pt(fpyTSCLp)Cl]**: Yield: 127 mg (0.18 mmol, 75 %). Anal. calc. for C_22_H_34_ClN_5_O_4_PtS (695.13) C 38.01, H 4.93, N 10.07, S 4.61; found C 38.02, H 4.95, N 10.09, S 4.60 %. ^1^H NMR: (300 MHz, [D_6_]DMSO): *δ*=8.72 (d, 1H, *J*=5.3 Hz, ^3^ *J*_PtH_=24 Hz, HPy6), 8.40 (s, 1H, ^3^ *J*_PtH_=12 Hz, H_im_), 8.30 (d, 1H, *J*=4.5 Hz, HPy3), 8.15 (t, 1H, *J*=8.2 Hz, HPy4), 7.71 (t, 1H, *J*=9.0 Hz, HPy5), 7.08 (d, 1H, *J*=7.8 Hz, *N*H7), 5.07 (d, 1H, *J*=8.3 Hz, NH), 3.75 (q, 2H, *J*=6.7 Hz, H1), 3.65‐3.55 (m, 1H, H5), 1.69‐1.47 (m, 4H, H3,4), 1.39 (m, 20H, H2,9,10,11,13,14,15) ppm (nomenclature as in Scheme 3). EI‐MS(+) *m*/*z* 695 [*M*]^+^, 659 [*M*−Cl]^+^, 465 [HfpyTSCLp]^+^.

**[Pt(apyTSCLp)Cl]**: Yield: 141 mg (0.2 mmol, 83 %). Anal. calc. for C_23_H_36_ClN_5_O_4_PtS (709.16) C 38.95, H 5.12, N 9.88, S 4.52; found C 38.98, H 5.12, N 9.87, S 4.55 %. ^1^H NMR: (300 MHz, CDCl_3_): *δ*=8.88 (d, 1H, *J*=5.5 Hz, ^3^ *J*_PtH_=14 Hz, HPy6), 7.90 (t, 1H, *J*=7.6 Hz, HPy4), 7.78 (d, 1H„ *J*=9.0 Hz, HPy3), 7.69 (d, 1H, *J*=6.7 Hz, HPy5), 7.07 (d, 1H, *J*=8.3 Hz, *N*H7), 4.24‐4.01 (m, 1H, H5), 3.47 (q, 2H, *J*=6.7 Hz, H1), 2.29 (s, 3H, CH_3_im), 1.86‐1.52 (m, 6H, H2,3,4), 1.45 (s, 9H, H13,14,15), 1.43 (s, 9H, H9,10,11) ppm. ^1^H/^195^Pt‐HMBC NMR: (300 MHz, CDCl_3_): *δ*=−3108 (^3^ *J*_Pt‐HPy6_=14 Hz, ^4^ *J*_Pt‐HCH3_ obscured, ^5^ *J*_Pt‐HPy4_=21 Hz) ppm. HR‐ESI‐MS(+): *m*/*z* calculated [*M*+Na]^+^: 732.17167; found: 732.16964. FTIR‐ATR: ν [cm^−1^] 3853 (w), 3675 (m), 3424 (w), 2987 (s), 2901 (s), 2359 (w), 1715 (m), 1558 (w), 1507 (m), 1455 (m), 1406 (m), 1394 (m), 1380 (m), 1250 (m), 1230 (m), 1154 (m), 1075 (s), 1066 (s), 1056 (s), 1028 (m), 891 (w), 789 (w), 706 (w), 642 (w), 626 (w), 618 (m), 606 (m), 591 (w), 581 (w), 570 (w), 560 (w), 546 (w), 540 (w), 526 (w).

**[Pt(dpyTSCLp)Cl]**: Yield: 162 mg (0.21 mmol, 87 %). Anal. calc. for C_27_H_37_ClN_6_O_4_PtS (772.22) C 42.00, H 4.83, N 10.88, S 4.15; found C 42.02, H 4.82, N 10.90, S 4.15 %. ^1^H NMR: (300 MHz, CDCl_3_): *δ*=8.90 (d, 1H, *J*=5.6 Hz, ^3^ *J*_PtH_=15 Hz, HPy6), 8.75 (d, 1H, *J*=4.7 Hz, HPy′6), 8.08 (td, 1H, *J*=7.9, 1.6 Hz, HPy4), 7.98 (td, 1H, *J*=7.8, 1.7 Hz, HPy′4), 7.85 (d, 1H, *J*=7.9 Hz, HPy3), 7.73 (td, 1H, *J*=7.9, 4.1 Hz, HPy′5), 7.53 (m, 1H, HPy5), 7.43 (d, 1H, *J*=8.1 Hz, HPy’3), 7.06 (d, 1H, *J*=7.7 Hz, *N*H7), 4.03 (quint, 1H, *J*=7.1 Hz, H5), 3.16 (d, 2H, *J*=6.4 Hz, H1), 1.49 (m, 6H, *J*=7.4 Hz, H2,3,4), 1.38 (d, 18H, *J*=3.0 Hz, H9,10,11,13,14,15) ppm.HR‐ESI‐MS(+): *m*/*z* calculated [*M*+Na]^+^: 795.18271; found: 795.18364.

**Synthesis of HdpyTSCL‐sC18**: sC18 (Gly‐−Leu−Arg−Lys−Arg−Leu−Arg−Lys−Phe−Arg−Asn−Lys−Ile−Lys−Glu−Lys−NH_2_) was synthesised as previously described.^[51]^ HdpyTSCL (15 μmol, 2 equiv.) was coupled manually to the resin using 2 equiv. HATU (*O*‐(7‐azabenzotriazol‐1‐yl)‐*N,N,N′,N*′‐tetramethyluronium hexafluorophosphate) and 2 equiv. DIPEA (*N,N*‐diisopropylethylamine) in DMF for 2 h at RT. The resulting HdpyTSCL‐sC18 conjugate was cleaved from the resin with trifluoroacetic acid/triisopropylsilane/H_2_O (95 : 2.5 : 2.5, *v*/*v*/*v*) and precipitated in ice‐cold diethyl ether. Then it was purified by preparative HPLC (column: Nucleodur C18ec; 100–5; Macherey‐Nagel; solvent: 10–60 % MeCN in H_2_O (incl. 0.1 % TFA) over 45 min, 6.0 mL/min flow rate). The product was identified via HPLC‐ESI MS (column: Nucleodur C18ec; 100–5; Macherey‐Nagel; gradient: 10–60 % MeCN in H_2_O (incl. 0.1 % formic acid) over 15 min; 0.6 mL/min flow rate). After purification a yield of 19.7 mg (8.08 μmol, 53.8 %) was determined.

**Synthesis of [Pt(dpyTSCL‐sC18)Cl]**: 500 μL 5 mM stock solution of HdpyTSCL‐sC18 in H_2_O were added to 250 μL of a freshly prepared 10 mM aqueous solution of K_2_PtCl_4_ (41.5 mg in 10 mL) and incubated for 2 h under shaking at RT. The complex formation reaction was UV/Vis monitored through the absorption at 500 nm. The crude complex was desalted using a C18ec cartridge (Chromafix C18ec, Macherey‐Nagel), solvent was evaporated and the red residue lyophilised. The product was analysed by ESI‐MS(+) (Figures 7 and S2) and UV/Vis absorption spectroscopy (Figure S21).

**Synthesis of [Pt(dpyTSCL‐sC18)(CN)]**: 75 μL of a 10 mM aqueous KCN solution (6.52 mg in 10 mL) were added to a 500 μL 1 mM stock solution of HdpyTSCL‐sC18 and incubated under shaking for 1 h at RT. Then, 50 μL aqueous K_2_PtCl_4_ solution (4.15 mg in 1 mL) was added and the reaction mixture incubated for 1 h at RT. The complex formation was UV/Vis monitored through the absorption at 500 nm. The crude complex was desalted using a C18ec cartridge (Chromafix C18ec, Macherey‐Nagel), solvent was evaporated and the red residue lyophilised. Figure 8 shows HPLC analysis and ESI‐MS(+) spectrum of [Pt(dpyTSCL‐sC18)(CN)]. Final HPLC chromatogram was recorded using a linear gradient from 10–60 % MeCN in H_2_O (incl. 0.1 % trifluoroacetic acid) within 15 min (Nucleodur C18ec; 100–5 (Macherey‐Nagel) column (1 mL/min flow rate)). UV/Vis absorption spectroscopy in Figure S21.

**Instrumentation**: NMR spectra were recorded on a Bruker Avance II 300 MHz spectrometer, using a triple resonance ^1^H, ^n^BB inverse probe head. The unambiguous assignment of the ^1^H and ^13^C resonances was obtained from 1H NOESY, ^1^H COSY, gradient selected ^1^H, ^13^C HSQC and ^1^H, ^195^Pt HMBC experiments. All 2D NMR experiments were performed using standard pulse sequences from the Bruker pulse program library. Chemical shifts were relative to TMS (^1^H, ^13^C) or H_2_PtCl_6_ (^195^Pt). UV/Vis absorption spectra were measured using a Varian 50 Scan UV–visible photometer. EPR spectra were recorded in the X‐band on a Bruker System ELEXSYS 500E equipped with a Bruker Variable Temperature Unit ER 4131VT (500 to 100 K); the *g* values were calibrated using a dpph sample. Electrochemical experiments were carried out in 0.1 m *n*Bu_4_NPF_6_ solutions using a three‐electrode configuration (glassy carbon working electrode, Pt counter electrode, Ag/AgCl pseudo reference) and an Autolab PGSTAT30 potentiostat and function generator. Experiments were run at a scan rate of 100 mV/s, at ambient temperature and the ferrocene/ferrocenium couple served as internal reference. UV/Vis spectroelectrochemical measurements were performed with an optical transparent thin‐layer electrochemical (OTTLE) cell.[^82^, ^83^] EI‐MS(+) spectra were recorded on a Thermo Quest Finnigan MAT 95 at 70 eV. HR‐ESI‐MS(+) spectra were measured at the Thermo Scientific LTQ Orbitrap XL mass spectrometer via electron spray ionisation and a FTMS Analyzer. IR spectra were obtained on a Si crystal Fourier‐Transform spectrometer by Thermo Scientific (Nicolet 380 FTIR).

**Crystal structure determination**: The measurements were performed at 293(2) K on IPDS II (STOE and Cie.) or at 100(2) K on a Bruker Apex II, both using graphite‐monochromatised Mo_Kα_ radiation (*λ*=0.71073 Å). These structures were solved by direct methods using SHELX‐97 and WinGX (SHELXS‐97)[^84^, ^85^, ^86^] and refined by full‐matrix least‐squares techniques against *F* ^*2*^ (SHELXL‐2017/1).[^87^, ^88^] The numerical absorption corrections (X‐RED V1.22; Stoe & Cie, 2001) were performed after optimising the crystal shapes using X‐SHAPE V1.06 (Stoe & Cie, 1999).[^89^, ^90^] The non‐hydrogen atoms were refined with anisotropic displacement parameters. H atoms were included by using appropriate riding models. Further measurements were performed on SC‐XRD Bruker D8 Venture at 100(2) K in kappa geometry equipped with a copper micro focus source and a Photon100 detector, or at the beamline P24 of the synchrotron PETRA III at DESY in Hamburg, Germany. These structures were resolved using OLEX2 and refined by charge flipping.^[91]^ Deposition numbers 1954934 (for HfpyTSCmB), 1954950 (for HapyTSCmB), 1954933 (for HdpyTSCmB^.^MeOH), 1954935 (for [Pt(apyTSCmB)Cl]), and 2022743 (for [Pt(dpyTSCmB)(CN)]) contain the supplementary crystallographic data for this paper. These data are provided free of charge by the joint Cambridge Crystallographic Data Centre and Fachinformationszentrum Karlsruhe Access Structures service www.ccdc.cam.ac.uk/structures.

**Quantumchemical DFT calculations**: DFT‐calculations on the geometries, were carried out at the def‐SV(P)^[92]^/B3LYP[^93^, ^94^, ^95^] level by using the Turbomole ^[96]^ program package and TmoleX user interface.^[97]^ HOMO and LUMO energies were calculated at the def2‐TZVP^[98]^/B3LYP level for H, C, N, S, and Cl atoms and LANL2DZ^[99]^/B3LYP with ecp60 Hay&Watt[^52^, ^53^] for Pt.

**Peptide conjugates**: The cell‐penetrating peptide was synthesised by automated solid‐phase peptide synthesis on a Syro I peptide synthesiser (MultiSynTech). HPLC chromatograms were recorded on an Agilent 1260 HPLC system (column: Nucleodur C18ec; 100–5; Macherey‐Nagel; gradient: 10–60 % MeCN in water (incl. 0.1 % formic acid) over 15 min; 0.6 mL/min flow rate). ESI‐MS(+) spectra were recorded on a LTQ‐XL mass spectrometer by electron spray ionisation (Thermo Scientific). Data was evaluated with XcaliburTM Software (Thermo Scientific).^[100]^

**Antiproliferative activities**: Antiproliferative activity was determined by photospectrometric measurements. The conversion of resazurin (*λ*_ex_=550 nm) into the resorufin product (*λ*_ex_=595 nm) was measured^[101]^ on a Tecan infinite M200 plate reader (Tecan Group AG). Data were normalised to untreated control cells were (100 % viability). Statistical analysis was performed via Microsoft Excel T‐test with two tails, type 2 (two‐sample, equal variances).

## Conflict of interest

The authors declare no conflict of interest.

## Supporting information

As a service to our authors and readers, this journal provides supporting information supplied by the authors. Such materials are peer reviewed and may be re‐organized for online delivery, but are not copy‐edited or typeset. Technical support issues arising from supporting information (other than missing files) should be addressed to the authors.

Supplementary
