# Supplementary material for: Building up PtII−Thiosemicarbazone−Lysine−sC18 Conjugates
Source: Chembiochem. 2020 Nov 6;22(4):694–704. doi: 10.1002/cbic.202000564 (PMC7894172; doi:10.1002/cbic.202000564)
Supplement: Supplementary file 1 — Supplementary [file CBIC-22-694-s001.pdf]

ChemBioChem

Supporting Information

Building up Pt^{II}–Thiosemicarbazone–Lysine–sC18 Conjugates

Alexander Haseloer, Tamara Lützenburg, Joss Pepe Strache, Jörg Neudörfl, Ines Neundorf,* and Axel Klein*

Contents:

1. Experimental Part - Syntheses **page 1 - 8**

2. Supplementary Figures and Tables **page 8 - 27**

Figure S1. HPLC chromatogram and ESI MS analysis of the HdpyTSCL-sC18 conjugate.
Figure S2. HPLC-ESI-MS(+) analysis of [Pt(dpyTSCL-sC18)Cl] conjugate after desalting (first batch).
Table S1. Calculated and identified m/z values for [Pt(dpyTSCL-sC18)Cl] and [Pt(dpyTSCL-sC18)].
Figure S3. Molecular structure of HfpyTSCmB.
Figure S4. Crystal structure of HfpyTSCmB.
Table S2. Crystal data and structure refinement for HfpyTSCmB.
Table S3. Atomic coordinates and equivalent isotropic displacement parameters for HfpyTSCmB.
Table S4. Bond lengths [Å] and angles [°] for HfpyTSCmB.
Table S5. Anisotropic displacement parameters for HfpyTSCmB.
Figure S5. Molecular structure of HapyTSCmB.
Figure S6. Crystal structure of HapyTSCmB.
Table S6. Crystal data and structure refinement for HdpyTSCmB.
Table S7. Atomic coordinates and equivalent isotropic displacement parameters for HapyTSCmB.
Table S8. Bond lengths [Å] and angles [deg] for HapyTSCmB.
Table S9. Anisotropic displacement parameters for HapyTSCmB.
Figure S7. Molecular structure of HdpyTSCmB·MeOH.
Figure S8. Crystal structure of HdpyTSCmB·MeOH.
Table S10. Crystal data and structure refinement for HdpyTSCmB.
Table S11. Atomic coordinates and equiv. isotropic displacement parameters for HdpyTSCmB.MeOH.
Table S12. Bond lengths [Å] and angles [°] for HdpyTSCmB.MeOH.
Table S13. Anisotropic displacement parameters for HdpyTSCmB·MeOH.
Figure S9. Molecular structure of [Pt(apyTSCmB)Cl].
Figure S10. Crystal structure of [Pt(apyTSCmB)Cl].
Table S14. Crystal data and structure refinement for [Pt(apyTSCmB)Cl].
Table S15. Atomic coordinates and equiv. isotropic displacement parameters for [Pt(apyTSCmB)Cl].
Table S16. Bond lengths [Å] and angles [deg] for [Pt(apyTSCmB)Cl].
Table S17. Anisotropic displacement parameters for [Pt(apyTSCmB)Cl].
Figure S11. Molecular structure of [Pt(dpyTSCmB)(CN)].
Figure S12. Crystal structure of [Pt(dpyTSCmB)(CN)] along the crystallographic axis.
Table S18. Crystal data and structure refinement for [Pt(dpyTSCmB)(CN)].
Table S19. Atomic coordinates and equiv. isotropic displacement parameters for [Pt(dpyTSCmB)(CN)].
Table S20. Bond lengths [Å] and angles [°] for [Pt(dpyTSCmB)(CN)].
Table S21. Anisotropic displacement parameters for [Pt(dpyTSCmB)(CN)].
Table S22. Selected NMR data of Pt TSC complexes.
Figure S13. UV-vis absorption spectra of HfpyTSCmB, [Pt(fpyTSCmB)Cl], HapyTSCmB, and [Pt(apyTSCmB)Cl].
Figure S14. UV-vis absorption spectra of TSC ligands and Pt complexes in THF.
Figure S15. UV-vis absorption spectra of (A) HfpyTSCLp and [Pt(fpyTSCLp)Cl], (B) HapyTSCLp and [Pt(apyTSCLp)Cl] and (C) HdpyTSCLp and [Pt(dpyTSCLp)Cl] in THF.
Table S23. UV-vis absorption data of selected TSC Pt complexes.
Figure S16. Cyclic and square wave voltammograms of [Pt(fpyTSCmB)Cl] and [Pt(apyTSCmB)Cl].
Figure S17. Cyclic and square wave voltammograms of [Pt(fpyTSCLp)Cl] (left) and [Pt(apyTSCLp)Cl].
Figure S18. Cyclic and square wave voltammograms of [Pt(dpyTSCLp)Cl] and [Pt(dpyTSCmB)(CN)].
Table S24. Electrochemical data for the TSC Pt complexes.
Figure S19. DFT-calculated composition of LUMO+2, LUMO+1, HOMO-1, HOMO-2 for [Pt(dpyTSCmB)Cl].
Figure S20. UV-vis absorption spectra recorded during the reduction of [Pt(dpyTSCmB)Cl].
Figure S21. UV-vis absorption spectra of the peptide conjugates HdpyTSCL-sC18, [Pt(dpyTSCL-sC18)Cl], HdpyTSCL-sC18, [Pt(dpyTSCL-sC18)(CN)] in demineralised H_2O.
Figure S22. Correlation of the long-wavelength MLCT absorption maxima of [Pt(dpyTSCLp)Cl] and [Pt(dpyTSCL-sC18)Cl] with the Reichhardt ET(30) values for the solvent polarity.
Figure S23. UV-vis absorption spectra of FBS solution (red) and [Pt(dpyTSCL-sC18)Cl] (left) and [Pt(dpyTSCL-sC18)(CN)] (right) in FBS (green).

Figure S24. Anti-proliferative activity of Dp44mT (Di-2-pyridylketone-4,4,-dimethyl-3-thiosemicarbazone) against MCF-7, HT-29 and HEK-293 cells.

3. Cell viability assay **page 28**
4. NMR spectra **page 29-71**

---

1. Experimental Part - Syntheses

1.1 Syntheses of ligand precursors and HTSCmB protoligands

Synthesis of isocyanate ITCmB

Under an argon atmosphere, 10.1 mL (78.4 mmol, 1 eq.) of (*S*)-(–)-α-methylbenzylamine were dissolved in 60 mL THF and 35.9 mL (259 mmol, 3.3 eq.) of triethylamine were added. The solution was cooled down to 0 °C and 4.7 mL of CS_2 (78 mmol, 1 eq.) were slowly added. The reaction mixture was stirred for 1 h and then 16.5 g (86.2 mmol, 1.1 eq.) of 4-toluenesulfonyl chloride were added. After another 1.5 h of stirring, the liquid as poured into 60 mL of 1M HCl. The aqueous phase was extracted with 3 × 50 mL MTBE and the combined organic phases were dried over $MgSO_4$. The crude product was filtered through a silica plug with *n*-hexane, yielding the product as orange oil.

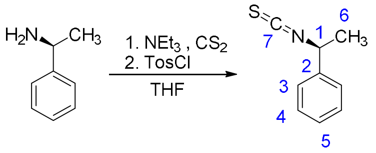

Yield: 13.4 g (82.0 mmol, > 99%, Lit.: 99% [1]). C_9H_9NS (163.24 g/mol). ^{1}H NMR: (300 MHz, DMSO-d$_6$) δ [ppm] = 7.32–7.45 (m, 5H, H3,4,5), 5.22 (q, 1H, *J* = 6.7 Hz, H6), 1.60 (d, 3H, *J* = 6.8 Hz, H6). ^{13}C NMR: (DMSO-d$_6$, 75 MHz) δ [ppm] = 140.2 (s, C2), 128.8 (d), 128.1 (d, C5), 125.6 (d), 56.4 (d, C1), 24.2 (q, C6).

Synthesis of the TSCmB thiosemicarbazide

4.0 mL (82 mmol, 1.0 eq.) of hydrazine monohydrate were dissolved in 75 mL of MeOH and the solution was heated to 70 °C. Then, 13.4 g (82.0 mmol, 1.0 eq.) of the isothiocyanate were slowly added and the reaction mixture was stirred for 1.5 h. The solvent was removed under reduced pressure and the crude product was poured into boiling petrol ether. The formed oil phase separated from the ether phase. The thiosemicarbazide was obtained as orange oil.

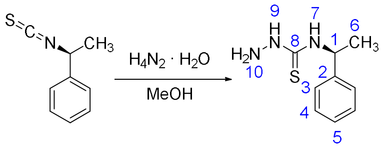

Yield: 15.8 g (80.9 mmol, 99%, Lit.: 77% [2]). $C_9H_{13}N_3S$ (195.29 g/mol). ^{1}H NMR: (300 MHz, DMSO-d$_6$) δ [ppm] = 8.72 (br s, 1H, *N*H9), 8.02 (d, 1H, *J* = 8.9 Hz, *N*H7), 7.14–7.41 (m, 5H, H3-H5), 4.54 (br s, 2H, *N*H10), 5.57 (quint, *J* = 6.9 Hz, 1H, H1), 1.45 (d, 3H, *J* = 7.0 Hz, H6). ^{13}C NMR: (DMSO-d$_6$, 75 MHz) δ [ppm] = 180.6 (s, C8), 144.3 (s, C2), 128.2 (t), 126.7 (t, C5), 126.2 (t), 51.8 (d, C1), 22.1 (q, C6).

Synthesis of the thiosemicarbazone protoligands HTSCmB – general description

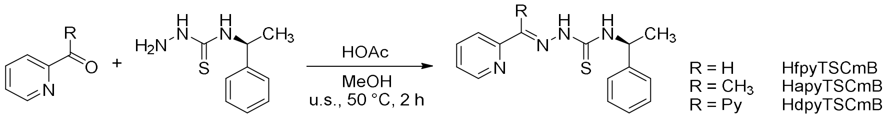

1 eq. of aldehyde or ketone and 1 eq. of thiosemicarbazide were dissolved in 5 mL of MeOH and 0.1 mL of acetic acid were added. The solution was sonicated at 50 °C for 30 min. The precipitate was filtrated and washed with cold MeOH to yield the desired TSC. The HTSCmB molecules were soluble in solvents like THF, MeCN and MeOH but not in H_2O and stable in these solutions for more than a month as studied through UV-vis absorption and ^{1}H NMR spectroscopy.

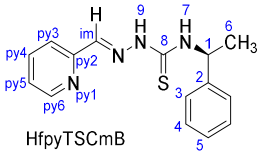

HfpyTSCmB. Yield: 1.37 g (4.82 mmol, > 99%), slight yellow solid. $C_{15}H_{16}N_4S$ (284.38 g/mol). ^{1}H NMR: (300 MHz, DMSO-d$_6$) δ [ppm] = 11.77 (s, 1H, *N*H9), 8.76 (d, 1H, *J* = 8.6 Hz, *N*H7), 8.57 (d, 1H, *J* = 4.2 Hz, Hpy6), 8.31 (d, 1H, *J* = 7.9 Hz, Hpy5), 8.76 (s, 1H, Him), 7.85 (t, 1H, *J* = 7.3 Hz, Hpy4), 7.32-7.44 (m, 5H, H3, H4, H5), 7.24 (t, 1H, *J* = 7.1 Hz, Hpy3), 5.75 (quint, 1H,

J = 6.8 Hz, H1), 1.58 (s, 3H, H6).

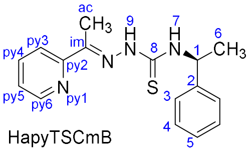

HapyTSCmB. Yield: 0.91 g (3.1 mmol, 74%), slight yellow solid. $C_{16}H_{18}N_4S$ (298.41 g/mol). ^{1}H NMR: (300 MHz, DMSO-d$_6$) δ [ppm] = 10.41 (s, 1H, *N*H9), 8.70 (d, 1H, J = 8.8 Hz, *N*H7), 8.59 (d, 1H, J = 4.7 Hz, Hpy6), 8.34 (d, 1H, J = 8.1 Hz, Hpy3), 7.84 (t, 1H, J =8.6 Hz, Hpy4), 7.32-7.44 (m, 5H, H3, H4, H5), 7.24 (t, 1H, J = 7.2 Hz, Hpy5), 5.76 (quint, 1H, J = 7.0 Hz, H1), 2.41 (s, 3H, Hac), 1.58 (3H, J = 7.0 Hz, H6). ^{13}C NMR: (DMSO-d$_6$, 75 MHz) δ [ppm] = 177.6 (C8), 154.6 (Cim), 148.8 (Cpy2), 148.5 (Cpy6), 143.6 (C2), 136.5 (Cpy5), 128.2 (C3), 126.7 (Cpy4), 126.3 (C4), 124.0 (C5), 120.9 (Cpy3), 52.7 (C1), 21.3 (C6), 12.4 (Cac).

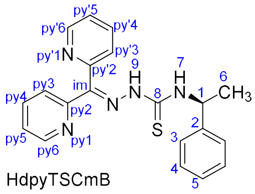

HdpyTSCmB. Yield: 1.30 g (3.60 mmol, 66%) yellow solid. $C_{20}H_{19}N_5S$ (361.47 g/mol). ^{1}H NMR: (300 MHz, DMSO-d$_6$) δ [ppm] = 13.31 (s, 1H, *N*H9), 8.97 (d, 1H, J = 8.7 Hz, *N*H7), 8.82 (d, 1H, J = 4.2 Hz, Hpy6), 8.58 (d, 1H, J = 4.6 Hz, Hpy'6), 8.21 (d, 1H, J = 7.9 Hz, Hpy'4), 7.94-8.01 (m, 2H, Hpy3/Hpy'3), 7.55-7.59 (m, 1H, Hpy4), 7.41-7.49 (m, 4H, H3, Hpy5, Hpy'5),7.34 (t, 2H, J = 7.4 Hz, H4), 7.24 (t, 1H, J = 7.2 Hz, H5), 5.71 (quint, J = 7.1 Hz, 1H, H1), 1.59 (d, 3H, J = 7.0 Hz, H6). ^{13}C NMR: (75 MHz, DMSO-d$_6$) δ [ppm] = 177.45 (C8), 155.46 (Cpy'2), 151.56 (Cpy2), 148.84 (Cpy3), 148.59 (Cpy'3), 143.76 (C2), 138.01 (Cpy'6), 137.59 (Cpy6), 128.70 (C4), 127.56 (C5), 127.29 (Cpy'5), 127.08 (Cpy5), 126.76 (C3), 125.19 (Cim), 124.37 (Cpy4), 124.31 (Cpy'4), 53.57 (C1), 21.54 (C6).

1.2 Syntheses of *L*-lysine derivatives

Synthesis of *N*^{6}-(benzyloxy)carbonyl)-*L*-lysine

18.25 g (99.92 mmol, 1 eq.) of *L*-lysine hydrochloride were dissolved in 80 mL H_2O to obtain a pale-yellow solution and 8 g (200 mmol, 2 eq.) NaOH were added. Separately 12.48 g (50 mmol, 0.5 eq.) of $CuSO_4{\cdot}5H_2O$ were dissolved in 40 mL H_2O and added to the lysine solution. The mixture was cooled in an ice bath and 9.98 g (119 mmol, 1.19 eq.) $NaHCO_3$ were added. Then 19 mL (22.8 g, 134 mmol, 1.34 eq.) CbzCl (benzylchloroformate) was added dropwise over 10 min. The reaction was stirred overnight, and the pale-blue voluminous precipitate was collected. The crude product was washed with 200 mL of H_2O and 100 mL of acetone.

In a 1000 mL three-necked flask equipped with a reflux condenser 41.8 g (110 mmol, 1.1 eq.) EDTA sodium salt was dissolved in 400 mL H_2O. The *pH* was adjusted to 7 using concentrated hydrochloric acid. The mixture was heated to reflux, and the lysine copper complex was added portion wise. During the addition the *pH* was maintained at 7 using HCl. After the addition was completed the mixture was heated to reflux for another 30 min before the solution was cooled to r.t. and the precipitate was filtered and washed with 3 × 100 mL H_2O and with 2 × 100 mL MeOH. The crude product was recrystallised from EtOH. 29.56 g of a slight blue solid was obtained.

***N*^{6}-Cbz-lysine.** Yield: 29.56 g (99.9 mmol, 99%). $C_{20}H_{19}N_5S$ (280.32 g/mol).

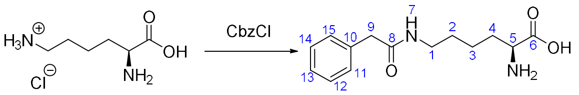

^{1}H NMR: (300 MHz, DMSO-d$_6$) δ [ppm] = 7.43-7.27 (m, 4H, H11, H12, H14, H15), 7.23 (t, 1H, J = 5.5 Hz, H13), 5.00 (s, 2H, H9), 3.16-3.10 (m, 1H, H5), 2.97 (q, J = 6.5 Hz, 2H, H1), 1.77-1.45 (m, 2H, H4), 1.41-1.19 (m, 4H, H2, H3).

Synthesis of *N*^{6}-(benzyloxy)carbonyl)-*N*^{2}-(*t*-butoxycarbonyl)-*L*-lysine

In a 500 mL round-bottomed flask 15 g (53.21 mmol, 1 eq.) *N*^{6}-Cbz lysine was treated with a solution of 5.67 g (53.5 mmol, 1 eq.) Na_2CO_3 in 100 mL H_2O 75 mL *t*-BuOH. To the slurry 11.67 g (53.5 mmol, 1 eq.) Boc_2O dissolved in 25 mL *t*-BuOH was added over 10 min. The slurry was heated under reflux for 4 h upon which cleared the solution. The mixture was allowed to cool to r. t. and stirred overnight. The pale-blue suspension was diluted with 450 mL H_2O and extracted using 200 mL *n*-pentane. The organic phase was discarded. The aqueous phase was cooled to 0 °C and carefully acidified with 120 mL of 1.2 M HCl. The aqueous phase was extracted with 3 × 250 mL EtOAc and the combined organic

phase was washed with 10% Na_2SO_4 solution and dried over Na_2SO_4. The solvent was removed under reduced pressure yielding 13.85 g of colourless oil.

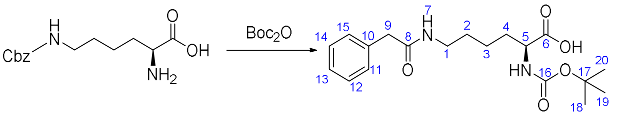

***N*⁶-Cbz *N*¹-Boc lysine.** Yield: 13.85 g (36.4 mmol, 68%). $C_{19}H_{28}N_2O_6$ (380.44 g/mol). ^{1}H NMR: (300 MHz, $CDCl_3$) δ [ppm] = 8.62 (s, 1H, *N*H7), 7.32 (s, 5H, H11, H12, H14, H15), 5.32 (t, 1H, *J* = 8.7 Hz, N*H*), 5.07 (s, 2H, H9), 4.27 (d, 1H, *J* = 6.5 Hz, H5), 3.16 (d, 2H, *J* = 6.5 Hz, H1), 1.87-1.56 (m, 2H, H4), 1.59-1.38 (m, 4H, H2, H3), 1.42 (s, 9H, H18, H19, H20). ^{13}C NMR: ($CDCl_3$, 75 MHz) δ [ppm] = 176.2 (C6), 156.8 (C8), 155.9 (C16), 136.6 (C10), 128.6 (C12, C13, C14), 128.2 (C15, C11), 80.1 (C17), 66.8 (C9), 53.2 (C5), 40.7 (C1), 32.1 (C4), 29.4 (C2), 28.4 (C18, C19, C20), 22.4 (C3).

Synthesis of *t*-butyl *N*⁶-((benzyloxy)carbonyl)-*N*²-(*t*-butoxycarbonyl)-L-lysinate

In a 1000 mL round-bottomed flask 15.2 g (39.95 mmol, 1 eq.) of *N*²-Boc *N*⁶-Cbz lysine was dissolved in 80 mL *t*-BuOH and 488 mg (3.99 mmol, 0.1 eq.) DMAP were added. To the colourless solution 11.34 g (51.94 mmol, 1.3 eq.) Boc_2O were added dropwise over 10 min. The mixture was stirred at r.t. for 24 h before it was stored at 6 °C overnight. The solvent was removed under reduced pressure yielding a yellow oil. The residue was dissolved in 300 mL diethyl ether and washed with 160 mL 0.5 M HCl, 160 mL 5% $NaHCO_3$ and 100 mL 10% Na_2SO_4 solution. The organic phase was dried over Na_2SO_4 and the solvent was removed under reduced pressure yielding yellow oil. The crude product was purified by flash column chromatography using a *c*-hexane/EtOAc (v/v ≈ 1:1) mixture. The solvent was removed under reduced pressure yielding 14.05 g of pale-yellow oil.

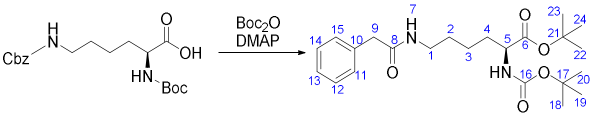

***N*⁶-Cbz-*N*¹-Boc *C*-*t*-butyl-lysine.** Yield: 14.05 g (32.1 mmol, 80%). $C_{19}H_{28}N_2O_6$ (380.44 g/mol). R_f: (*c*-hexane/EtOAc; v/v ≈ 6:4): 0.61. FTIR-ATR: ν [cm^{-1}] = 3855 (w), 3675 (m), 2987 (s), 2901 (s), 2359 (w), 2173 (w), 2116 (w), 1716 (m), 1653 (w), 1507 (w), 1454 (w), 1406 (m), 1394 (m), 1381 (m), 1152 (m), 1075 (s), 1066 (s), 1056 (s), 1028 (m), 892 (w), 880 (w), 776 (w), 737 (w), 697 (w), 618 (w), 611 (w), 599 (w). ^{1}H NMR: (300 MHz, $CDCl_3$) δ [ppm] = 7.38-7.27 (m, 2H, H11, H12, H13, H14, H15), 5.07 (s, 3H, H5, H9), 4.93 (s, 1 H, N*H*), 3.16 (q, *J* = 6.5 Hz, 2H, H1), 1.85-1.47 (m, 6H, H2, H3, H4), 1.44 (s, 9H, H22, H23, H24), 1.42 (s, 9H, H18, H19, H20). ^{13}C NMR: (75 MHz, $CDCl_3$) δ [ppm] = 156.5 (C8), 155.5 (C16), 136.7 (C7), 128.6 (C12, C14), 128.2 (C13), 128.1 (C11, C15), 81.9 (C17), 79.7 (C21), 66.6 (C9), 53.8 (C5), 40.8 (C1), 32.7 (C4), 29.5 (C2), 28.4 (C18, C19, C20), 28.1 (C22, C23, C24), 22.4 (C3).

Synthesis of *t*-butyl (*t*-butoxycarbonyl)-L-lysinate

In a dry 250 mL round-bottom Schlenk flask 13.97 g (32 mmol, 1 eq.) of the protected lysine were dissolved in 100 mL MeOH and 1.221 g Pd on charcoal (5% Pd-basis) was suspended in the solution. The argon atmosphere was removed under reduced pressure and replaced by a H_2 atmosphere. The mixture was stirred at r.t. for 19 h. The suspension was filtered over Celite and washed with 3 × 50 mL MeOH. The solvent was removed under reduced pressure yielding 9.26 g of pale-yellow oil.

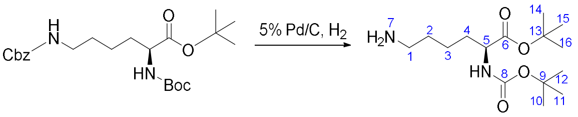

***N*¹-Boc-*C*-*t*-butyl-lysine.** Yield: 9.26 g (30.6 mmol, 96%). $C_{15}H_{30}N_2O_4$ (302.42 g/mol). R_f: (*c*-hexane/EtOAc; v/v ≈ 6:4): 0.69 (UV-inactive). FTIR-ATR: ν [cm^{-1}] = 3675 (m), 2987 (s), 2901 (s), 1713 (m), 1454 (w), 1394 (m), 1369(m), 1249 (m), 1155 (m), 1099 (w), 1065 (s), 1054 (s), 1021 (m), 910 (w), 879 (w), 818 (w), 799 (w), 756 (w), 737 (w), 722 (w), 690 (w), 661 (m), 652 (m), 639 (m), 630 (m), 615 (m), 606 (m), 611 (m), 590 (w), 579 (m), 566 (w), 555 (w), 544 (w), 527 (w). ^{1}H NMR: ($CDCl_3$, 300 MHz) δ [ppm] = 5.10 (d, 2H, *J* = 8.4 Hz, *N*H7), 4.09 (q, 2H, *J* = 7.0 Hz, H5), 2.62 (t, 2H, *J* = 6.7 Hz, H1), 1.79-1.45 (m, 5H, H2, H3, H4), 1.39 (s, 9H, H14, H15, H16), 1.37 (s, 9H, H10, H11, H12). ^{13}C NMR: (75 MHz, $CDCl_3$, 298

K) δ [ppm] = 172.0 (C6), 155.4 (C8), 81.7 (C9), 79.5 (C13), 53.9 (C5), 41.9 (C1), 33.3 (C4), 32.7 (C3), 28.4 (C10, C11, C12), 28.0 (C14, C15, C16), 22.5 (C2).

Synthesis of *t*-butyl-*N*²-(*t*-butoxycarbonyl)-*N*⁶-thioxomethylidene-*L*-lysinate (ITCLp)

5 g (16.5 mmol, 1 eq.) of the partially unprotected lysine is dissolved under inert conditions in 25 mL dry THF and 7 mL Et_3N. The mixture was cooled to 0 °C and 1 mL (1.26 g, 16.5 mmol, 1 eq.) CS_2 was added dropwise over 30 min. The reaction mixture was allowed to warm to r.t. and stirred overnight. The mixture was cooled to 0 °C and 3.49 g (18.2 mmol, 1.1 eq.) TosCl was added. To the resulting suspension MTBE (methyl-*t*-butyl ether) was added, the organic phase was separated, and the aqueous phase was extracted with 2 × 50 mL MTBE. The collected organic phases were dried over Na_2SO_4 and the solvent was removed under reduced pressure yielding yellow oil. The crude product was purified by flash chromatography (*c*-hexane/EtOAc; v/v ≈ 18:1). The corresponding fractions were collected and the solvent was removed under reduced pressure yielding pale-yellow oil.

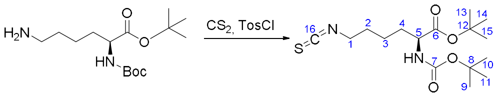

Yield: 4.482 g (13 mmol, 79%). $C_{15}H_{30}N_2O_4$ (302.42 g/mol). R_f: (*c*-hexane/EtOAc; v/v≈18:1): 0.23. FTIR-ATR: ν [cm⁻¹] = 3675 (m), 2987 (s), 2901 (s), 2102 (br), 1711 (m), 1506 (m), 1453 (m), 1405 (m), 1394 (s), 1381 (m), 1366 (m), 1250 (m), 1230 (m), 1152 (m), 1069 (s), 1028 (s), 893 (w), 800 (w), 780 (w), 712 (w), 696 (w), 682 (w), 669 (w), 649 (w), 634 (w), 627 (w), 608 (s), 590 (w), 579 (w), 539 (w), 518 (w). ^{1}H NMR: ($CDCl_3$, 300 MHz) δ [ppm] = 4.16 (q, 2H, *J* = 7.0 Hz, H5), 3.50 (t, 2H, *J* = 6.5 Hz, H1), 1.86-1.53 (m, 6H, H2, H3, H4), 1.45 (s, 9H, H13, H14, H15), 1.43 (s, 9H, H9, H10, H11). ^{13}C NMR: (75 MHz, $CDCl_3$) δ [ppm] = 171.7 (C6), 155.4 (C7), 100.1 (C16), 82.2 (C8), 79.8 (C12), 53.6 (C5), 44.9 (C1), 32.4 (C4), 29.6 (C3) 28.4 (C9/C10/C11), 28.0 (C13/C14/C15), 22.4 (C2).

Synthesis of *t*-butyl-*N*²-(*t*-butoxycarbonyl)-*N*⁶-(hydrazinecarbonothioyl)-*L*-lysinate (thiosemicarbazide-Lp)

0.63 (12.6 mmol, 1 eq.) hydrazine hydrate was diluted with 20 mL MeOH and heated to reflux. At this temperature 4.338 g (12.6 mmol, 1 eq.) of the isothiocyanate dissolved in 9 mL MeOH were added dropwise over 20 min. The reaction mixture was stirred at 75 °C for 2 h. The solvent was removed under reduced pressure yielding 4.713 g of sticky yellow oil.

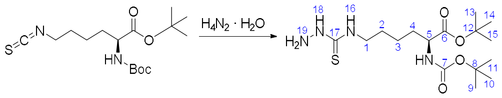

Yield: 4.713 g (12.6 mmol, 99%). $C_{16}H_{32}N_4O_4S$ (376.52 g/mol). R_f: (*c*-hexane/EtOAc; v/v≈6:4): 0.69 (UV-inactive). FTIR-ATR: ν [cm⁻¹] = 3903 (w), 3853 (w), 3839 (w), 3750 (w), 3734 (w), 3689 (m), 3675 (m), 3566 (w), 3335 (br), 2972 (s), 2900 (s), 2361 (w), 2174 (w), 2116 (w), 1706 (m), 1653 (w), 1635 (w), 1540 (m), 1507 (m), 1456 (m), 1394 (m), 1367 (m), 1250 (m), 1152 (s), 1068 (s), 1028 (m), 950 (w), 892 (w), 864 (w), 848 (w), 774 (w), 737 (w), 629 (w), 621 (w), 614 (w), 602 (w), 582 (w), 570 (w), 558 (w), 539 (w). ^{1}H NMR: ($CDCl_3$, 300 MHz) δ [ppm] = 7.76 (s, 1H, *N*H18), 7.43 (s, 1H, *N*H16), 5.06 (d, *J* = 8.3 Hz, 1H, *N*H), 4.13 (q, 1H, *J* = 7.3 Hz, H5), 3.78 (brs, 2H, *N*H19), 3.60 (q, 2H, *J* = 7.1 Hz, H1), 1.84-1.52 (m, 6H, H2, H3, H4), 1.44 (s, 9H, H13, H14, H15), 1.42 (s, 9H, H9, H10, H11). ^{13}C NMR: ($CDCl_3$, 75 MHz) δ [ppm] = 182.2 (C17), 172.0 (C6), 155.5 (C7), 82.0 (C8), 79.7 (C12), 53.9 (C5), 43.8 (C1), 32.8 (C4), 29.1 (C3), 28.4 (C9/C10/C11), 28.1 (C13/C14/C15), 22.7 (C2).

1.3 Synthesis of thiosemicarbazone protoligands HTSCLp – general procedure

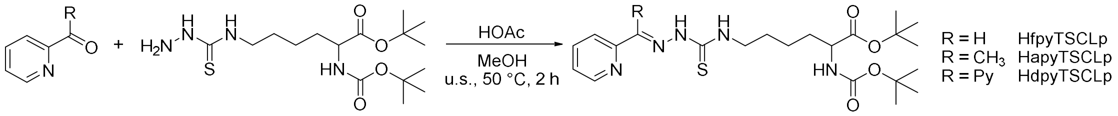

In a 5 mL pear shaped flask 989 mg (2.63 mmol) of the thiosemicarbazide-Lp derivative was dissolved in 3.5 mL MeOH and 0.1 mL HOAc. To the colourless solution 2.63 mmol of the aldehyde or ketone was added and the reaction mixture was sonicated at 50 °C for 30 min. The solvent was removed under reduced pressure and the crude product was purified by flash chromatography (first *c*-hexane/EtOAc; v/v ≈ 4:6; then pure EtOAc). The solvent was removed under reduced pressure. The

HTSCLp molecules were soluble and stable for more than 2 months (UV-vis and NMR spectroscopy) in solvents ranging from non-polar *p*-xylene to polar MeOH but not in H_2O.

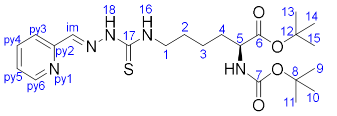

HfpyTSCLp. Yield: 0.25 g (0.54 mmol, 58%). $C_{22}H_{35}N_5O_4S$ (465.61 g/mol). ^{1}H NMR: (300 MHz, DMSO-*d*$_6$) δ [ppm] = 10.00 (1H, *N*H18), 9.81 (s, 1H, Him), 8.15 (t, *J* = 6.0 Hz, 1H, Hpy6), 8.06 (td, 1H, *J* = 7.6, *J* = 1.6 Hz, Hpy3), 7.94 (dt, 1H, *J* = 7.8, *J* = 1.2 Hz, Hpy4), 7.72 (ddd, 1H, *J* = 7.5, 4.7, 1.4 Hz, Hpy5), 7.09 (d, 1H, *J* = 7.7 Hz, *N*H16), 3.75 (q, 1H, *J* = 7.5 Hz, H5), 3,49 (m, 2H, H1), 1.54 (m, 6H, H2, H3, H4), 1.38 (s, 18H, H9, H10, H11, H13, H14, H15).

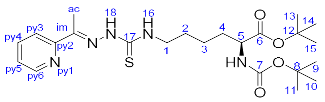

HapyTSCLp. Yield: 1.13 g (2.36 mmol, 91%). $C_{23}H_{37}N_5O_4S$ (479.64 g/mol). R*f*: (*c*-hexane/EtOAc 9:1) = 0.26.

FTIR-ATR: ν [cm^{-1}] = 3675 (m), 2987 (s), 2901 (s), 1701 (m), 1533 (m), 1455 (m), 1405 (m), 1384 (s), 1250 (m), 1152 (m), 1075 (s), 1028 (s), 880 (w), 785 (w), 747 (w), 737 (w), 704 (w), 689 (w), 670 (w), 662 (w), 626 (m), 615 (s), 600 (w), 579 (w), 545 (w). ^{1}H NMR: (300 MHz, $CDCl_3$) δ [ppm] = 10.28 (s, 1H, *N*H18), 8.66 (t, *J* =5.7 Hz, 1H, *N*H16), 8.59 (dt, 1H, *J* = 7.8 Hz, *J* = 1.3 Hz, Hpy3), 8.39 (d, 1H, *J* = 8.1 Hz, Hpy6), 7.84 (td, 1H, *J* = 7.8 Hz, *J* = 1.8 Hz, H2py4), 7.40 (ddd, 1H, *J* = 8.1 Hz, *J* = 1.2 Hz, Hpy5), 3.77 (td, 1H, *J* = 8.2 Hz, *J* = 5.5 Hz, H5), 3.59 (q, 2H, *J* = 7.0 Hz, H1), 2.38 (s, 3H, Hac), 1.60 (m, 6H, H2/H3/H4), 1.37 (s, 18H, H9/H10/H11/H13/H14/H15). ^{13}C NMR: ($CDCl_3$, 75 MHz) δ [ppm] = 177.9 (C17), 171.9 (C6), 155.5 (C7), 154.4 (Cim), 148.2 (Cpy3), 147.5 (Cpy2), 136.7 (Cpy4), 123.9 (Cpy5), 120.9 (Cpy6), 80.1 (C8), 77.9 (C12), 54.4 (C5), 43.5 (C1), 30.6 (C4), 28.4 (C3), 28.2 (C9, C10, C11), 27.6 (C13, C14, C15), 23.0 (C2), 12.1 (Cac). HR-ESI-MS(+):calculated $[C_{23}H_{37}N_5O_4S{+}Na]^+$: 502.24585; found: 502.24584.

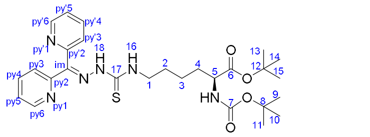

HdpyTSCLp. Yield: 5.26 g (9.7 mmol, 92%). $C_{27}H_{38}N_6O_4S$ (542.70 g/mol). R*f*: (100% EtOAc) = 0.16-0.47. FTIR-ATR: ν [cm^{-1}] = 3853 (w), 3839 (w), 3801 (w), 3734 (w), 3684 (m), 3675 (m), 3566 (w), 3335 (w), 2987 (s), 2972 (s),2091 (s), 2360 (w), 2173 (w), 2112 (w), 1923 (w), 1706 (m), 1653 (w), 1636 (w), 1588 (w), 1558 (w), 1522 (w), 1507 (w), 1455 (m), 1433 (w), 1406 (m), 1394 (s), 1230 (m), 1152 (m), 1075 (s), 1066 (s), 1057 (s), 949 (w), 892 (w), 880 (w), 868 (w), 803 (w), 774 (w), 747 (w), 679 (w), 663 (w), 650 (w), 616 (w), 608 (w), 598 (w), 591 (w), 567 (w), 546 (w), 537 (w), 518 (w). ^{1}H NMR: (300 MHz, $CDCl_3$) δ [ppm] = 8.93-8.77 (m, 1H, Hpy6), 8.68 (d, 1H, NH16), 8.59-8.47 (m, 1H, Hpy'6), 8.22 (d, 1H, Hpy'5), 8.08-7.93 (m, 3H, Hpy3/Hpy4/Hpy'3), 7.66-7.59 (m, 1H, Hpy5), 7.59-7.53 (m, 1H, Hpy'4), 7.08 (d, 1H, *J* = 7.6 Hz, *N*H16), 3.82-3.70 (m, 1H, H5), 3.66-3.36 (m, 2H, H1), 1.69-1.43 (m, 6H, H2/H3/H4), 1.38 (s, 9H, H13/H14/H15), 1.36 (s, 9H, H9/H10/H11). ^{13}C NMR: (75 MHz, $CDCl_3$) δ [ppm] = 177.2 (C17), 171.9 (C6), 155.5 (C7), 155.1 (Cpy'2), 154.3 (Cpy), 151.1 (Cim), 148.9 (Cpy6/Cpy'6), 137.1 (Cpy4/Cpy'4), 126.6 (Cpy5/Cpy'5), 124.0 (Cpy3/Cpy'3), 80.1 (C8), 77.9 (C12), 54.4 (C5), 43.7 (C1), 30.5 (C4), 28.8 (C3), 28.2 (C9/C10/C11), 27.63 (C13/C14/C15), 22.9 (C2). HR-ESI-MS(+):calculated $[C_{27}H_{38}N_6O_4S{+}Na]^+$: 565.2567455; found: 565.25674.

1.4 Synthesis of *N*6-(2-(di(pyridin-2-yl)methylene)hydrazine-1-carbonothioyl)-*L*-lysine hydrochloride

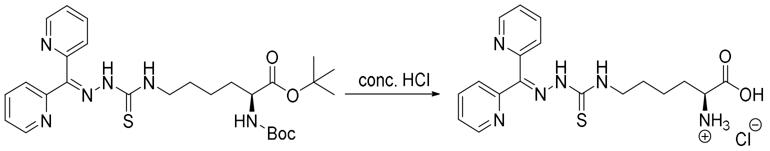

0.64 g (1.18 mmol) of HdpyTSCLp were dissolved in 150 mL concentrated HCl and stirred at ambient temperature for 48 h. The protected amino acid dissolves rapidly under gas formation. The solvent was removed under reduced pressure yielding 0.26 g of a dark yellow solid. The product was used without further purification. Yield: 0.26 g (0.61 mmol, 52%).

1.5 Synthesis of *N*2-(*t*-butoxycarbonyl)-*N*6-(2-(di(pyridin-2-yl)methylene)hydrazine-1-carbonothioyl)-*L*-lysine HdpyTSCL.

123 mg (0.3 mmol, 1 eq.) of the unprotected amino acid and 50 mg (0.45 mmol, 1.5 eq.) Na_2CO_3 were dissolved in 10 mL H_2O and 7.5 mL *t*-BuOH. 65 mg (0.3 mmol, 1 eq.) Boc_2O were added to the mixture. The reaction mixture was heated to reflux overnight. Then stirring was continued for 24 h at ambient temperature. 100 mL of H_2O were added to the reaction followed by 100 mL of *n*-pentane. The organic phase was discarded, and the aqueous phase was acidified with concentrated HCl to a *pH* of 1. Then, the aqueous phase was extracted with 3 × 100 mL EtOAc. The combined organic phases were washed with 10 wt.% Na_2SO_4 solution, dried over Na_2SO_4 and the solvent was evaporated under reduced pressure yielding 130 mg (0.27 mmol, 90%) of yellow oil. $C_{23}H_{30}N_6O_4S$ (486.59 g/mol).

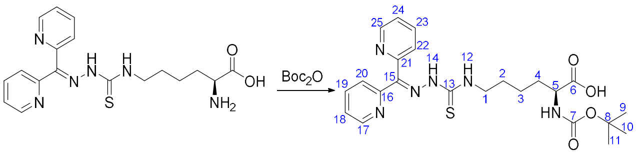

^1H NMR: (DMSO-d_6, 300 MHz) δ 13.15 (s, 1H, *N*H14), 8.89 (t, 1H, *J* = 5.9 Hz, H17), 8.83 (m, 1H, H25),8.58 (d, 1H, H22), 8.28-8.18 (m, 1H, H20), 7.98 (m, 2H, H19,23), 7.55-7.42 (m, 2H, H18,24), 7.06 (d, 1H, *J* = 8.0 Hz, *N*H12), 3.84 (dd, 1H, *J* = 8.5, 4.5 Hz, H5), 3.58 (q, 2H, *J* = 6.9 Hz, H1), 1.65 (m, 6H, H2,3,4), 1.36 (s, 9H, H9,10,11).

1.6 Synthesis of HdpyTSCL-sC18

sC18 (Gly-Leu-Arg-Lys-Arg-Leu-Arg-Lys-Phe-Arg-Asn-Lys-Ile-Lys-Glu-Lys-NH_2) was synthesised as previously described.[3]

HdpyTSCL (15 µmol, 2eq) was coupled manually to the resin using 2 eq HATU (O-(7-azabenzotriazol-1-yl)-*N,N,N′,N′*-tetramethyluronium-hexafluorphosphate) and 2 eq DIPEA (*N,N*-diisopropyl-ethyl-amine) in DMF for 2 h at r.t. The resulting HdpyTSCL-sC18 conjugate was cleaved from the resin with trifluoroacetic acid/triisopropylsilane/H_2O (95/2.5/2.5, v/v/v) and precipitated in ice cold diethyl ether. Then it was purified by preparative HPLC (column: Nucleodur C18ec; 100-5; Macherey-Nagel; solvent: 10-60% MeCN in H_2O (incl. 0.1% TFA) over 45 min, 6.0 mL/min flow rate). After purification a yield of 19.7 mg (8.08 µmol, 53.8%) was determined. The product was identified via HPLC-ESI MS (column: Nucleodur C18ec; 100-5; Macherey-Nagel; gradient: 10-60% MeCN in H_2O (incl. 0.1% formic acid) over 15 min; 0.6 mL/min flow rate). The identified molecular ions in Figure S1 correspond well with the calculated molecular weight (calculated: 2438.05 g/mol; experimental: 2438.63 g/mol).

1.7 Synthesis of [Pt(dpyTSCL-sC18)Cl]

HdpyTSCL-sC18 and K_2PtCl_4 were dissolved in H_2O (concentration of stocks: 5 mM; ratio: 1:1) and incubated for 2 h under shaking at r.t. Complex formation was UV-vis monitored through the typical absorption band at 480 nm and was verified by ESI mass spectrometry. The crude complex was desalted using a C18ec cartridge (Chromafix C18ec, Macherey-Nagel), solvent was evaporated and the red residue lyophilised. The HPLC-ESI-MS(+) analytics were shown in Figure 7 in the manuscript.

From a first batch prepared in the same way we received slightly different HPLC-ESI-MS(+) results. Figure S2 shows a detailed HPLC-ESI-MS(+) spectrum of this batch. The LC-MS analysis (Figure S2A) revealed the desired product [Pt(dpyTSCL-sC18)Cl] (marked in green), and the $[Pt(dpyTSCL\text{-}sC18)]^+$ (marked in blue), without coordinated chloride as in the second batch. However, an additional species species was detected which very probably corresponding to a trimer of the formula $[Pt(dpyTSCL\text{-}sC18]_3$ (marked in red).

----

2. Supplementary Figures and Tables:

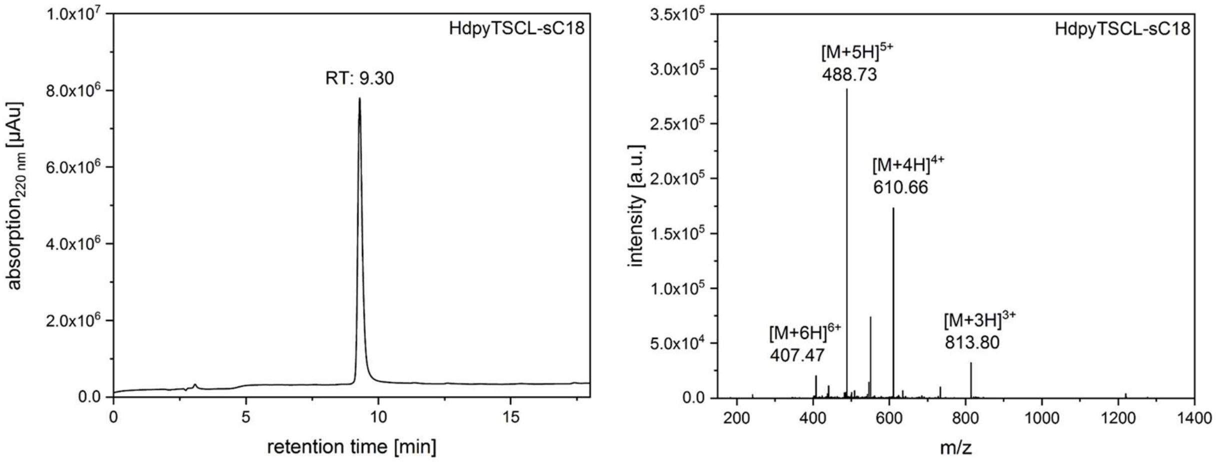

Figure S1. HPLC chromatogram and corresponding ESI mass spectrometry analysis of HdpyTSCL-sC18 conjugate after purification. The sample was recorded using a linear gradient from 10-60% MeCN in H_2O (incl. 0.1% trifluoroacetic acid) within 15 min.

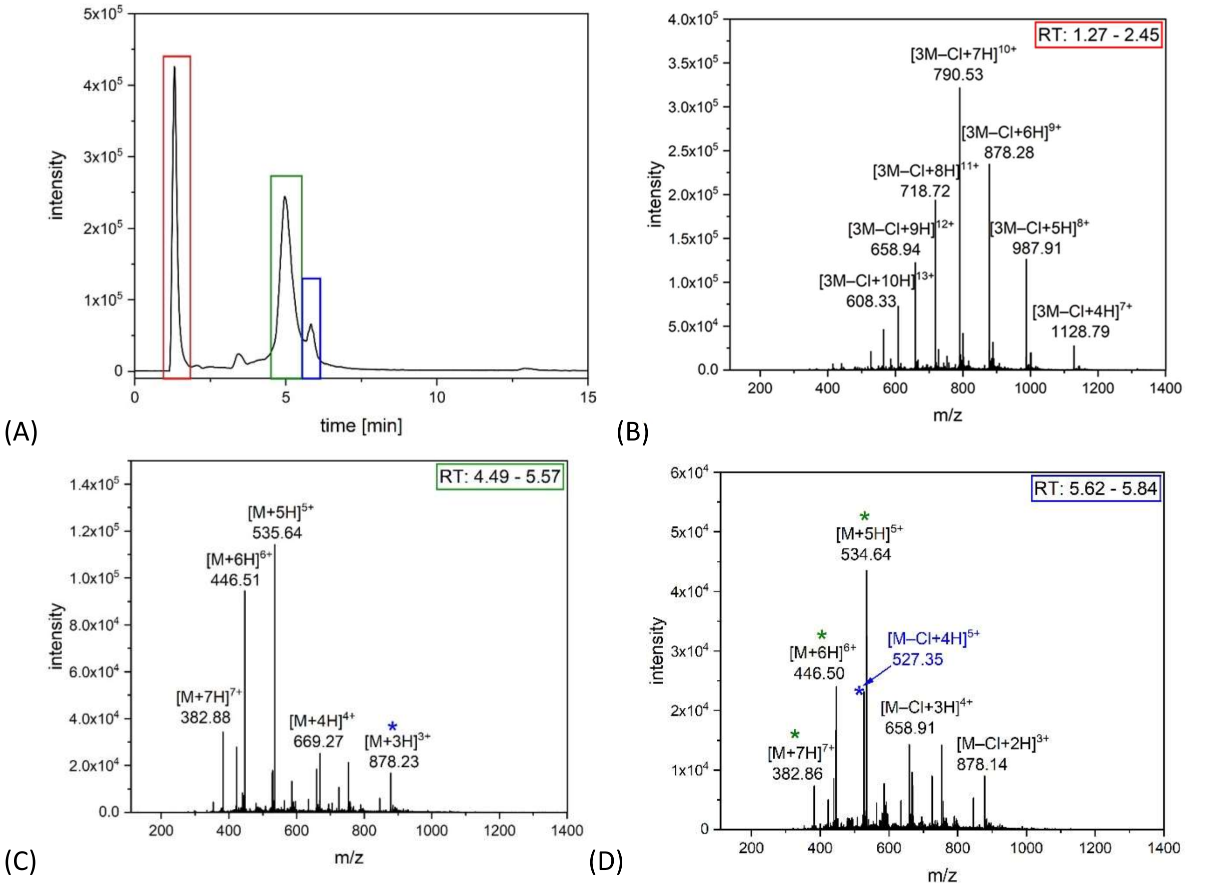

Figure S2. HPLC-ESI-MS(+) analysis of the [Pt(dpyTSCL-sC18)Cl] conjugate and by-products after desalting. (A) Ion current. (B) ESI-MS(+) for the red labelled peak at RT: 1.27–2.45. The labelled m/z values refer to a mass of 7895.16 g/mol corresponding presumably to a trimer [Pt(dpyTSCL-sC18]$_3$. (C) ESI-MS(+) for the green labelled peak at RT: 4.49–5.57. Labelled are m/z values which indicate a mass of 2673.13 g/mol*. This corresponds to the target conjugate [Pt(dpyTSCL-sC18)Cl] (calc.: 2666.40). The molecular ion marked with a blue asterisk is also found in (D). (D) ESI-MS(+) of the blue labelled peak at RT: 5.62–5.84. The labelled m/z values refer to a mass of 2631.60 g/mol*. This corresponds to the species [Pt(dpyTSCL-sC18)]$^+$ without the chloride ligand (calc.: 2631.43). The molecular ions marked with green asterisk are also found in the MS for the green labelled peak.

* For the mass calculation, only m/z values were considered, which could clearly be assigned with the theoretical calculated values (Table S1).

Table S1. Calculated and identified m/z values for [Pt(dpyTSCL-sC18)Cl] and [Pt(dpyTSCL-sC18)].

molecular ions	calculated m/z	found m/z
$[M]^{+}$	2667.40	-
$[M+2H]^{2+}$	1334.20	1333.37
$[M+3H]^{3+}$	889.80	889.47
$[M+4H]^{4+}$	667.60	669.27
$[M+5H]^{5+}$	534.28	535.64
$[M+6H]^{6+}$	445.40	446.51
$[M+7H]^{7+}$	381.91	382.88
$[M–Cl]^{+}$	2631.43	-
$[M–Cl+H]^{2+}$	1316.22	1316.88
$[M–Cl+2H]^{3+}$	877.81	878.14
$[M–Cl+3H]^{4+}$	658.61	658.91
$[M–Cl+4H]^{5+}$	527.10	527.35
$[M–Cl+5H]^{6+}$	439.41	439.65
$[3M–Cl]^{+}$	7895.30	-
$[3M–Cl+4H]^{7+}$	1128.47	1128.79
$[3M–Cl+5H]^{8+}$	987.54	987.91
$[3M–Cl+6H]^{8+}$	877.92	878.28
$[3M–Cl+7H]^{10+}$	790.23	790.53
$[3M–Cl+8H]^{11+}$	718.48	718.72
$[3M–Cl+9H]^{12+}$	658.69	658.94
$[3M–Cl+10H]^{13+}$	608.10	608.33

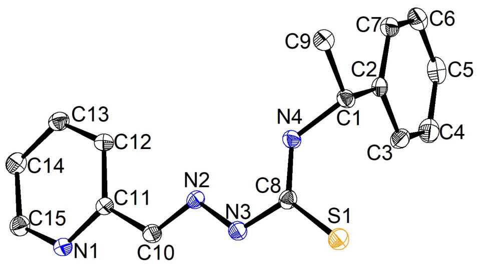

Figure S3. Molecular structure of HfpyTSCmB. Atoms are shown with 50% probability. H atoms are omitted for clarity.

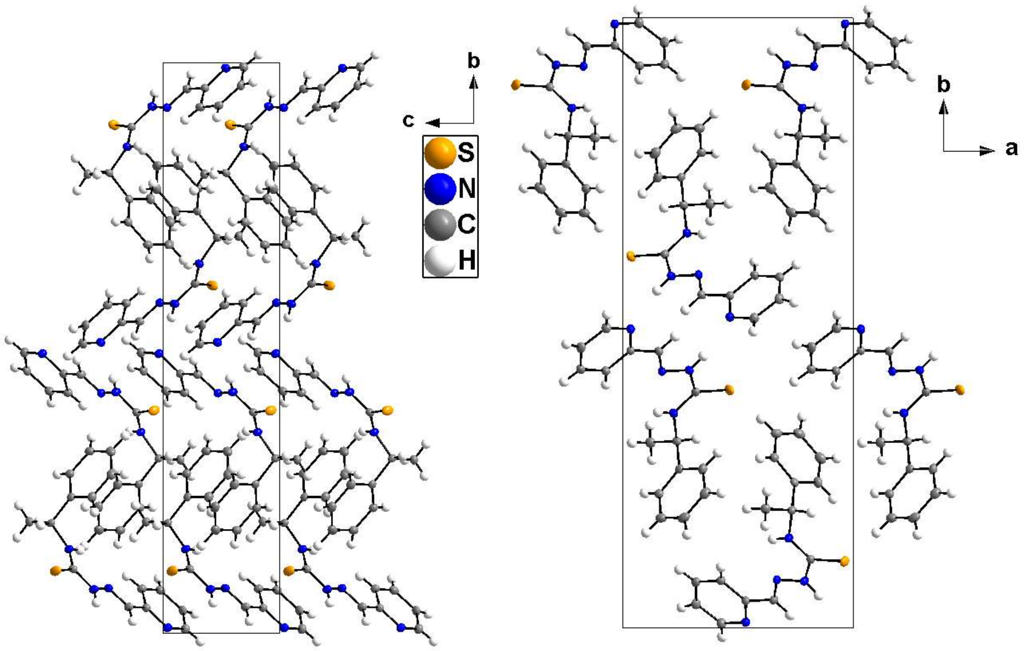

Figure S4. Crystal structure of HfpyTSCmB along the crystallographic *a*-axis (left) and *c*-axis (right).

Table S2. Crystal data and structure refinement for HfpyTSCmB.

empirical formula / formula weight	$C_{15}H_{14}N_4S$ / 284.38 g/mol
temperature / wavelength	100(1) K / 0.56076 Å
crystal system / space group	orthorhombic / $P2_12_12_1$
unit cell dimensions	*a* = 9.994(2) Å; *b* = 26.463(5) Å; *c* = 5.3980(11) Å
volume / Z / calculated density	1427.6(5) $Å^3$ / 16 / 2.316 g/cm^3
absorption coefficient / F(000)	4.328 mm^{-1} / 944
theta range for data collection	1.539 to 29.567°
limiting indices	–13 ≤ h ≤ 13, –36 ≤ k ≤ 36, –7 ≤ l ≤ 7
reflections collected / unique	16989 / 3961 [R_{int} = 0.0390]
completeness to theta = 25.242	100%
refinement method	full-matrix least-squares on F^2
data / restraints / parameters	3961 / 0 / 245
goodness-of-fit on F^2	0.741
final *R* indices [$I_0>2\sigma_{I0}$]	R_1 = 0.0406, wR_2 = 0.1124
R indices (all data)	R_1 = 0.0447, wR_2 = 0.1173
absolute structure parameter	0.78(3)
largest diff. peak and hole	0.337 and –0.478 e·Å^{-3}
CCDC	1954934

Table S3. Atomic coordinates (x 10^4) and equivalent isotropic displacement parameters (Å^2 x 10^3) for HfpyTSCmB. U(eq) is defined as one third of the trace of the orthogonalised U_{ij} tensor.

	x	y	z	U(eq)
S(1)	4636(1)	3905(1)	782(1)	24(1)
N(4)	2214(2)	3524(1)	1913(4)	18(1)
N(3)	2886(2)	4227(1)	4042(4)	18(1)
N(2)	1699(2)	4208(1)	5309(4)	16(1)
N(1)	345(2)	4901(1)	10359(4)	18(1)
C(8)	3165(2)	3872(1)	2279(4)	16(1)
C(2)	2466(2)	2610(1)	2096(5)	16(1)
C(3)	3473(2)	2607(1)	3897(5)	20(1)
C(13)	-1815(2)	4246(1)	9760(5)	19(1)
C(7)	1579(3)	2201(1)	1997(5)	18(1)

C(12)	-730(2)	4217(1)	8177(5)	17(1)
C(14)	-1810(3)	4599(1)	11663(5)	19(1)
C(15)	-710(3)	4920(1)	11878(5)	20(1)
C(11)	344(2)	4550(1)	8553(4)	16(1)
C(1)	2352(2)	3066(1)	382(4)	16(1)
C(9)	1205(3)	3043(1)	-1476(5)	19(1)
C(10)	1563(2)	4536(1)	7044(5)	18(1)
C(4)	3583(3)	2212(1)	5571(5)	25(1)
C(5)	2665(3)	1813(1)	5502(5)	24(1)
C(6)	1677(3)	1807(1)	3704(5)	22(1)

Table S4. Bond lengths [Å] and angles [°] for HfpyTSCmB.

S(1)-C(8)	1.679(2)	C(14)-C(15)	1.394(3)	C(12)-C(13)-C(14)	119.5(2)
N(4)-C(8)	1.338(3)	C(11)-C(10)	1.466(3)	C(6)-C(7)-C(2)	120.6(2)
N(4)-C(1)	1.473(3)	C(1)-C(9)	1.524(3)	C(13)-C(12)-C(11)	118.3(2)
N(3)-C(8)	1.366(3)	C(4)-C(5)	1.398(4)	C(15)-C(14)-C(13)	118.3(2)
N(3)-N(2)	1.370(3)	C(5)-C(6)	1.384(4)	N(1)-C(15)-C(14)	123.3(2)
N(2)-C(10)	1.285(3)	C(8)-N(4)-C(1)	125.6(2)	N(1)-C(11)-C(12)	122.6(2)
N(1)-C(15)	1.336(3)	C(8)-N(3)-N(2)	119.9(2)	N(1)-C(11)-C(10)	114.8(2)
N(1)-C(11)	1.347(3)	C(10)-N(2)-N(3)	115.5(2)	C(12)-C(11)-C(10)	122.7(2)
C(2)-C(7)	1.400(3)	C(15)-N(1)-C(11)	118.0(2)	N(4)-C(1)-C(9)	109.3(2)
C(2)-C(3)	1.400(3)	N(4)-C(8)-N(3)	115.5(2)	N(4)-C(1)-C(2)	108.5(2)
C(2)-C(1)	1.524(3)	N(4)-C(8)-S(1)	125.8(2)	C(9)-C(1)-C(2)	115.1(2)
C(3)-C(4)	1.387(4)	N(3)-C(8)-S(1)	118.5(2)	N(2)-C(10)-C(11)	120.6(2)
C(13)-C(12)	1.383(3)	C(7)-C(2)-C(3)	118.5(2)	C(3)-C(4)-C(5)	119.9(2)
C(13)-C(14)	1.388(3)	C(7)-C(2)-C(1)	122.8(2)	C(6)-C(5)-C(4)	119.7(2)
C(7)-C(6)	1.395(3)	C(3)-C(2)-C(1)	118.7(2)	C(5)-C(6)-C(7)	120.3(2)
C(12)-C(11)	1.404(3)	C(4)-C(3)-C(2)	121.0(2)		

Table S5. Anisotropic displacement parameters ($Å^2 \times 10^3$) for HfpyTSCmB. The anisotropic displacement factor exponent takes the form: $-2\pi^2[h^2a^{*2}U_{11}+2hka^*b^*U_{12}+\ldots]$.

	U11	U22	U33	U23	U13	U12
S(1)	18(1)	23(1)	30(1)	-4(1)	11(1)	-4(1)
N(4)	15(1)	17(1)	21(1)	-3(1)	5(1)	-1(1)
N(3)	15(1)	17(1)	22(1)	-2(1)	5(1)	-3(1)
N(2)	14(1)	16(1)	18(1)	0(1)	3(1)	0(1)
N(1)	16(1)	17(1)	20(1)	-3(1)	0(1)	-1(1)
C(8)	15(1)	16(1)	18(1)	2(1)	2(1)	1(1)
C(2)	15(1)	16(1)	17(1)	-2(1)	2(1)	1(1)
C(3)	16(1)	23(1)	21(1)	-2(1)	-2(1)	1(1)
C(13)	16(1)	19(1)	23(1)	2(1)	-1(1)	-2(1)
C(7)	18(1)	19(1)	19(1)	-2(1)	0(1)	0(1)
C(12)	16(1)	17(1)	19(1)	-1(1)	0(1)	-1(1)
C(14)	17(1)	24(1)	16(1)	1(1)	4(1)	-1(1)
C(15)	19(1)	22(1)	18(1)	-3(1)	1(1)	0(1)
C(11)	15(1)	16(1)	18(1)	-1(1)	0(1)	0(1)
C(1)	17(1)	16(1)	16(1)	-2(1)	2(1)	-1(1)
C(9)	21(1)	21(1)	16(1)	1(1)	0(1)	0(1)
C(10)	15(1)	17(1)	21(1)	-1(1)	3(1)	-3(1)
C(4)	24(1)	29(1)	21(1)	-1(1)	-2(1)	6(1)
C(5)	31(1)	20(1)	21(1)	2(1)	4(1)	7(1)
C(6)	26(1)	17(1)	23(1)	-1(1)	2(1)	-1(1)

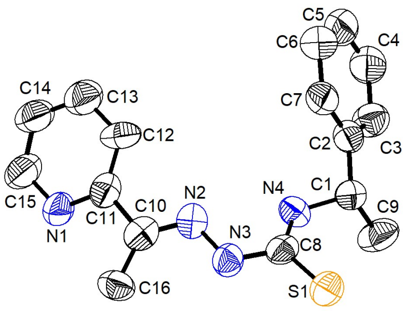

Figure S5. Molecular structure of HapyTSCmB. Atoms are shown with 50% probability. H atoms are omitted for clarity.

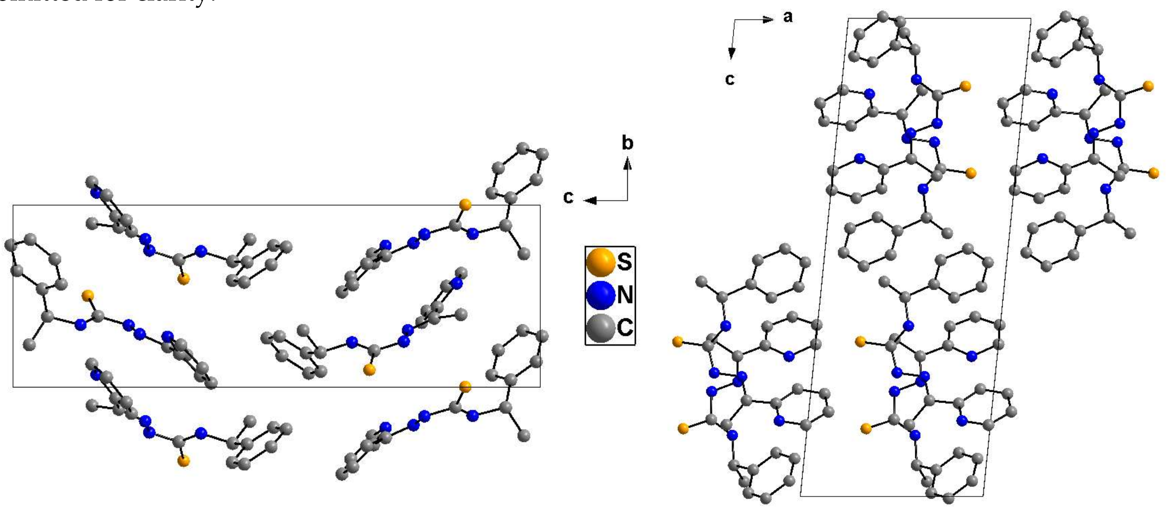

Figure S6. Crystal structure of HapyTSCmB along the crystallographic *a*-axis (left) and *b*-axis (right).

Table S6. Crystal data and structure refinement for HdpyTSCmB.

empirical formula / formula weight	$C_{16}H_{18}N_4S$ / 298.41 g/mol
temperature / wavelength	293(2) K / 0.71073 Å
crystal system / space group	orthorhombic / $P2_1$
unit cell dimensions	a = 8.7895(6) Å; b = 7.9268(4) Å; c = 23.0863(19) Å; β = 95.750(6)
Volume / Z / calculated density	1600.39(19) Å^3 / 4 / 1.1631 g/cm^3
absorption coefficient / F(000)	0.178 mm^{-1} / 560.7
theta range for data collection	3.54 to 53.64°
limiting indices	$-11 \leq h \leq 11$, $-10 \leq k \leq 8$, $-29 \leq l \leq 29$
reflections collected / unique	36405 / 6509 [R_{int}= 0.2299]
completeness to theta = 25.242	100%
refinement method	charge flip
data / restraints / parameters	6509 / 1 / 379
goodness-of-fit on F^2	0.796
final R indices [$I_0>2\sigma_{I0}$]	R_1 = 0.0703, wR_2 = 0.1619
R indices (all data)	R_1 = 0.2186, wR_2 = 0.2341
largest diff. peak and hole	0.55 and –0.43 e·Å^{-3}
CCDC	1954950

Table S7. Atomic coordinates (x 10^4) and equivalent isotropic displacement parameters (Å^2 x 10^3) for HapyTSCmB. U(eq) is defined as one third of the trace of the orthogonalised U_{ij} tensor.

	x	*y*	*z*	**U(eq)**
S1	-7552(2)	-4047(3)	-3237.5(1)	76.5(7)
N2	-3867(8)	-1873(8)	-2508(3)	70.8(19)
C8	-5872(9)	-3003(9)	-3147(4)	65(2)

N3	-5387(8)	-2490(8)	-2587(3)	66.7(18)
N4	-5017(7)	-2538(8)	-3568(3)	61.5(17)
N1	-1566(7)	697(9)	-1583(3)	77(2)
C10	-3464(9)	-1214(10)	-2007(4)	67(2)
C1	-5371(9)	-3088(10)	-4169(3)	66(2)
C7	-2678(10)	-4132(11)	-4160(4)	77(2)
C2	-3857(9)	-3125(10)	-4442(3)	65(2)
C3	-3619(10)	-2212(12)	-4945(4)	82(3)
C13	766(11)	-825(13)	-2149(5)	94(3)
C9	-6591(10)	-1880(13)	-4494(4)	92(3)
C14	1075(10)	550(13)	-1774(5)	93(3)
C11	-1844(10)	-613(11)	-1948(4)	73(2)
C12	-738(10)	-1444(12)	-2247(4)	79(3)
C15	-96(10)	1261(12)	-1495(4)	91(3)
C5	-1025(11)	-3268(13)	-4899(5)	96(3)
C4	-2198(12)	-2304(13)	-5182(4)	95(3)
C16	-4436(10)	-1010(11)	-1501(4)	76(3)
C6	-1245(10)	-4233(14)	-4389(5)	103(3)

Table S8. Bond lengths [Å] and angles [deg] for HapyTSCmB.

S1-C8	1.687(8)	C13-C12	1.407(12)	C9-C1-C2	113.2(7)
N2-N3	1.417(9)	C14-C15	1.387(12)	C6-C7-C2	119.8(8)
N2-C10	1.285(9)	C11-C12	1.411(12)	C7-C2-C1	116.6(7)
C8-N3	1.383(10)	C5-C4	1.393(13)	C3-C2-C1	122.7(7)
C8-N4	1.337(10)	C5-C6	1.433(13)	C3-C2-C7	120.7(8)
N4-C1	1.458(9)	C10-N2-N3	115.3(7)	C4-C3-C2	120.3(8)
N1-C11	1.344(10)	N3-C8-S1	116.4(7)	C12-C13-C14	119.4(9)
N1-C15	1.363(10)	N4-C8-S1	126.4(7)	C15-C14-C13	119.7(9)
C10-C11	1.494(11)	N4-C8-N3	117.1(7)	C10-C11-N1	114.6(8)
C10-C16	1.524(11)	C8-N3-N2	114.9(6)	C12-C11-N1	125.2(8)
C1-C2	1.530(10)	C1-N4-C8	121.8(6)	C12-C11-C10	120.2(8)
C1-C9	1.572(11)	C15-N1-C11	117.2(8)	C11-C12-C13	116.2(9)
C7-C2	1.414(11)	C11-C10-N2	112.8(8)	C14-C15-N1	122.3(9)
C7-C6	1.416(12)	C16-C10-N2	127.4(8)	C6-C5-C4	121.6(9)
C2-C3	1.401(11)	C16-C10-C11	119.9(8)	C5-C4-C3	119.1(9)
C3-C4	1.416(12)	C2-C1-N4	106.7(6)	C5-C6-C7	118.4(9)
C13-C14	1.402(13)	C9-C1-N4	110.1(7)		

Table S9. Anisotropic displacement parameters ($Å^2 \times 10^3$) for HapyTSCmB. The Anisotropic displacement factor exponent takes the form: $-2\pi^2[h^2a^{*2}U_{11}+2hka^*b^*U_{12}+\ldots]$.

Atom	U_{11}	U_{22}	U_{33}	U_{12}	U_{13}	U_{23}
S1	66.3(2)	76.6(2)	86.7(2)	-14.4(2)	8.4(11)	-7.5(14)
N2	72(5)	72(5)	66(5)	-4(4)	0(4)	-3(4)
C8	54(5)	44(5)	94(6)	11(4)	-2(5)	-10(5)
N3	64(4)	66(5)	69(5)	-7(4)	4(3)	-3(4)
N4	69(4)	61(4)	56(4)	-2(4)	16(3)	-5(3)
N1	60(4)	73(5)	94(5)	-7(4)	-2(4)	-23(4)
C10	65(5)	47(5)	87(6)	-1(4)	1(5)	11(5)
C1	57(5)	80(6)	61(5)	6(4)	6(4)	-4(4)
C7	77(5)	73(6)	80(6)	12(6)	1(5)	-17(5)
C2	69(5)	64(5)	61(5)	0(4)	6(4)	-5(4)
C3	69(6)	92(7)	85(6)	-2(5)	12(5)	5(6)
C13	74(7)	93(8)	112(8)	12(6)	0(6)	-7(7)
C9	62(5)	113(8)	99(7)	22(5)	1(5)	22(6)
C14	61(6)	99(9)	117(8)	11(6)	4(5)	-9(7)

C11	66(6)	78(6)	72(6)	-1(5)	-8(4)	0(5)
C12	55(5)	93(7)	91(6)	21(5)	21(5)	14(5)
C15	61(6)	99(8)	110(8)	2(5)	-6(5)	-3(6)
C5	77(7)	122(9)	89(7)	-21(6)	13(6)	-25(7)
C4	85(7)	114(9)	87(7)	-10(7)	13(6)	-11(7)
C6	75(6)	132(10)	101(8)	9(7)	11(6)	-26(8)

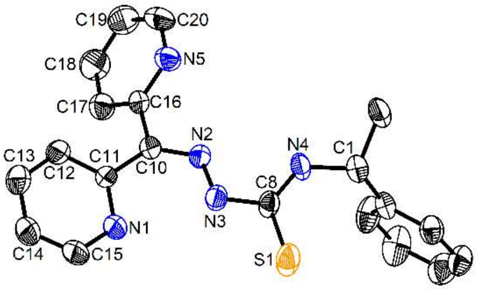

Figure S7. Molecular structure of HdpyTSCmB·MeOH. Atoms are shown with 50% probability. H atoms are omitted for clarity.

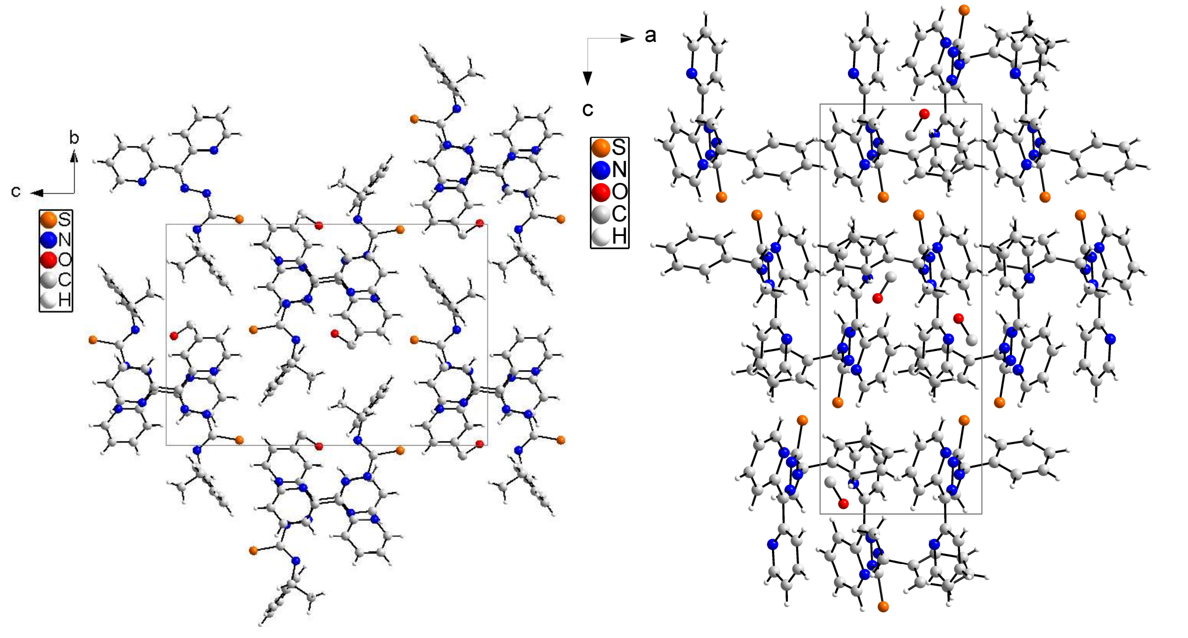

Figure S8. Crystal structure of HdpyTSCmB·MeOH along the crystallographic *a*-axis (left) and *b*-axis (right).

Table S10. Crystal data and structure refinement for HdpyTSCmB·MeOH.

empirical formula / formula weight	$C_{21}H_{17}N_5OS$ / 387.46 g/mol
temperature / wavelength	293(2) K / 0.71073 Å
crystal system / space group	orthorhombic / $P2_12_12_1$
unit cell dimensions	*a* = 7.7512(4) Å; *b* = 13.4952(11) Å; *c* = 19.6265(11) Å
Volume / Z / calculated density	2053.0(2) $Å^3$ / 4 / 1.254 g/cm^3
absorption coefficient / F(000)	0.178 mm^{-1} / 808
theta range for data collection	1.831 to 26.954°
limiting indices	$-9 \le h \le 8, -17 \le k \le 17, -24 \le l \le 24$
reflections collected / unique	37979 / 4394 [R_{int}= 0.0910]
completeness to θ = 25.242	100%
refinement method	full-matrix least-squares on F^2
data / restraints / parameters	4394 / 0 / 254

goodness-of-fit on F^2	1.102
final R indices [$I_0>2\sigma_{I0}$]	R_1 = 0.0676, wR_2 = 0.1614
R indices (all data)	R_1 = 0.0926, wR_2 = 0.1729
absolute structure parameter	0.97(5)
largest diff. peak and hole	0.360 and –0.192 e·Å^{-3}
CCDC	1954933

Table S11. Atomic coordinates (x 10^4) and equivalent isotropic displacement parameters (Å^2 x 10^3) for HdpyTSCmB·MeOH. U(eq) is defined as one third of the trace of the orthogonalised U_{ij} tensor.

	x	y	z	U(eq)
S(1)	3906(3)	4767(1)	2281(1)	69(1)
N(4)	3605(6)	5230(3)	961(2)	42(1)
N(3)	3227(6)	3595(3)	1258(2)	43(1)
O(1)	1444(6)	4949(3)	-245(2)	62(1)
N(1)	2762(6)	1649(3)	1488(2)	47(1)
C(10)	2717(6)	2482(3)	390(2)	35(1)
C(8)	3565(7)	4551(4)	1448(2)	39(1)
N(2)	3144(5)	3366(3)	578(2)	35(1)
C(16)	2753(7)	2345(3)	-368(2)	36(1)
C(11)	2201(6)	1642(3)	832(2)	35(1)
N(5)	2066(6)	3071(3)	-743(2)	45(1)
C(2)	5772(7)	6516(3)	1258(2)	38(1)
C(1)	3919(7)	6277(3)	1108(3)	43(1)
C(7)	6188(7)	7305(4)	1681(3)	45(1)
C(18)	3648(9)	1460(5)	-1354(3)	63(2)
C(12)	1123(7)	900(4)	584(3)	45(1)
C(17)	3552(7)	1527(4)	-653(3)	47(1)
C(14)	1224(8)	153(4)	1679(3)	60(2)
C(3)	7131(8)	6006(4)	956(3)	59(2)
C(6)	7872(8)	7584(4)	1782(3)	53(1)
C(20)	2162(9)	2977(5)	-1420(3)	61(2)
C(13)	623(8)	145(5)	1016(3)	58(2)
C(9)	3335(9)	6884(4)	479(3)	61(2)
C(15)	2254(8)	904(4)	1888(3)	53(1)
C(5)	9184(9)	7066(5)	1480(3)	62(2)
C(21)	628(11)	5463(5)	-771(4)	80(2)
C(19)	2925(9)	2188(5)	-1744(3)	64(2)
C(4)	8809(8)	6275(5)	1067(4)	69(2)

Table S12. Bond lengths [Å] and angles [deg] for HdpyTSCmB·MeOH.

S(1)-C(8)	1.681(5)	C(12)-C(13)	1.381(7)	C(12)-C(11)-C(10)	120.5(4)
N(4)-C(8)	1.324(6)	C(14)-C(15)	1.353(8)	C(16)-N(5)-C(20)	117.1(5)
N(4)-C(1)	1.463(6)	C(14)-C(13)	1.380(8)	C(7)-C(2)-C(3)	117.3(5)
N(3)-C(8)	1.368(6)	C(3)-C(4)	1.368(9)	C(7)-C(2)-C(1)	120.3(4)
N(3)-N(2)	1.372(5)	C(6)-C(5)	1.369(9)	C(3)-C(2)-C(1)	122.3(5)
O(1)-C(21)	1.397(7)	C(20)-C(19)	1.374(8)	N(4)-C(1)-C(2)	113.9(4)
N(1)-C(15)	1.335(6)	C(5)-C(4)	1.373(9)	N(4)-C(1)-C(9)	107.8(4)
N(1)-C(11)	1.358(6)	C(8)-N(4)-C(1)	122.0(4)	C(2)-C(1)-C(9)	108.8(4)
C(10)-N(2)	1.293(6)	C(8)-N(3)-N(2)	119.1(4)	C(6)-C(7)-C(2)	121.2(5)
C(10)-C(11)	1.482(6)	C(15)-N(1)-C(11)	117.3(4)	C(19)-C(18)-C(17)	119.3(6)
C(10)-C(16)	1.500(6)	N(2)-C(10)-C(11)	127.4(4)	C(13)-C(12)-C(11)	119.0(5)
C(16)-N(5)	1.336(6)	N(2)-C(10)-C(16)	113.1(4)	C(18)-C(17)-C(16)	118.6(5)
C(16)-C(17)	1.384(7)	C(11)-C(10)-C(16)	119.5(4)	C(15)-C(14)-C(13)	119.4(5)
C(11)-C(12)	1.393(7)	N(4)-C(8)-N(3)	117.4(4)	C(4)-C(3)-C(2)	121.4(5)
N(5)-C(20)	1.337(7)	N(4)-C(8)-S(1)	125.3(4)	C(5)-C(6)-C(7)	120.3(5)

C(2)-C(7)	1.388(7)	N(3)-C(8)-S(1)	117.3(3)	N(5)-C(20)-C(19)	123.9(5)
C(2)-C(3)	1.392(7)	C(10)-N(2)-N(3)	119.8(4)	C(14)-C(13)-C(12)	118.5(5)
C(2)-C(1)	1.502(8)	N(5)-C(16)-C(17)	122.8(5)	N(1)-C(15)-C(14)	124.0(5)
C(1)-C(9)	1.549(7)	N(5)-C(16)-C(10)	116.6(4)	C(6)-C(5)-C(4)	119.7(6)
C(7)-C(6)	1.373(8)	C(17)-C(16)-C(10)	120.5(4)	C(18)-C(19)-C(20)	118.3(5)
C(18)-C(19)	1.366(9)	N(1)-C(11)-C(12)	121.8(4)	C(3)-C(4)-C(5)	120.2(6)
C(18)-C(17)	1.381(8)	N(1)-C(11)-C(10)	117.6(4)		

Table S13. Anisotropic displacement parameters (Å ^{2} x 10^{3}) for HdpyTSCmB·MeOH. The anisotropic displacement factor exponent takes the form: $-2\pi^{2}[h^{2}a^{*2}U_{11}+2hka^{*}b^{*}U_{12}+\ldots]$.

	U11	U22	U33	U23	U13	U12
S(1)	123(2)	48(1)	35(1)	-4(1)	3(1)	-29(1)
N(4)	51(3)	26(2)	49(2)	3(2)	1(2)	-4(2)
N(3)	58(3)	35(2)	35(2)	-1(2)	-1(2)	-9(2)
O(1)	87(3)	49(2)	51(2)	6(2)	-14(2)	10(2)
N(1)	65(3)	38(2)	37(2)	7(2)	-6(2)	-9(2)
C(10)	39(3)	30(2)	38(2)	0(2)	-3(2)	1(2)
C(8)	44(3)	35(2)	39(2)	-1(2)	5(2)	-12(2)
N(2)	38(2)	32(2)	35(2)	-1(2)	-1(2)	-3(2)
C(16)	40(3)	29(2)	38(2)	3(2)	-3(2)	-5(2)
C(11)	38(3)	31(2)	35(2)	-1(2)	-6(2)	0(2)
N(5)	55(3)	43(2)	36(2)	2(2)	-3(2)	4(2)
C(2)	42(3)	26(2)	45(2)	3(2)	8(2)	1(2)
C(1)	48(3)	31(2)	50(3)	0(2)	2(3)	-1(2)
C(7)	47(3)	36(3)	53(3)	-5(2)	5(2)	4(3)
C(18)	83(5)	59(4)	47(3)	-13(3)	4(3)	11(3)
C(12)	41(3)	45(3)	48(3)	5(2)	-9(2)	-10(2)
C(17)	52(3)	42(3)	46(3)	-2(2)	0(2)	3(3)
C(14)	82(4)	46(3)	52(3)	15(2)	-4(3)	-17(3)
C(3)	53(4)	46(3)	77(4)	-22(3)	17(3)	-5(3)
C(6)	59(4)	42(3)	58(3)	-13(3)	-1(3)	-2(3)
C(20)	81(4)	59(4)	42(3)	11(3)	-15(3)	5(3)
C(13)	59(4)	51(3)	64(3)	8(3)	-6(3)	-21(3)
C(9)	67(4)	35(3)	82(4)	17(3)	-15(3)	-8(3)
C(15)	75(4)	47(3)	36(3)	12(2)	-5(3)	-5(3)
C(5)	47(3)	62(4)	78(4)	-5(3)	-5(3)	-6(3)
C(21)	111(6)	47(4)	81(5)	13(3)	-23(4)	14(4)
C(19)	87(5)	66(4)	39(3)	-6(3)	0(3)	-1(4)
C(4)	44(4)	60(4)	103(5)	-22(4)	20(4)	-2(3)

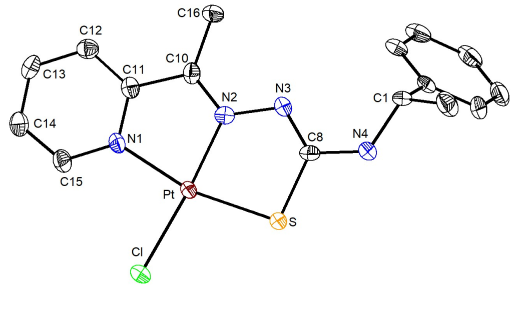

Figure S9. Molecular structure of [Pt(apyTSCmB)Cl]. Atoms are shown with 50% probability. H atoms are omitted for clarity.

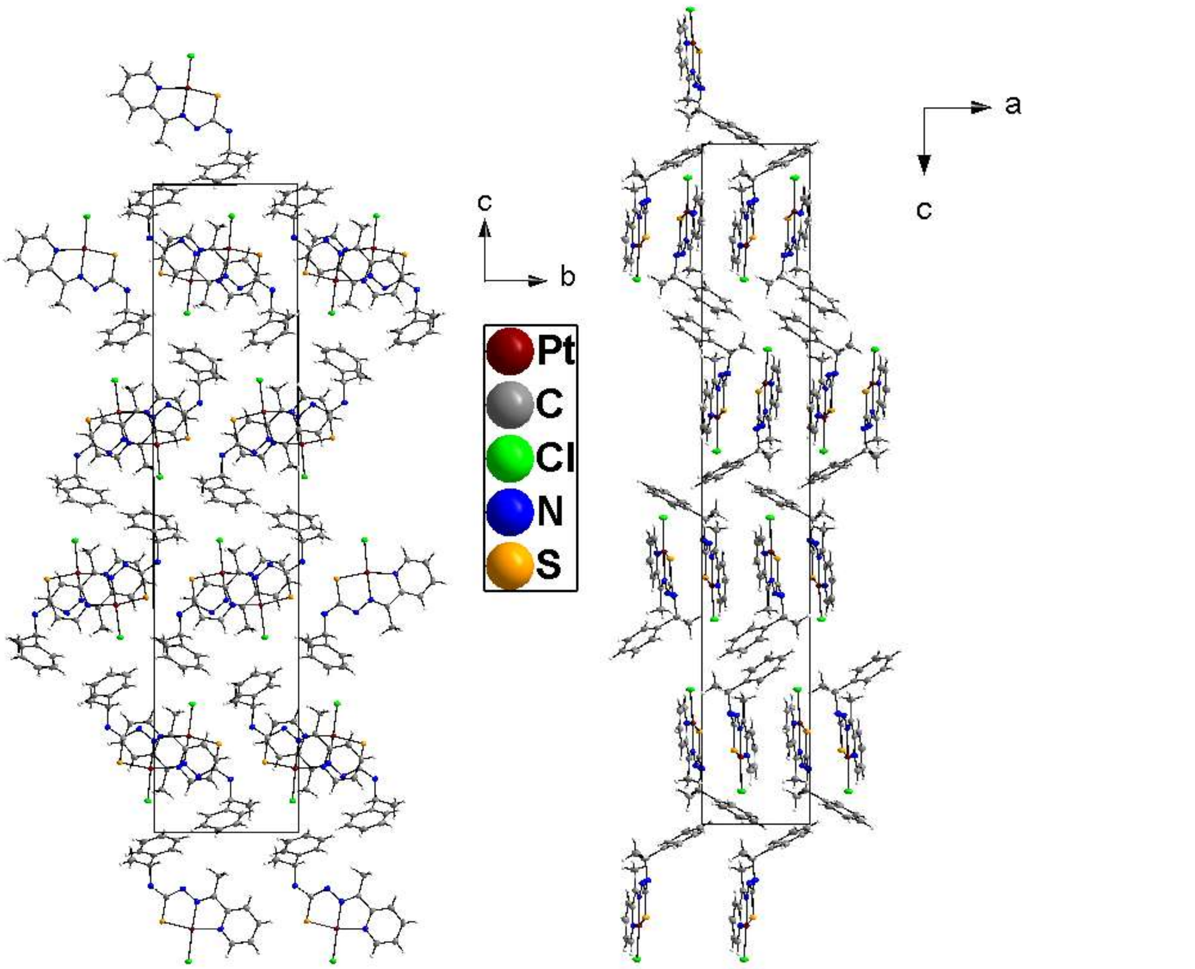

Figure S10. Crystal structure of [Pt(apyTSCmB)Cl] along the crystallographic *a*-axis (left) and *b*-axis (right).

Table S14. Crystal data and structure refinement for [Pt(apyTSCmB)Cl].

empirical formula / formula weight	$C_{16}H_{16}ClN_4PtS$ / 512.92 g/mol
temperature / wavelength	100(2) K / 1.54178 Å
crystal system, space group	orthorhombic / $P2_12_12_1$
unit cell dimensions	a = 7.2536(10) Å; b = 10.2592(14) Å; c = 46.003(6) Å
volume / Z / density (calculated)	3423.4(8) Å^3 / 8 / 1.990 g/cm^3
absorption coefficient / F(000)	17.895 mm^{-1} / 1952
theta range for data collection	3.843 to 72.411°.
index ranges	$-8 \le h \le 8$, $-12 \le k \le 12$, $-56 \le l \le 54$
reflections collected	35614
independent reflections	6735 [R_{int}= 0.0502]
completeness to theta = 67.679°	100.0%
refinement method	Full-matrix least-squares on F^2
data / restraints / parameters	6735 / 0 / 419
goodness-of-fit on F^2	1.190
final R indices [I_0>2σ_{I0}]	R_1 = 0.0249, wR_2 = 0.0621
R indices (all data)	R_1 = 0.0261, wR_2 = 0.0621
absolute structure parameter	0.103(4)
largest diff. peak and hole	1.004 and –1.225 e·Å^{-1}
CCDC	1954935

Table S15. Atomic coordinates (x 10^4) and equivalent isotropic displacement parameters (Å^2 x 10^3) for [Pt(apyTSCmB)Cl]. U(eq) is defined as one third of the trace of the orthogonalised U_{ij} tensor.

	x	y	z	U(eq)
Pt(1)	1418(1)	4755(1)	4009(1)	18(1)
C(8)	2050(10)	2858(7)	3531(2)	21(1)
Cl(1)	1348(3)	4566(2)	4511(1)	28(1)
C(44)	-1235(15)	3083(9)	2562(2)	40(2)
C(33)	366(13)	3030(8)	2728(2)	31(2)
Cl(2)	6110(3)	7659(2)	3015(1)	27(1)
C(3)	-407(12)	876(8)	2878(2)	26(2)
C(6)	3737(12)	657(8)	2963(2)	30(2)
C(14)	151(12)	8831(8)	4154(2)	29(2)
N(1)	830(9)	6712(6)	3986(1)	22(1)
N(3)	1797(9)	3973(6)	3395(1)	20(1)
C(10)	1145(10)	6151(6)	3483(2)	20(1)
C(2)	814(11)	1926(7)	2889(1)	22(2)
C(11)	793(10)	7142(7)	3705(2)	22(1)
C(5)	-2404(13)	2031(10)	2553(2)	37(2)
C(4)	-1969(12)	916(9)	2710(2)	29(2)
C(15)	494(11)	7532(8)	4206(2)	27(2)
C(12)	426(11)	8430(7)	3646(2)	26(2)
C(13)	118(11)	9301(7)	3874(2)	27(2)
S(1)	2005(2)	2633(2)	3913(1)	21(1)
S(2)	5268(3)	9478(2)	3631(1)	20(1)
C(16)	1139(13)	6471(7)	3166(2)	27(2)
C(1)	2609(11)	1850(7)	3051(2)	24(2)
N(4)	2348(10)	1787(6)	3370(1)	23(1)
N(2)	1458(8)	4998(6)	3585(1)	19(1)

Table S16. Bond lengths [Å] and angles [deg] for [Pt(apyTSCmB)Cl].

Pt(1)-N(2)	1.966(5)	C(11)-C(12)	1.375(11)	N(2)-C(10)-C(16)	123.7(6)
Pt(1)-N(1)	2.055(6)	C(5)-C(4)	1.391(13)	C(11)-C(10)-C(16)	121.8(6)
Pt(1)-S(1)	2.2623(18)	C(12)-C(13)	1.397(11)	C(33)-C(2)-C(3)	117.4(8)
Pt(1)-Cl(1)	2.3173(16)	C(1)-N(4)	1.481(9)	C(33)-C(2)-C(1)	120.6(7)
C(8)-N(3)	1.317(10)	N(2)-Pt(1)-N(1)	80.2(2)	C(3)-C(2)-C(1)	121.9(7)
C(8)-N(4)	1.341(10)	N(2)-Pt(1)-S(1)	85.75(17)	N(1)-C(11)-C(12)	120.1(7)
C(8)-S(1)	1.775(8)	N(1)-Pt(1)-S(1)	165.82(18)	N(1)-C(11)-C(10)	115.6(6)
C(44)-C(5)	1.373(14)	N(2)-Pt(1)-Cl(1)	177.50(17)	C(12)-C(11)-C(10)	124.3(7)
C(44)-C(33)	1.391(13)	N(1)-Pt(1)-Cl(1)	97.35(18)	C(44)-C(5)-C(4)	119.4(8)
C(33)-C(2)	1.392(11)	S(1)-Pt(1)-Cl(1)	96.75(6)	C(3)-C(4)-C(5)	120.3(8)
C(3)-C(4)	1.372(12)	N(3)-C(8)-N(4)	118.2(6)	N(1)-C(15)-C(14)	120.9(7)
C(3)-C(2)	1.395(11)	N(3)-C(8)-S(1)	125.6(6)	C(11)-C(12)-C(13)	119.8(7)
C(6)-C(1)	1.527(11)	N(4)-C(8)-S(1)	116.2(5)	C(14)-C(13)-C(12)	118.5(7)
C(14)-C(15)	1.376(11)	C(5)-C(44)-C(33)	120.0(8)	C(8)-S(1)-Pt(1)	94.1(2)
C(14)-C(13)	1.376(12)	C(44)-C(33)-C(2)	121.3(8)	N(4)-C(1)-C(2)	112.5(6)
N(1)-C(15)	1.336(9)	C(4)-C(3)-C(2)	121.4(8)	N(4)-C(1)-C(6)	107.2(6)
N(1)-C(11)	1.368(9)	C(15)-C(14)-C(13)	120.2(7)	C(2)-C(1)-C(6)	112.0(6)
N(3)-N(2)	1.391(8)	C(15)-N(1)-C(11)	120.5(6)	C(8)-N(4)-C(1)	122.0(6)
C(10)-N(2)	1.293(9)	C(15)-N(1)-Pt(1)	127.9(5)	C(10)-N(2)-N(3)	119.6(6)
C(10)-C(11)	1.462(10)	C(11)-N(1)-Pt(1)	111.5(5)	C(10)-N(2)-Pt(1)	118.2(5)
C(10)-C(16)	1.496(9)	C(8)-N(3)-N(2)	112.4(6)	N(3)-N(2)-Pt(1)	122.1(4)
C(2)-C(1)	1.502(11)	N(2)-C(10)-C(11)	114.5(6)		

Table S17. Anisotropic displacement parameters ($Å^2 \times 10^3$) for [Pt(apyTSCmB)Cl]. The anisotropic displacement factor exponent takes the form: $-2\pi^2[h^2a^{*2}U_{11}+2hka^*b^*U_{12}+\ldots]$.

	U11	U22	U33	U23	U13	U12
Pt(1)	23(1)	17(1)	14(1)	-1(1)	-2(1)	0(1)

C(8)	18(3)	25(3)	20(3)	2(3)	-8(3)	3(3)
Cl(1)	43(1)	26(1)	15(1)	0(1)	-2(1)	4(1)
C(44)	45(6)	50(5)	25(4)	14(4)	-6(4)	9(4)
C(33)	41(5)	32(4)	22(4)	7(3)	-1(3)	-3(4)
Cl(2)	39(1)	29(1)	14(1)	0(1)	1(1)	5(1)
C(3)	33(4)	24(4)	20(3)	-2(3)	7(3)	3(3)
C(6)	34(4)	35(4)	21(3)	-7(3)	-1(3)	6(4)
C(14)	31(4)	21(4)	34(4)	-8(3)	-3(3)	1(3)
N(1)	27(3)	20(3)	18(3)	-4(2)	-3(3)	-1(3)
N(3)	24(3)	21(3)	16(3)	-5(2)	0(2)	1(2)
C(10)	18(4)	19(3)	22(3)	4(3)	1(3)	1(3)
C(2)	31(4)	23(3)	11(3)	-2(2)	1(3)	2(3)
C(11)	19(3)	25(3)	22(3)	2(3)	1(3)	-1(3)
C(5)	35(5)	59(6)	17(4)	-3(4)	-5(3)	5(4)
C(4)	30(4)	40(4)	15(3)	-12(3)	4(3)	0(4)
C(15)	33(4)	23(4)	25(4)	-3(3)	-3(3)	-4(3)
C(12)	26(4)	23(4)	27(4)	3(3)	-1(3)	-3(3)
C(13)	25(4)	16(3)	39(4)	0(3)	-4(3)	-2(3)
S(1)	28(1)	18(1)	16(1)	-1(1)	-2(1)	2(1)
S(2)	27(1)	18(1)	16(1)	-1(1)	-2(1)	3(1)
C(16)	39(5)	25(4)	18(3)	5(3)	3(3)	4(4)
C(1)	29(4)	25(4)	19(3)	-2(3)	6(3)	0(3)
N(4)	29(3)	22(3)	19(3)	-2(2)	-1(3)	4(3)
N(2)	17(3)	20(3)	19(3)	2(2)	4(2)	3(2)

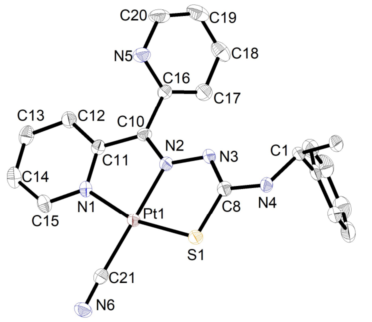

(A)

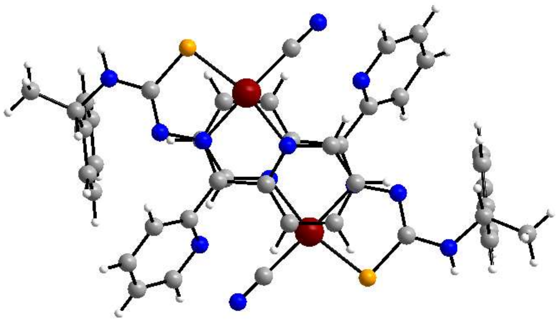

(B)

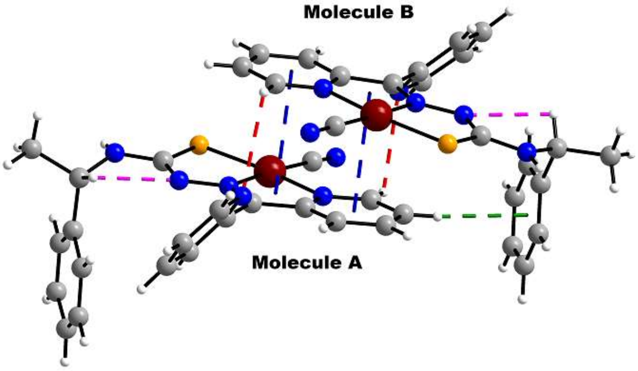

(C)

Figure S11. Molecular structure of [Pt(dpyTSCmB)(CN)]. **(A)**: one out of two independent molecules. Atoms are shown with 50% probability. H atoms are omitted for clarity. B and C: Stacking and further intermolecular interactions between the two independent molecules in the crystal: **blue** = π-π: 3.6493(1) and 3.5155(1) Å; **pink** = N3···HC1: 2.425(4) and 2.503(4) Å; **red** = N5···HC15: 2.787(6) and 2.907(6) Å; **green** = π···HC14: 2.505(4) Å.

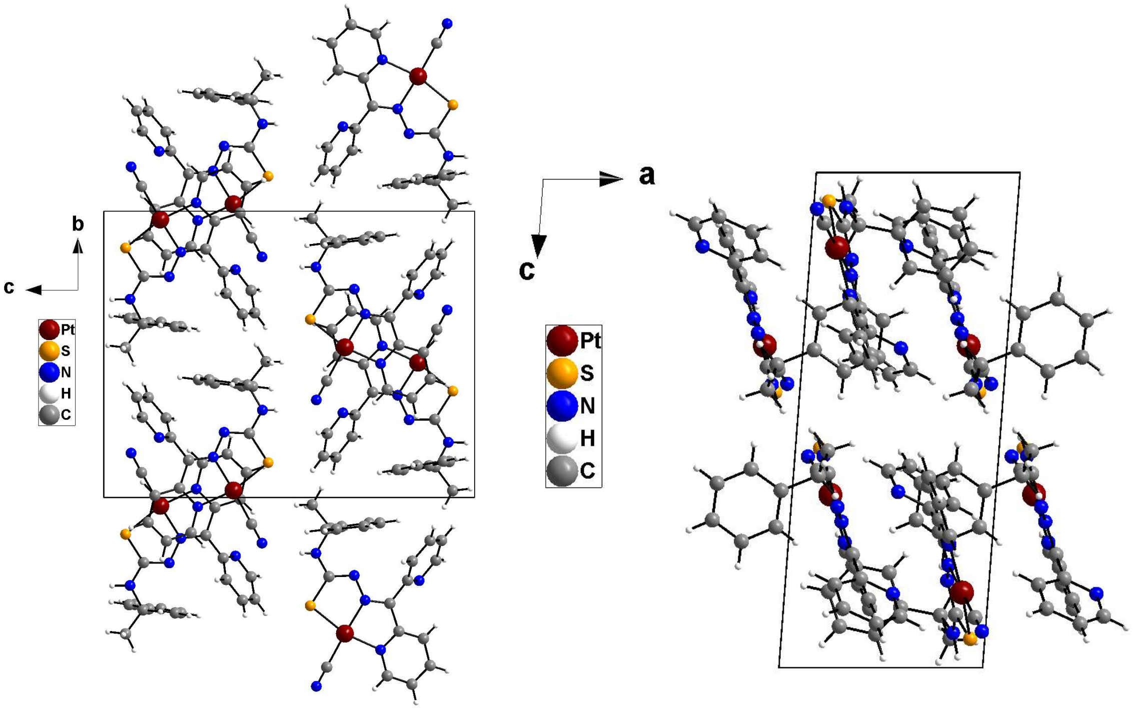

Figure S12. Crystal structure of [Pt(dpyTSCmB)(CN)] along the crystallographic *a*-axis (left) and *b*-axis (right).

Table S18. Crystal data and structure refinement for [Pt(dpyTSCmB)(CN)].

Empirical formula / Formula weight	$C_{21}H_{18}N_6PtS$ / 581.56 g/mol
Temperature / Wavelength	150(2) K / 1.54178 Å
Crystal system / Space group	Monoclinic / $P2_1$
Unit Cell Dimensions	a = 7.6316(3) Å, b = 14.3698(6) Å c = 18.6158(7) Å, β =94.3320(10)
Volume / Z	2035.66(14) Å^3 / 4 (two independent molecules)
ρ_{calc} / μ / F(000)	1.8975 g/cm^3 / 14.011 mm^{-1} / 1106.5
Crystal size	0.15 mm × 0.1 mm × 0.02 mm
2Θ range for data collection	4.76° to 144.14°
Index ranges	$-9 \le h \le 9$, $-17 \le k \le 17$, $-22 \le l \le 22$
Reflections collected	52088
Independent reflections	7847 [R_{int} = 0.0429, R_{sigma} = 0.0318]
Data/restraints/parameters	7847 / 1 / 532
Goodness-of-fit on F^2	1.034
Final R indexes [I_0>2σ_{I0}]	R_1 = 0.0195, wR_2 = 0.0492
Final R indexes [all data]	R_1 = 0.0196, wR_2 = 0.0493
Largest diff. peak/hole	1.01/–1.10 e Å^{-3}
Flack parameter	0.091(7)
CCDC	2022743

Table S19. Atomic coordinates (x 10^4) and equivalent isotropic displacement parameters (Å^2 x 10^3) for [Pt(dpyTSCmB)(CN)]. U(eq) is defined as one third of the trace of the orthogonalised U_{ij} tensor.

**Atom**	***x***	***y***	***z***	**U(eq)**
Pt1	8115.5(2)	5244.01(3)	3497.38(7)	17.02(4)
Pt1b	1291.4(2)	4708.89(3)	1521.26(8)	17.41(4)
S1	8794.5(15)	6219.0(5)	4430.7(6)	22.9(2)
S1b	721.7(15)	3714.4(5)	585.3(6)	22.9(2)
N2	7467(5)	6410(2)	2951.1(18)	19.3(7)
N3	7723(5)	7278(2)	3244.0(18)	20.1(7)
N4	8597(5)	8050(2)	4278(2)	20.7(7)
N5	4686(5)	7094(2)	1492.4(19)	27.4(8)

N1	7237(4)	4677(2)	2531.8(17)	20.3(6)
N6	9356(6)	3406(2)	4266(2)	28.4(9)
N2b	2056(5)	3572(2)	2074.1(18)	19.6(7)
N3b	2061(5)	2713(2)	1763.9(18)	19.3(7)
N4b	1587(5)	1927(2)	706.7(19)	20.8(7)
N1b	2086(4)	5295(2)	2490.1(17)	20.3(6)
N5b	4803(5)	2957(2)	3550(2)	27.0(8)
N6b	168(6)	6552(2)	735(2)	27.5(9)
C10	6815(5)	6302(3)	2285(2)	18.3(8)
C8	8313(5)	7238(3)	3927(2)	19.9(8)
C1	8780(6)	8933(2)	3881(2)	22.5(8)
C9	8409(6)	9747(3)	4378(2)	28.9(9)
C2	10553(6)	8987(2)	3580(2)	24.4(9)
C7	10674(7)	9111(3)	2843(2)	32.7(10)
C6	12305(8)	9115(3)	2554(3)	40.4(12)
C5	13815(7)	8995(3)	2987(3)	38.9(12)
C4	13715(7)	8893(3)	3727(3)	36.2(12)
C3	12097(6)	8883(3)	4014(3)	26.6(9)
C16	6331(6)	7106(3)	1802(2)	20.0(8)
C20	4256(7)	7805(3)	1041(2)	33.3(11)
C19	5377(8)	8502(3)	889(3)	35.5(12)
C18	7084(8)	8496(3)	1199(3)	32.8(11)
C17	7569(6)	7778(3)	1676(2)	27.3(9)
C11	6617(5)	5328(3)	2041(2)	20.0(8)
C15	7128(6)	3771(3)	2353(2)	23.2(9)
C14	6398(7)	3479(3)	1689(3)	29.5(10)
C13	5767(6)	4130(3)	1197(2)	27.9(10)
C12	5857(6)	5070(3)	1377(2)	24.2(9)
C21	8852(6)	4074(3)	3986(2)	24.3(9)
C10b	2633(6)	3678(3)	2748(2)	19.1(8)
C8b	1521(5)	2726(3)	1071(2)	18.7(8)
C1b	2268(5)	1072(2)	1058(2)	20.6(8)
C9b	1711(6)	238(3)	588(2)	25.7(8)
C2b	4246(5)	1108(2)	1218(2)	19.6(8)
C7b	4946(6)	969(3)	1924(2)	23.2(8)
C6b	6744(7)	971(3)	2079(3)	34.6(11)
C5b	7854(6)	1119(3)	1543(3)	40.8(12)
C4b	7185(7)	1265(3)	839(3)	34.8(11)
C3b	5383(6)	1256(3)	678(2)	25.9(9)
C11b	2686(5)	4654(3)	2994(2)	21.4(8)
C15b	2120(6)	6202(3)	2660(2)	25.9(9)
C14b	2746(7)	6518(3)	3326(3)	29.6(10)
C13b	3361(7)	5875(3)	3846(3)	31.2(10)
C12b	3329(6)	4938(3)	3677(3)	27.2(10)
C16b	3187(6)	2883(3)	3219(2)	21.9(9)
C17b	2071(6)	2134(3)	3299(2)	25.0(9)
C18b	2653(7)	1409(3)	3739(2)	27.9(10)
C19b	4341(7)	1451(3)	4072(3)	32.8(11)
C20b	5346(7)	2233(3)	3972(3)	35.0(11)
C21b	564(6)	5870(3)	1018(2)	25.1(9)

Table S20. Selected bond lengths [Å] and angles [°] for [Pt(dpyTSCmB)(CN)].

Pt1–S1	2.2614(9)	C1-C2	1.506(6)	N1b-Pt1b-S1b	164.80(9)	C18-C17-C16	118.2(5)
Pt1-N2	2.003(3)	C2-C7	1.394(6)	N1b-Pt1b-N2b	79.98(13)	C10-C11-N1	115.0(3)
Pt1-N1	2.040(3)	C2-C3	1.385(6)	C21b-Pt1b-S1b	97.86(12)	C12-C11-N1	121.1(4)

Pt1-C21	1.973(4)	C7-C6	1.393(8)	C21b-Pt1b-N2b	177.19(16)	C12-C11-C10	124.0(4)
Pt1b-S1b	2.2700(9)	C6-C5	1.366(8)	C21b-Pt1b-N1b	97.21(15)	C14-C15-N1	121.8(4)
Pt1b-N2b	1.995(3)	C5-C4	1.394(8)	C8-S1-Pt1	94.57(14)	C13-C14-C15	119.2(4)
Pt1b-N1b	2.041(3)	C4-C3	1.381(7)	C8b-S1b-Pt1b	94.25(13)	C12-C13-C14	119.5(4)
Pt1b-C21b	1.973(4)	C16-C17	1.383(6)	N3-N2-Pt1	122.5(3)	C13-C12-C11	119.2(4)
S1-C8	1.763(4)	C20-C19	1.362(7)	C10-N2-Pt1	116.3(3)	N6-C21-Pt1	177.0(4)
S1b-C8b	1.767(4)	C19-C18	1.385(8)	C10-N2-N3	121.2(3)	C11b-C10b-N2b	114.1(3)
N2-N3	1.369(4)	C18-C17	1.393(6)	C8-N3-N2	111.9(3)	C16b-C10b-N2b	122.6(3)
N2-C10	1.311(5)	C11-C12	1.377(6)	C1-N4-C8	121.3(3)	C16b-C10b-C11b	123.3(4)
N3-C8	1.318(6)	C15-C14	1.382(7)	C20-N5-C16	115.7(4)	N3b-C8b-S1b	125.5(3)
N4-C8	1.346(5)	C14-C13	1.372(7)	C11-N1-Pt1	112.9(3)	N4b-C8b-S1b	117.1(3)
N4-C1	1.479(5)	C13-C12	1.391(6)	C15-N1-Pt1	127.9(3)	N4b-C8b-N3b	117.4(3)
N5-C16	1.341(6)	C10b-C11b	1.475(5)	C15-N1-C11	119.1(4)	C9b-C1b-N4b	109.1(3)
N5-C20	1.348(5)	C10b-C16b	1.483(6)	N3b-N2b-Pt1b	122.2(2)	C2b-C1b-N4b	111.8(3)
N1-C11	1.367(5)	C1b-C9b	1.525(5)	C10b-N2b-Pt1b	117.6(3)	C2b-C1b-C9b	111.7(3)
N1-C15	1.345(5)	C1b-C2b	1.517(6)	C10b-N2b-N3b	120.1(3)	C7b-C2b-C1b	118.9(4)
N6-C21	1.145(5)	C2b-C7b	1.395(6)	C8b-N3b-N2b	112.9(3)	C3b-C2b-C1b	121.9(4)
N2b-N3b	1.363(4)	C2b-C3b	1.393(6)	C1b-N4b-C8b	121.2(3)	C3b-C2b-C7b	119.1(4)
N2b-C10b	1.306(5)	C7b-C6b	1.381(6)	C11b-N1b-Pt1b	112.9(3)	C6b-C7b-C2b	120.0(4)
N3b-C8b	1.324(5)	C6b-C5b	1.373(8)	C15b-N1b-Pt1b	127.5(3)	C5b-C6b-C7b	120.4(5)
N4b-C8b	1.337(5)	C5b-C4b	1.385(8)	C15b-N1b-C11b	119.5(4)	C4b-C5b-C6b	120.5(5)
N4b-C1b	1.469(5)	C4b-C3b	1.386(7)	C20b-N5b-C16b	115.9(4)	C3b-C4b-C5b	119.5(4)
N1b-C11b	1.368(5)	C11b-C12b	1.389(6)	C16-C10-N2	122.5(3)	C4b-C3b-C2b	120.5(4)
N1b-C15b	1.342(5)	C15b-C14b	1.372(7)	C11-C10-N2	115.2(3)	C10b-C11b-N1b	115.3(4)
N5b-C16b	1.341(6)	C14b-C13b	1.392(7)	C11-C10-C16	122.3(3)	C12b-C11b-N1b	120.4(4)
N5b-C20b	1.349(5)	C13b-C12b	1.383(6)	N3-C8-S1	126.2(3)	C12b-C11b-C10b	124.2(4)
N6b-C21b	1.142(5)	C16b-C17b	1.388(6)	N4-C8-S1	116.3(3)	C14b-C15b-N1b	122.3(4)
C10-C16	1.492(5)	C17b-C18b	1.378(6)	N4-C8-N3	117.5(3)	C13b-C14b-C15b	119.0(4)
C10-C11	1.475(5)	C18b-C19b	1.388(7)	C9-C1-N4	109.0(3)	C12b-C13b-C14b	119.3(4)
C1-C9	1.531(5)	C19b-C20b	1.382(7)	C2-C1-N4	110.6(3)	C13b-C12b-C11b	119.5(4)
				C2-C1-C9	113.4(3)	C10b-C16b-N5b	115.0(3)
N2-Pt1-S1	84.57(9)	C4-C3-C2	121.3(5)	C7-C2-C1	120.1(4)	C17b-C16b-N5b	124.3(4)
N1-Pt1-S1	164.86(10)	C10-C16-N5	115.4(4)	C3-C2-C1	121.7(4)	C17b-C16b-C10b	120.7(4)
N1-Pt1-N2	80.48(13)	C17-C16-N5	124.4(4)	C3-C2-C7	118.1(4)	C18b-C17b-C16b	118.5(4)
C21-Pt1-S1	97.62(12)	C17-C16-C10	120.1(4)	C6-C7-C2	120.5(5)	C19b-C18b-C17b	118.6(4)
C21-Pt1-N2	176.33(17)	C19-C20-N5	124.1(5)	C5-C6-C7	120.7(5)	C20b-C19b-C18b	118.7(4)
C21-Pt1-N1	97.44(15)	C18-C19-C20	119.6(4)	C4-C5-C6	119.3(5)	C19b-C20b-N5b	123.9(5)
N2b-Pt1b-S1b	84.95(9)	C17-C18-C19	117.8(4)	C3-C4-C5	120.0(5)	N6b-C21b-Pt1b	178.5(4)

Table S21. Anisotropic displacement parameters ($Å^2 \times 10^3$) for [Pt(dpyTSCmB)(CN)]. The anisotropic displacement factor exponent takes the form: $-2\pi^2[h^2a^{*2}U_{11}+2hka^*b^*U_{12}+\ldots]$.

Atom	U_{11}	U_{22}	U_{33}	U_{12}	U_{13}	U_{23}
Pt1	19.89(8)	15.40(8)	15.43(8)	-0.62(6)	-0.91(5)	1.02(6)
Pt1b	20.56(8)	14.47(8)	16.99(8)	2.12(6)	-0.03(5)	0.06(6)
S1	31.2(6)	20.1(5)	16.2(5)	0.1(4)	-5.3(4)	0.6(4)
S1b	30.4(6)	18.8(5)	18.5(5)	4.9(4)	-4.9(4)	-1.3(4)
N2	21.1(17)	18.6(17)	17.8(16)	-1.1(14)	-1.0(13)	-1.1(13)
N3	23.7(17)	14.4(16)	21.7(17)	-0.0(13)	-0.8(13)	-1.7(13)
N4	26.8(18)	16.8(17)	17.8(18)	-3.5(14)	-3.5(15)	-1.0(14)
N5	31(2)	27.8(19)	22.6(18)	4.3(16)	-5.8(15)	-0.1(15)
N1	21.3(15)	16.8(15)	22.7(16)	-3.6(15)	1.7(12)	-1.7(16)
N6	37(2)	23(2)	24(2)	4.2(17)	-3.3(17)	4.4(16)
N2b	24.1(18)	16.9(17)	17.3(16)	2.0(14)	-1.9(13)	-1.7(13)
N3b	23.5(17)	14.7(16)	19.4(16)	2.0(13)	-0.5(13)	-3.0(13)
N4b	23.8(17)	19.7(17)	18.1(17)	2.1(14)	-4.5(13)	-2.8(13)

N1b	20.5(15)	19.5(16)	21.1(15)	-3.5(16)	1.8(12)	-1.0(16)
N5b	30(2)	23.6(18)	26.5(19)	-3.3(16)	-5.4(15)	6.7(15)
N6b	40(2)	18.8(19)	22.3(19)	6.2(17)	-4.0(17)	4.8(15)
C10	19.8(19)	20(2)	14.3(18)	1.1(16)	-1.1(15)	1.4(16)
C8	16.5(19)	21(2)	23(2)	-0.3(16)	4.0(15)	-0.6(16)
C1	30(2)	14.1(18)	22.4(19)	-0.6(16)	-7.0(16)	0.5(15)
C9	29(2)	17.5(19)	39(2)	3(2)	-4.9(17)	-7(2)
C2	34(2)	10.6(17)	28(2)	-2.2(16)	-3.3(17)	-1.5(15)
C7	42(3)	26(2)	29(2)	-4(2)	-3.7(19)	3.1(18)
C6	52(3)	36(3)	34(3)	-6(3)	11(2)	4(2)
C5	36(3)	33(3)	50(3)	-7(2)	15(2)	-1(2)
C4	32(3)	22(2)	55(3)	-5(2)	3(2)	-5(2)
C3	30(2)	18(2)	31(2)	-2.6(17)	-1.9(18)	-0.7(17)
C16	28(2)	20(2)	11.3(19)	0.9(17)	-2.3(16)	-2.3(15)
C20	39(3)	36(3)	23(2)	13(2)	-8.6(19)	1.3(19)
C19	54(3)	31(3)	22(2)	17(2)	3(2)	3.9(19)
C18	45(3)	23(2)	31(3)	2(2)	13(2)	7.6(19)
C17	31(2)	26(2)	24(2)	0.4(19)	0.4(18)	-0.6(17)
C11	19.8(18)	21(2)	19.5(19)	-2.4(17)	1.2(14)	-0.7(17)
C15	27(2)	19(2)	25(2)	-2.4(17)	7.4(17)	-0.5(17)
C14	36(3)	24(2)	29(2)	-8.5(19)	7(2)	-4.7(19)
C13	33(2)	30(2)	21(2)	-6(2)	1.5(17)	-8.5(18)
C12	30(2)	21(2)	21.4(19)	-2.0(17)	-0.0(17)	0.2(16)
C21	27(2)	24(2)	21(2)	-1.1(18)	-4.1(17)	-3.6(18)
C10b	21(2)	16.9(19)	19(2)	-0.7(16)	0.8(15)	-0.6(16)
C8b	16.9(19)	17.7(19)	22(2)	1.9(15)	2.4(15)	1.5(15)
C1b	20.9(19)	18.2(19)	22.4(19)	2.4(15)	1.1(15)	-1.1(15)
C9b	28(2)	18.3(19)	30(2)	0(2)	-2.7(16)	-3(2)
C2b	20.9(19)	13.4(17)	24.1(19)	1.8(15)	-0.3(16)	-4.0(14)
C7b	24(2)	21(2)	25(2)	-1.8(16)	3.6(16)	1.5(16)
C6b	25(2)	34(3)	43(3)	2(2)	-9(2)	6(2)
C5b	16(2)	44(3)	62(4)	-1(2)	0(2)	-6(3)
C4b	28(2)	33(3)	45(3)	-5(2)	15(2)	-4(2)
C3b	30(2)	21(2)	28(2)	-1.8(18)	6.5(18)	-3.6(17)
C11b	26(2)	15.3(18)	23(2)	0.0(18)	3.8(15)	0.6(17)
C15b	31(2)	23(2)	24(2)	4.0(18)	5.0(18)	2.0(17)
C14b	40(3)	20(2)	29(2)	-3.4(19)	3(2)	-5.4(19)
C13b	41(3)	27(2)	25(2)	-7(2)	-3.4(19)	-8.7(18)
C12b	34(2)	28(3)	18.6(19)	-1.0(17)	-4.6(17)	-0.5(17)
C16b	28(2)	21(2)	17(2)	-1.2(17)	1.4(16)	-0.9(16)
C17b	26(2)	24(2)	25(2)	-0.3(17)	2.2(17)	1.1(17)
C18b	36(3)	25(2)	23(2)	-3.1(19)	4.7(19)	3.5(17)
C19b	43(3)	24(2)	31(3)	2(2)	-1(2)	9(2)
C20b	35(3)	38(3)	29(2)	3(2)	-12(2)	8(2)
C21b	27(2)	26(2)	22(2)	3.8(19)	-1.0(17)	-6.7(18)

Table S22. Selected NMR data of Pt TSC complexes [a]

	[Pt(fpyTSC-R)Cl]		[Pt(apyTSC-R)Cl]		[Pt(dpyTSC-R)Cl]		[Pt(dpyTSC-R)(CN)]
R	mB	Lp	mB	Lp	mB	Lp	mB
Py3	7.25 (dd)	8.30 (d)	7.68 (d)	7.78 (d)	7.45 (d)	7.85 (d)	7.44 (d)
Py4	8.13 (t)	8.15 (t)	8.16 (t)	7.90 (t)	8.06 (t)	8.08 (td)	8.10 (t)
Py5	7.70 (t)	7.71 (t)	7.74 (d)	7.69 (t)	7.70 (t)	7.53 (m)	7.59 (t)
Py6	8.70 (d)	8.72 (d)	8.76 (d)	8.88 (d)	8.84 (d)	8.90 (d)	8.85 (d)
	$^3J_{PtH}$ = 27 Hz	$^3J_{PtH}$ =24 Hz	$^3J_{PtH}$ =16 Hz	$^3J_{PtH}$ = 14 Hz	$^3J_{PtH}$ = 27 Hz	$^3J_{PtH}$ = 31 Hz	$^3J_{PtH}$ = 35 Hz

H_{im}	8.35 (s)	8.40 (s)					
	$^3J_{PtH}$ = 12 Hz	$^3J_{PtH}$ = 12 Hz					
CH_{3im}			2.26 (s)	2.30 (s)			
Py'3					7.36 (d)	7.43 (d)	7.49 (d)
Py'4					7.97 (t)	7.98 (td)	7.99 (t)
Py'5					7.60 (dd)	7.73 (td)	7.66 (t)
Py'6					8.72 (d)	8.75 (d)	8.77 (d)
*N*H7	8.87 (d)	7.08 (d)	8.76 (d)	7.07 (d)	9.02 (d)	7.06 (d)	9.19 (d)

[a] Chemical shifts in ppm, nomenclature:

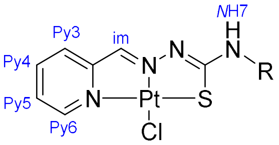

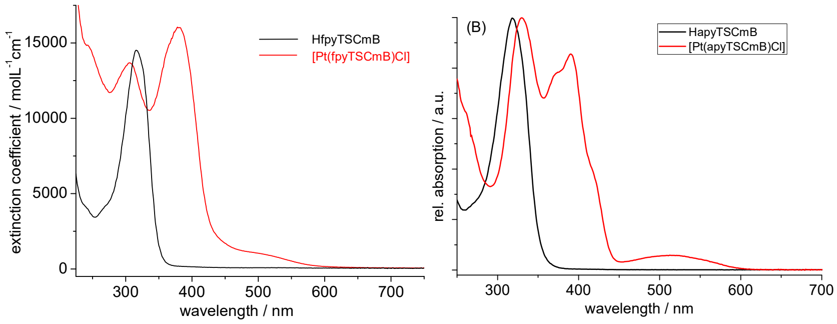

Figure S13. UV-vis absorption spectra of (A) HfpyTSCmB (black) and [Pt(fpyTSCmB)Cl] (red), (B) HapyTSCmB (black) and [Pt(apyTSCmB)Cl] (red) in THF.

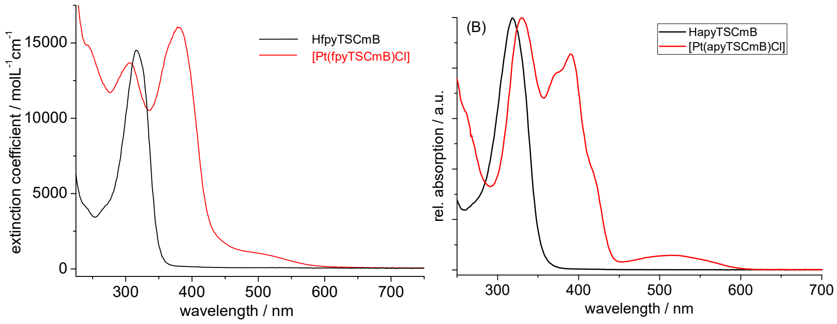

Figure S14. UV-vis absorption spectra of (A) HfpyTSCmB (black) and [Pt(fpyTSCmB)Cl] (red), (B) HapyTSCmB (black) and [Pt(apyTSCmB)Cl] (red) in THF.

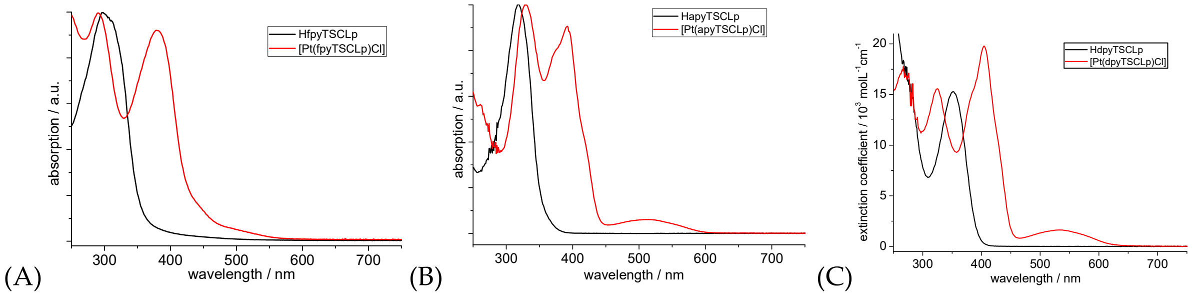

Figure S15. UV-vis absorption spectra of (A) HfpyTSCLp (black) and [Pt(fpyTSCLp)Cl] (red), (B) HapyTSCLp (black) and [Pt(apyTSCLp)Cl] (red) and (C) HdpyTSCLp (black) and [Pt(dpyTSCLp)Cl] (red) in THF.

Table S23. UV-vis absorption data of selected TSC protoligands and Pt complexes.[a]

λ_n							

protoligand	HfpyTSCmB	HfpyTSCLp	HapyTSCmB	HapyTSCLp	HdpyTSCmB	HdpyTSCLp	
1	316 [14505]	299 [15928]	319 [25604]	318 [25610]	275 [13171]	272 [17444]	275 [13171]
2					339 [17809]	351 [15498]	339 [17809]
complexes	[Pt(fpyTSCmB)Cl]	[Pt(fpyTSCLp)Cl]	[Pt(apyTSCmB)Cl]	[Pt(apyTSCLp)Cl]	[Pt(dpyTSCmB)Cl]	[Pt(dpyTSCLp)Cl]	[Pt(dpyTSCmB)(CN)]
1	244 [14892]	247 [13518]	330 [18465]	329 [18347]	277 [24199]	268 [17685]	316 [13880]
2	307 [13720]	290 [13451]	372 [12423]	372 [12847]	391 [20223]	324 [15802]	294 [15143]
3	380 [16064]	379 [12463]	391 [16342]	391 [16366]	526 [1492]	403 [19747]	380 [21017]
4	511 [983]	512 [676]	418 [7743]	419 [7695]		533 [1624]	399 [24091]
5			515 [1463]	515 [1440]			491 [1477]

[a] Measured in MeCN, wavelengths λ in nm, extinction coefficients in brackets [L/(mol·cm)].

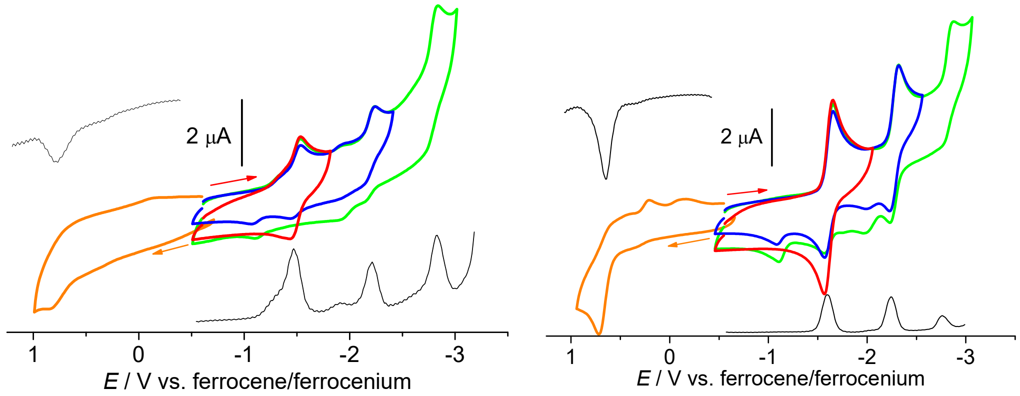

Figure S16. Cyclic voltammograms of [Pt(fpyTSCmB)Cl] (left) [Pt(apyTSCmB)Cl] (right) in 0.1 M *n*-Bu$_4$NPF$_6$/MeCN solution at 100 mV/s scan rate. Square wave measurements in black.

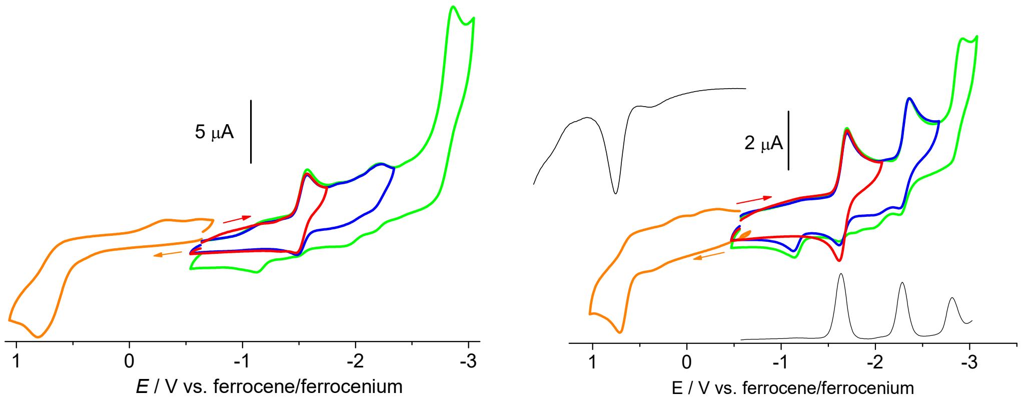

Figure S17. Cyclic voltammograms of [Pt(fpyTSCLp)Cl] (left) and [Pt(apyTSCLp)Cl] (right) in 0.1 M *n*-Bu$_4$NPF$_6$/MeCN solution at 100 mV/s scan rate. Square wave measurements in black.

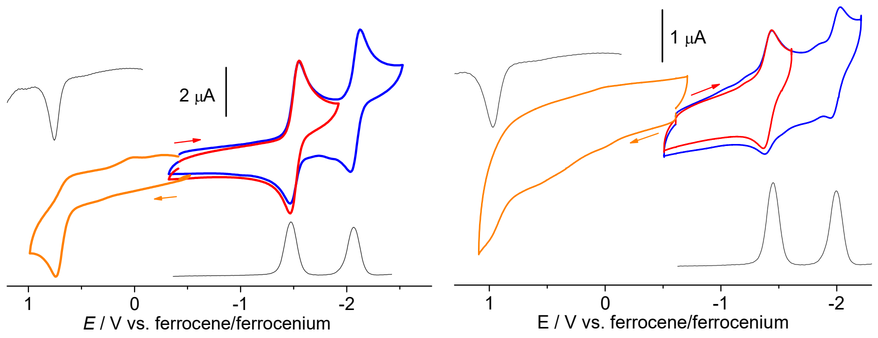

Figure S18. Cyclic voltammograms of [Pt(dpyTSCLp)Cl] (left) and [Pt(dpyTSCmB)(CN)] (right) in 0.1 M *n*-Bu$_4$NPF$_6$/MeCN solution at 100 mV/s scan rate. Square wave measurements in black.

Table S24. Electrochemical data for the TSC Pt complexes [a]

[Pt(TSC)Cl]	*Epa* Ox	$E_{1/2}$ Red1	$E_{1/2}$ Red2	*Epc* Red3	*ΔE* (Red1-Red2)
TSC =					
fpyTSCmB	0.82	–1.50	–2.19 irr	–2.83	0.69
fpyTSCLp	0.81	–1.50	–2.20 irr	–2.86	0.70
apyTSCmB	0.71	–1.60	–2.25	–2.90	0.65
apyTSCLp	0.72	–1.65	–2.30	–2.92	0.65
dpyTSCmB	0.74	–1.51	–2.08	–2.90	0.57
dpyTSCLp	0.74	–1.50	–2.10	-	0.60
[Pt(dpyTSCmB)(CN)]	0.97	–1.41	–1.99	-	0.58

[a] Measured in 0.1 M *n*-Bu_4NPF_6/MeCN at 100 mV/s. Potentials *E* in V vs. ferrocene/ferocenium. Half-wave potentials $E_{1/2}$ for reversible waves, *Epa* = anodic peak potential and *Epc* = cathodic peak potential for irreversible waves; scan rate = 100 mV/s.

LUMO+2:

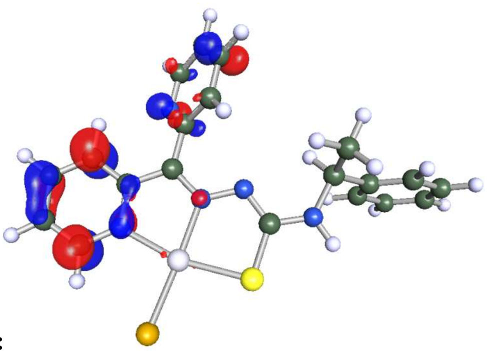

LUMO+1

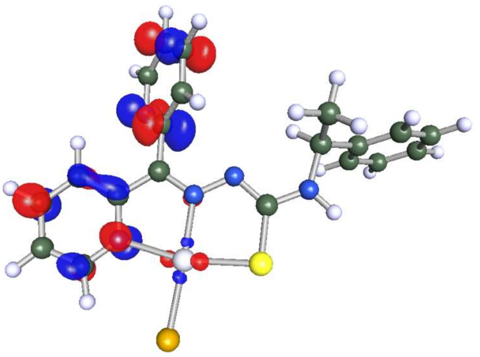

HOMO-1:

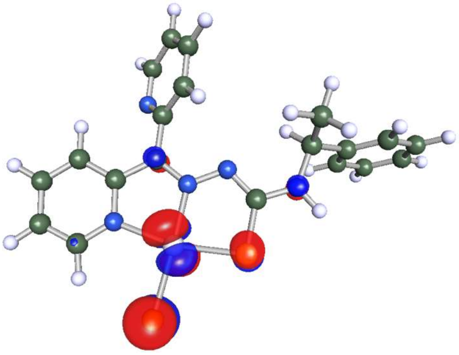

HOMO-2:

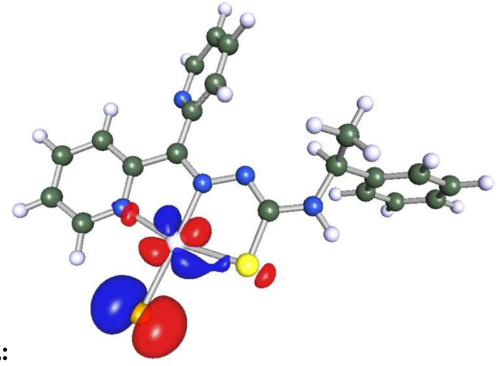

Figure S19. DFT-calculated composition of LUMO+2, LUMO+1, HOMO-1 and HOMO-2 for [Pt(dpyTSCmB)Cl] at B3LYP def2-TZVP level (C,H,N,S,Cl) and B3LYP LANL2DZ level with ecp60 Hay & Wadt (Pt). Iso-surface level at 0.06.

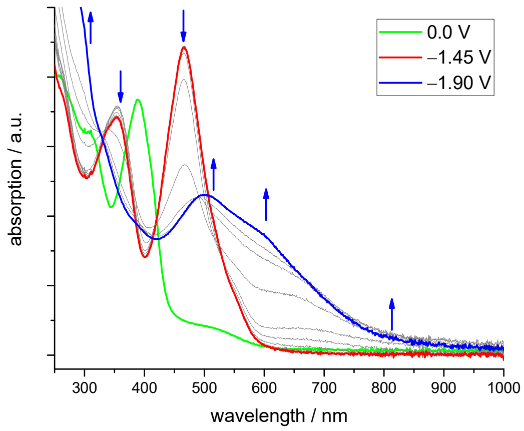

Figure S20. UV-vis absorption spectra recorded during the second reduction of [Pt(dpyTSCmB)Cl] in 0.1 M *n*-Bu_4NPF_6/MeCN solution (0.05 V increments).

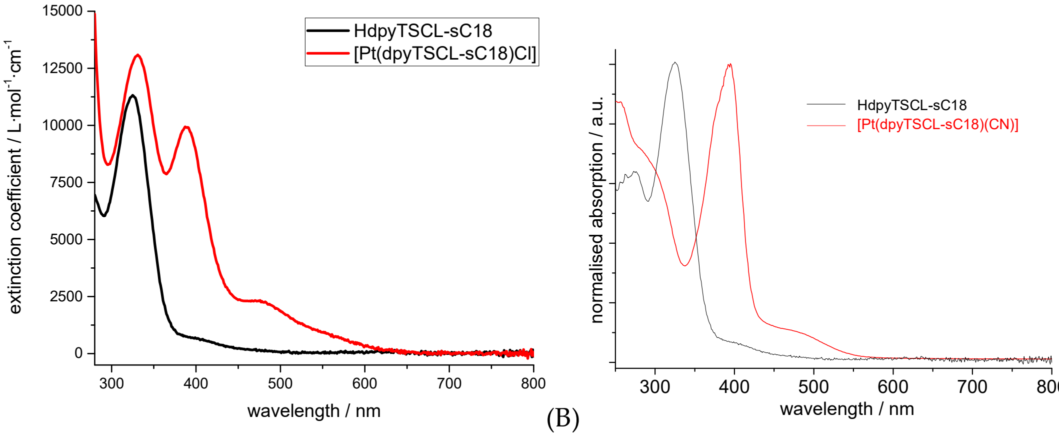

(A) (B)

Figure S21. UV-vis absorption spectra of the peptide conjugates (A) HdpyTSCL-sC18 (black), [Pt(dpyTSCL-sC18)Cl] (red) and (B) HdpyTSCL-sC18 (black), [Pt(dpyTSCL-sC18)(CN)] (red) in demineralised H_2O.

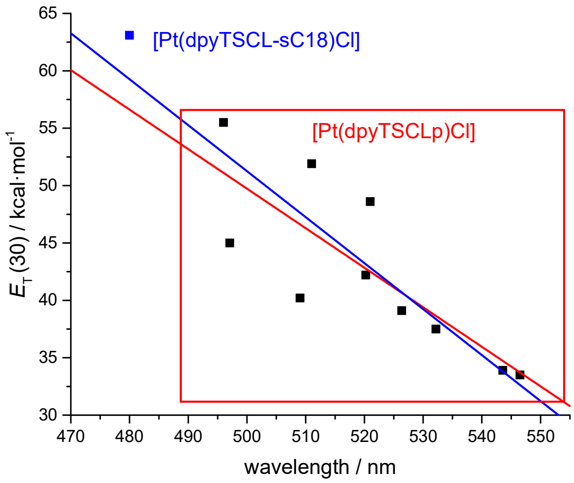

Figure S22. Correlation of the long-wavelength MLCT absorption maxima of [Pt(dpyTSCLp)Cl] and [Pt(dpyTSCL-sC18)Cl] with the Reichhardt E_T(30) values for the solvent polarity.[4] Linear fits for [Pt(dpyTSCLp)Cl] (red) and including the conjugate [Pt(dpyTSCL-sC18)Cl] (blue).

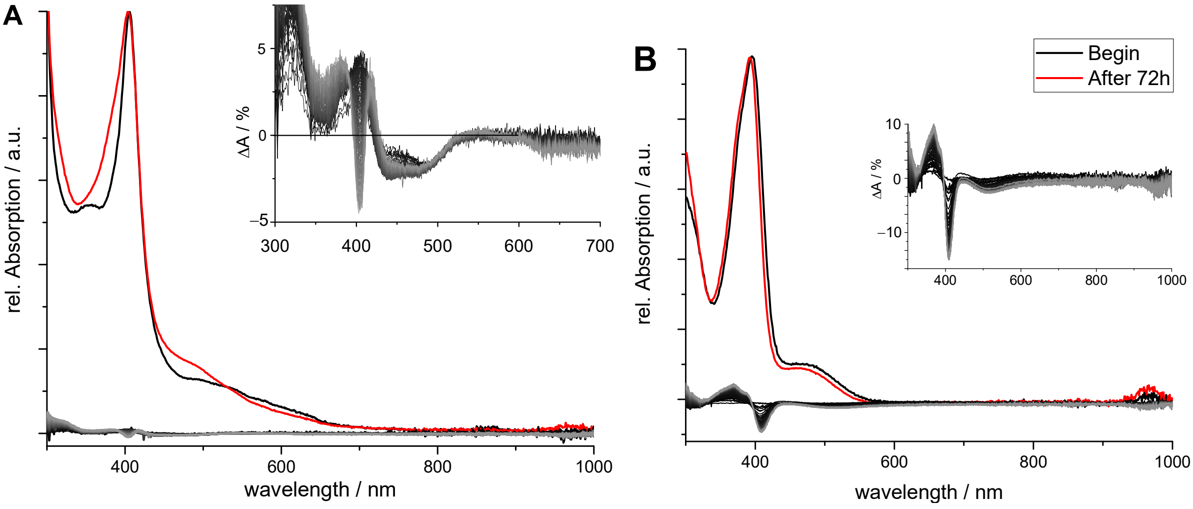

Figure S23. UV-vis absorption spectra of FBS solution (red) and [Pt(dpyTSCL-sC18)Cl] (left) and [Pt(dpyTSCL-sC18)(CN)] (right) in FBS (green trace left). The insert shows the difference spectra for 72 h measured every 30 min (black to grey).

----

3. Cell viability assay

MCF-7, HT-29 and HEK-293 cells were seeded onto 96-well plates (Sarstedt, 10000, 12500 cells and 8500 cells per well, respectively). After reaching 50-60 % confluency, medium was removed and cells were incubated with peptides at different concentrations (1-25 µM in serum supplemented medium) for 72 h at 37 °C. Cells treated with 70 % EtOH for 10 min served as a positive control. After washing with serum supplemented medium, cells were incubated with 10 % (v/v) resazurin (Sigma-Aldrich) in serum supplemented medium for 1.5 h or 3 h at 37 °C, respectively. Finally, cell viability of treated cells was determined relative to that of untreated cells by measurement of the resorufin product at 595 nm (λ_{ex} = 550 nm) on a Tecan infinite M200 plate reader (Tecan Group AG).

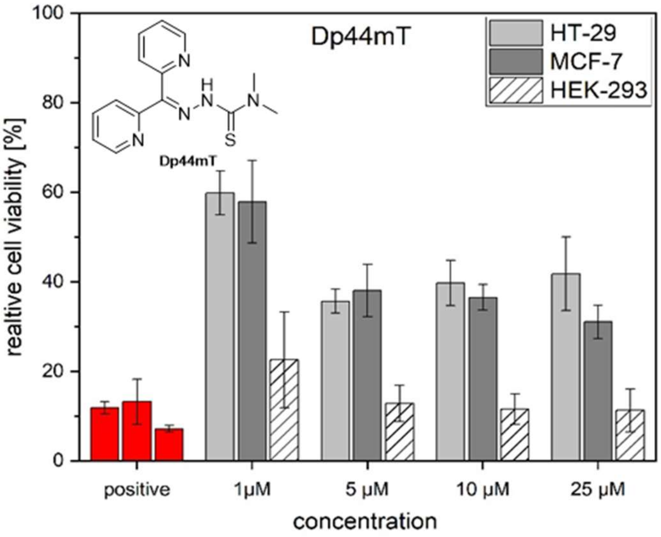

Figure S24. Anti-proliferative activity of Dp44mT (di-2-pyridylketone-4,4,-dimethyl-3-thiosemicarbazone) at different concentrations against MCF-7, HT-29 and HEK-293 cells after incubation for 72 h. Cells treated with 70 % EtOH served as a positive control. Data were normalised to untreated cells (100 % viability). Shown are mean ± SD values of two independent experiments, each performed in triplicate.

References

1. R. Wong and S. J. Dolman, Isothiocyanates from Tosyl Chloride Mediated Decomposition of in Situ Generated Dithiocarbamic Acid Salts, *J. Org. Chem.*, 2007, **72**, 3969–3971.
2. D. Taşdemir, A. Karaküçük-İyidoğan, M. Ulaşli, T. Taşkin-Tok, E. E. Oruç-Emre and H. Bayram, Synthesis, Molecular Modeling, and Biological Evaluation of Novel Chiral Thiosemicarbazone Derivatives as Potent Anticancer Agents, *Chirality*, 2015, **27**, 177–188.
3. M. Horn and I. Neundorf, Design of a novel cell-permeable chimeric peptide to promote wound healing, *Sci. Rep.* 2018, **8**, 16279–16279.
4. C. Reichardt, Solvatochromic Dyes as Solvent Polarity Indicators, *Chem. Rev.* **1994**, *94*, 2319–2358.

4. NMR spectra

TSCmB Series **pages 29-35**

precursors:

Fig. X1. ^{1}H NMR spectrum of ITCmB (isocyanate) in DMSO-d_6 at 300 MHz.
Fig. X2. ^{13}C APT NMR spectrum of ITCmB in DMSO-d_6 at 75 MHz.
Fig. X3. ^{13}C DEPTQ NMR spectrum of ITCmB in DMSO-d_6 at 75 MHz.
Fig. X4. ^{1}H, ^{13}C HSQC NMR spectrum of ITCmB in DMSO-d_6.
Fig. X5. ^{1}H, ^{13}C HMBC NMR spectrum of ITCmB in DMSO-d_6.
Fig. X6 ^{1}H, ^{1}H COSY NMR spectrum of ITCmB in DMSO-d_6.
Fig. X7. ^{1}H NMR spectrum of TSCmB (thiosemicarbazide) in DMSO-d_6 at 300 MHz.
Fig. X8. ^{13}C DEPTQ NMR spectrum of TSCmB in DMSO-d_6 at 75 MHz.
Fig. X9. ^{13}C APT NMR spectrum of TSCmB in DMSO-d_6 at 75 MHz.

protoligands: **pages 35-42**

Fig. X10. ^{1}H, ^{13}C HSQC NMR spectrum of TSCmB in DMSO-d_6.
Fig. X11. ^{1}H, ^{13}C HMBC NMR spectrum of TSCmB in DMSO-d_6.
Fig. X12. ^{1}H, ^{1}H COSY NMR spectrum of TSCmB in DMSO-d_6.
Fig. X13 ^{1}H, ^{1}H TOCSY NMR spectrum of TSCmB in DMSO-d_6.
Fig. X14. ^{1}H NMR spectrum of HfpyTSCmB in $CDCl_3$ at 300 MHz.
Fig. X15. ^{13}C DEPTQ NMR spectrum of HfpyTSCmB in $CDCl_3$ at 75 MHz.
Fig. X16. ^{1}H,^{13}C HMBC NMR spectrum of HfpyTSCmB in $CDCl_3$.
Fig. X17. ^{1}H,^{13}C HSQC NMR spectrum of HfpyTSCmB in $CDCl_3$.
Fig. X18. ^{1}H,^{13}C COSY NMR spectrum of HfpyTSCmB in $CDCl_3$.
Fig. X19. ^{1}H spectrum of HfpyTSCmB in DMSO-d$_6$ at 300 MHz.
Fig. X20. ^{13}C DEPTQ NMR spectrum of HfpyTSCmB in DMSO-d$_6$ at 75 MHz.
Fig. X21. ^{1}H NMR spectrum of HapyTSCmB in DMSO-d_6 at 300 MHz.
Fig. X22. ^{13}C DEPTQ NMR spectrum of HapyTSCmB in DMSO-d_6 at 75 MHz.
Fig. X23. ^{1}H,^{13}C HSQC NMR spectrum of HapyTSCmB in DMSO-d_6.
Fig. X24. ^{1}H,^{13}C HMBC NMR spectrum of HapyTSCmB in DMSO-d_6.
Fig. X25. ^{1}H NMR spectrum of HdpyTSCmB in DMSO-d_6 at 300 MHz.
Fig. X26. ^{13}C APT NMR spectrum of HdpyTSCmB in DMSO-d_6 at 75 MHz.
Fig. X27. ^{1}H,^{13}C HSQC NMR spectrum of HdpyTSCmB in DMSO-d_6.
Fig. X28. ^{1}H,^{13}C HMBC NMR spectrum of HdpyTSCmB in DMSO-d_6.
Fig. X29. ^{1}H,^{1}H COSY NMR spectrum of HdpyTSCmB in DMSO-d_6.
Fig. X30. ^{1}H,^{1}H NOESY NMR spectrum of HdpyTSCmB in DMSO-d_6.
Fig. X31. ^{1}H,^{1}H TOCSY NMR spectrum of HdpyTSCmB in DMSO-d_6.

TSCLp Series

precursors: **pages 42-57**

Fig. X32. ^{1}H NMR spectrum of *N*^{6}-Cbz lysine in DMSO-d_6 at 300 MHz.
Fig. X33. ^{1}H NMR spectrum of *N*^{6}-Cbz *N*^{1}-Boc lysine in $CDCl_3$ at 300 MHz.
Fig. X34. ^{13}C DEPTQ NMR spectrum of *N*^{6}-Cbz *N*^{1}-Boc lysine in $CDCl_3$ at 75 MHz.
Fig. X35. ^{1}H,^{13}C HSQCed NMR spectrum of *N*^{6}-Cbz *N*^{1}-Boc lysine in $CDCl_3$.
Fig. X36. ^{1}H,^{13}C HMBC NMR spectrum of *N*^{6}-Cbz *N*^{1}-Boc lysine in $CDCl_3$.
Fig. X37. ^{1}H,^{1}H COSY NMR spectrum of *N*^{6}-Cbz *N*^{1}-Boc lysine in $CDCl_3$.
Fig. X38. ^{1}H NMR spectrum of *N*^{6}-Cbz *N*^{1}-Boc *C*-tBu lysine in $CDCl_3$ at 300 MHz.
Fig. X39. ^{13}C DEPTQ NMR spectrum of *N*^{6}-Cbz *N*^{1}-Boc *C*-tBu lysine in $CDCl_3$ at 75 MHz.
Fig. X40. ^{1}H,^{13}C HSQCed NMR spectrum of *N*^{6}-Cbz *N*^{1}-Boc *C*-tBu lysine in $CDCl_3$.
Fig. X41. ^{1}H,^{13}C HMBC NMR spectrum of *N*^{6}-Cbz *N*^{1}-Boc *C*-tBu lysine in $CDCl_3$.
Fig. X42. ^{1}H,^{1}H COSY NMR spectrum of *N*^{6}-Cbz *N*^{1}-Boc *C*-tBu lysine in $CDCl_3$.

Fig. X43. ^{1}H NMR spectrum of *N*1-Boc *C*-tBu lysine in $CDCl_3$ at 300 MHz.
Fig. X44. ^{13}C DEPTQ NMR spectrum of *N*1-Boc *C*-tBu lysine in CDCl at 75 MHz.
Fig. X45. $^{1}H,^{13}C$ HSQCed NMR spectrum of *N*1-Boc *C*-tBu lysine in $CDCl_3$.
Fig. X46. $^{1}H,^{13}C$ HMBC NMR spectrum of *N*1-Boc *C*-tBu lysine in $CDCl_3$.
Fig. X47. $^{1}H,^{1}H$ COSY NMR spectrum of *N*1-Boc *C*-tBu lysine in $CDCl_3$.
Fig. X48. ^{1}H NMR spectrum of ITCLp in $CDCl_3$ at 300 MHz.
Fig. X49. ^{13}C DEPTQ NMR spectrum of ITCLp in $CDCl_3$ at 75 MHz.
Fig. X50. $^{1}H,^{13}C$ HSQCed NMR spectrum of ITCLp in $CDCl_3$.
Fig. X51. $^{1}H,^{1}H$ COSY NMR spectrum of ITCLp in $CDCl_3$.
Fig. X52. ^{1}H NMR spectrum of TSCLp in $CDCl_3$ at 300 MHz.
Fig. X53. ^{13}C DEPTQ NMR spectrum of TSCLp in $CDCl_3$ at 75 MHz.
Fig. X54. $^{1}H,^{13}C$ HSQCed NMR spectrum of TSCLp in $CDCl_3$.
Fig. X55. $^{1}H,^{13}C$ HMBC NMR spectrum of TSCLp in $CDCl_3$.
Fig. X56. $^{1}H,^{1}H$ COSY NMR spectrum of TSCLp in $CDCl_3$.

protoligands: **pages 58-63**
Fig. X57. ^{1}H NMR spectrum of HfpyTSCLp in DMSO-d_6 at 300 MHz.
Fig. X58. ^{1}H NMR spectrum of HapyTSCLp in DMSO-d_6 at 300 MHz.
Fig. X59. ^{13}C DEPTQ NMR spectrum of HapyTSCLp in DMSO-d_6 at 75 MHz.
Fig. X60. $^{1}H,^{13}C$ HSQCed NMR spectrum of HapyTSCLp in DMSO-d_6.
Fig. X61. $^{1}H,^{1}H$ COSY NMR spectrum of HapyTSCLp in DMSO-d_6.
Fig. X62. ^{1}H NMR spectrum of HdpyTSCLp in DMSO-d_6 at 300 MHz.
Fig. X63. ^{13}C DEPTQ NMR spectrum of HdpyTSCLp in DMSO-d_6 at 75 MHz.
Fig. X64. $^{1}H,^{13}C$ HSQCed NMR spectrum of HdpyTSCLp in DMSO-d_6.
Fig. X65. $^{1}H,^{1}H$ COSY NMR spectrum of HdpyTSCLp in DMSO-d_6.
Fig. X66. ^{1}H NMR spectrum of HdpyTSCLBoc in DMSO-d_6 at 300 MHz.

[Pt(RpyTSCmB)X] Series **page 63-71**
Fig. X67. ^{1}H NMR spectrum of [Pt(fpyTSCmB)Cl] in DMSO-d_6 at 300 MHz.
Fig. S68.^{1}H NMR spectrum of [Pt(apyTSCmB)Cl] in DMSO-d_6 at 300 MHz.
Fig. X69. ^{1}H NMR spectrum of [Pt(dpyTSCmB)Cl] in DMSO-d_6 at 300 MHz.
Fig. X70. $^{1}H,^{1}H$ COSY NMR spectrum of [Pt(dpyTSCmB)Cl] in DMSO-d_6.
Fig. X71. $^{1}H,^{195}Pt$ HMBC NMR spectrum of [Pt(dpyTSCmB)Cl] in DMSO-d_6.
Fig. X72. ^{1}H NMR spectrum of [Pt(dpyTSCmB)(CN)] in DMSO-d_6 at 300 MHz.
Fig. X73. $^{1}H,^{13}C$ HSQC NMR spectrum of [Pt(dpyTSCmB)(CN)] in DMSO-d_6.
Fig. X74. $^{1}H,^{13}C$ HMBC NMR spectrum of [Pt(dpyTSCmB)(CN)] in DMSO-d_6.
Fig. X75. $^{1}H,^{1}H$ COSY NMR spectrum of [Pt(dpyTSCmB)(CN)] in DMSO-d_6.
Fig. X76. $^{1}H,^{195}Pt$ HMBC NMR spectrum of [Pt(dpyTSCmB)(CN)] in DMSO-d_6.
Fig. X77. ^{1}H NMR spectrum of [Pt(fpyTSCLp)Cl] in DMSO-d_6 at 300 MHz.
Fig. X78. ^{1}H NMR spectrum of [Pt(apyTSCLp)Cl] in DMSO-d_6 at 300 MHz.
Fig. X79. $^{1}H,^{13}C$ HSQCed NMR spectrum of [Pt(apyTSCLp)Cl] in DMSO-d_6.
Fig. X80. $^{1}H,^{13}C$ HMBC NMR spectrum of [Pt(apyTSCLp)Cl] in DMSO-d_6.
Fig. X81. $^{1}H,^{195}Pt$ HMBC NMR spectrum of [Pt(apyTSCLp)Cl] in DMSO-d_6.
Fig. X82. ^{1}H NMR spectrum of [Pt(dpyTSCLp)Cl] in DMSO-d_6 at 300 MHz.
TSCmB Series

precursors:

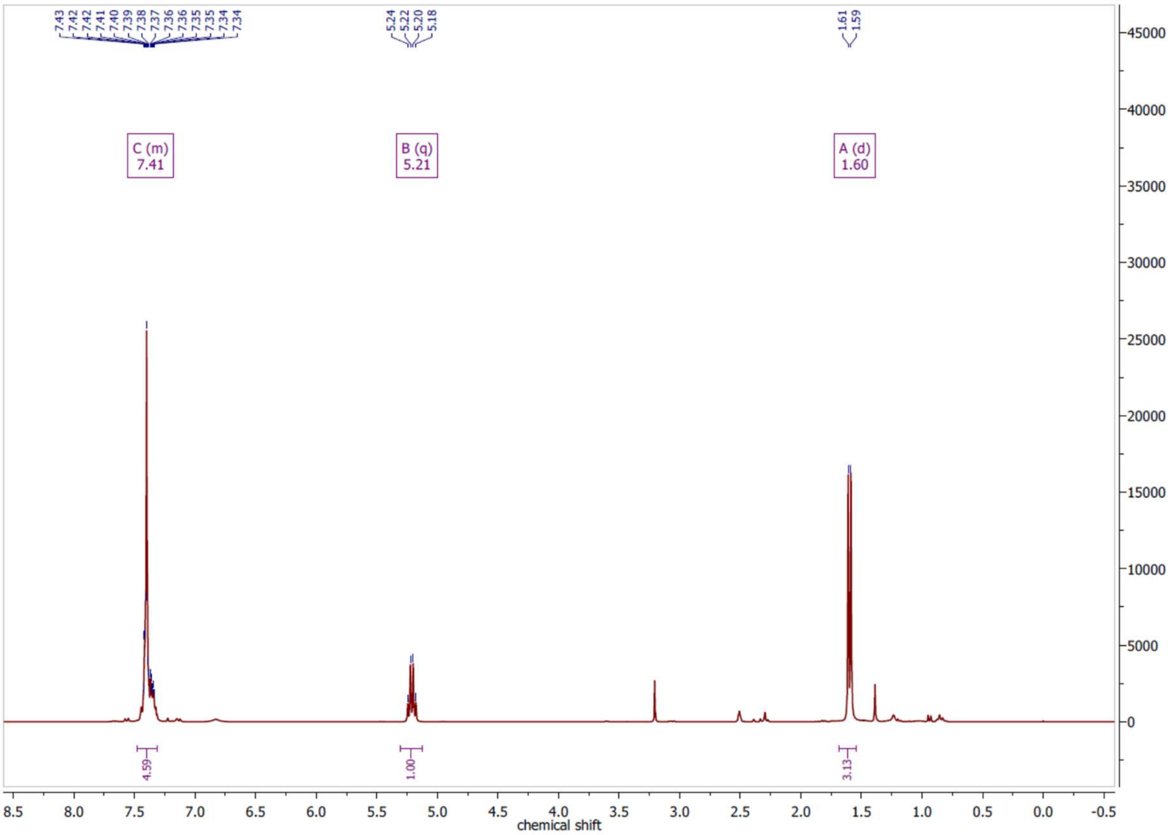

Fig. X1. ^{1}H NMR spectrum of ITCmB (isocyanate) in DMSO-d_6 at 300 MHz.

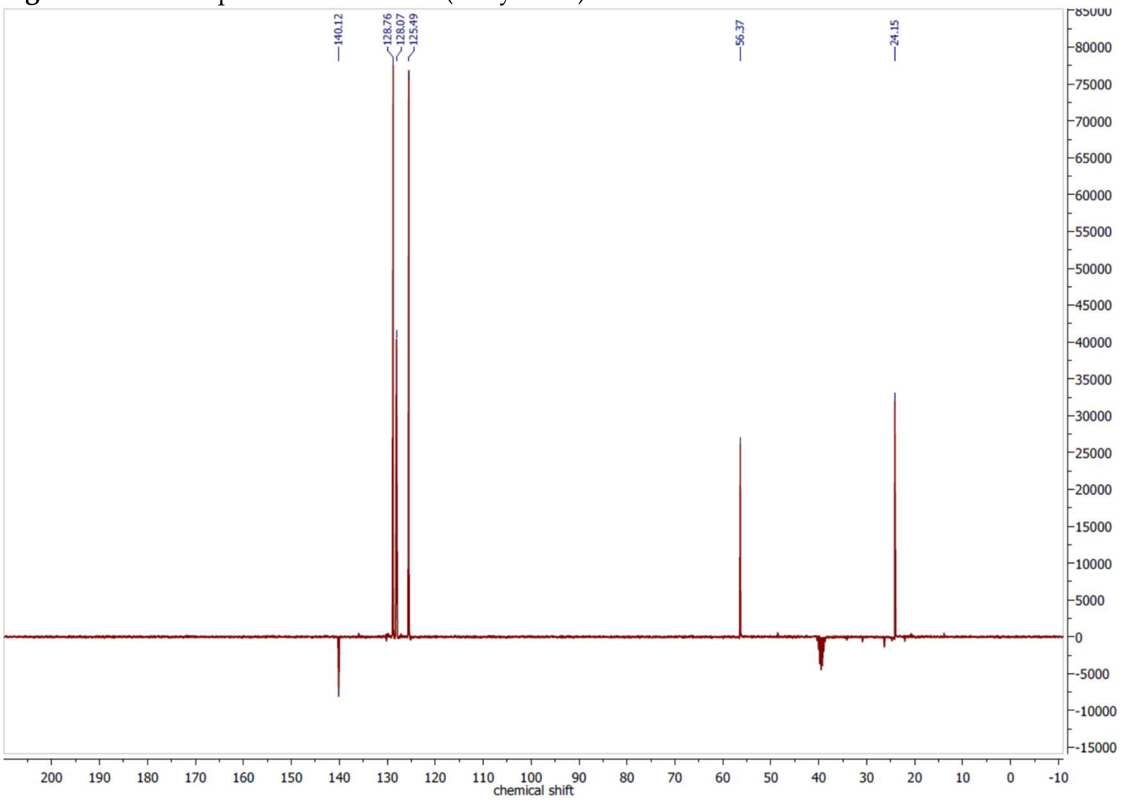

Fig. X2. ^{13}C APT NMR spectrum of ITCmB in DMSO-d_6 at 75 MHz.

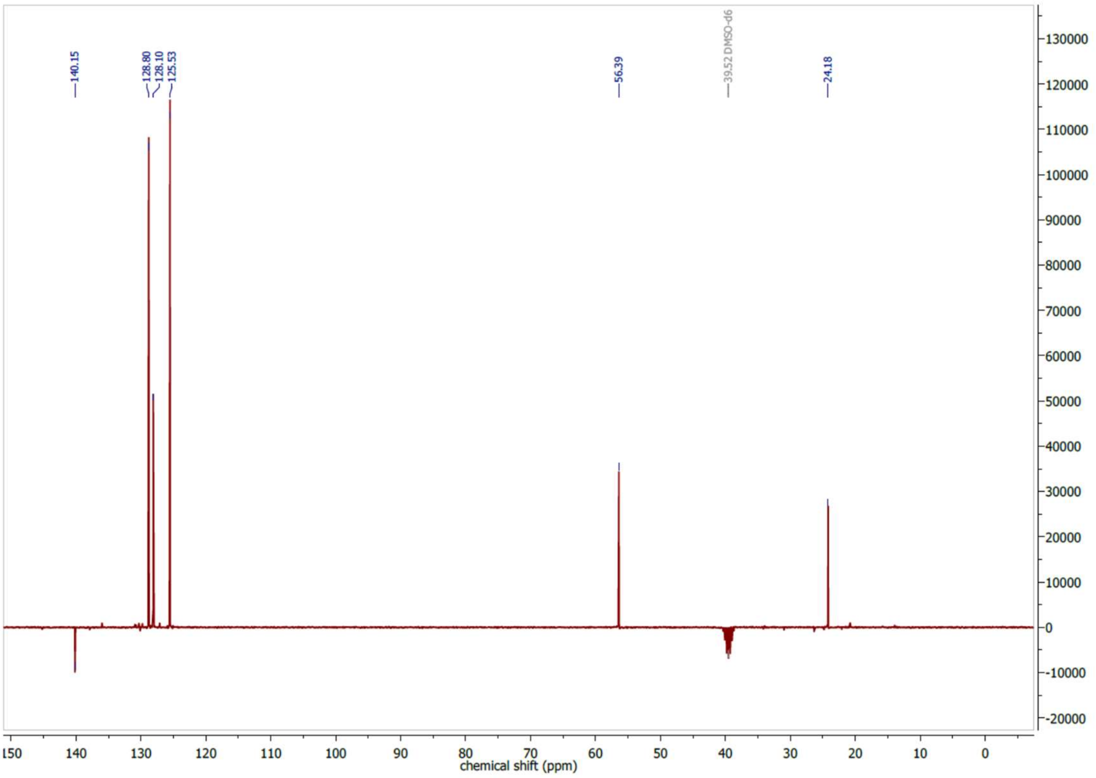

Fig. X3. ^{13}C DEPTQ NMR spectrum of ITCmB in DMSO-d_6 at 75 MHz.

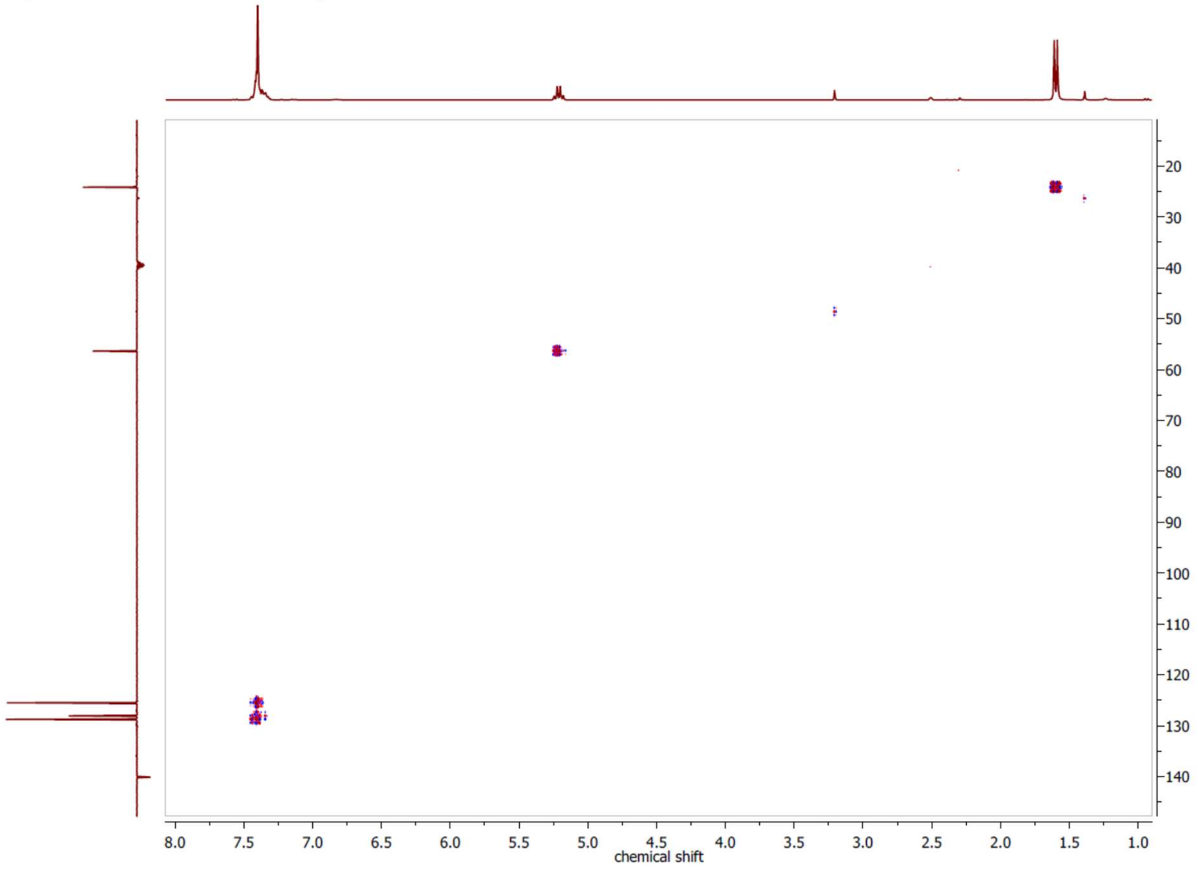

Fig. X4. ^{1}H, ^{13}C HSQC NMR spectrum of ITCmB in DMSO-d_6.

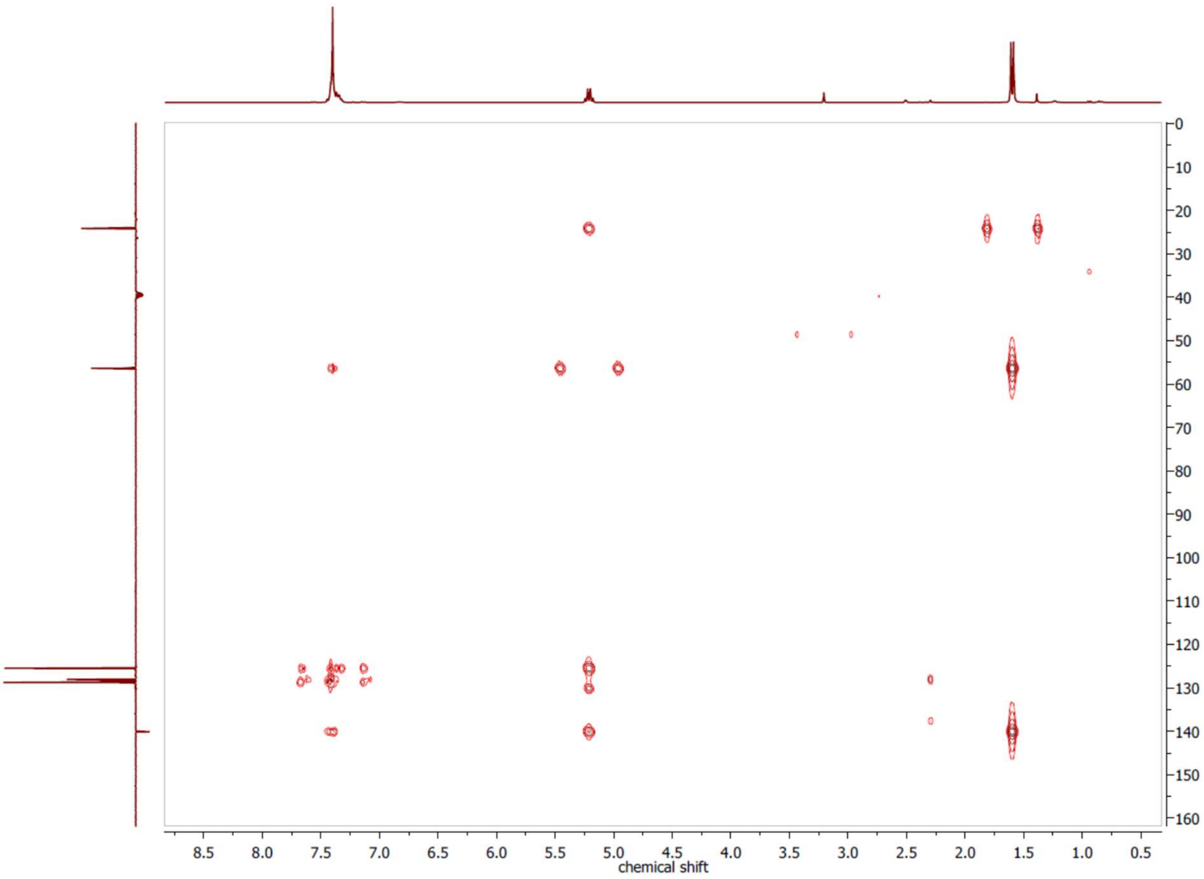

Fig. X5. ^{1}H, ^{13}C HMBC NMR spectrum of ITCmB in DMSO-*d*6.

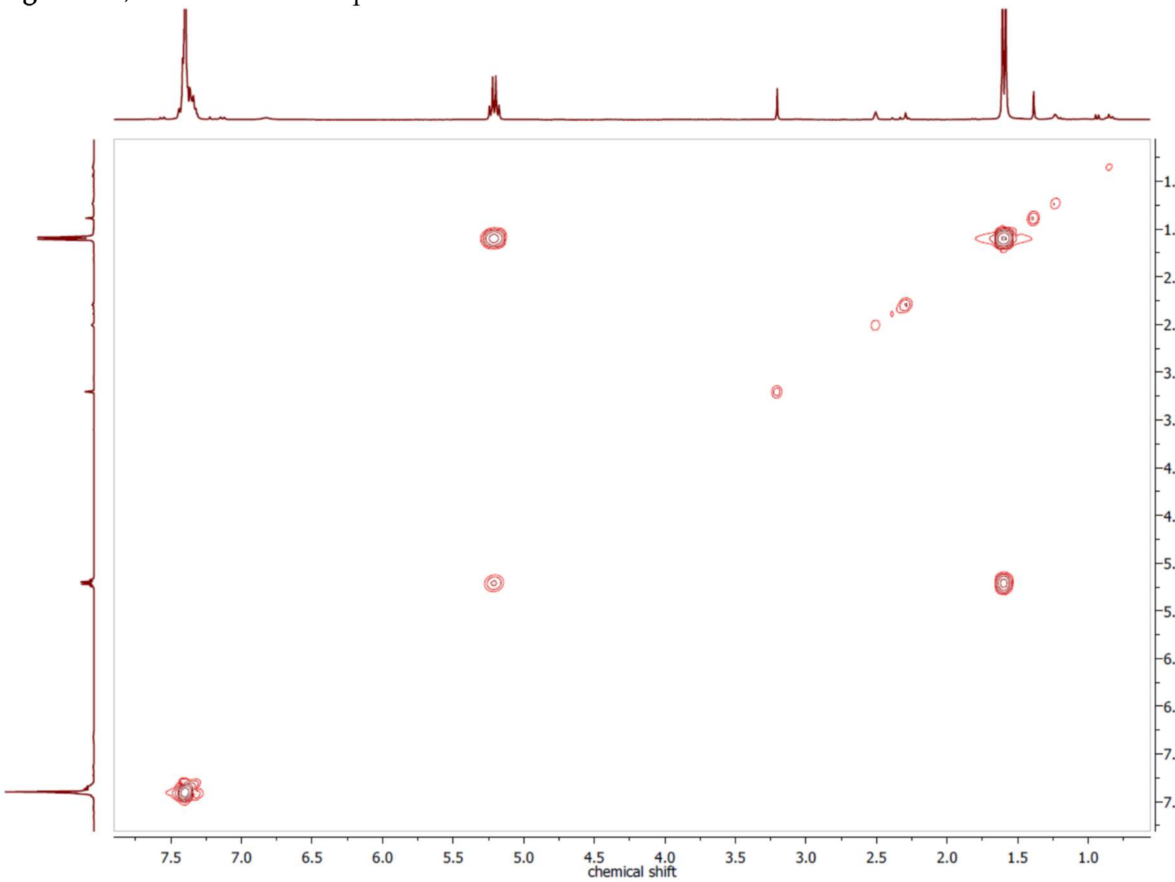

Fig. X6 ^{1}H, ^{1}H COSY NMR spectrum of ITCmB in DMSO-*d*6.

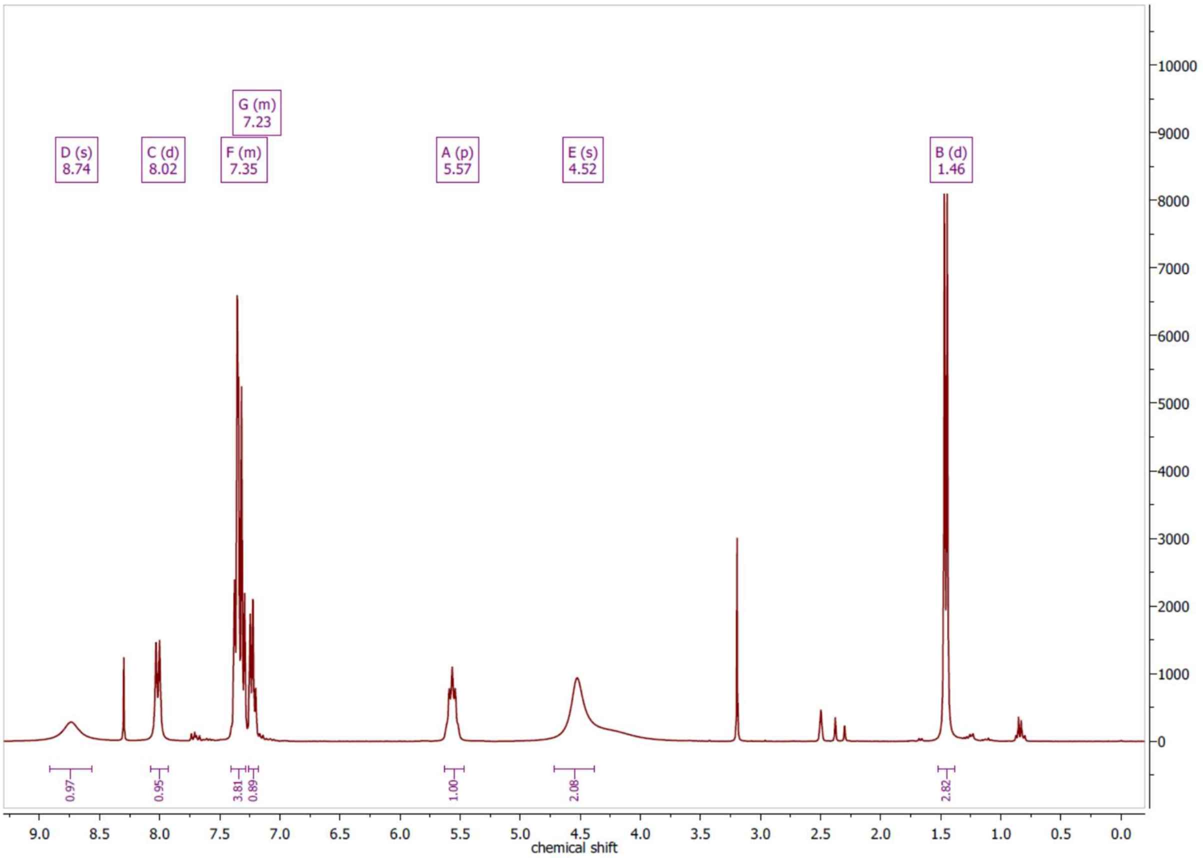

Fig. X7. ^{1}H NMR spectrum of TSCmB (thiosemicarbazide) in DMSO-d_6 at 300 MHz.

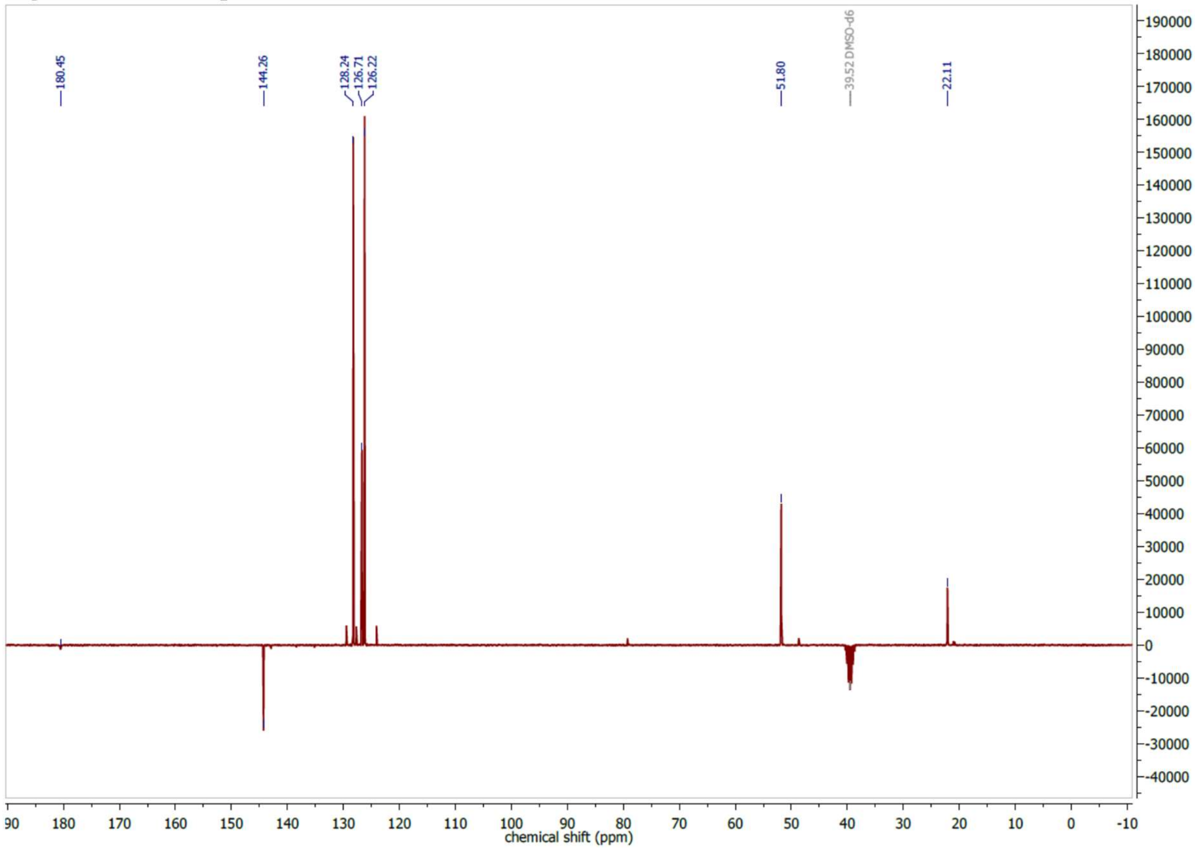

Fig. X8. ^{13}C DEPTQ NMR spectrum of TSCmB in DMSO-d_6 at 75 MHz.

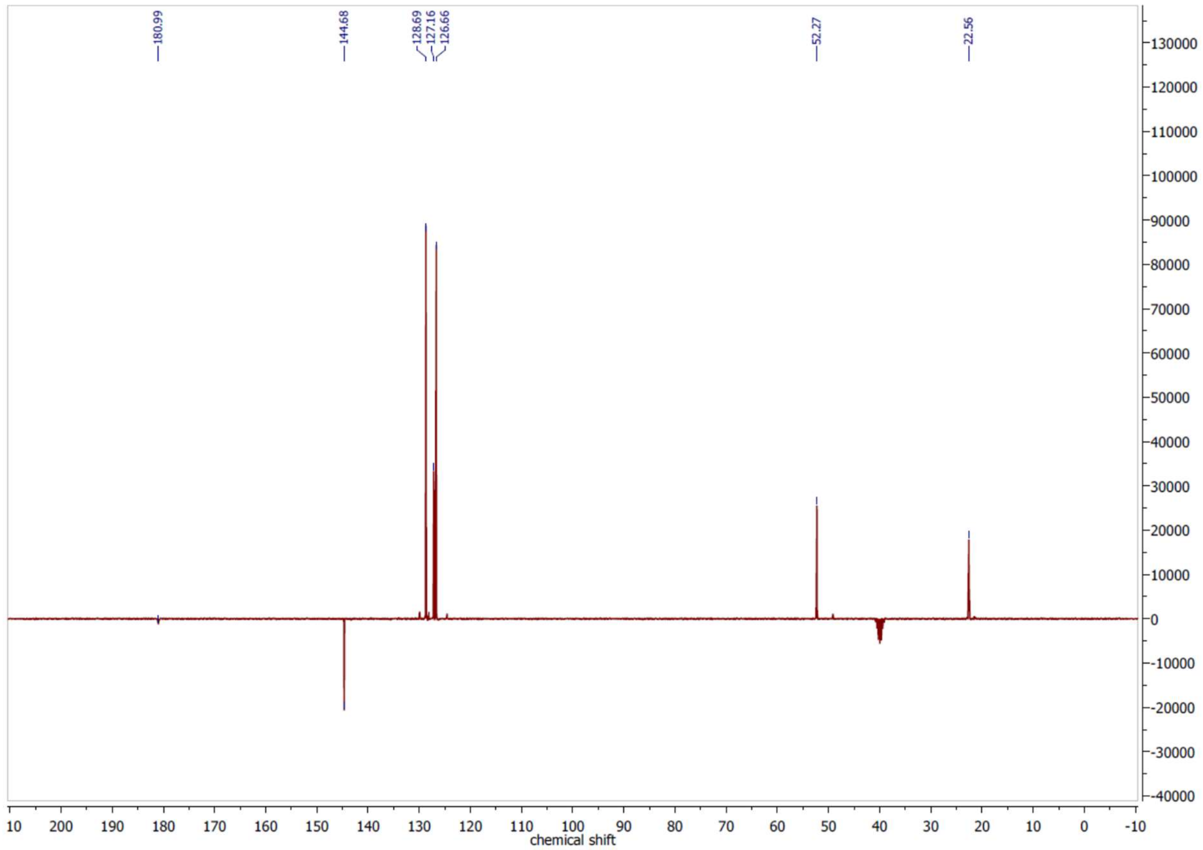

Fig. X9. ^{13}C APT NMR spectrum of TSCmB in DMSO-d_6 at 75 MHz.

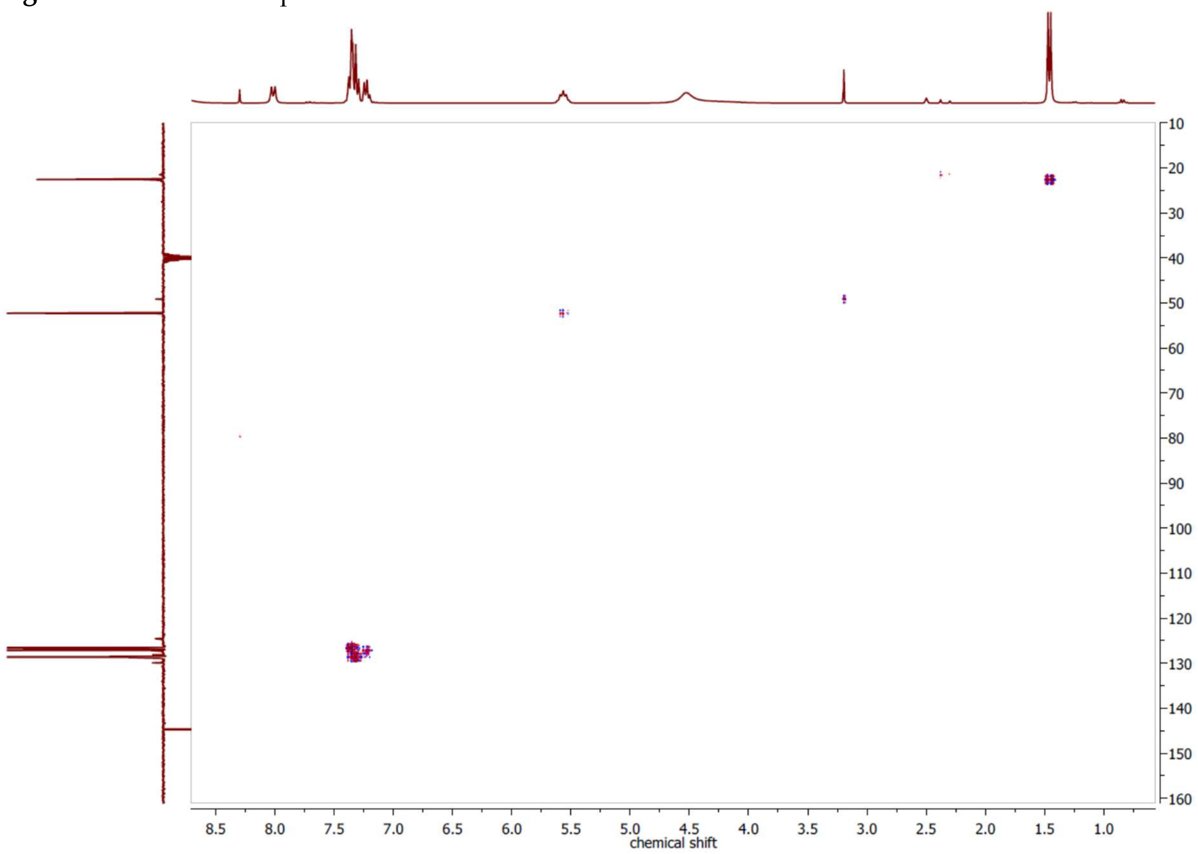

Fig. X10. ^{1}H, ^{13}C HSQC NMR spectrum of TSCmB in DMSO-d_6.

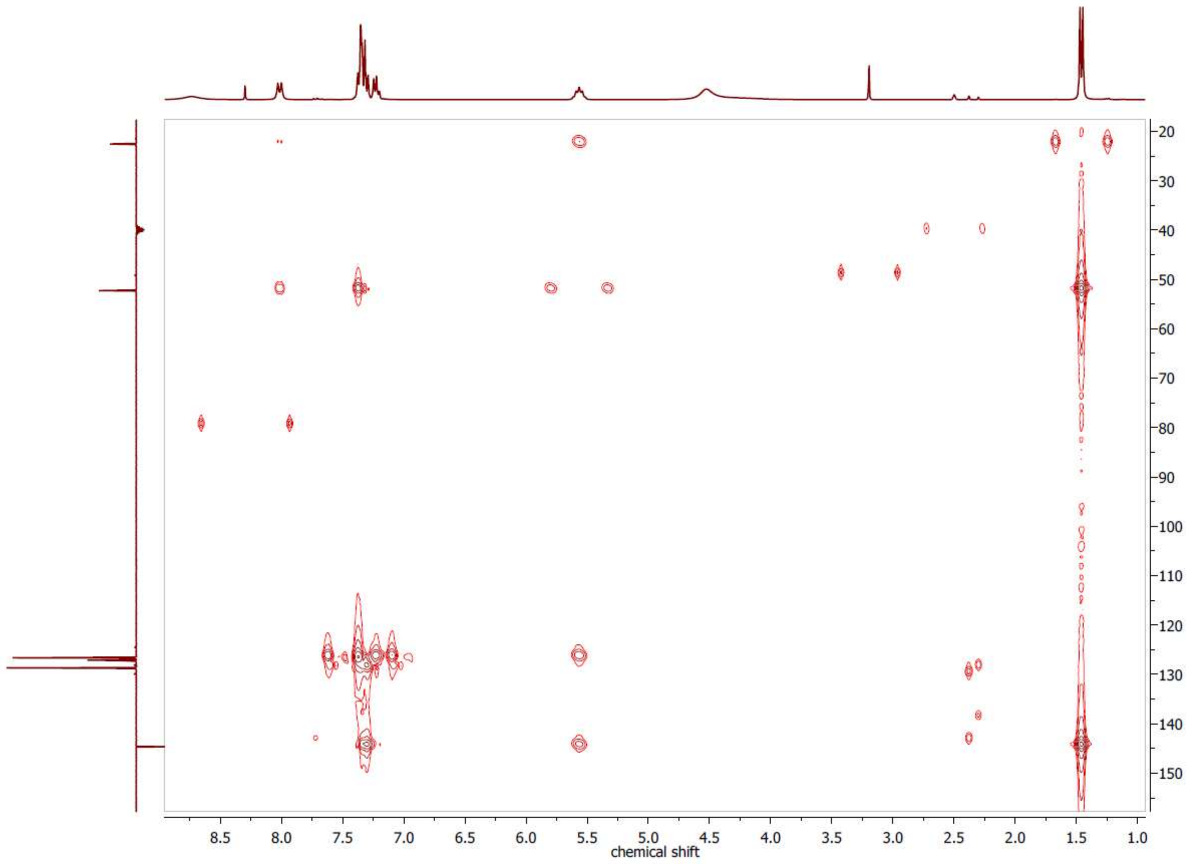

Fig. X11. ^{1}H, ^{13}C HMBC NMR spectrum of TSCmB in DMSO-d_6.

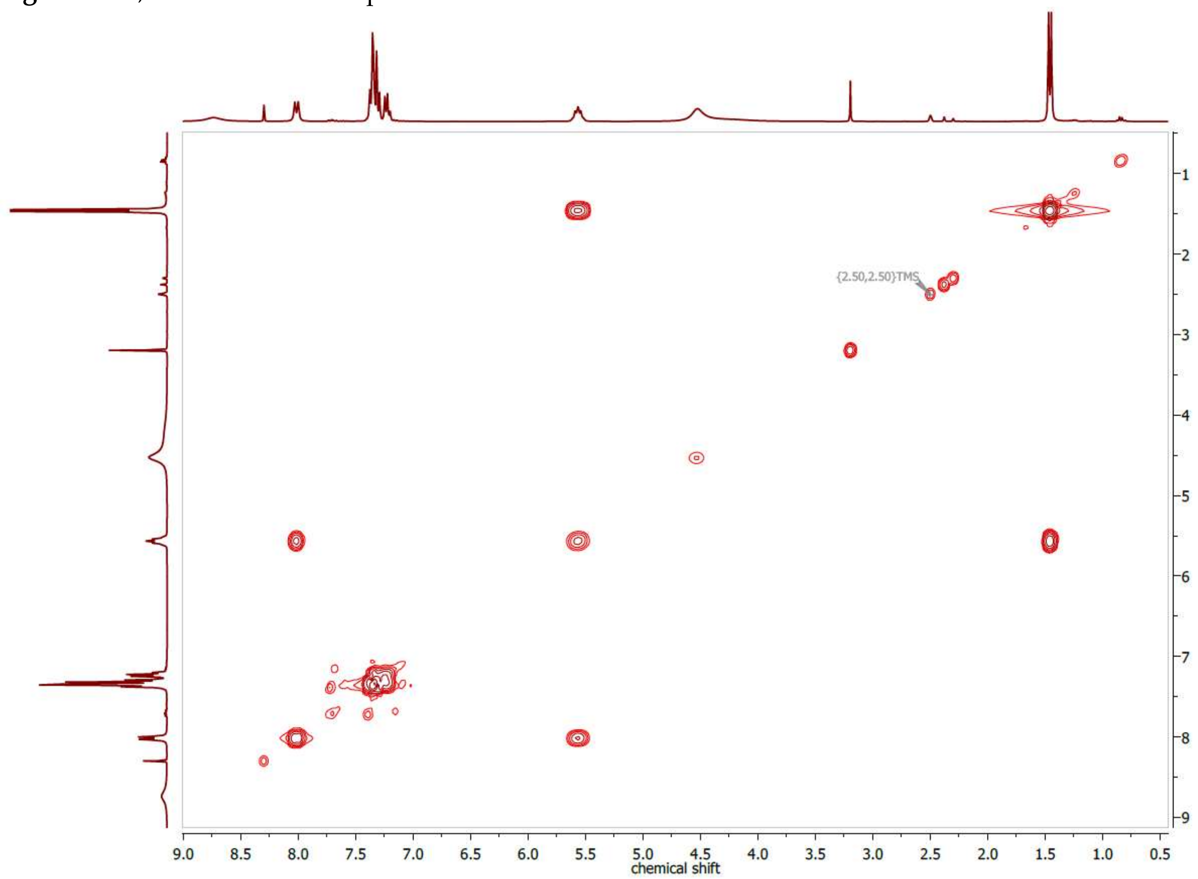

Fig. X12. ^{1}H, ^{1}H COSY NMR spectrum of TSCmB in DMSO-d_6.

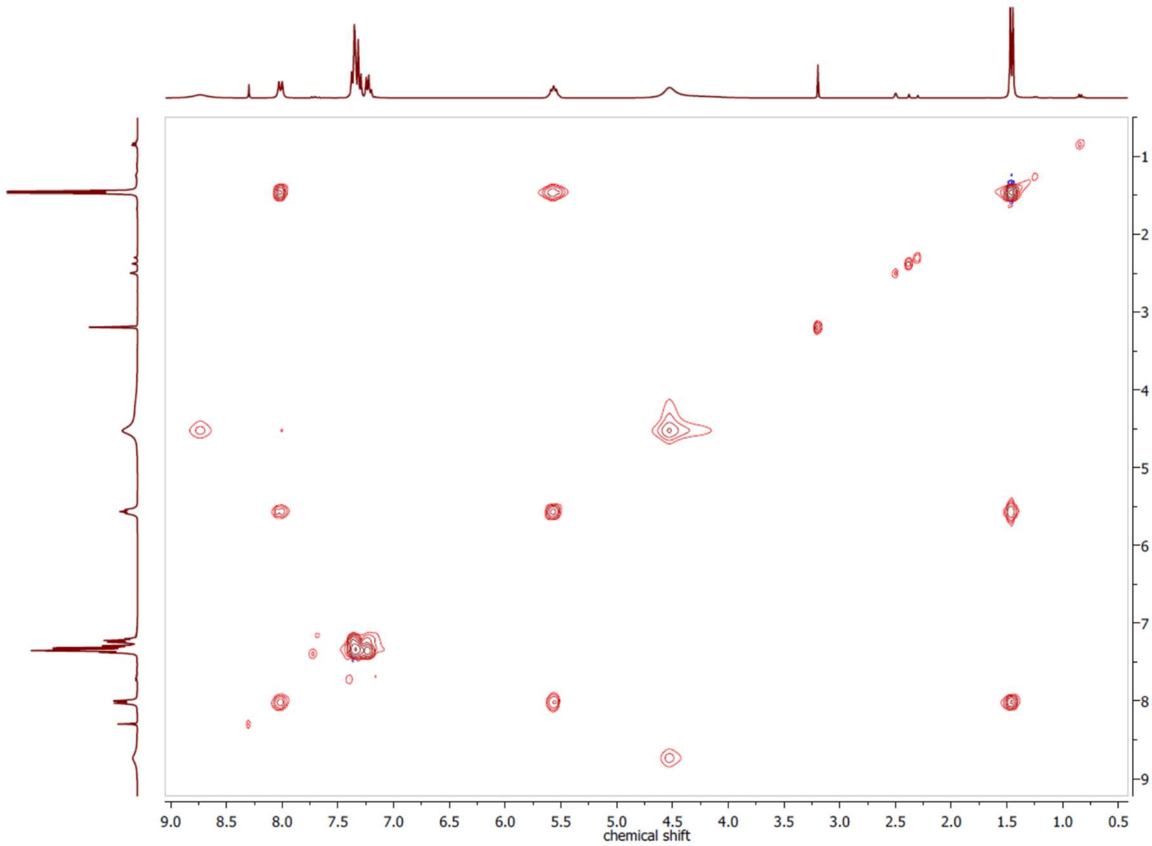

Fig. X13 ^{1}H, ^{1}H TOCSY NMR spectrum of TSCmB in DMSO-d_6.

protoligands:

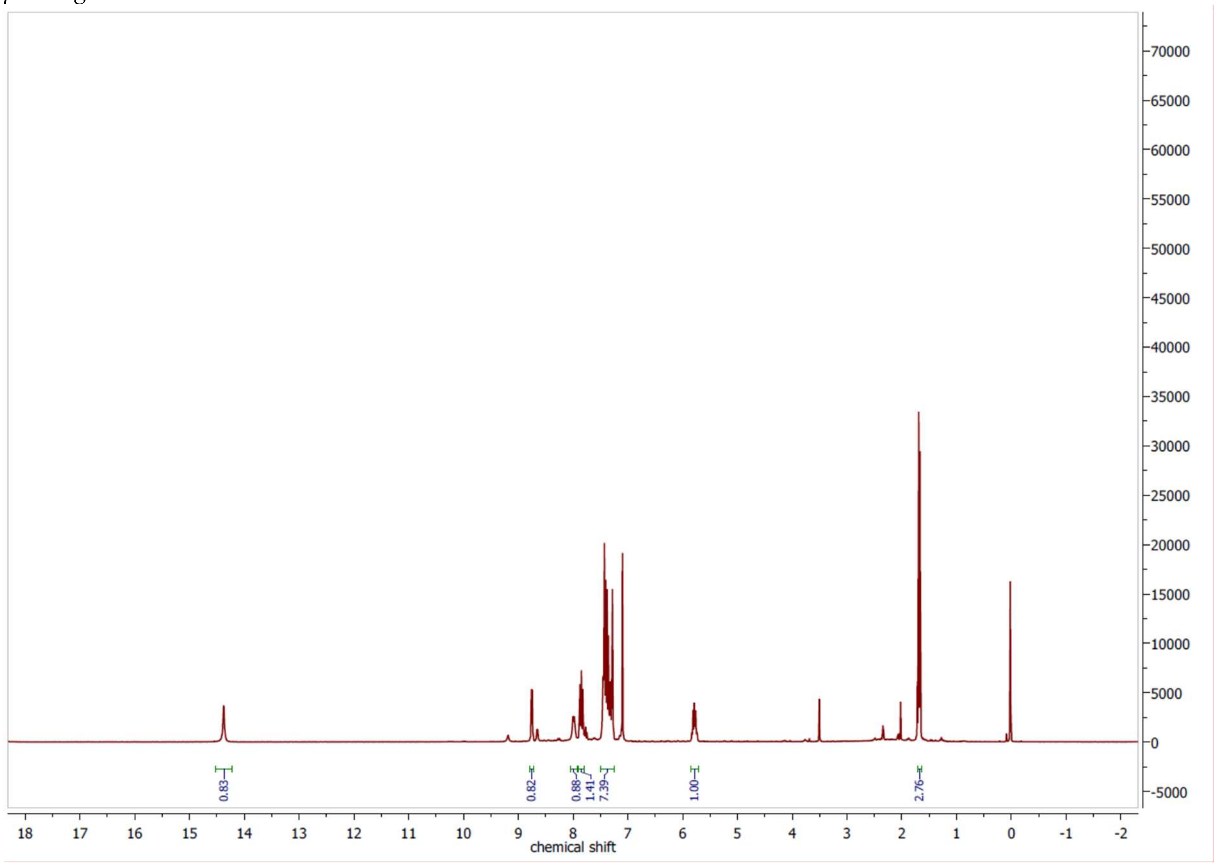

Fig. X14. ^{1}H NMR spectrum of HfpyTSCmB in $CDCl_3$ at 300 MHz.

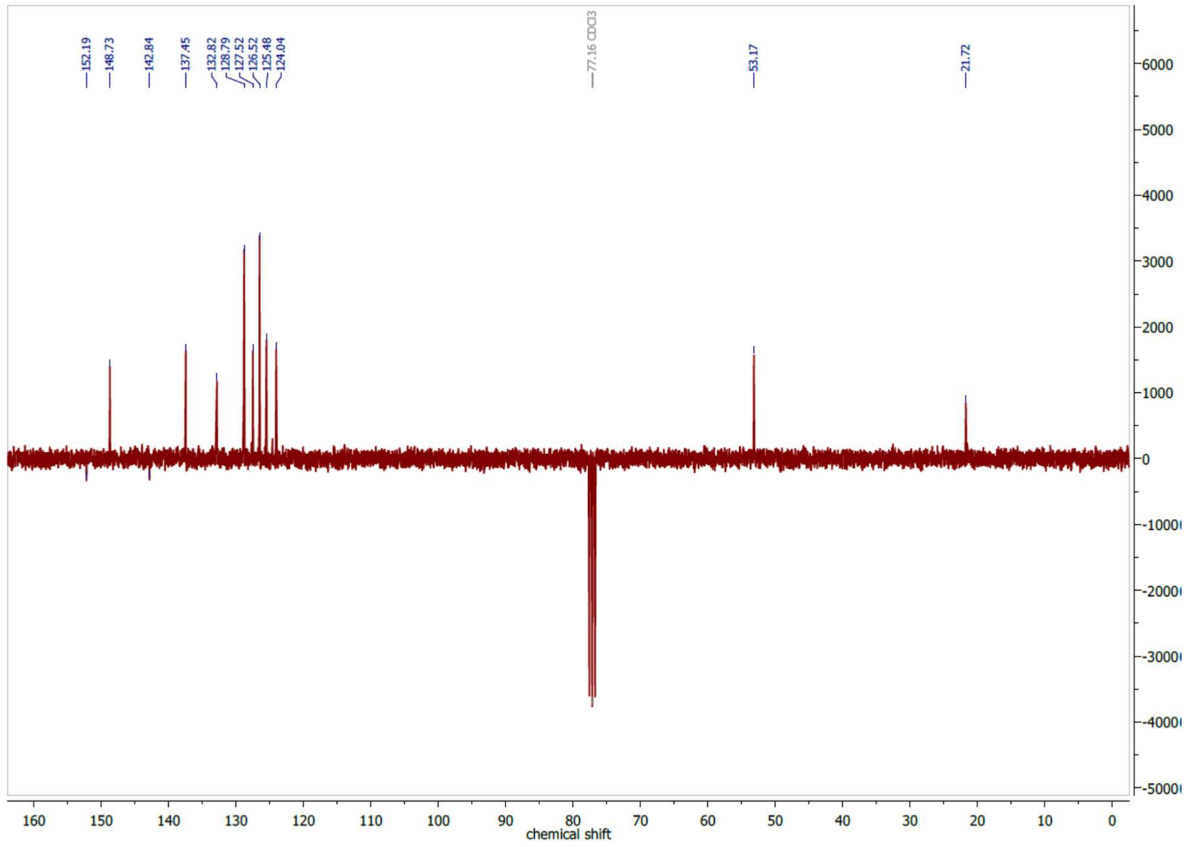

Fig. X15. ^{13}C DEPTQ NMR spectrum of HfpyTSCmB in $CDCl_3$ at 75 MHz.

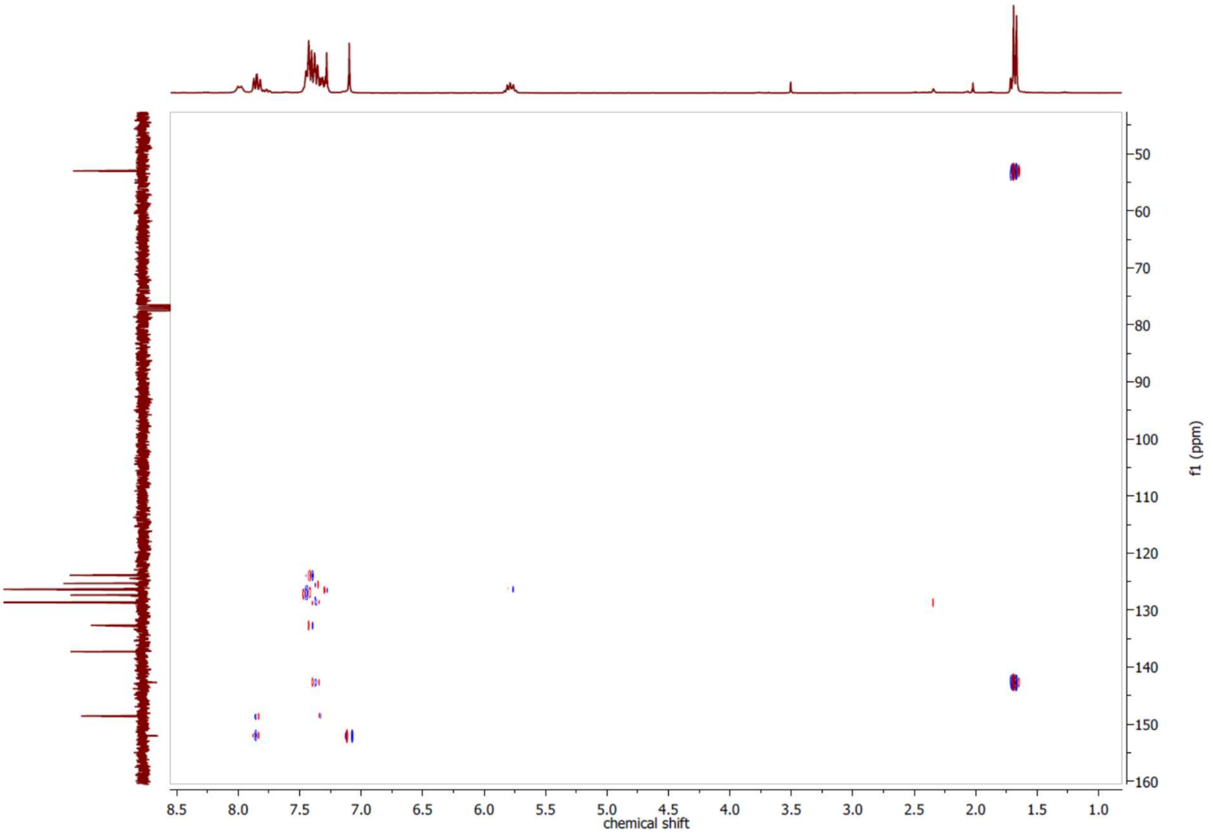

Fig. X16. $^{1}H,^{13}C$ HMBC NMR spectrum of HfpyTSCmB in $CDCl_3$.

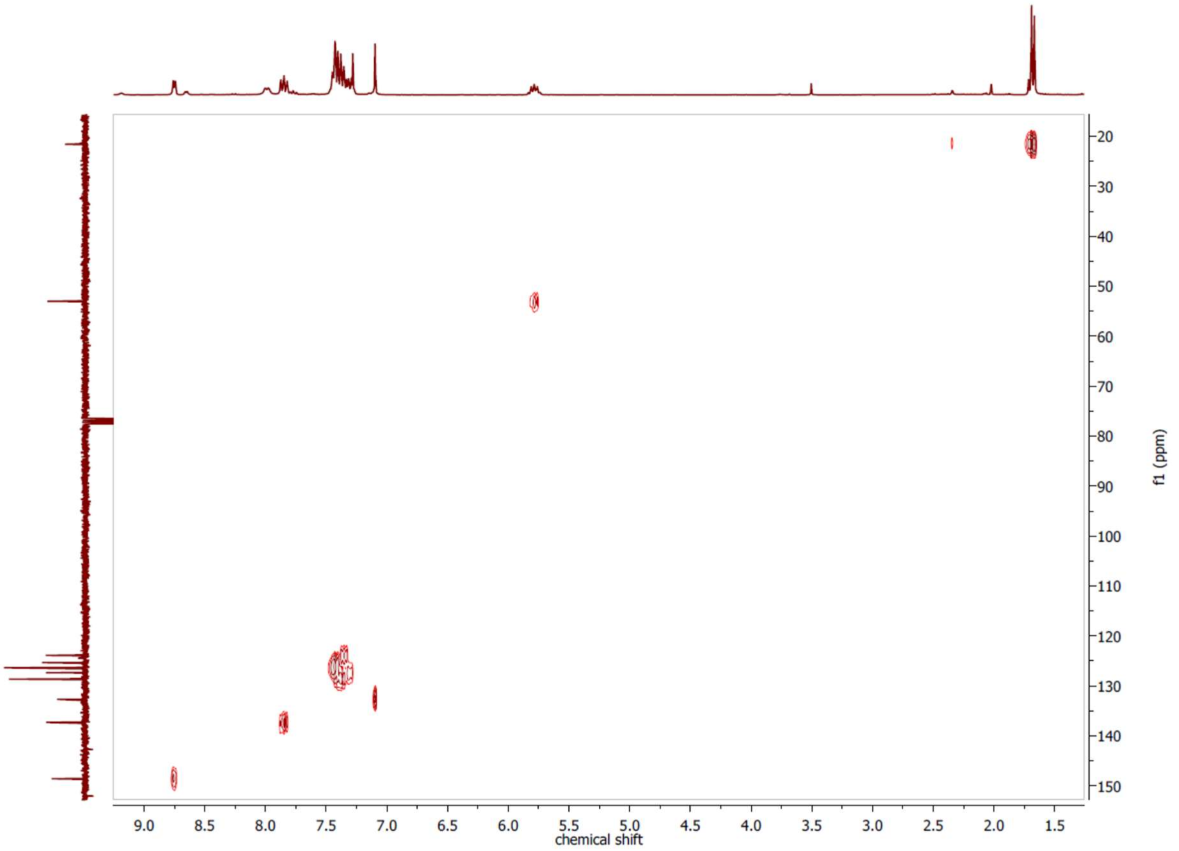

Fig. X17. $^{1}H,^{13}C$ HSQC NMR spectrum of HfpyTSCmB in $CDCl_3$.

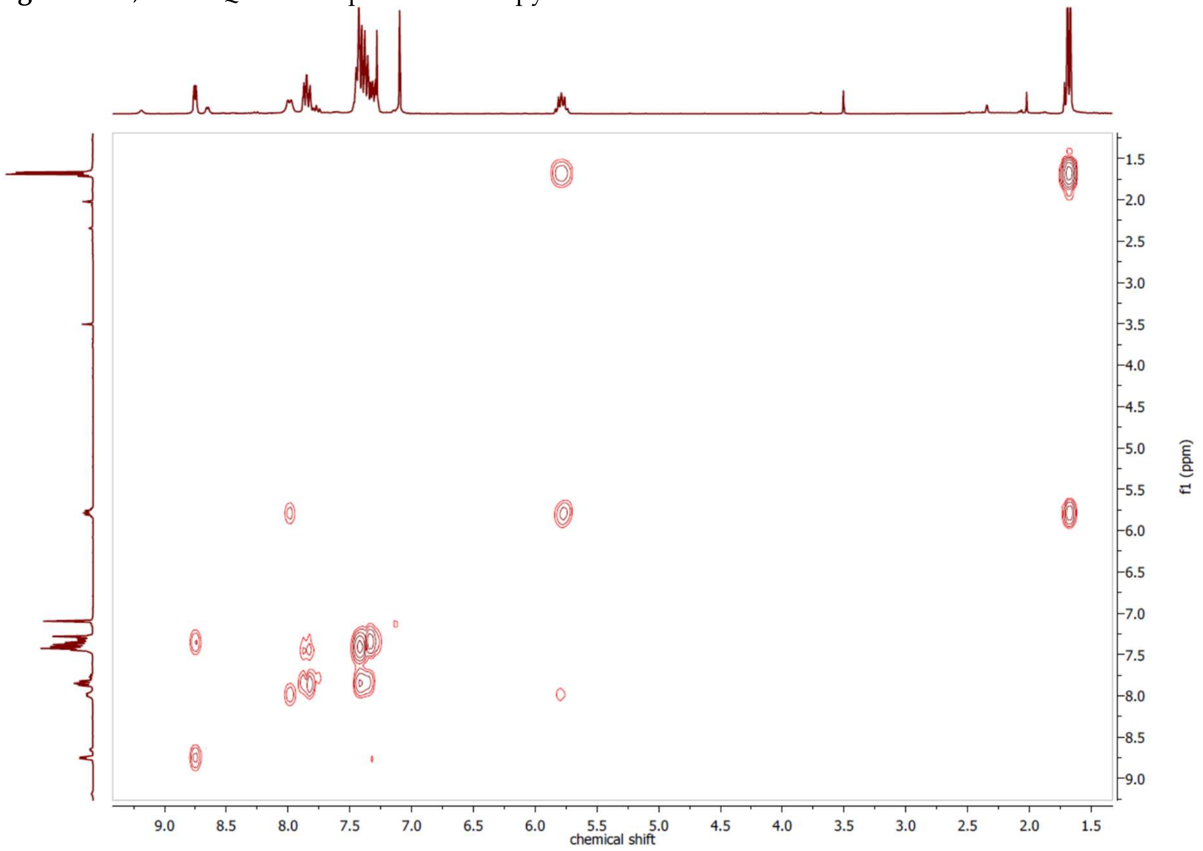

Fig. X18. $^{1}H,^{13}C$ COSY NMR spectrum of HfpyTSCmB in $CDCl_3$.

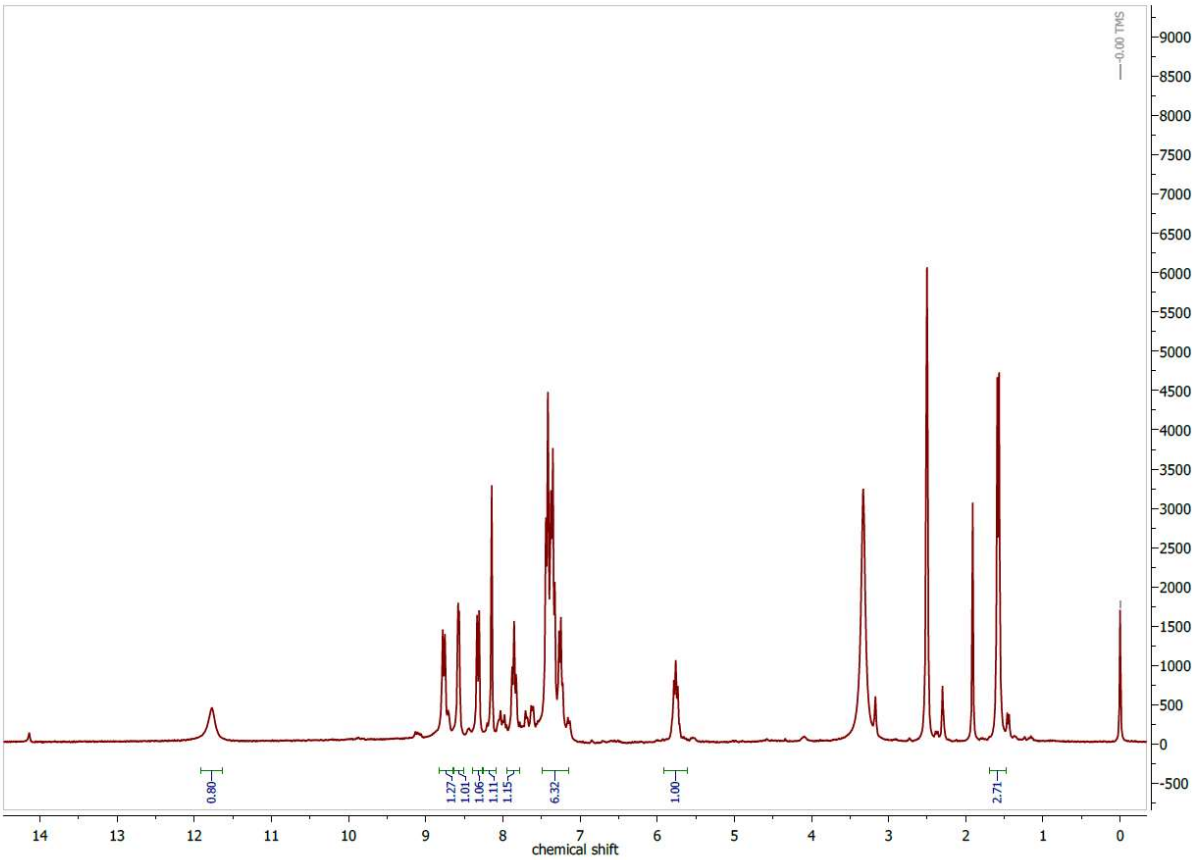

Fig. X19. ^{1}H spectrum of HfpyTSCmB in DMSO-d_6 at 300 MHz.

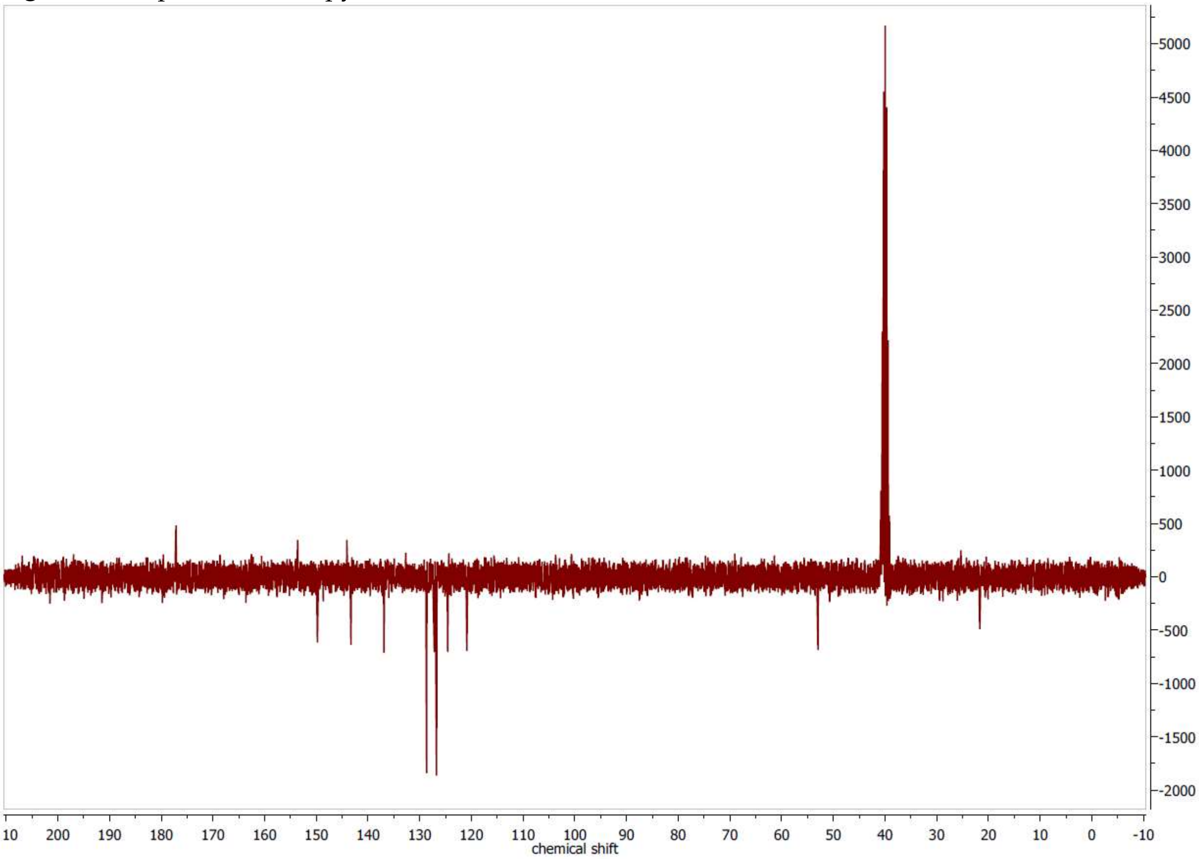

Fig. X20. ^{13}C DEPTQ NMR spectrum of HfpyTSCmB in DMSO-d_6 at 75 MHz.

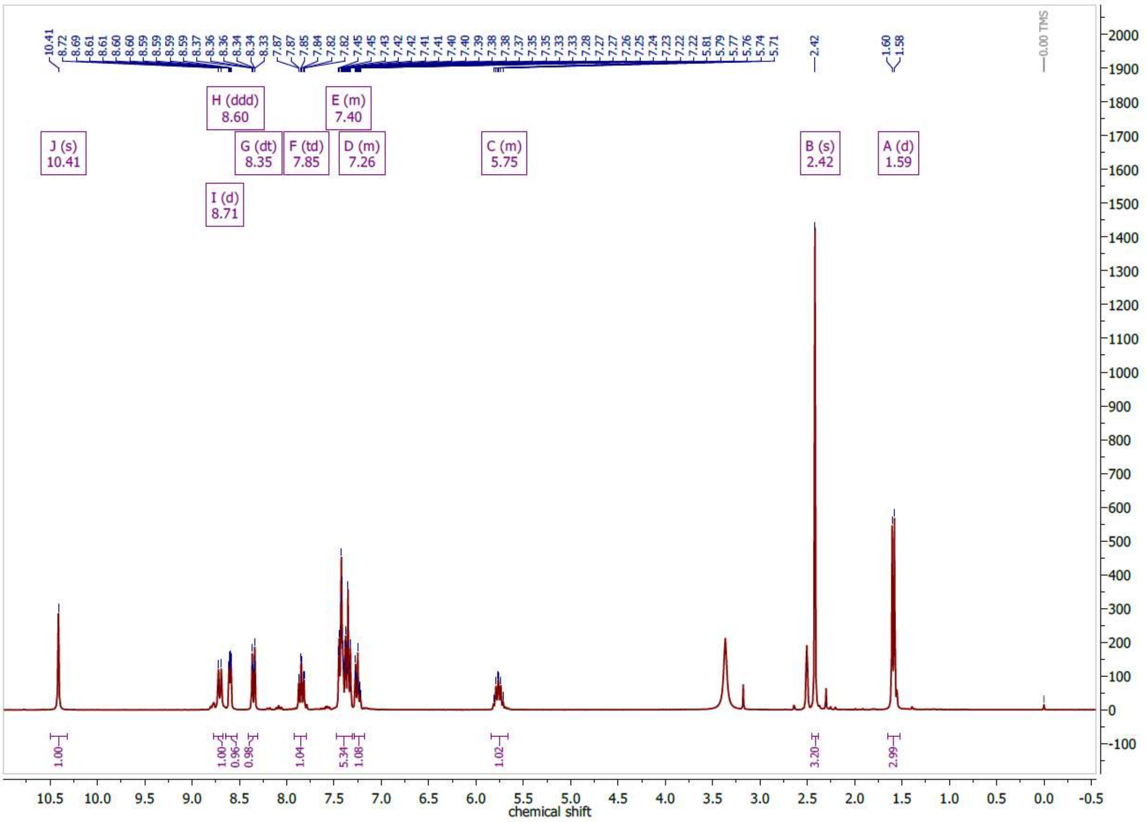

Fig. X21. ^{1}H NMR spectrum of HapyTSCmB in DMSO-d_6 at 300 MHz.

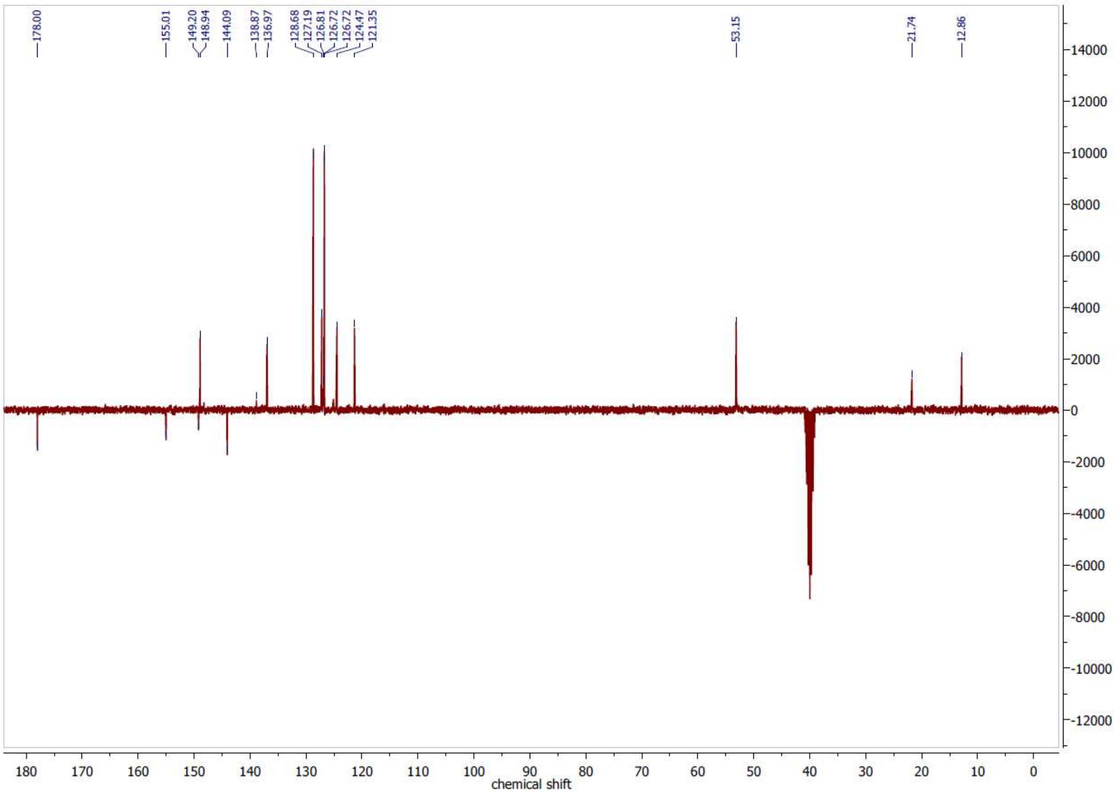

Fig. X22. ^{13}C DEPTQ NMR spectrum of HapyTSCmB in DMSO-d_6 at 75 MHz.

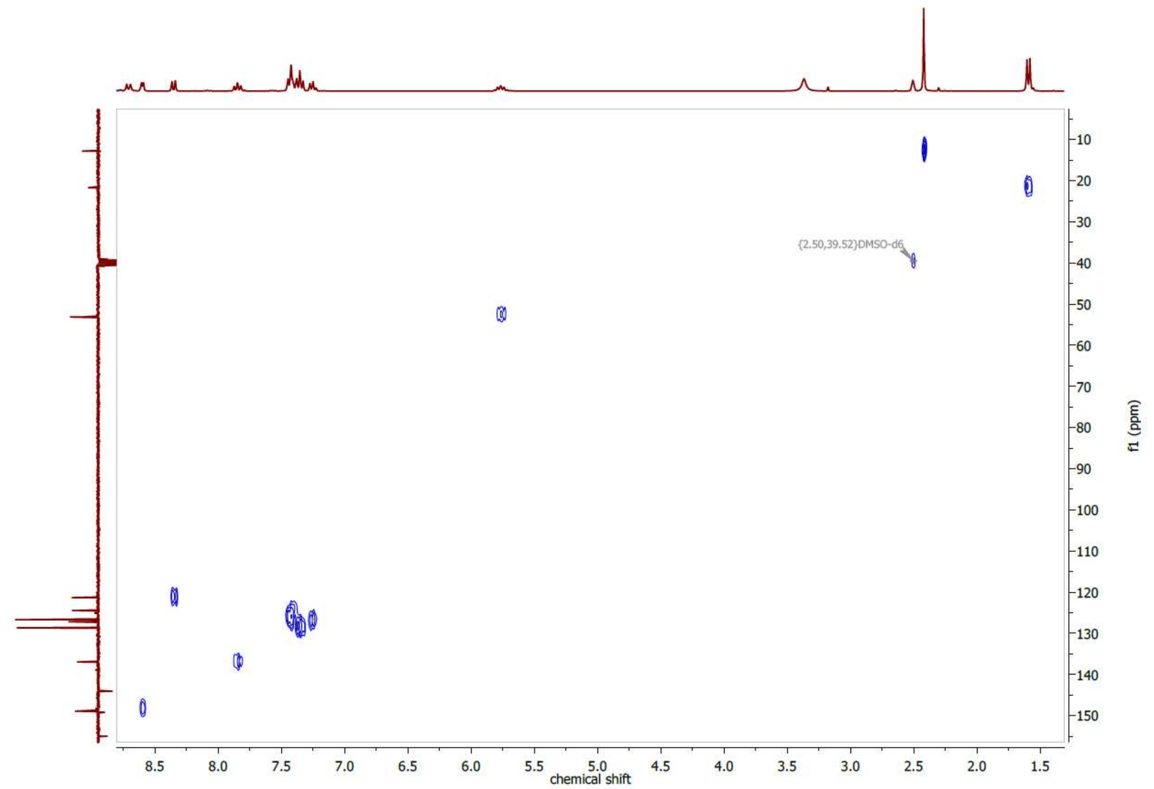

Fig. X23. ^{1}H,^{13}C HSQC NMR spectrum of HapyTSCmB in DMSO-d_6.

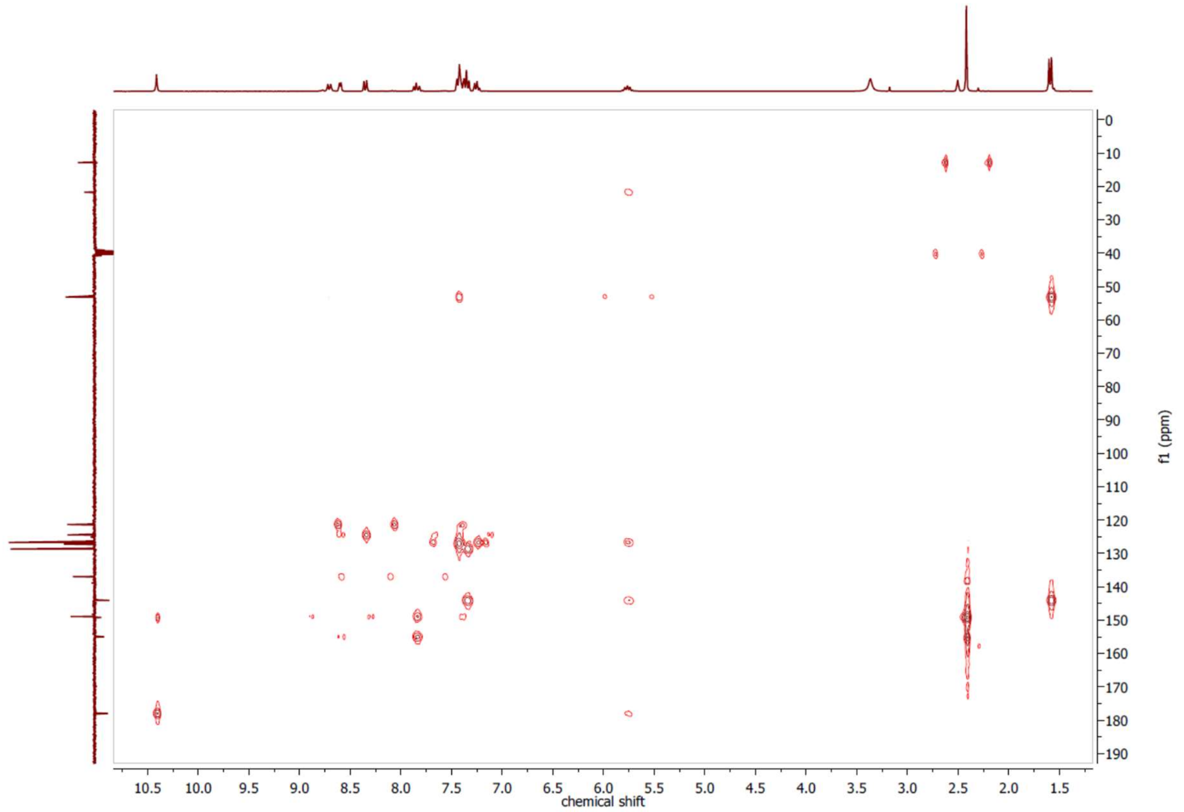

Fig. X24. ^{1}H,^{13}C HMBC NMR spectrum of HapyTSCmB in DMSO-d_6.

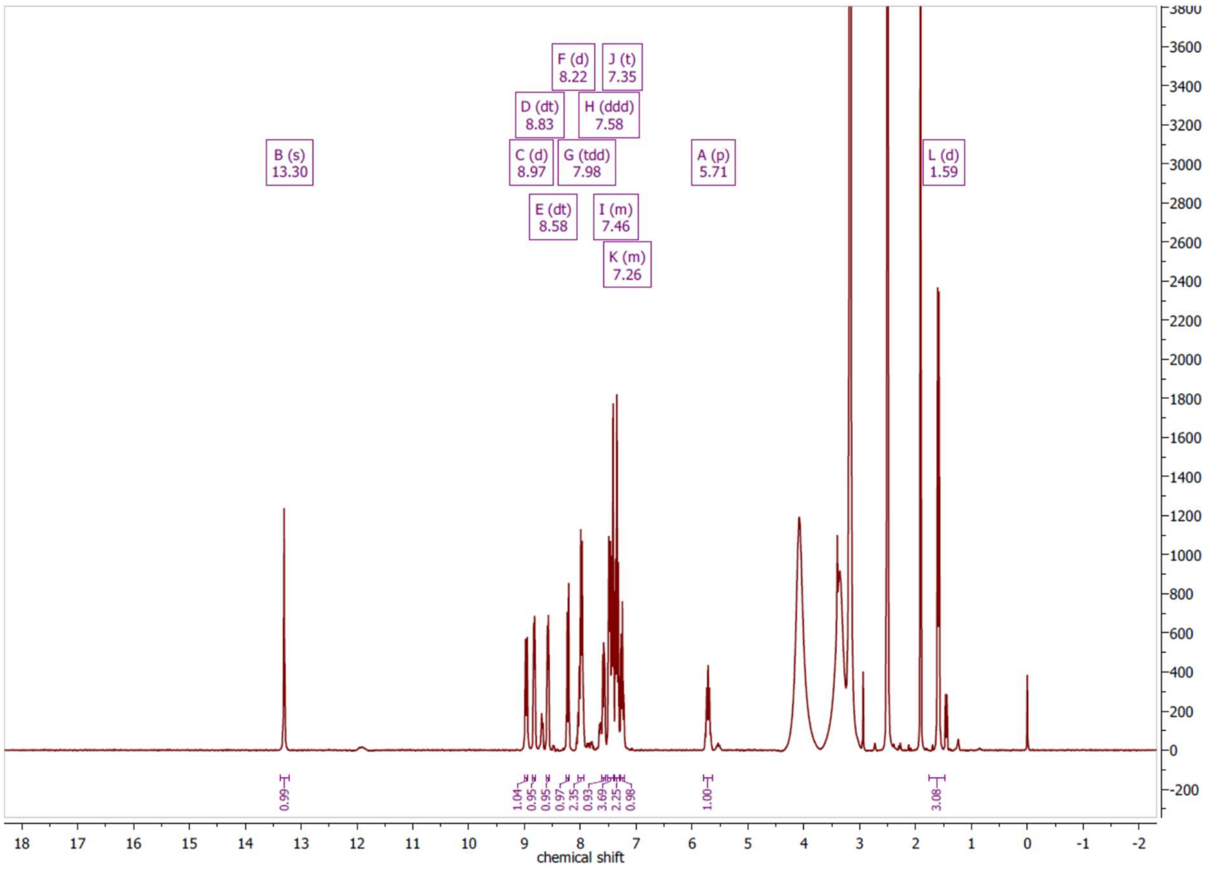

Fig. X25. ^{1}H NMR spectrum of HdpyTSCmB in DMSO-d_6 at 300 MHz.

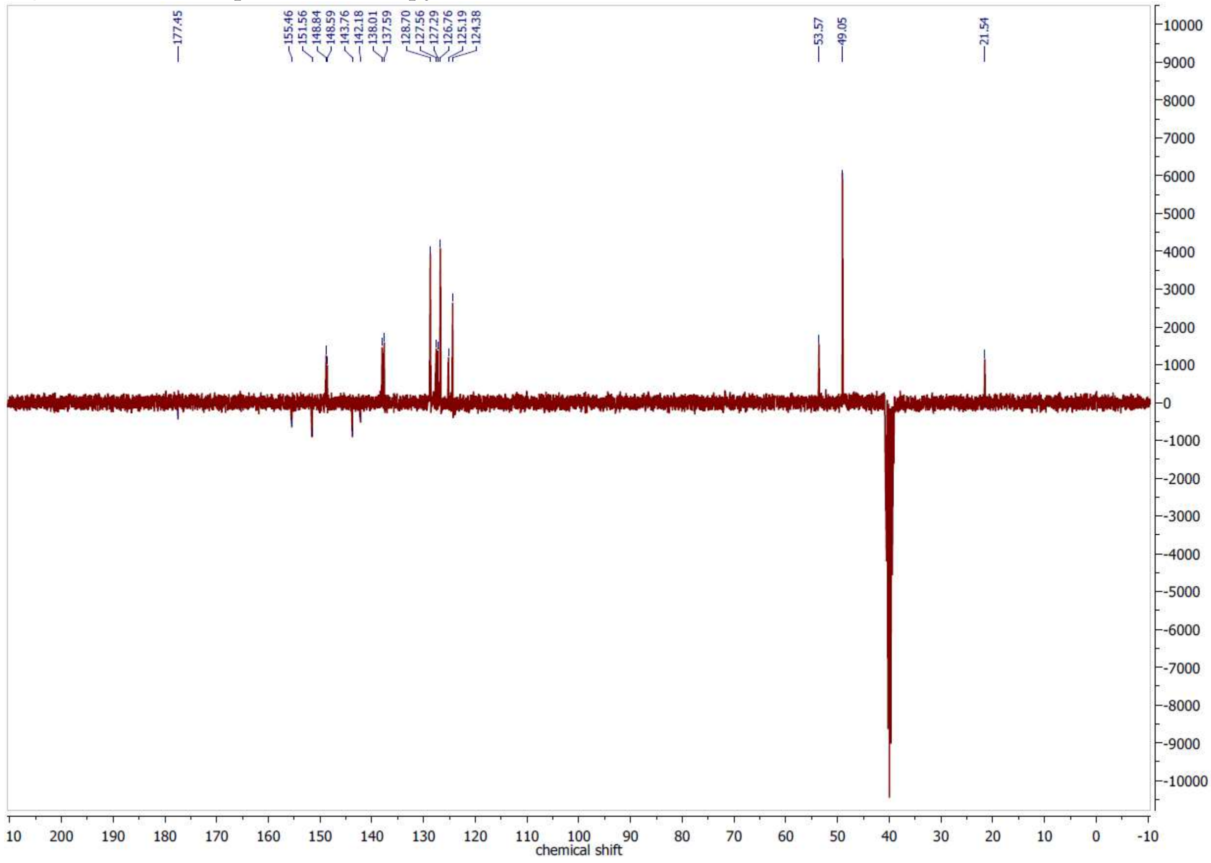

Fig. X26. ^{13}C APT NMR spectrum of HdpyTSCmB in DMSO-d_6 at 75 MHz.

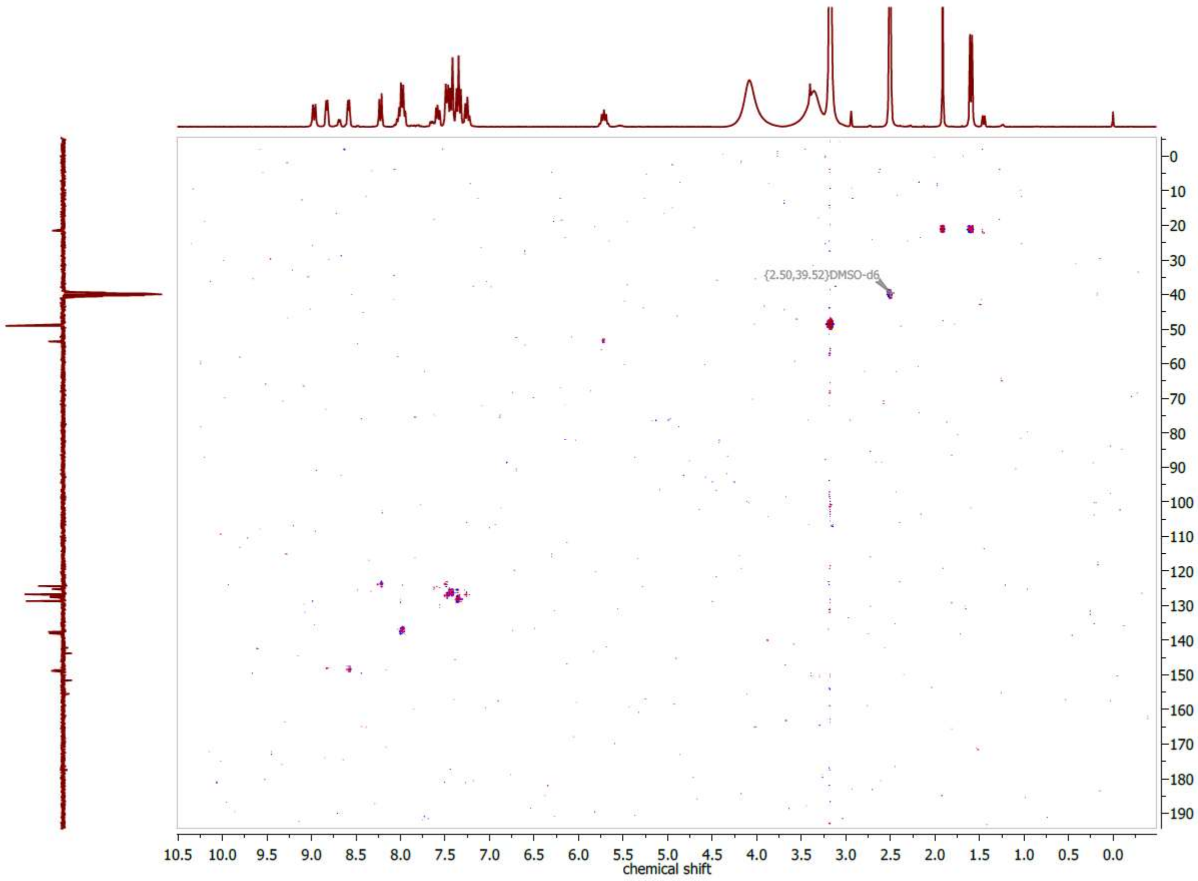

Fig. X27. ${}^{1}H,{}^{13}C$ HSQC NMR spectrum of HdpyTSCmB in DMSO-d_6.

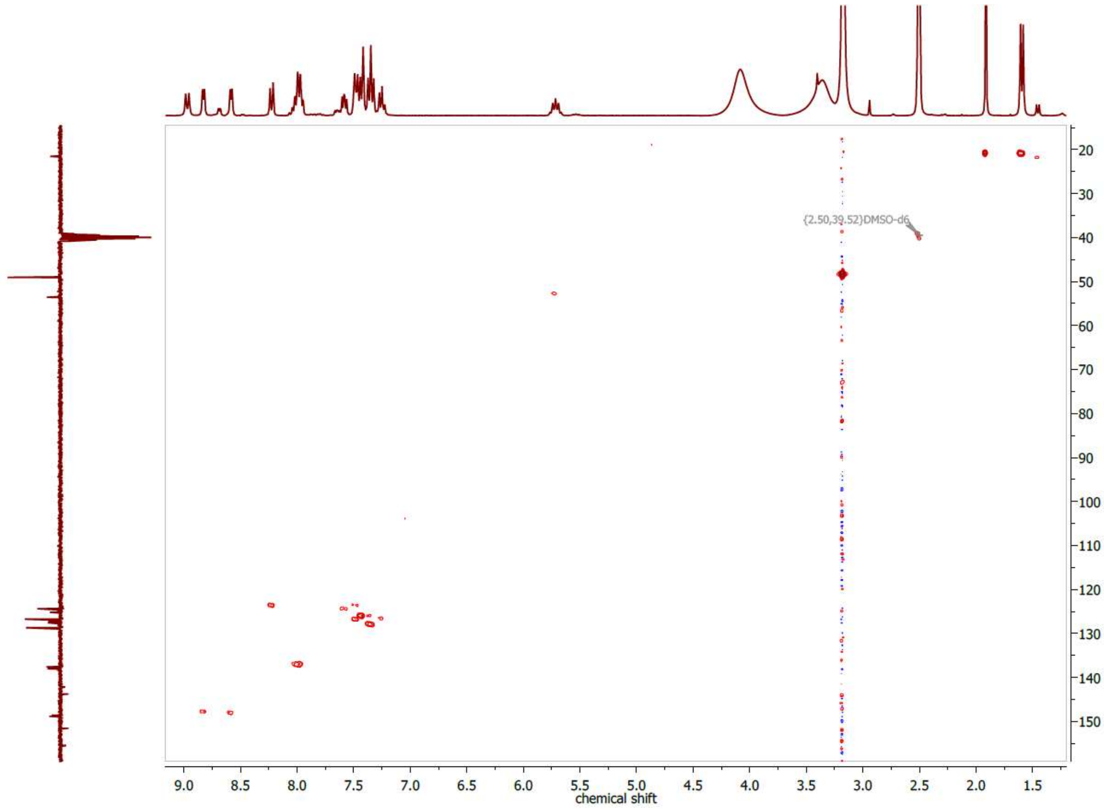

Fig. X28. ${}^{1}H,{}^{13}C$ HMBC NMR spectrum of HdpyTSCmB in DMSO-d_6.

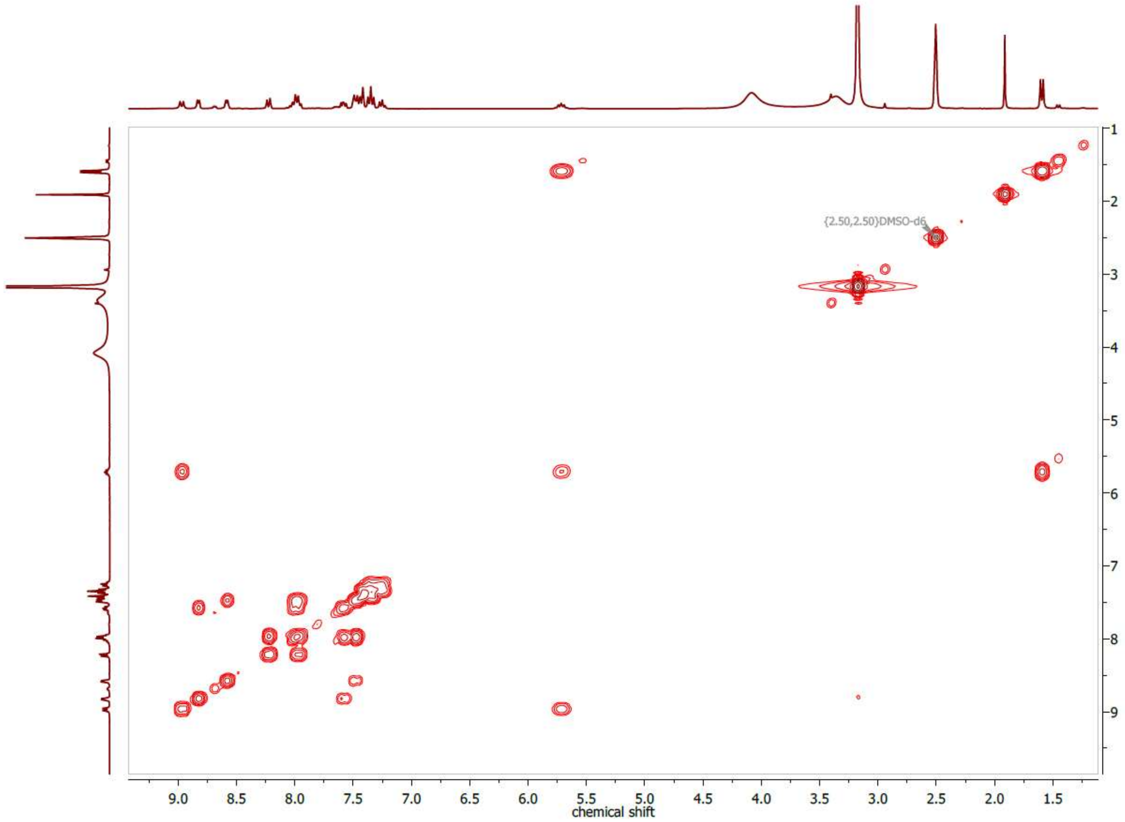

Fig. X29. $^{1}H,^{1}H$ COSY NMR spectrum of HdpyTSCmB in DMSO-d_6.

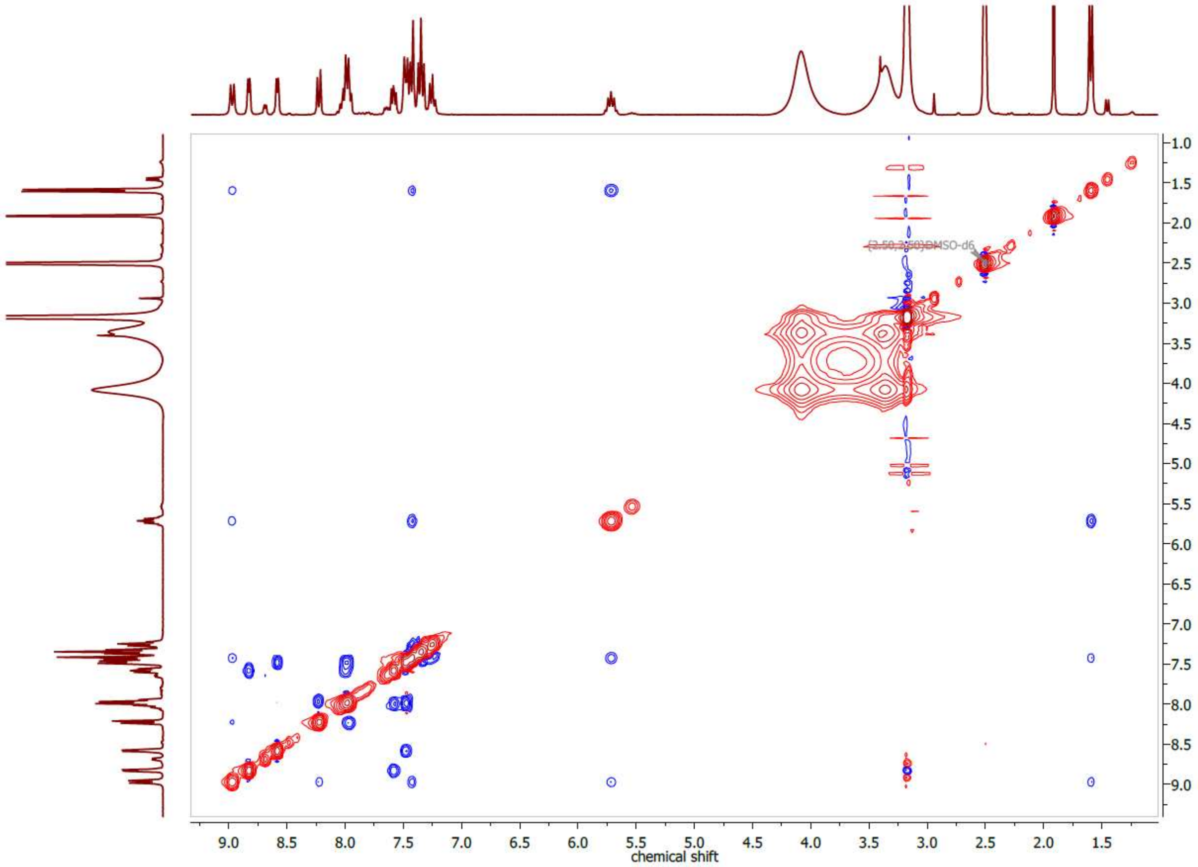

Fig. X30. $^{1}H,^{1}H$ NOESY NMR spectrum of HdpyTSCmB in DMSO-d_6.

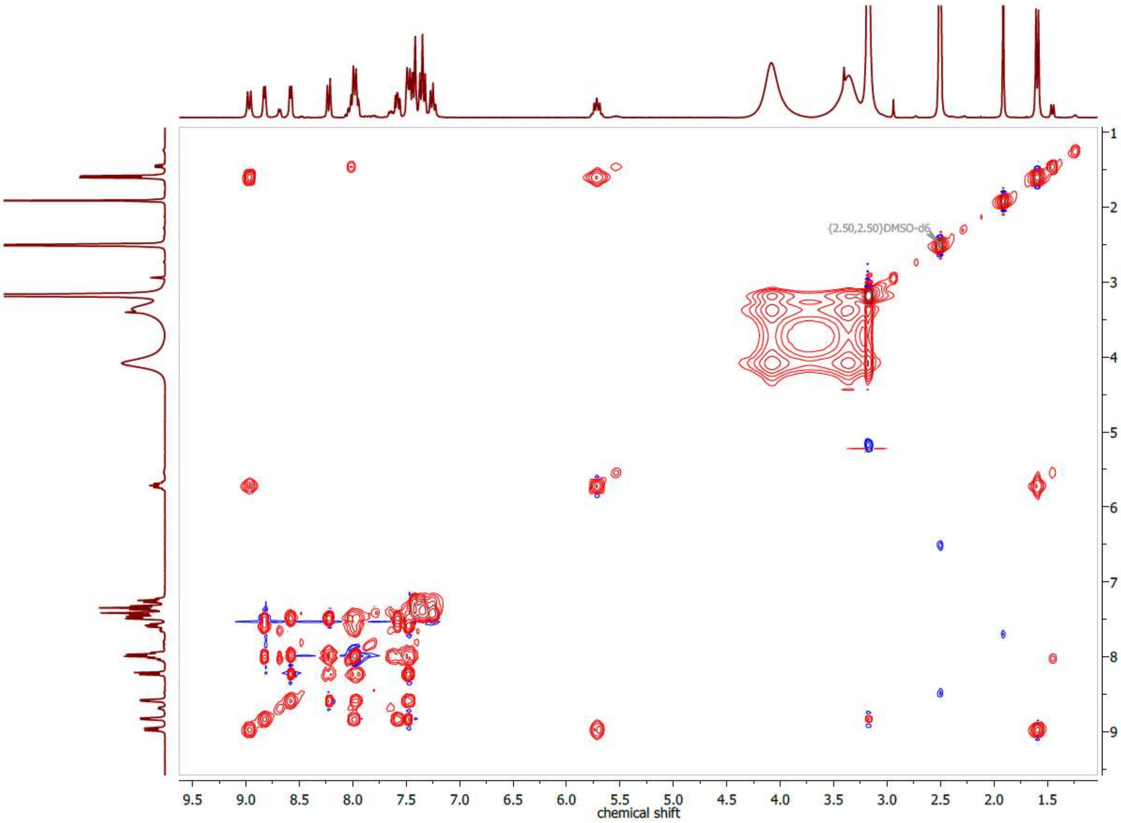

Fig. X31. ^{1}H,^{1}H TOCSY NMR spectrum of HdpyTSCmB in DMSO-d_6.

TSCLp Series

percursors:

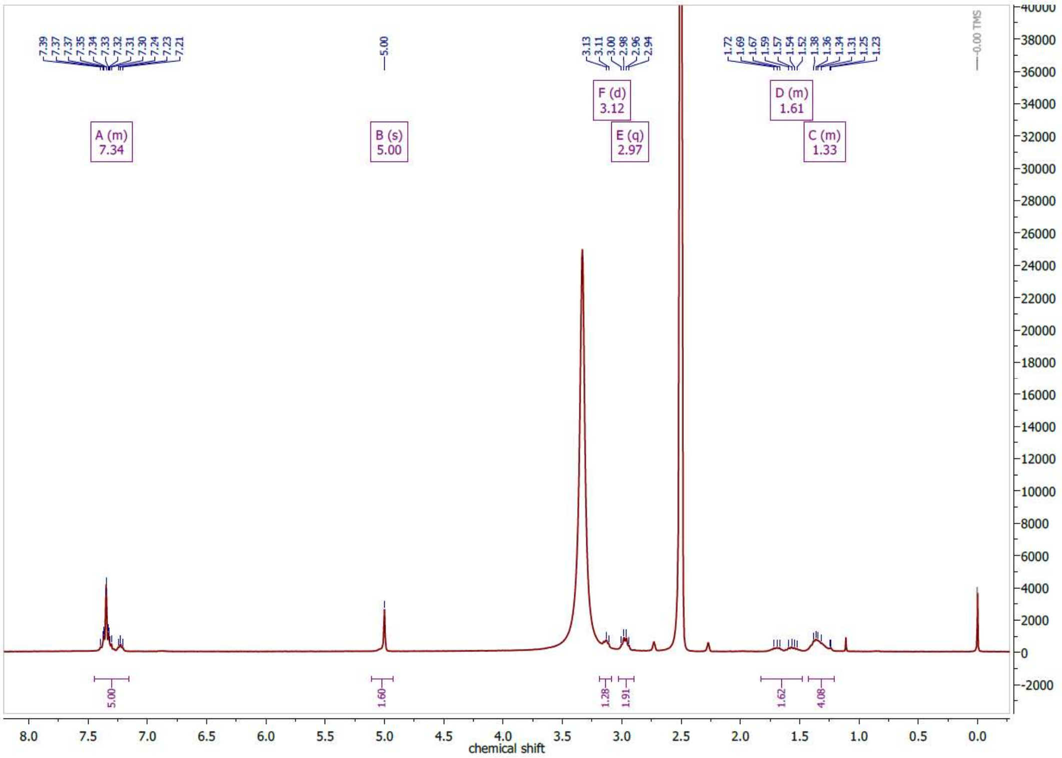

Fig. X32. ^{1}H NMR spectrum of N^6-Cbz lysine in DMSO-d_6 at 300 MHz.

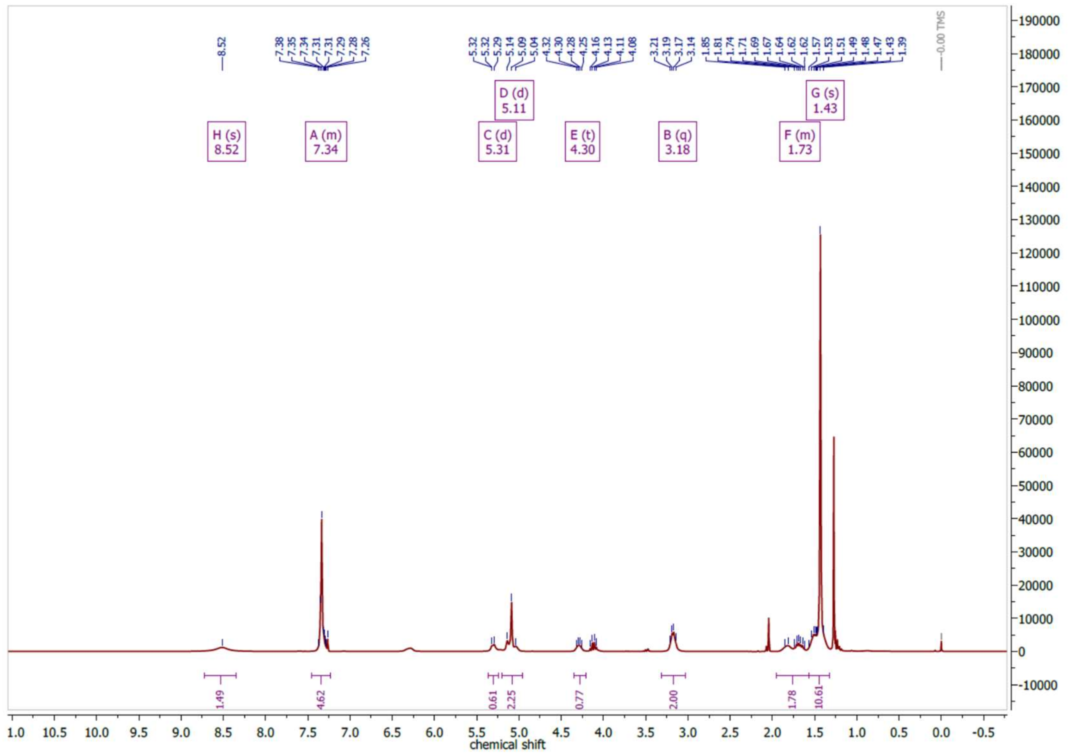

Fig. X33. ^{1}H NMR spectrum of *N*^{6}-Cbz *N*^{1}-Boc lysine in $CDCl_3$ at 300 MHz.

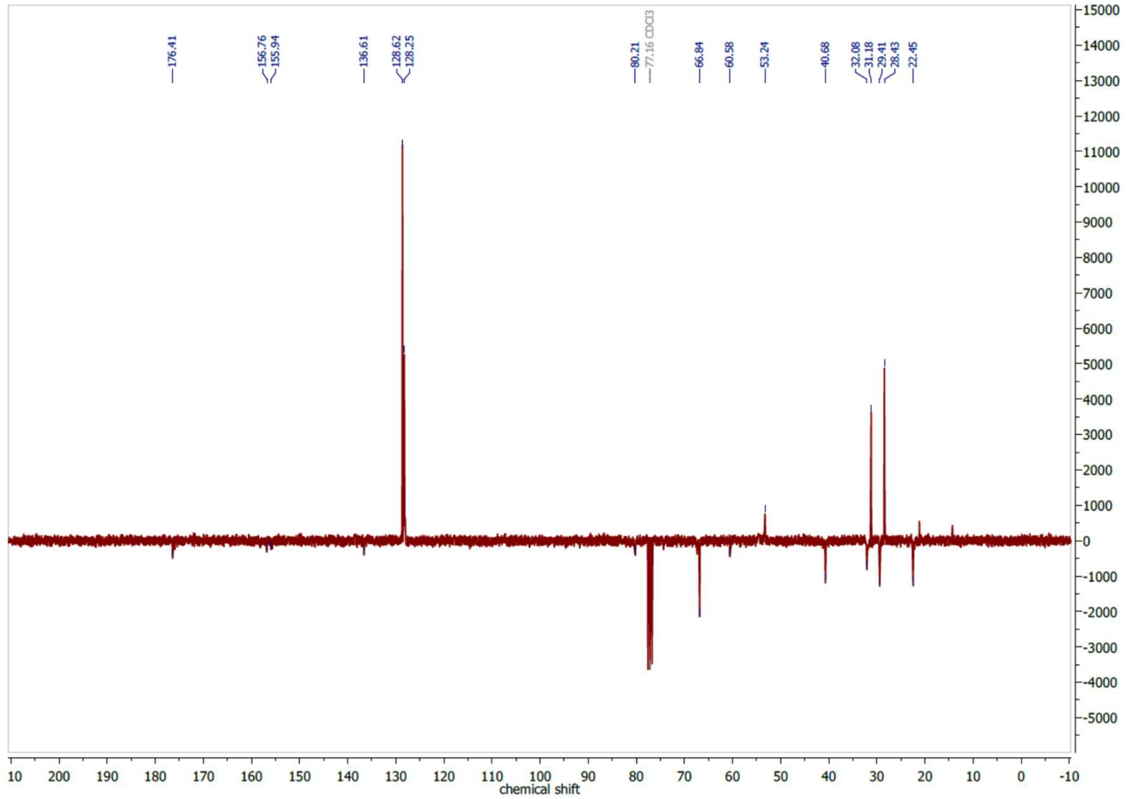

Fig. X34. ^{13}C DEPTQ NMR spectrum of *N*^{6}-Cbz *N*^{1}-Boc lysine in $CDCl_3$ at 75 MHz.

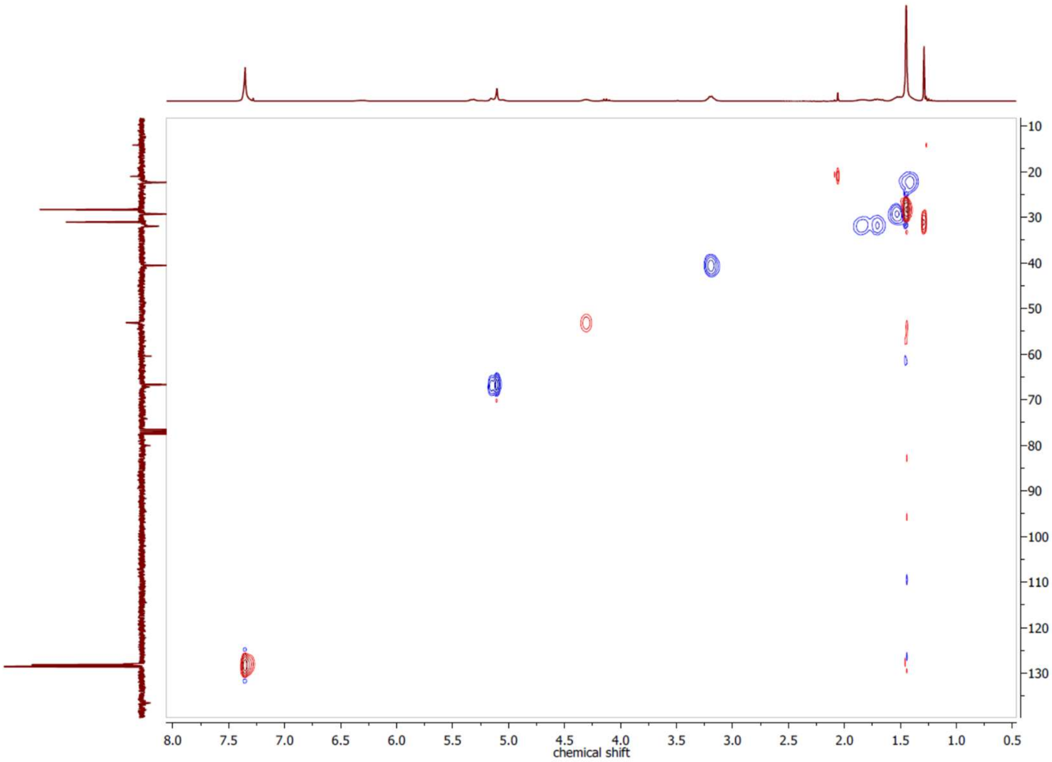

Fig. X35. $^{1}H,^{13}C$ HSQCed NMR spectrum of *N*[6]-Cbz *N*[1]-Boc lysine in $CDCl_3$.

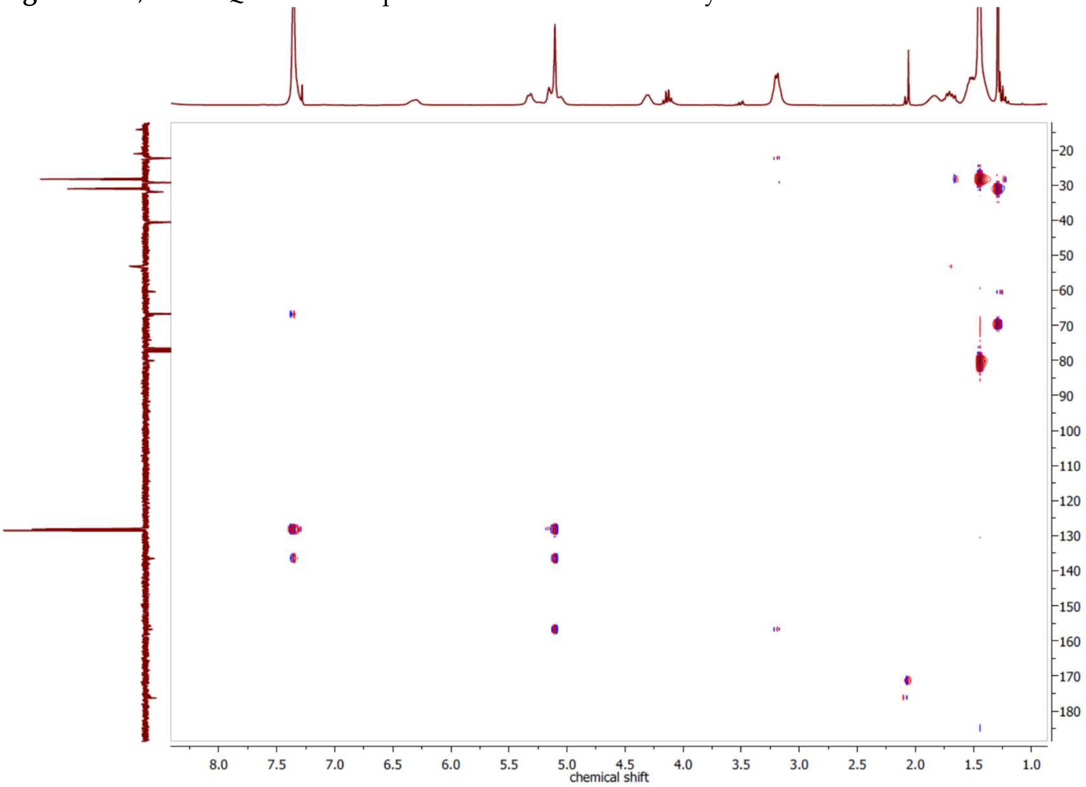

Fig. X36. $^{1}H,^{13}C$ HMBC NMR spectrum of *N*[6]-Cbz *N*[1]-Boc lysine in $CDCl_3$.

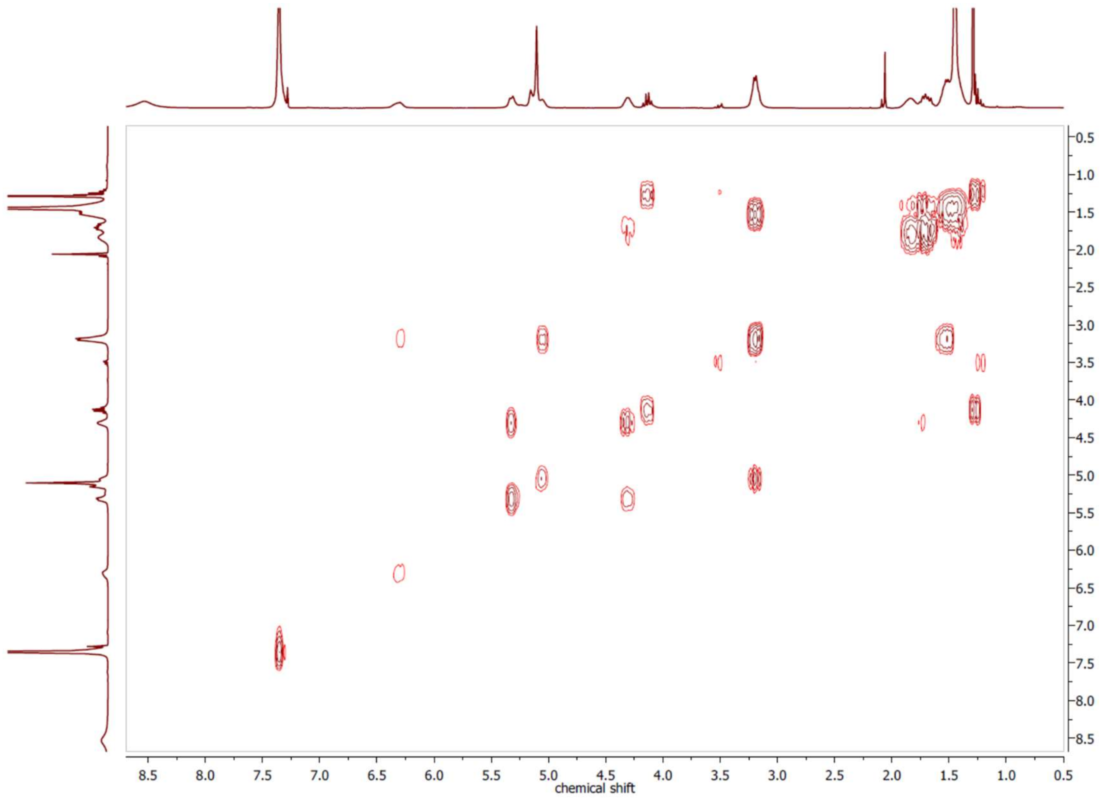

Fig. X37. $^{1}H,^{1}H$ COSY NMR spectrum of *N*[6]-Cbz *N*[1]-Boc lysine in $CDCl_3$.

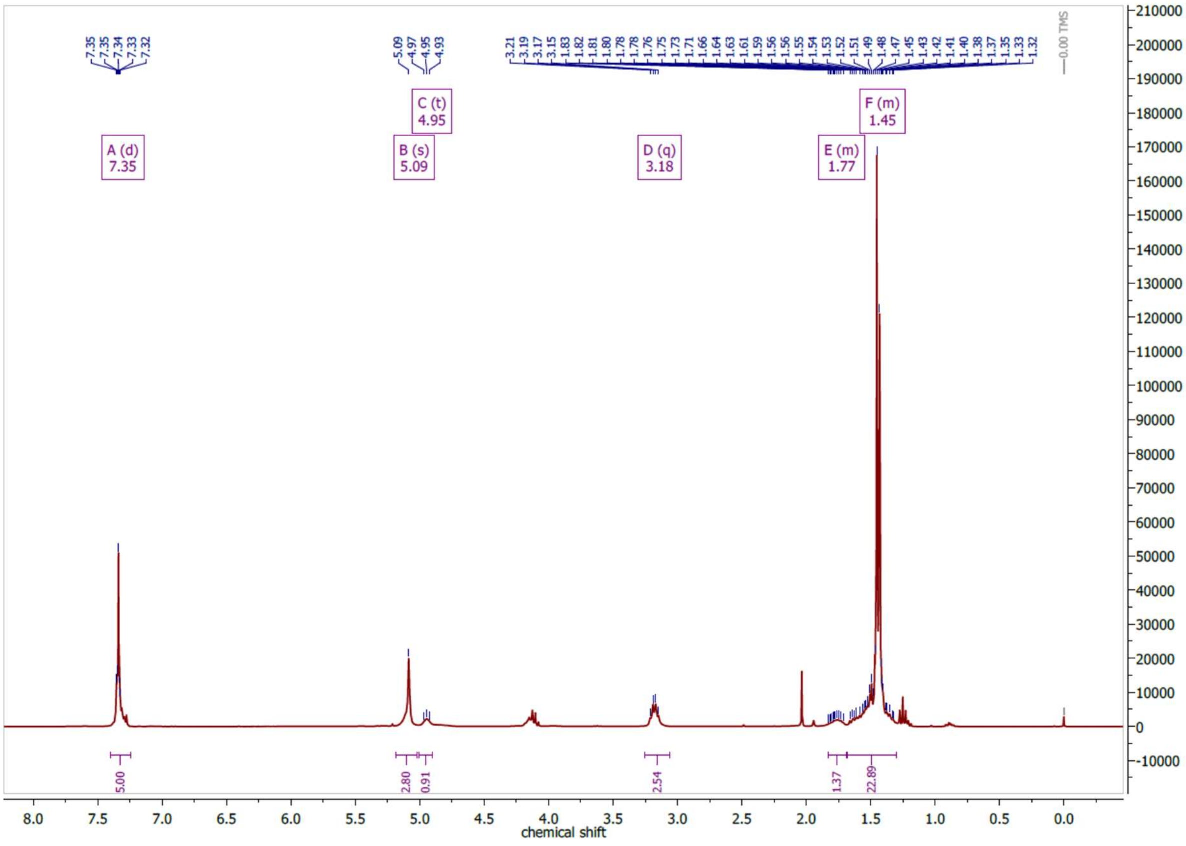

Fig. X38. ^{1}H NMR spectrum of *N*[6]-Cbz *N*[1]-Boc *C*-tBu lysine in $CDCl_3$ at 300 MHz.

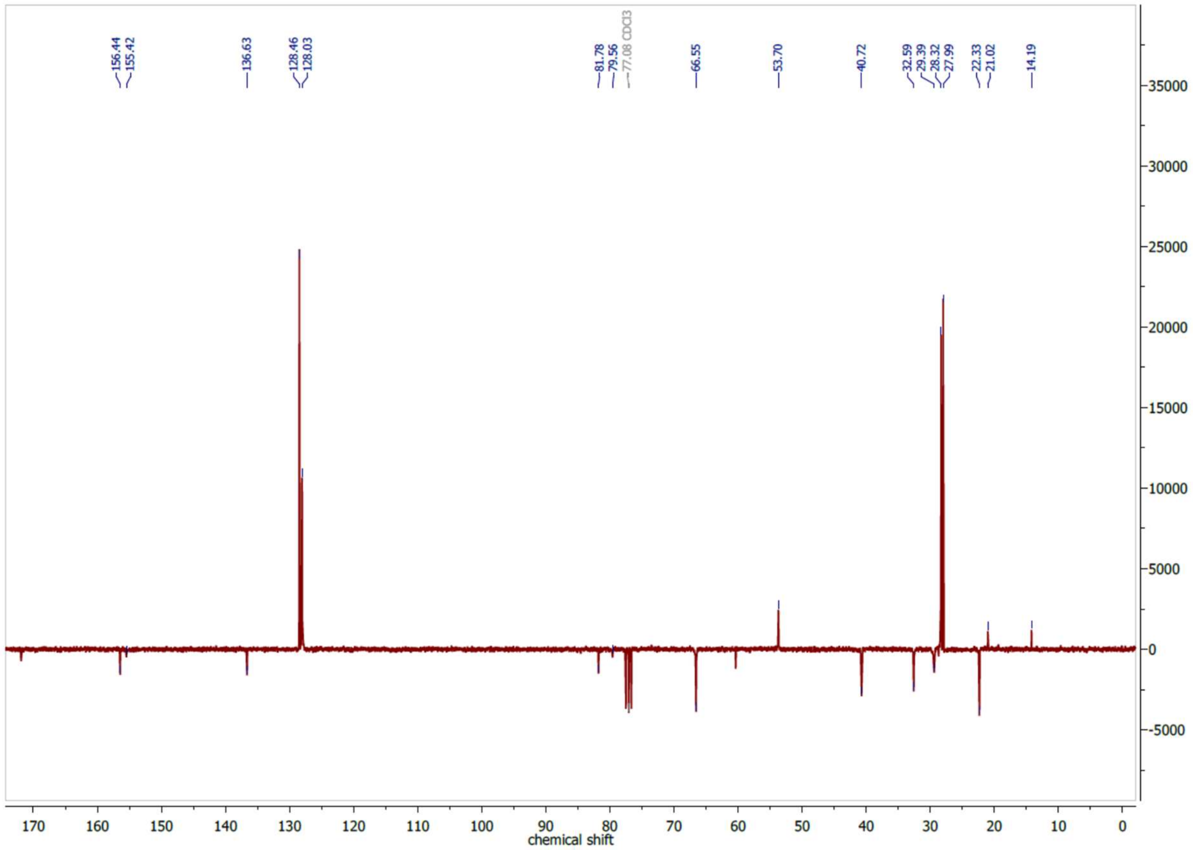

Fig. X39. ^{13}C DEPTQ NMR spectrum of *N*[6]-Cbz *N*[1]-Boc *C*-tBu lysine in $CDCl_3$ at 75 MHz.

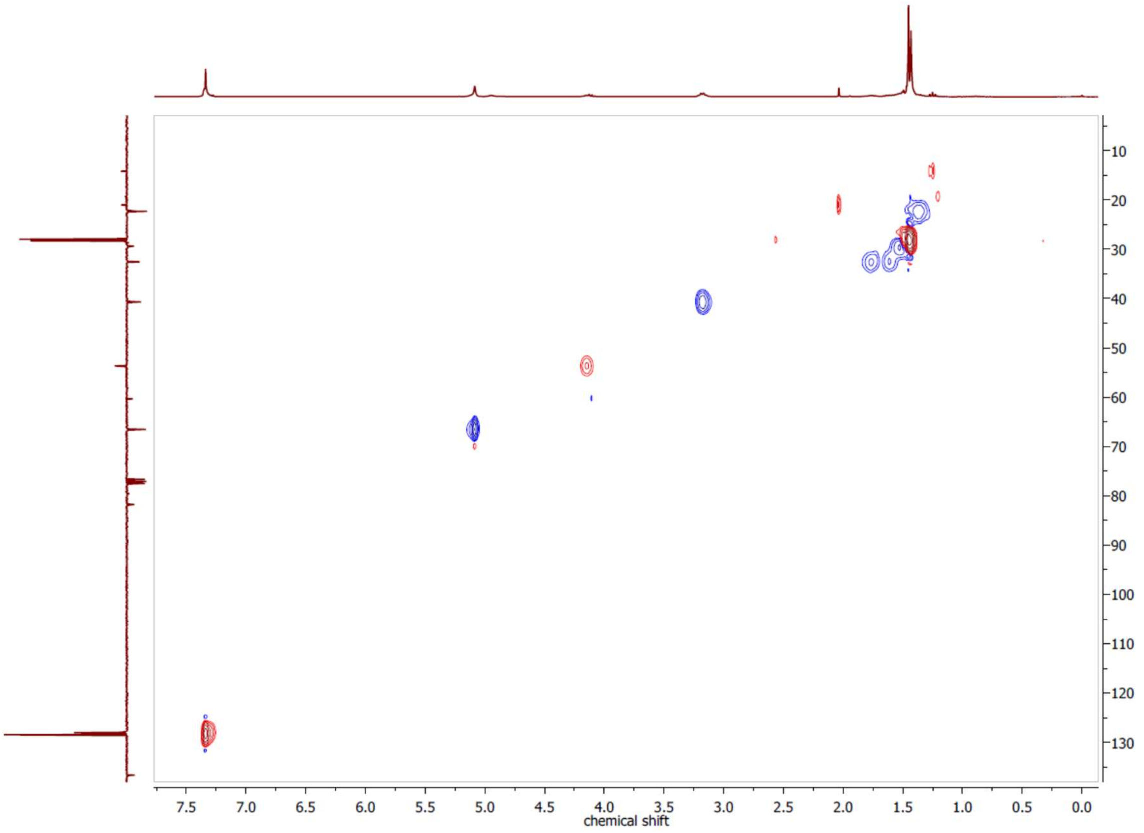

Fig. X40. ^{1}H,^{13}C HSQCed NMR spectrum of *N*[6]-Cbz *N*[1]-Boc *C*-tBu lysine in $CDCl_3$.

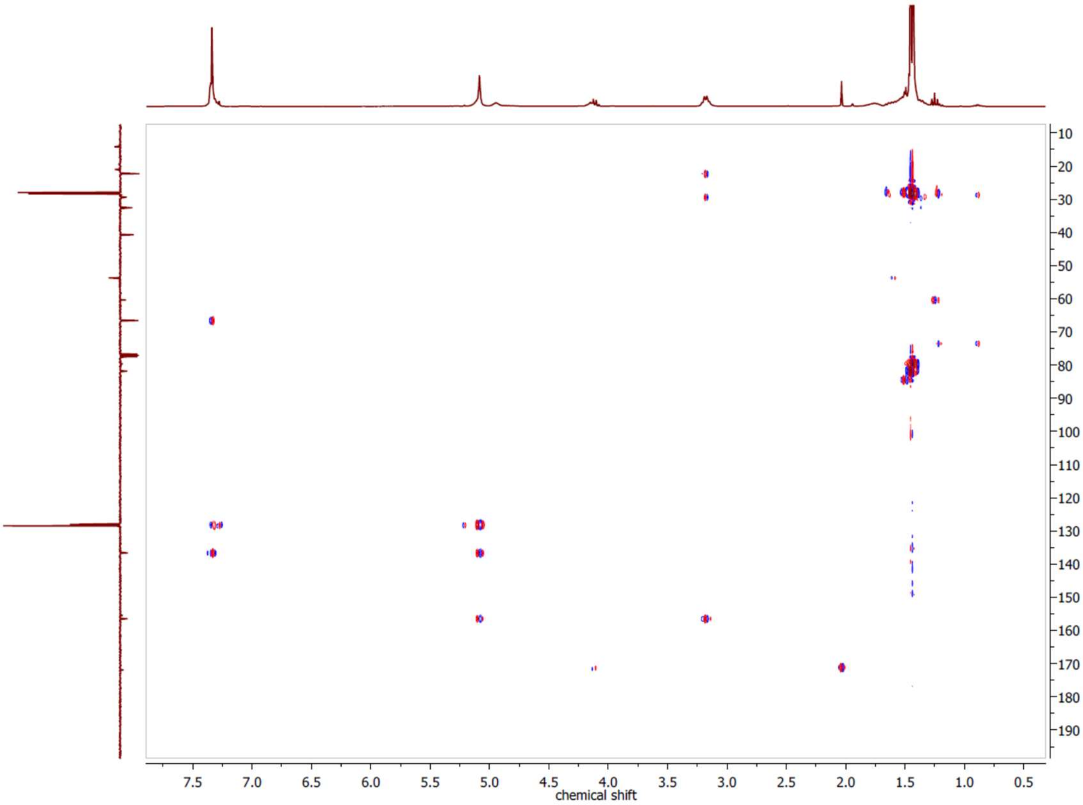

Fig. X41. $^{1}H,^{13}C$ HMBC NMR spectrum of N^{6}-Cbz N^{1}-Boc C-tBu lysine in $CDCl_3$.

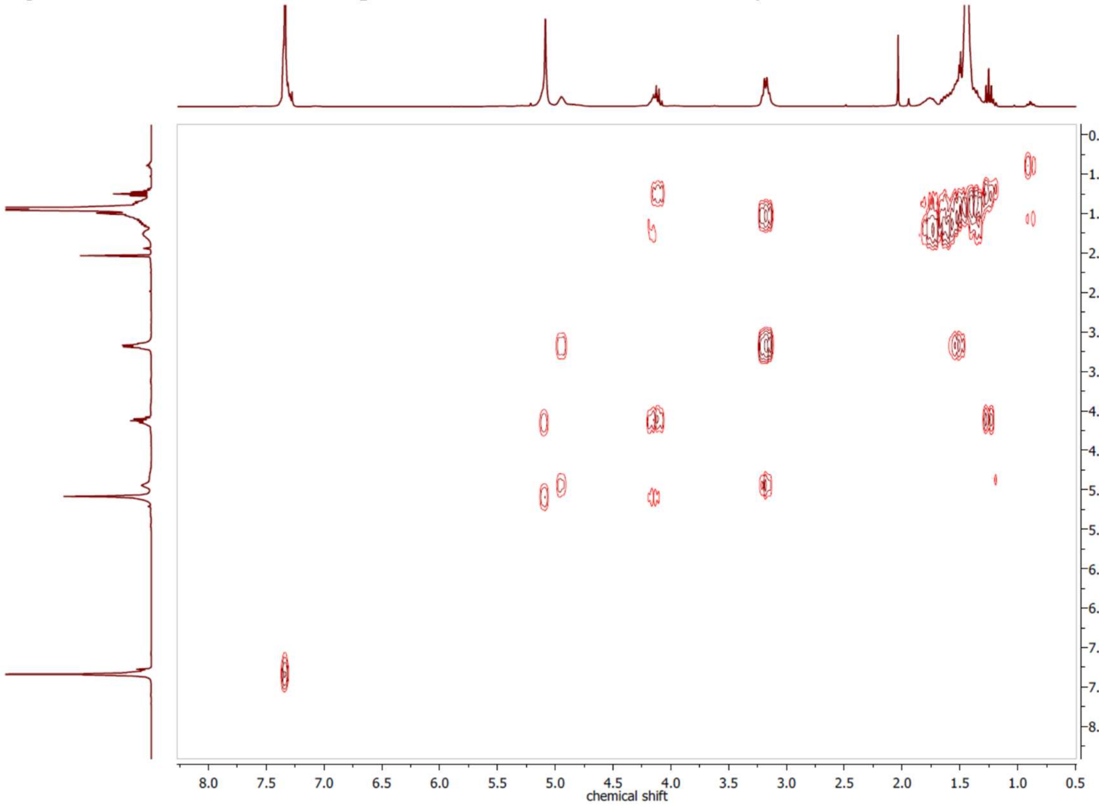

Fig. X42. $^{1}H,^{1}H$ COSY NMR spectrum of N^{6}-Cbz N^{1}-Boc C-tBu lysine in $CDCl_3$.

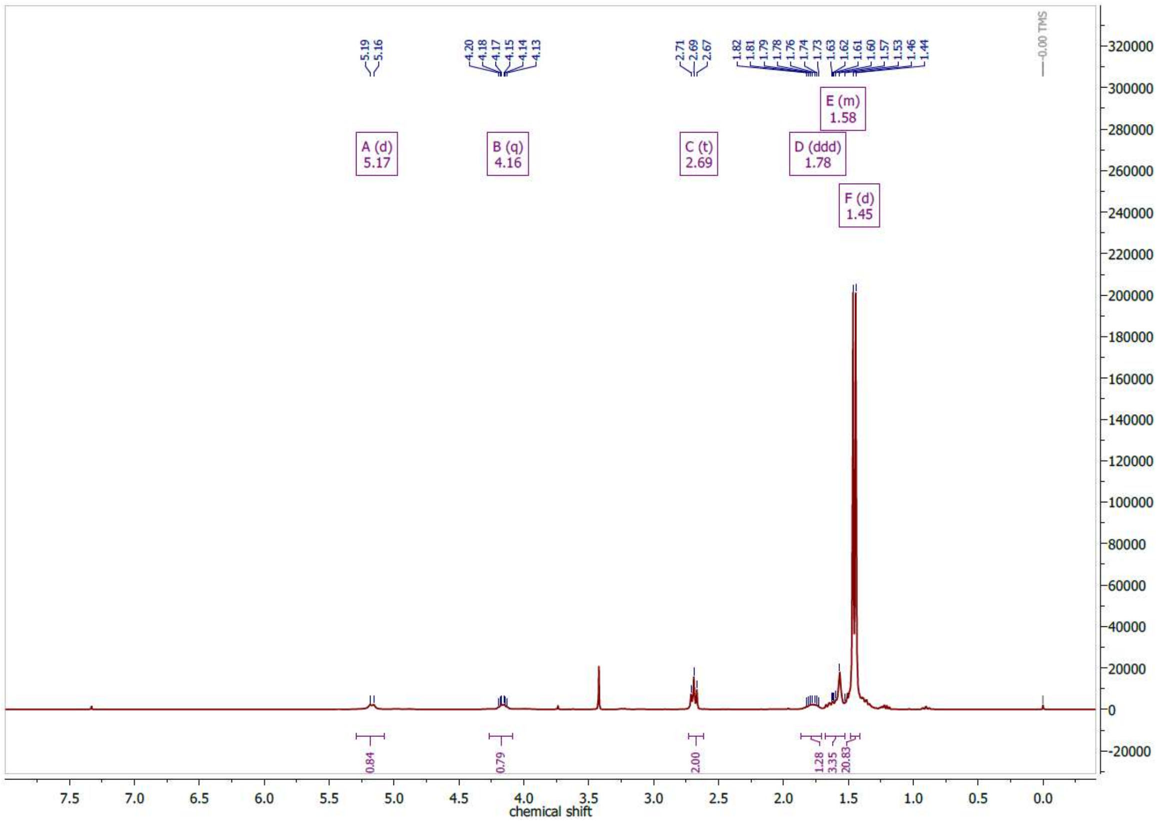

Fig. X43. ^{1}H NMR spectrum of *N*[1]-Boc *C*-tBu lysine in $CDCl_3$ at 300 MHz.

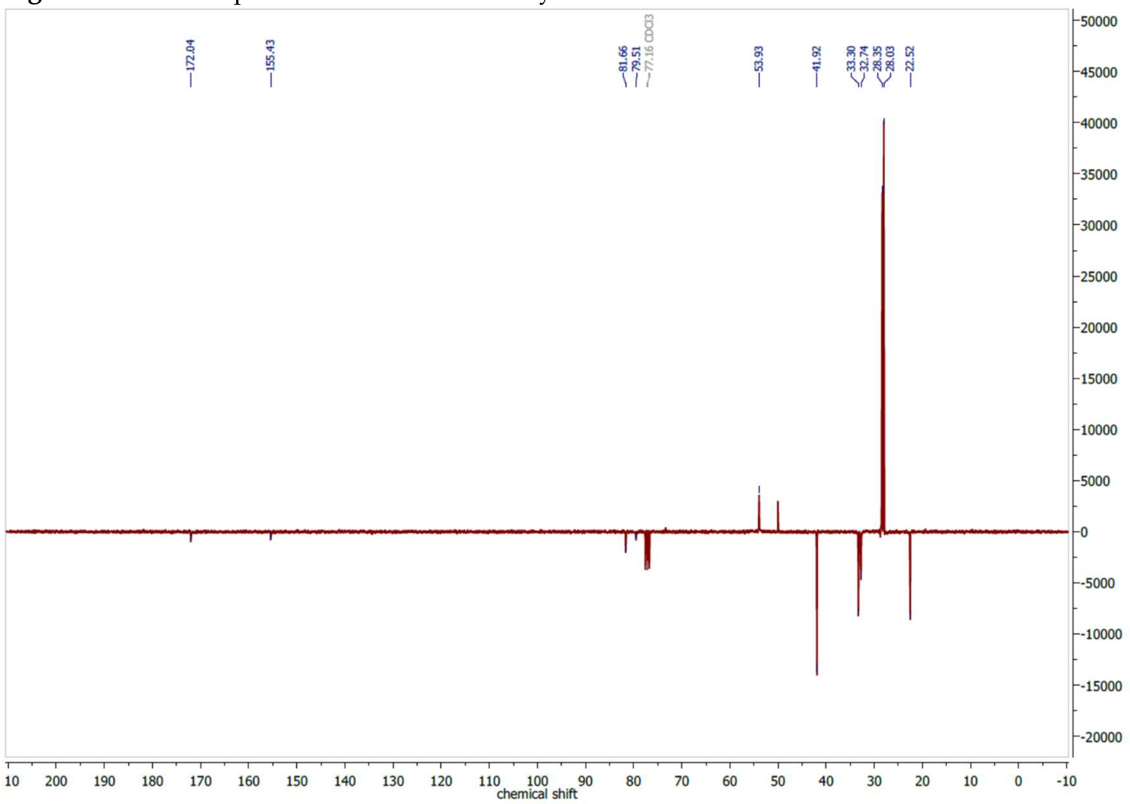

Fig. X44. ^{13}C DEPTQ NMR spectrum of *N*[1]-Boc *C*-tBu lysine in CDCl at 75 MHz.

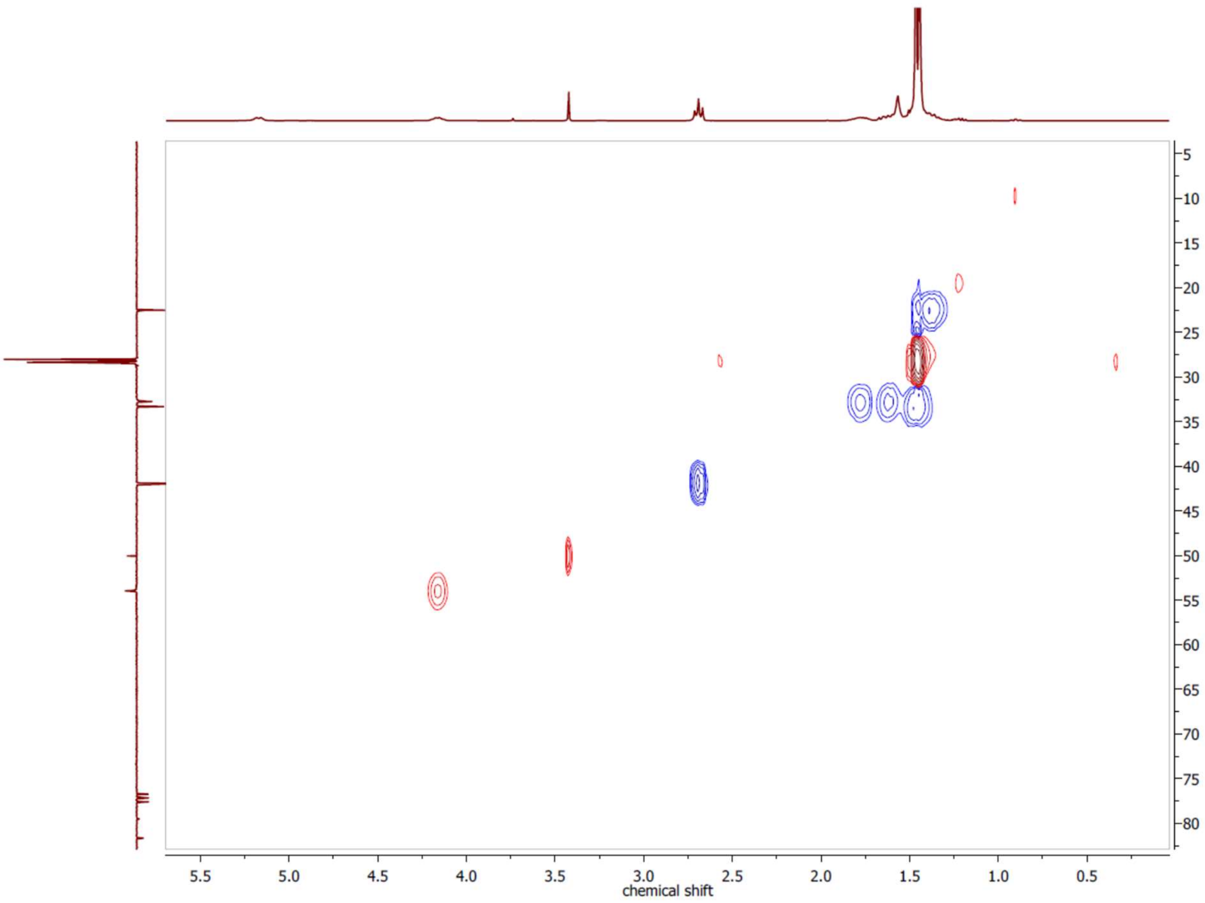

Fig. X45. $^{1}H,^{13}C$ HSQCed NMR spectrum of *N*1-Boc *C*-tBu lysine in $CDCl_3$.

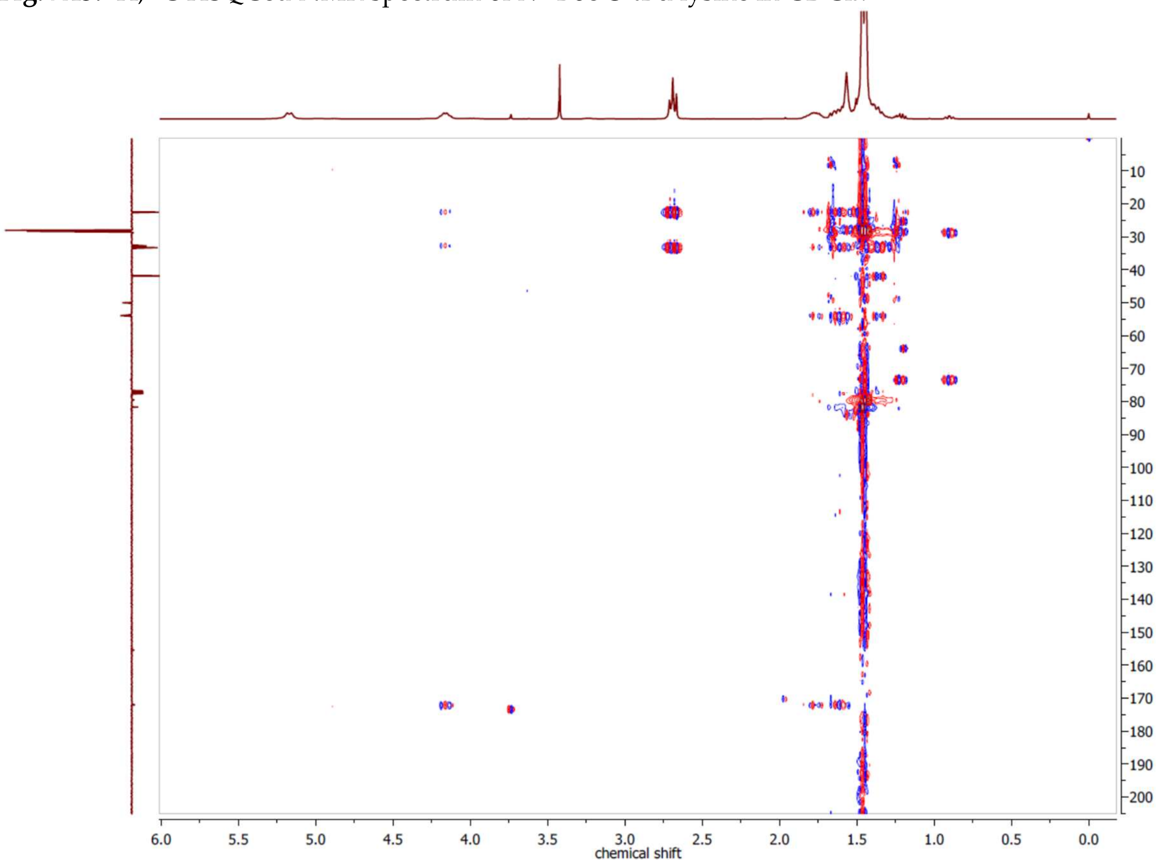

Fig. X46. $^{1}H,^{13}C$ HMBC NMR spectrum of *N*1-Boc *C*-tBu lysine in $CDCl_3$.

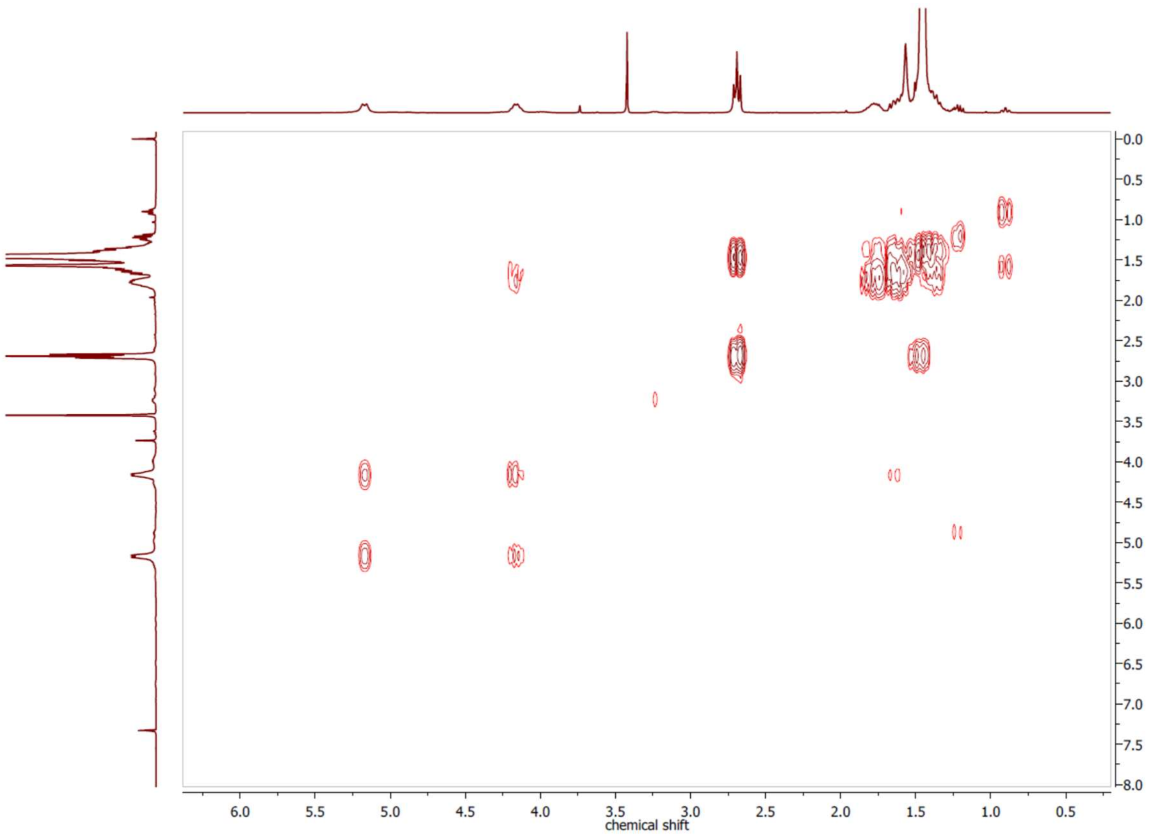

Fig. X47. ^{1}H,^{1}H COSY NMR spectrum of *N*[1]-Boc *C*-tBu lysine in $CDCl_3$.

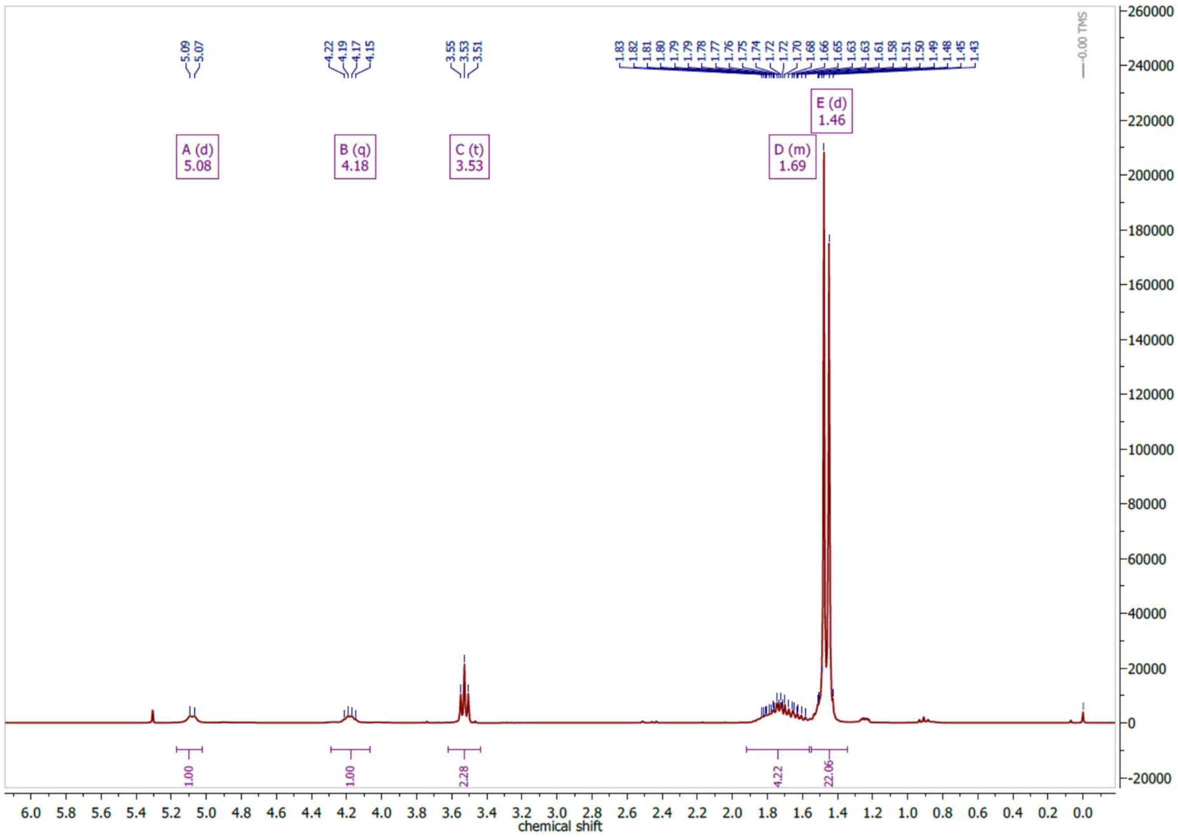

Fig. X48. ^{1}H NMR spectrum of ITCLp in $CDCl_3$ at 300 MHz.

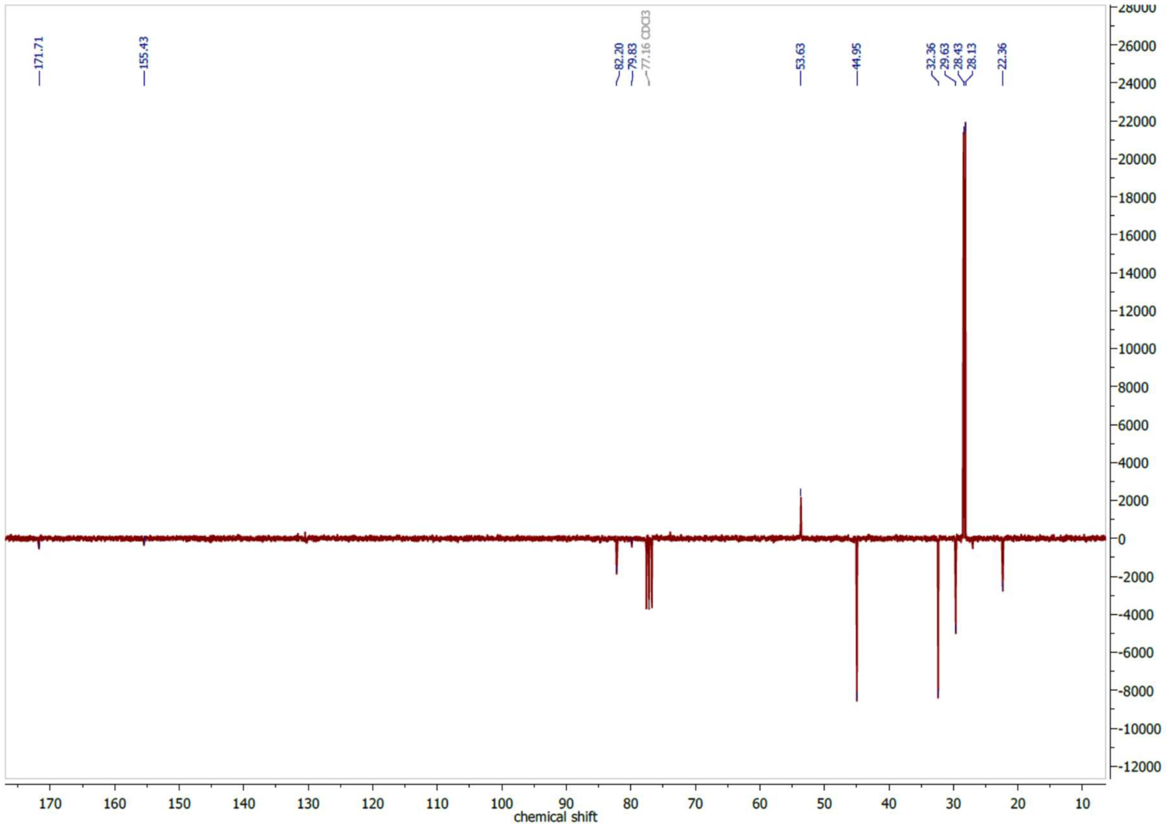

Fig. X49. ^{13}C DEPTQ NMR spectrum of ITCLp in $CDCl_3$ at 75 MHz.

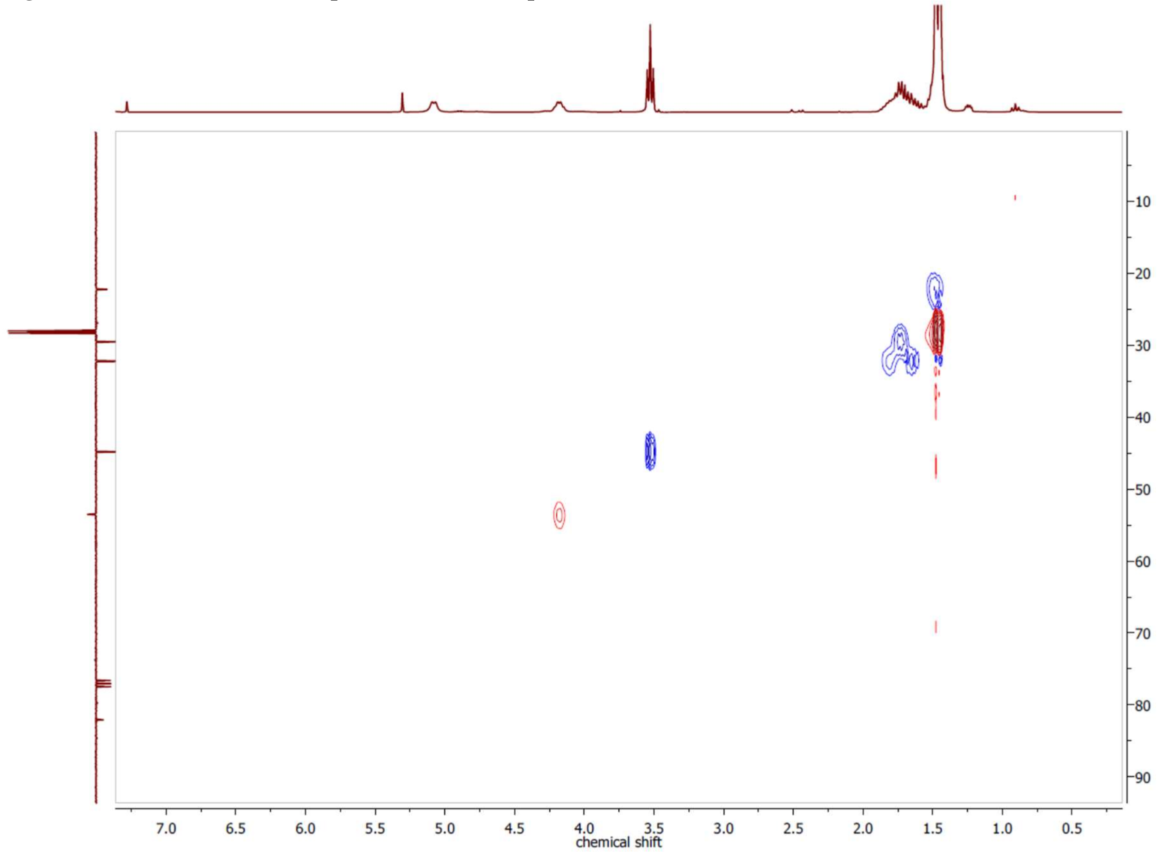

Fig. X50. $^{1}H,^{13}C$ HSQCed NMR spectrum of ITCLp in $CDCl_3$.

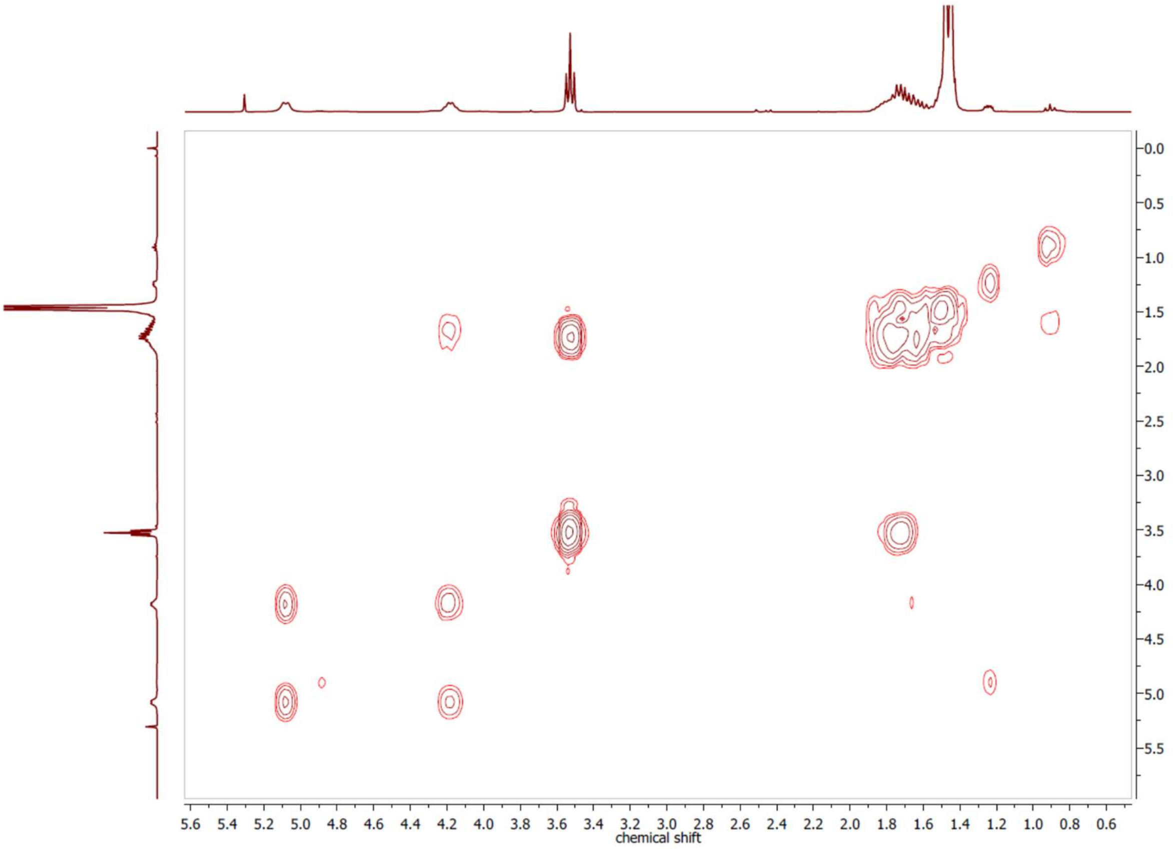

Fig. X51. ^{1}H,^{1}H COSY NMR spectrum of ITCLp in $CDCl_3$.

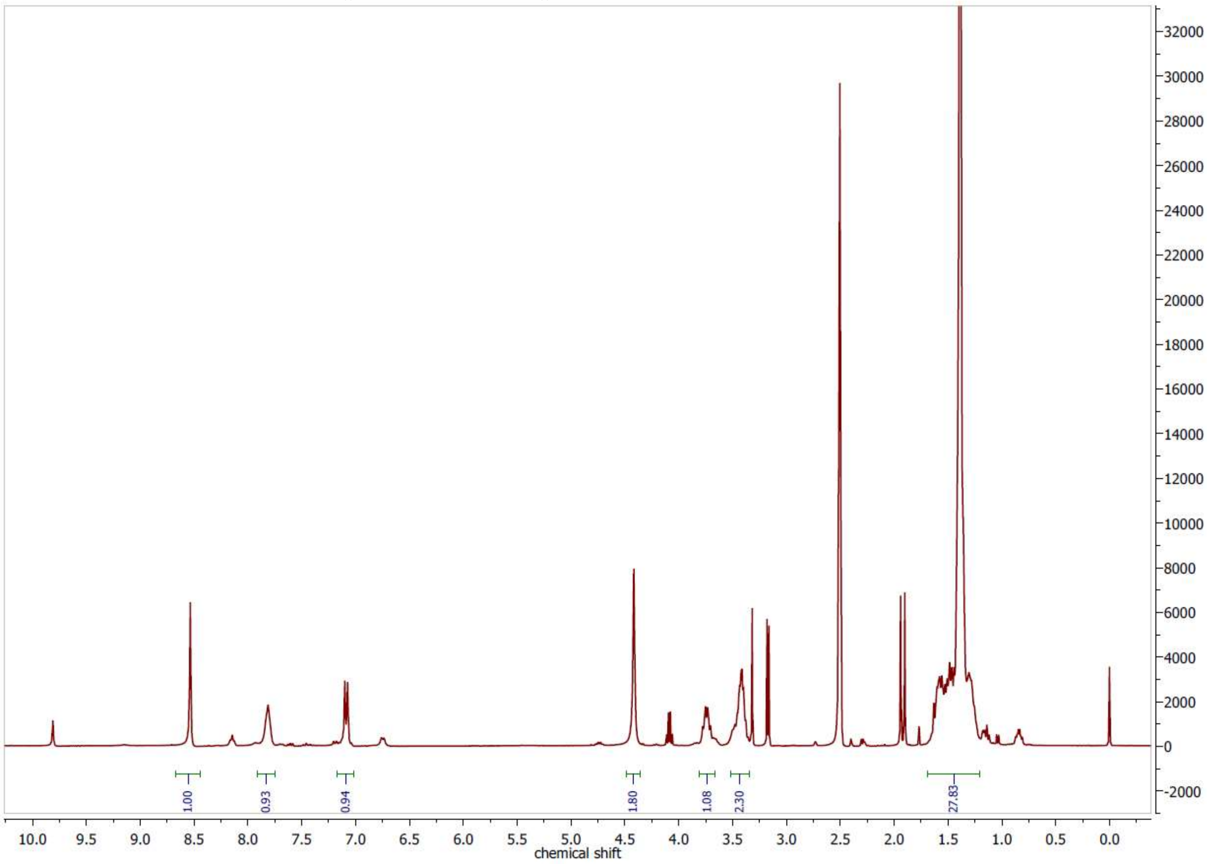

Fig. X52. ^{1}H NMR spectrum of TSCLp in $CDCl_3$ at 300 MHz.

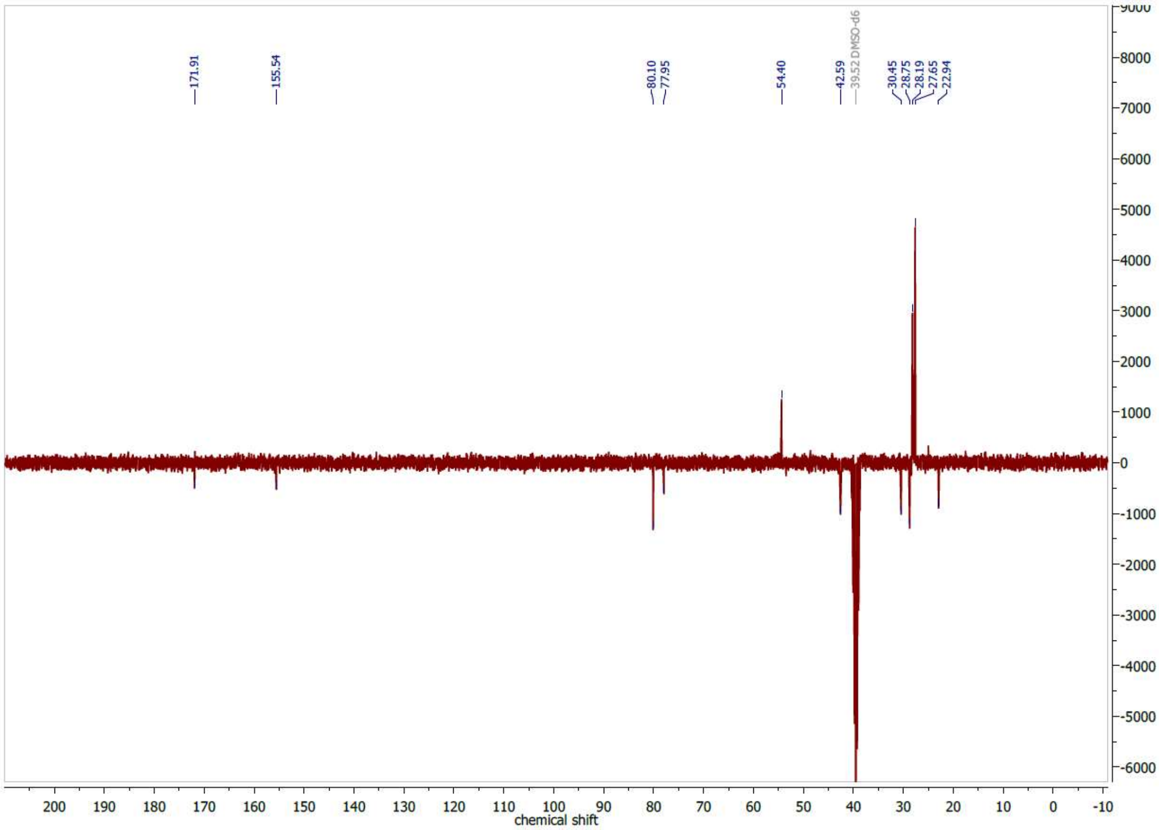

Fig. X53. ^{13}C DEPTQ NMR spectrum of TSCLp in $CDCl_3$ at 75 MHz.

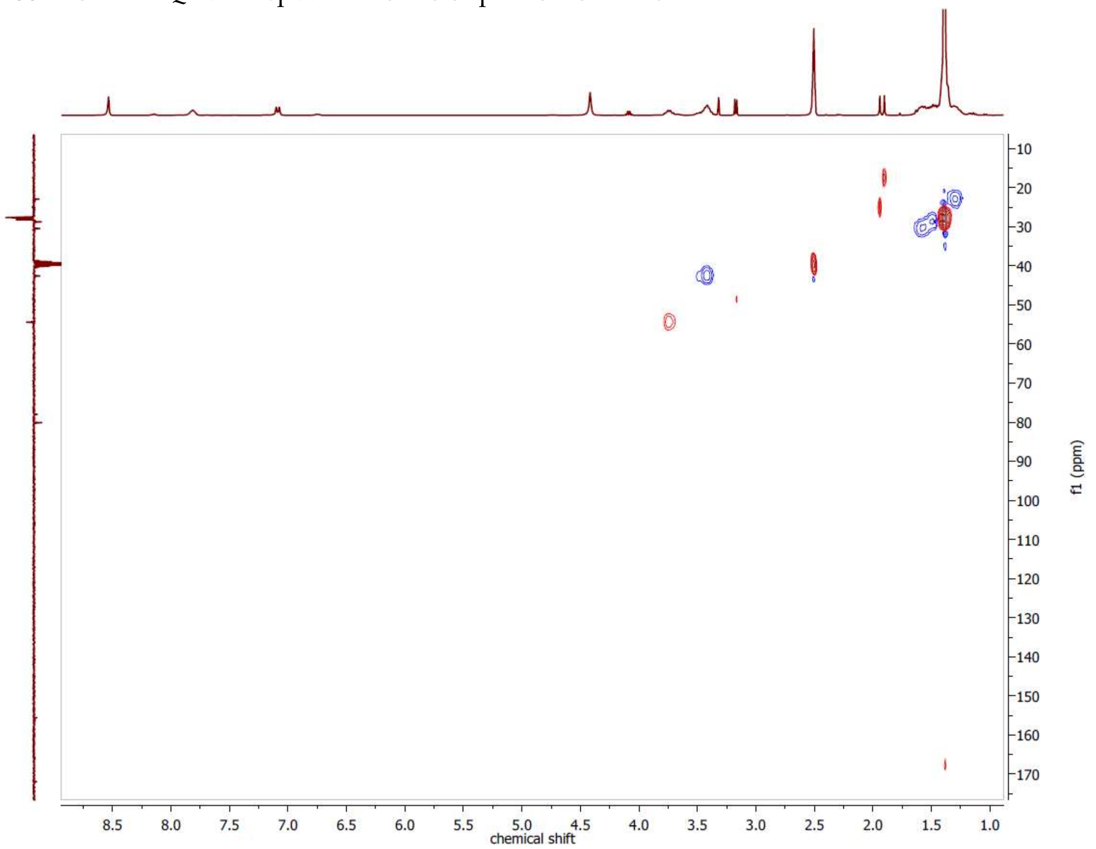

Fig. X54. $^{1}H,^{13}C$ HSQCed NMR spectrum of TSCLp in $CDCl_3$.

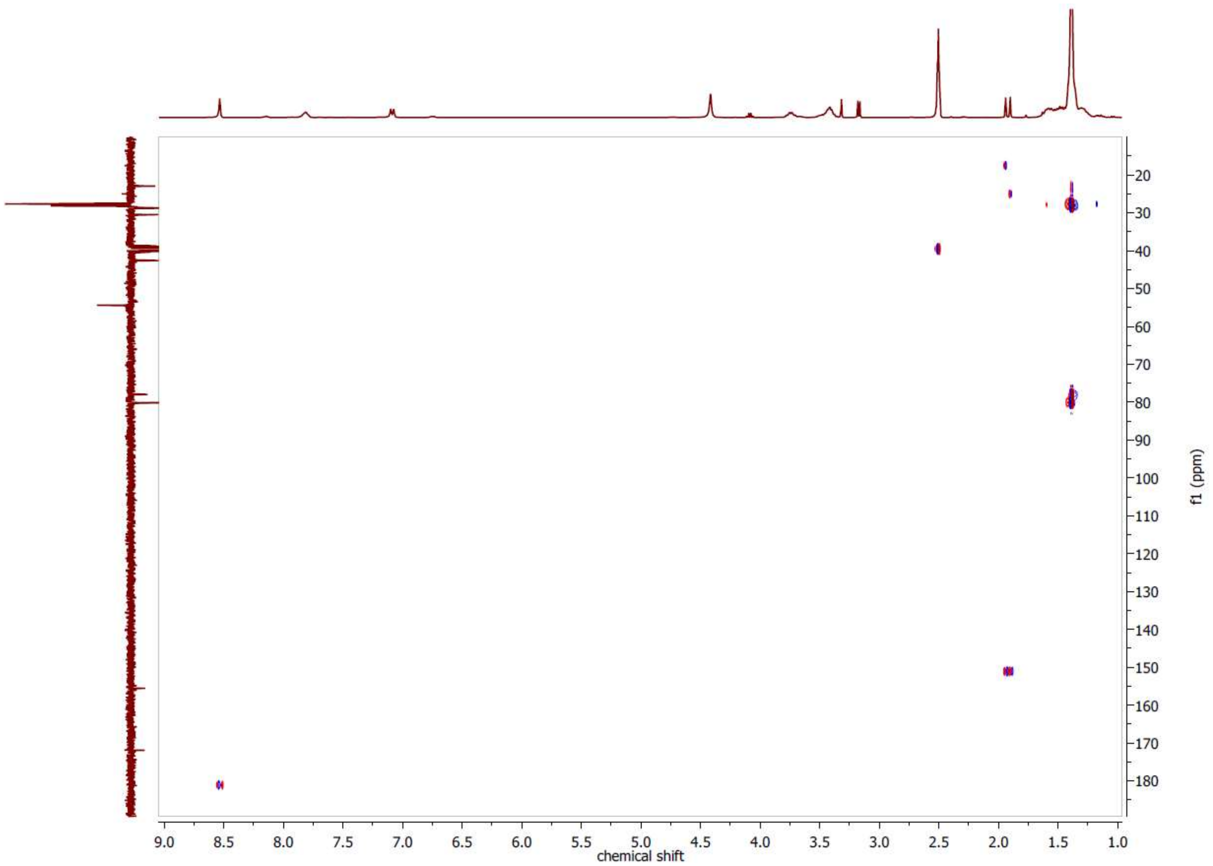

Fig. X55. $^{1}H,^{13}C$ HMBC NMR spectrum of TSCLp in $CDCl_3$.

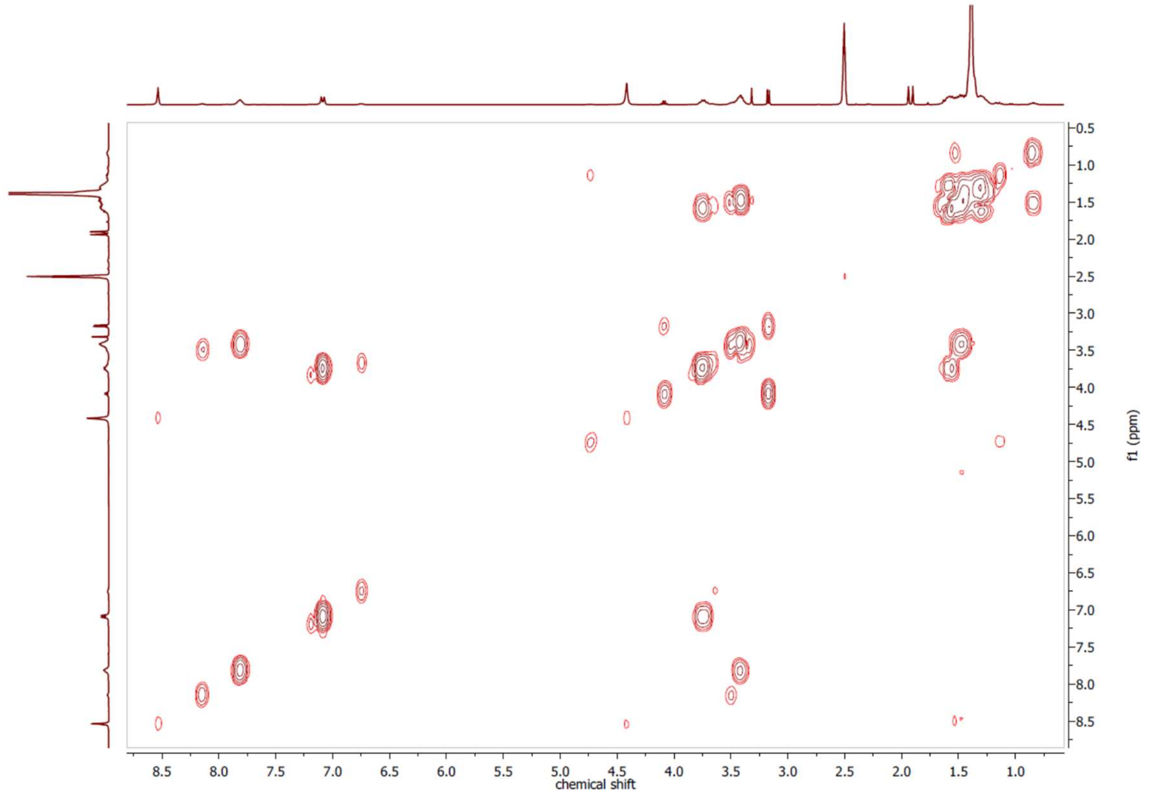

Fig. X56. $^{1}H,^{1}H$ COSY NMR spectrum of TSCLp in $CDCl_3$.

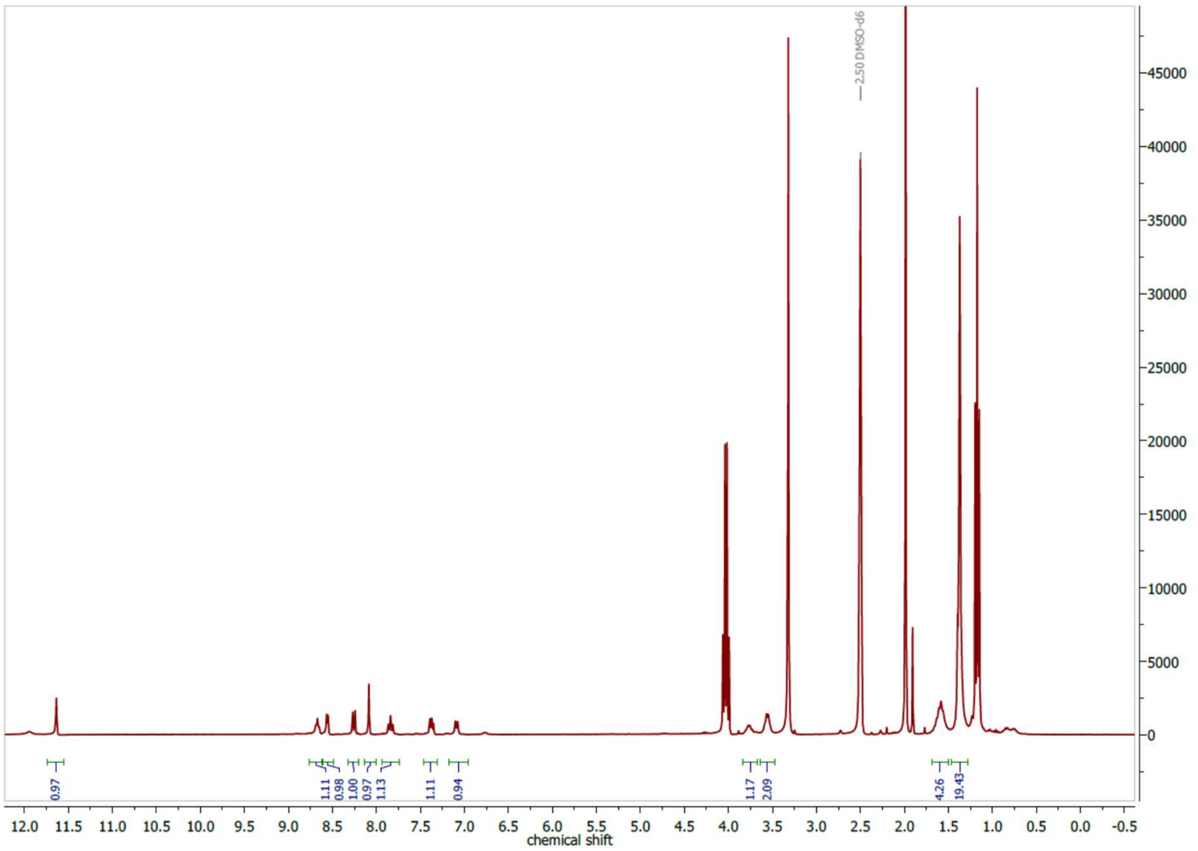

protoligands:

Fig. X57. ^{1}H NMR spectrum of HfpyTSCLp in DMSO-d_6 at 300 MHz.

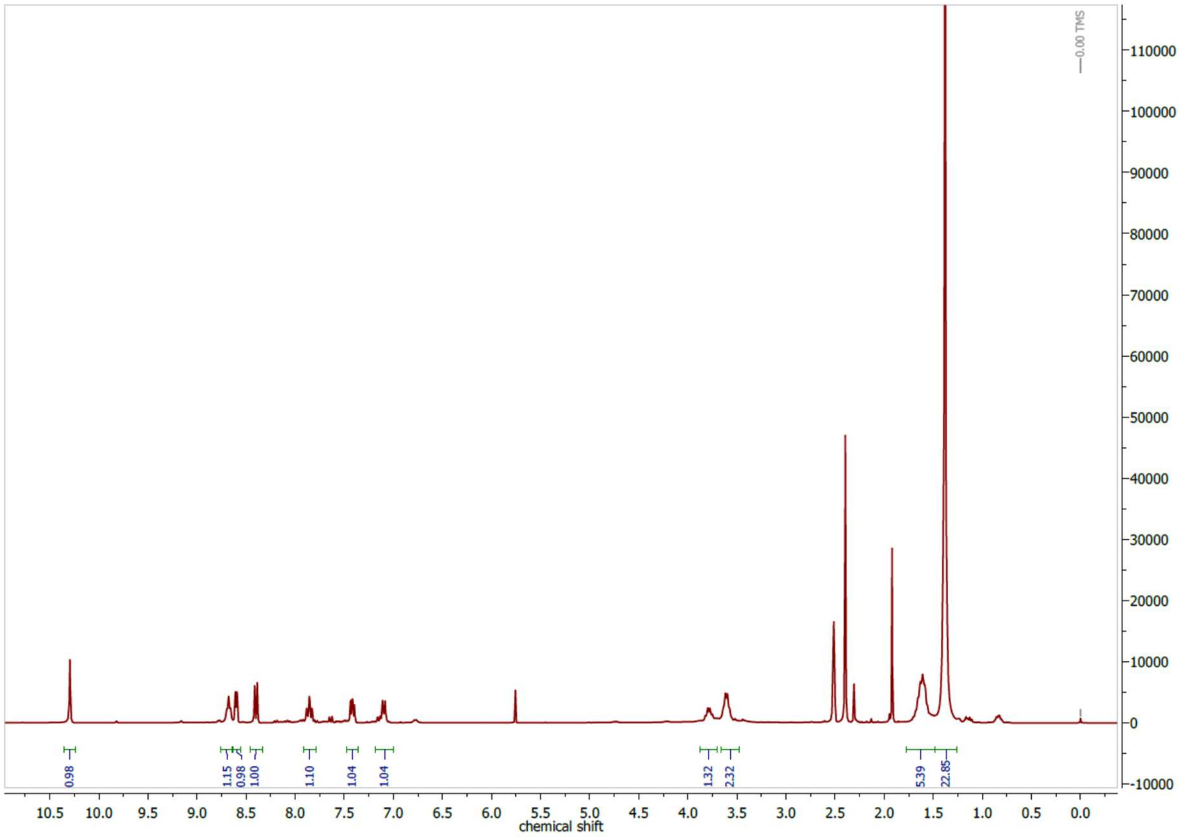

Fig. X58. ^{1}H NMR spectrum of HapyTSCLp in DMSO-d_6 at 300 MHz.

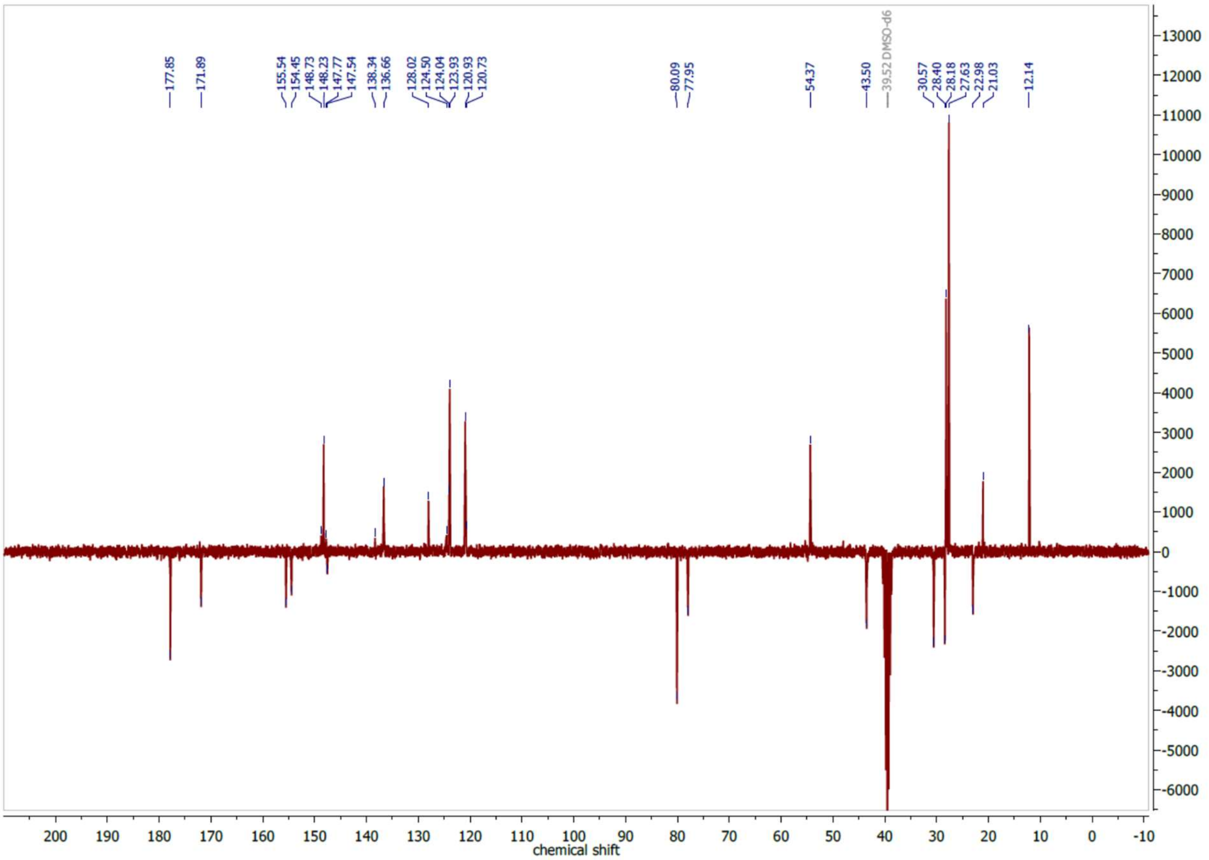

Fig. X59. ^{13}C DEPTQ NMR spectrum of HapyTSCLp in DMSO-d_6 at 75 MHz.

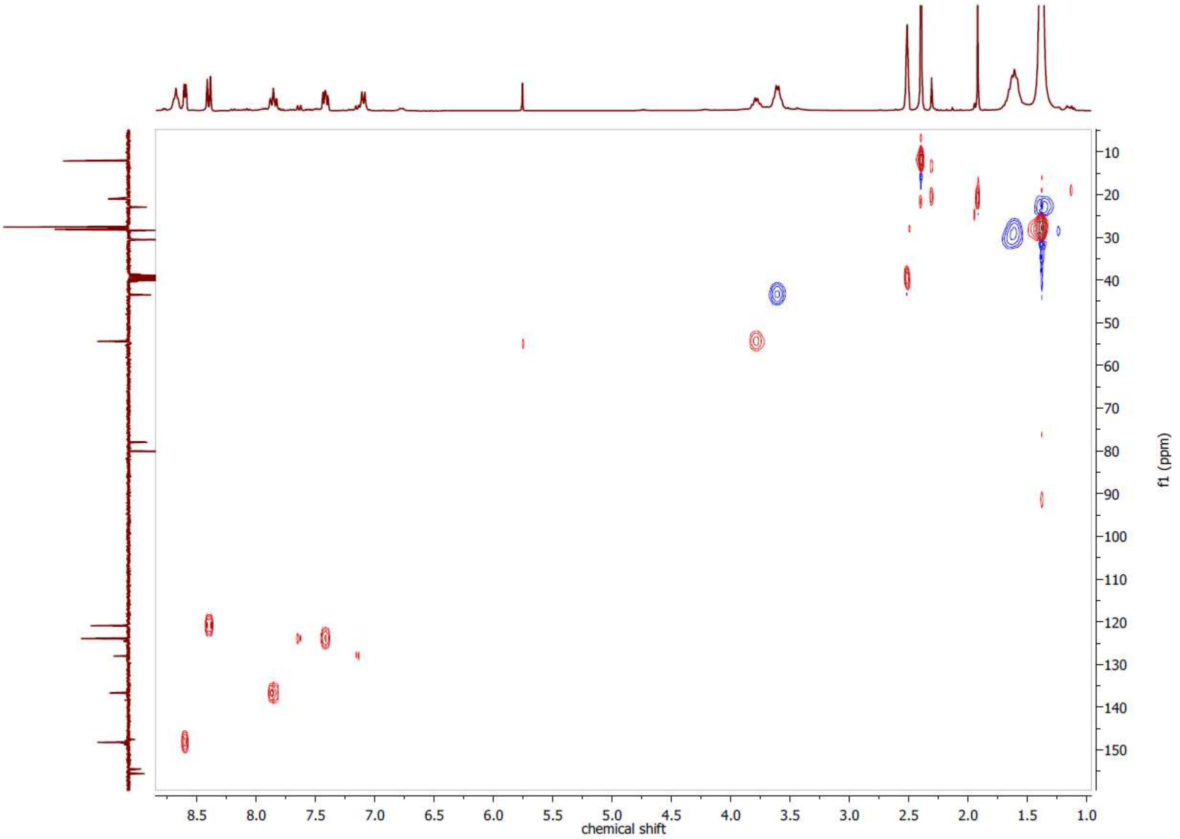

Fig. X60. ^{1}H,^{13}C HSQCed NMR spectrum of HapyTSCLp in DMSO-d_6.

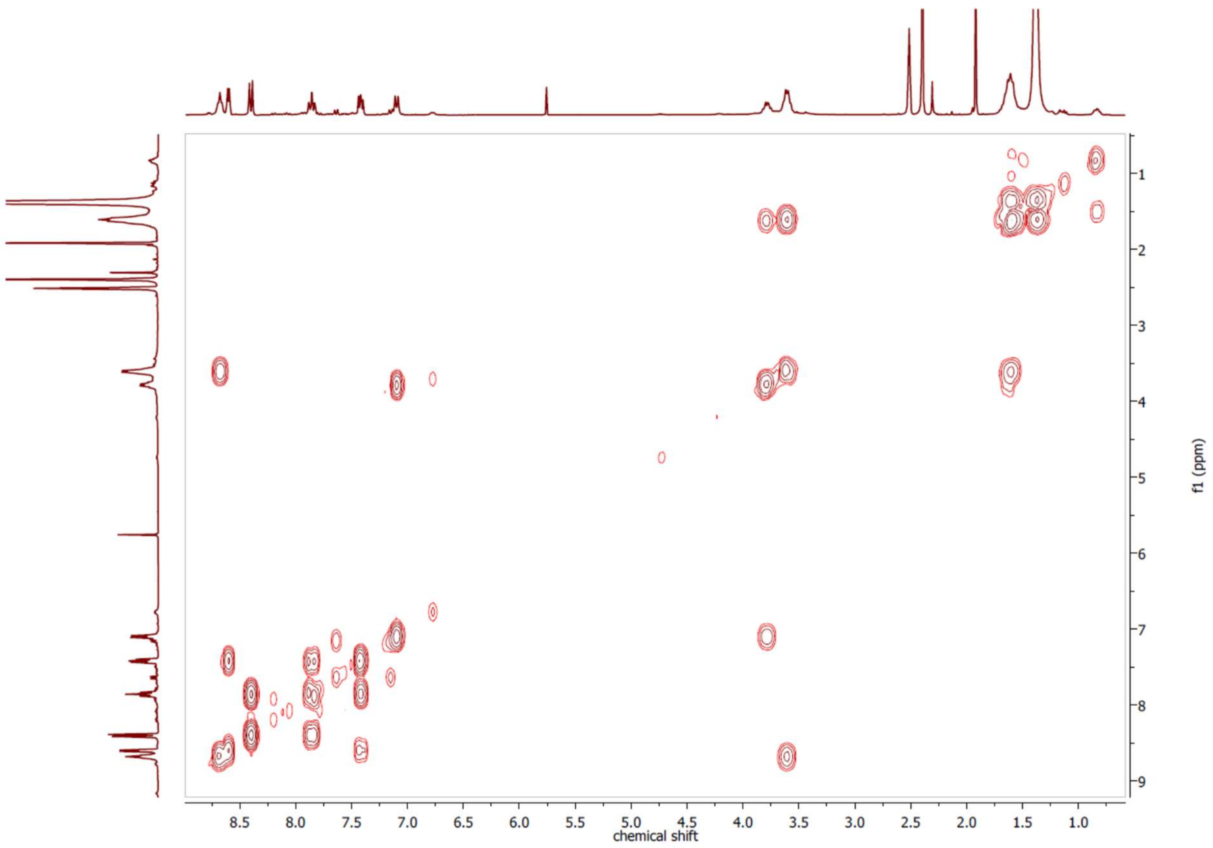

Fig. X61. ^{1}H,^{1}H COSY NMR spectrum of HapyTSCLp in DMSO-d_6.

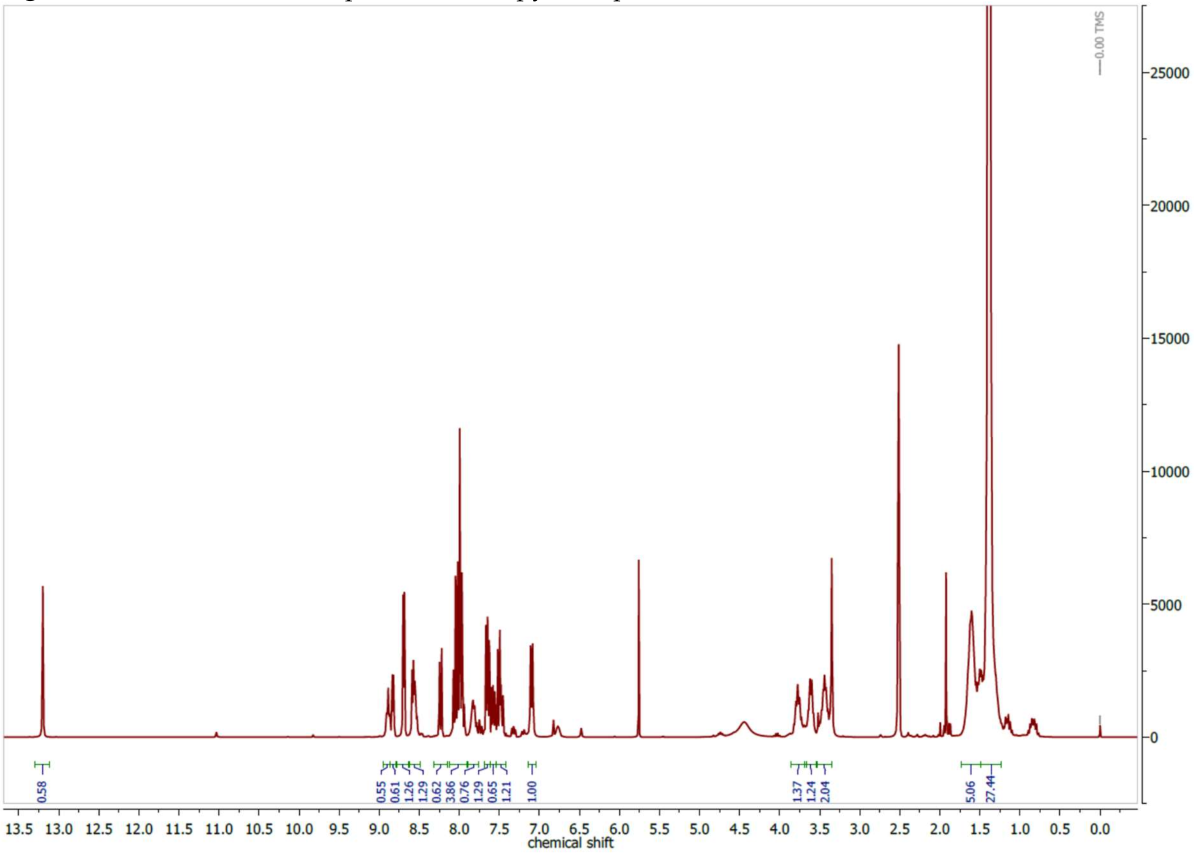

Fig. X62. ^{1}H NMR spectrum of HdpyTSCLp in DMSO-d_6 at 300 MHz.

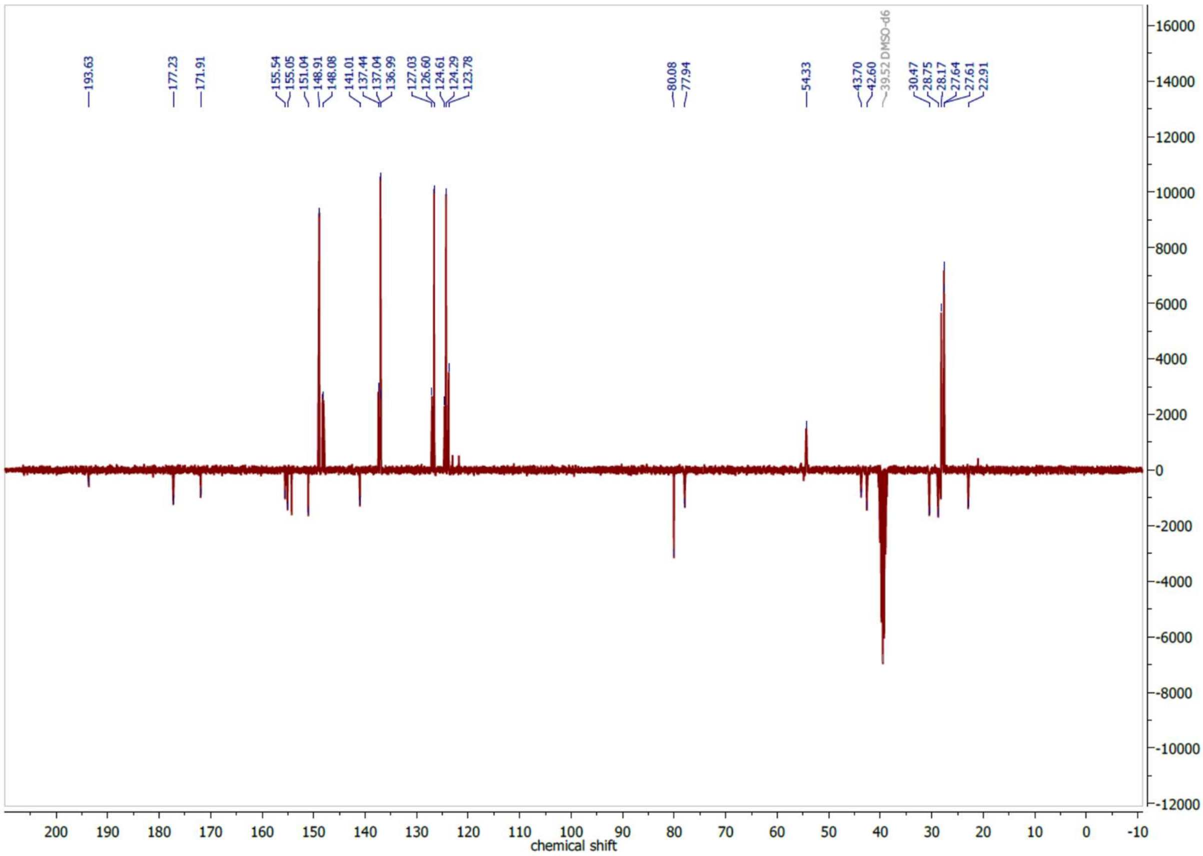

Fig. X63. ^{13}C DEPTQ NMR spectrum of HdpyTSCLp in DMSO-d_6 at 75 MHz.

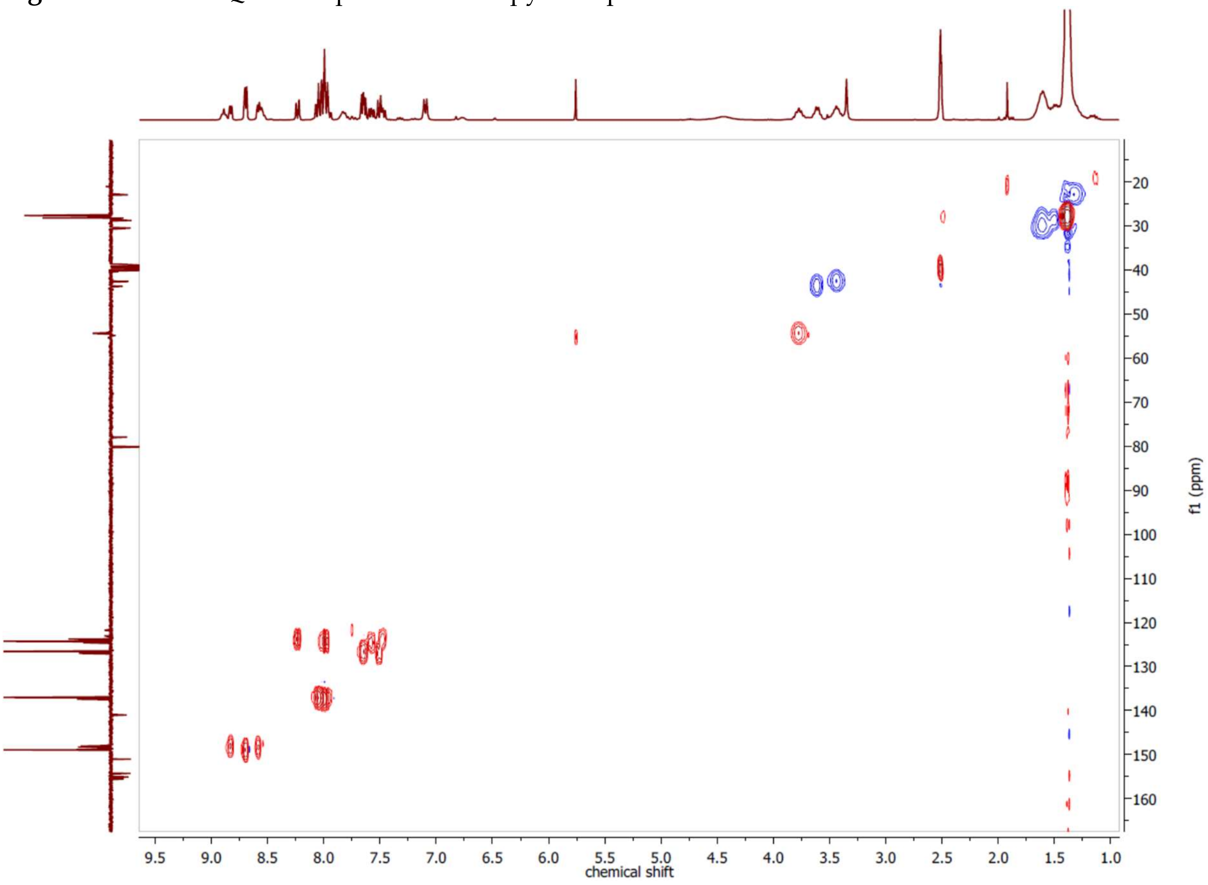

Fig. X64. ^{1}H,^{13}C HSQCed NMR spectrum of HdpyTSCLp in DMSO-d_6.

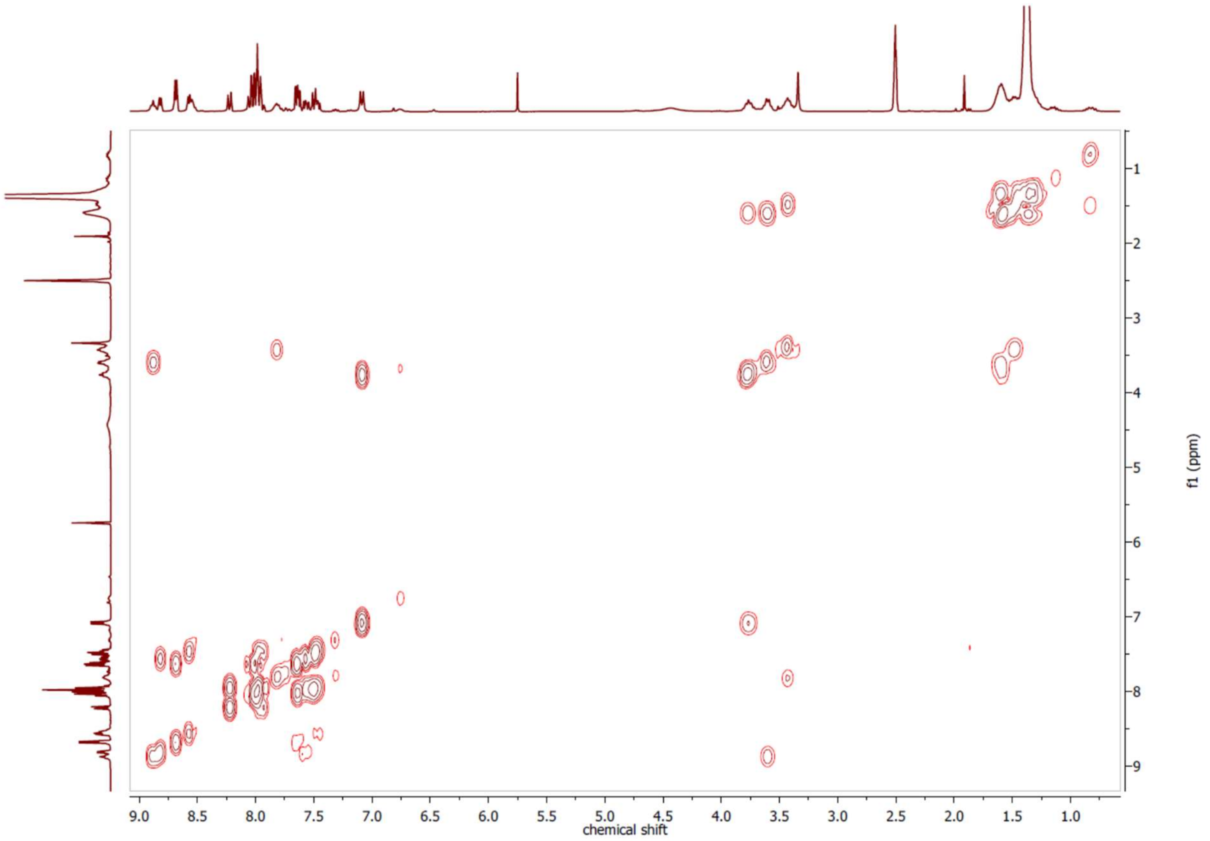

Fig. X65. ^{1}H,^{1}H COSY NMR spectrum of HdpyTSCLp in DMSO-d_6.

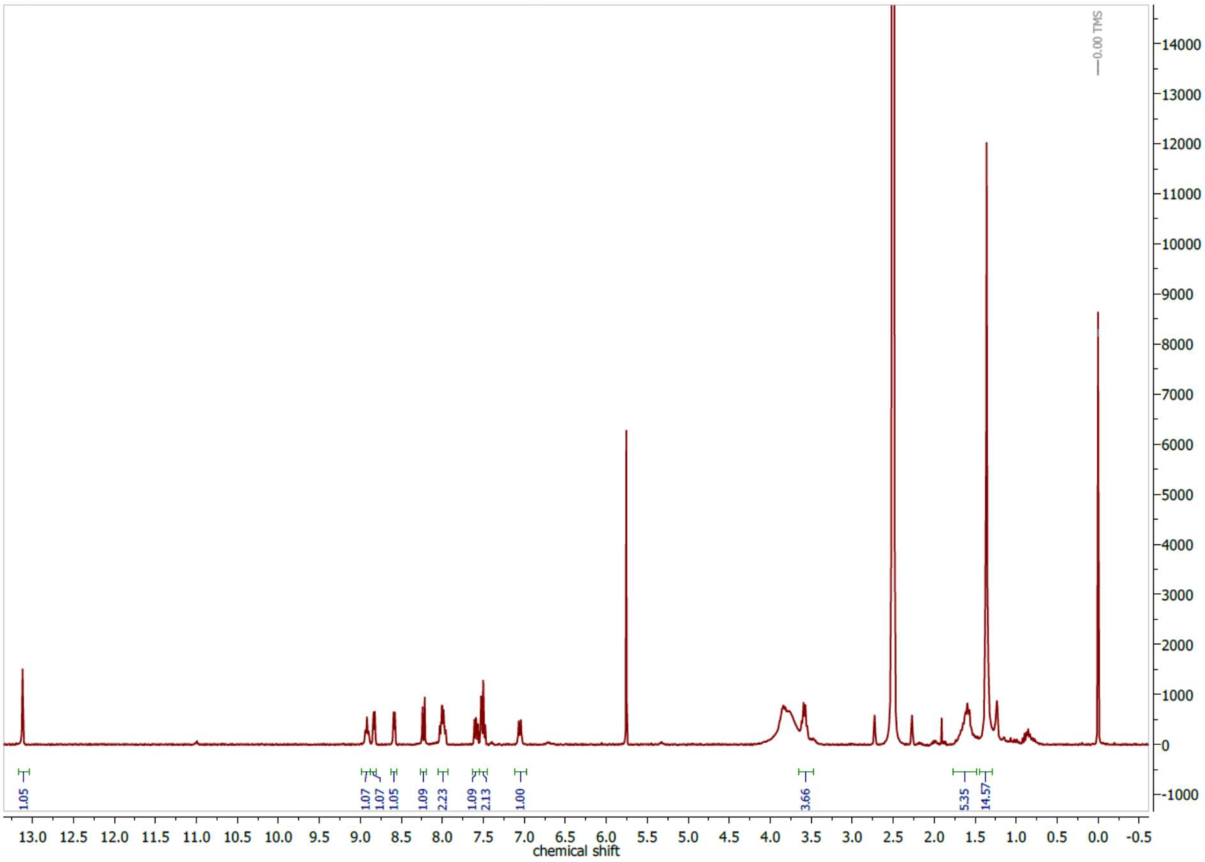

Fig. X66. ^{1}H NMR spectrum of HdpyTSCLBoc in DMSO-d_6 at 300 MHz.

Complexes

[Pt(RpyTSCmB)X] Series

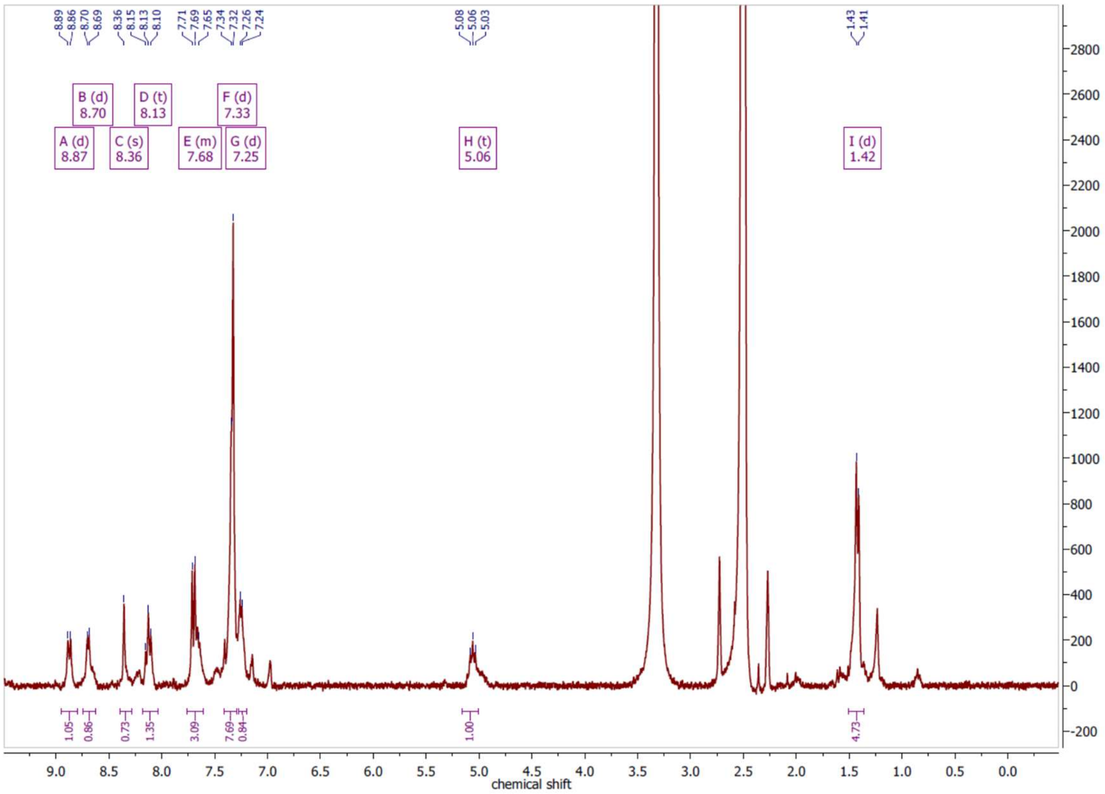

Fig. X67. ^{1}H NMR spectrum of [Pt(fpyTSCmB)Cl] in DMSO-d_6 at 300 MHz.

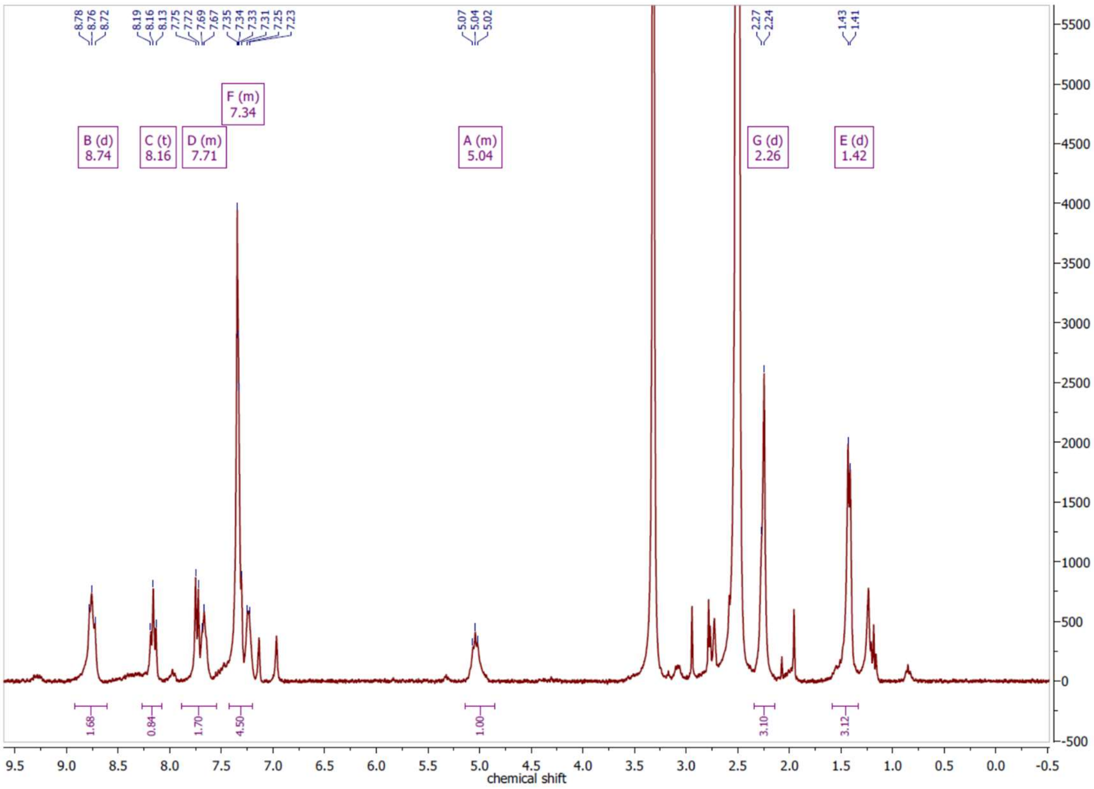

Fig. S68. ^{1}H NMR spectrum of [Pt(apyTSCmB)Cl] in DMSO-d_6 at 300 MHz.

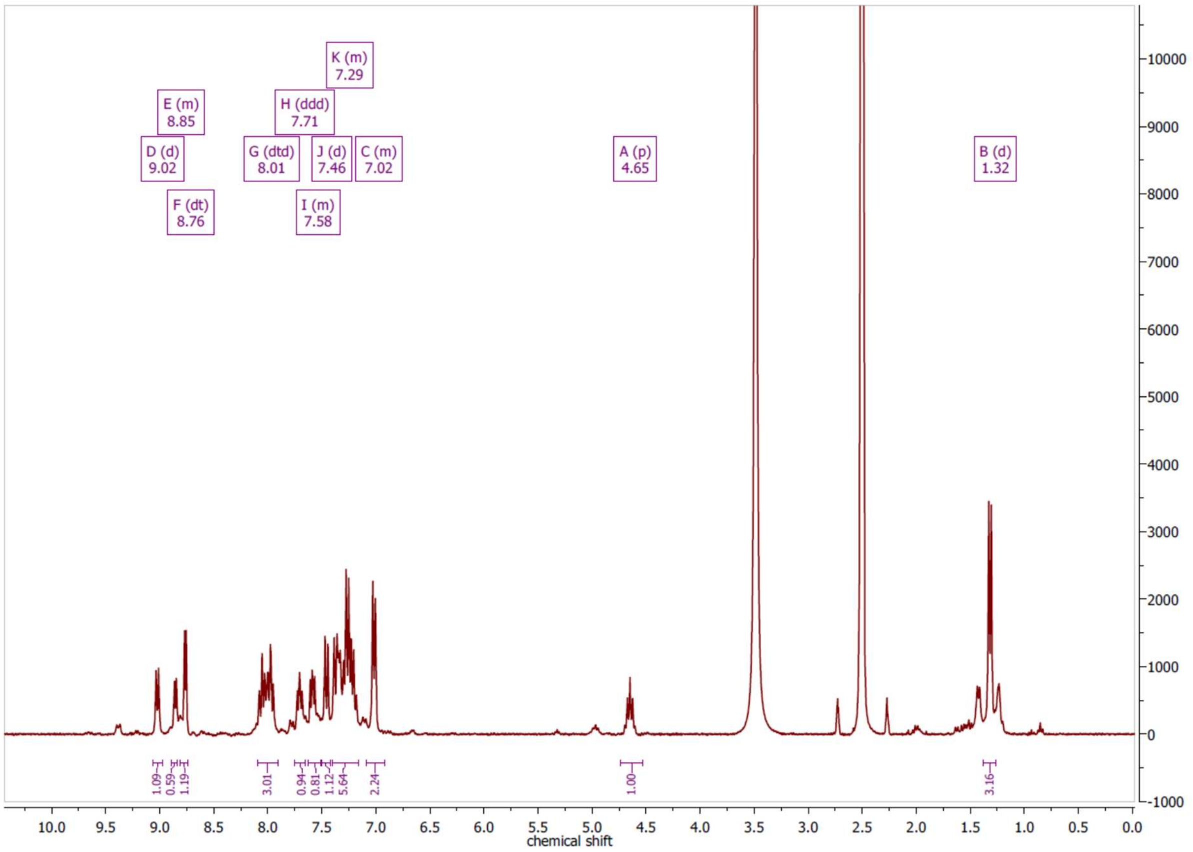

Fig. X69. ^{1}H NMR spectrum of [Pt(dpyTSCmB)Cl] in DMSO-d_6 at 300 MHz.

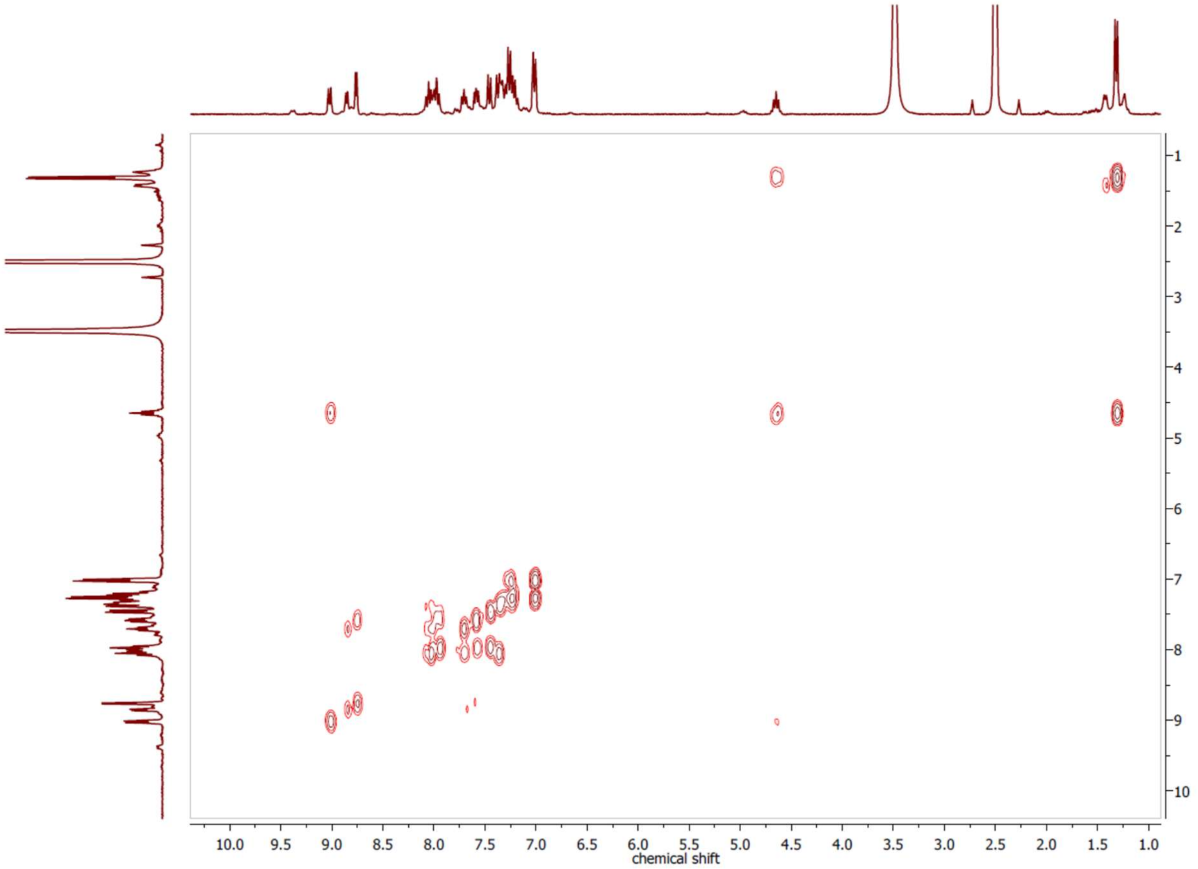

Fig. X70. $^{1}H,^{1}H$ COSY NMR spectrum of [Pt(dpyTSCmB)Cl] in DMSO-d_6.

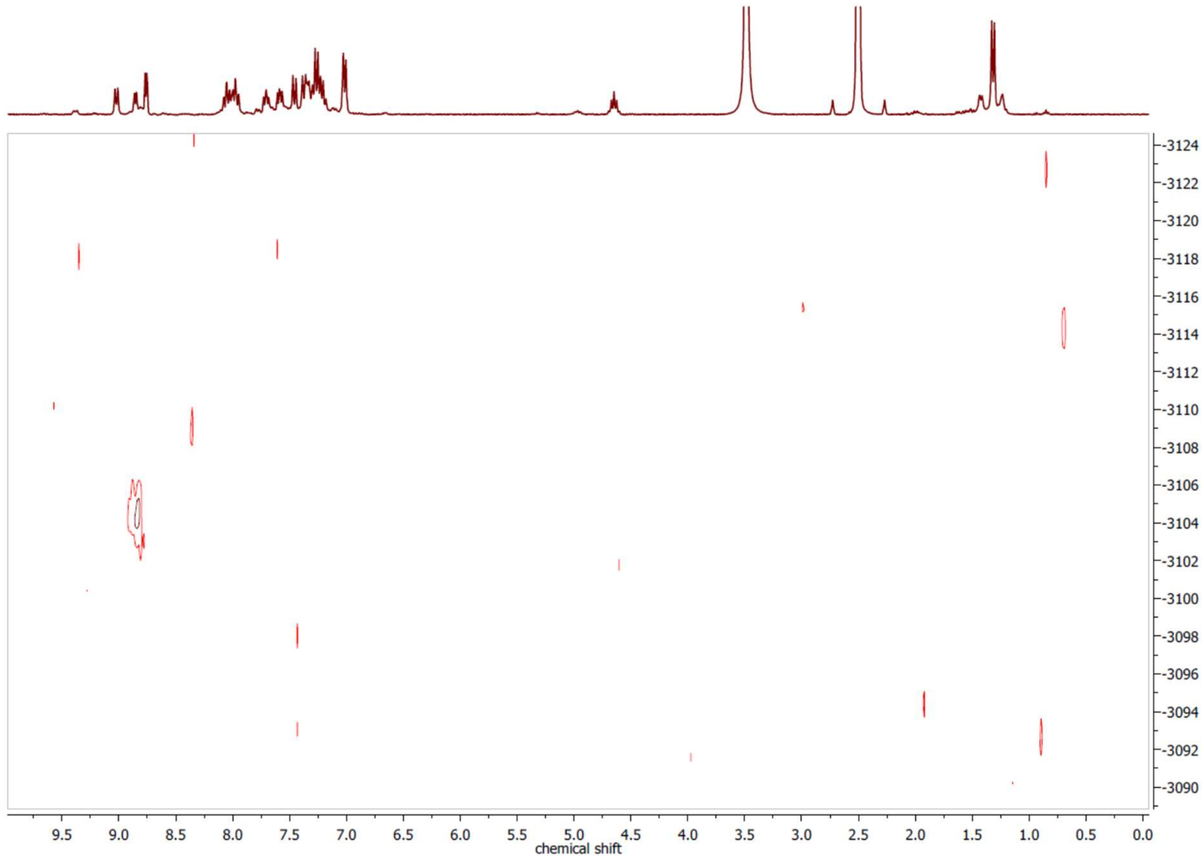

Fig. X71. $^{1}H,^{195}Pt$ HMBC NMR spectrum of [Pt(dpyTSCmB)Cl] in DMSO-d_6.

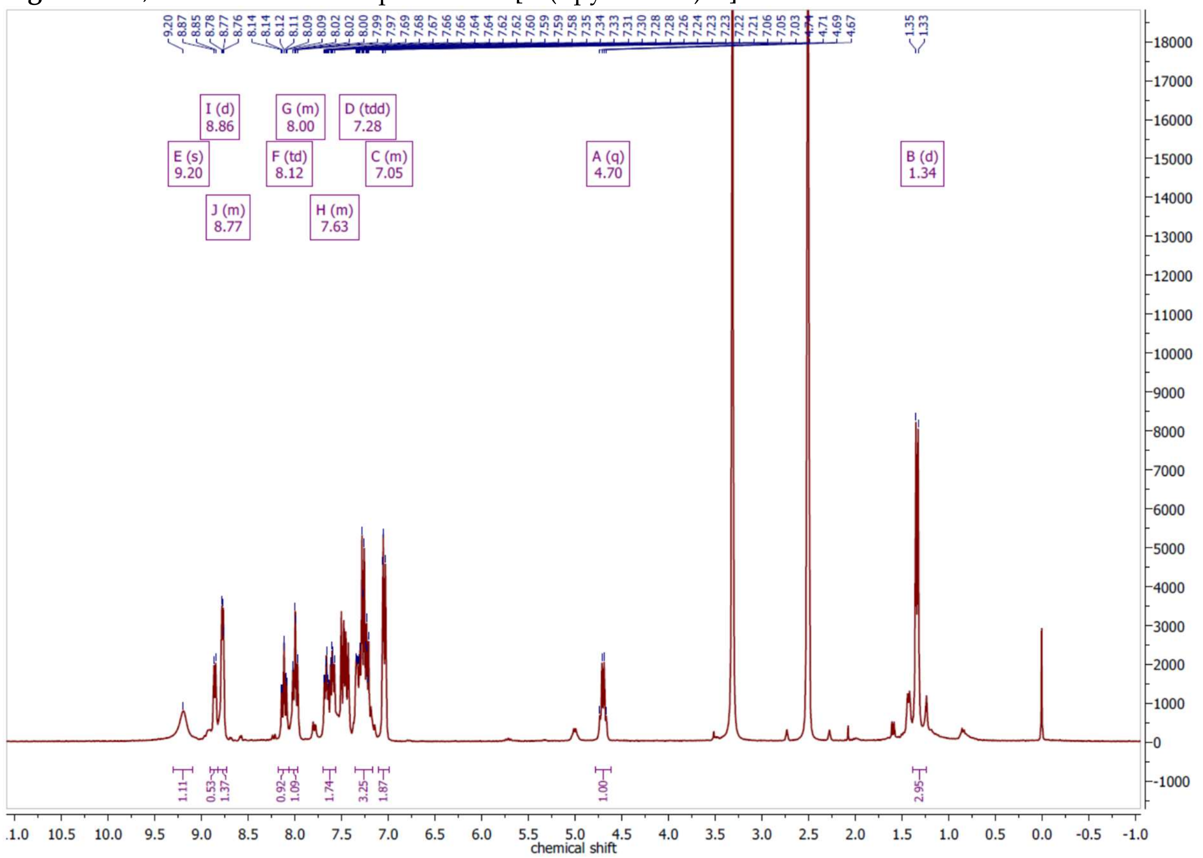

Fig. X72. ^{1}H NMR spectrum of [Pt(dpyTSCmB)(CN)] in DMSO-d_6 at 300 MHz.

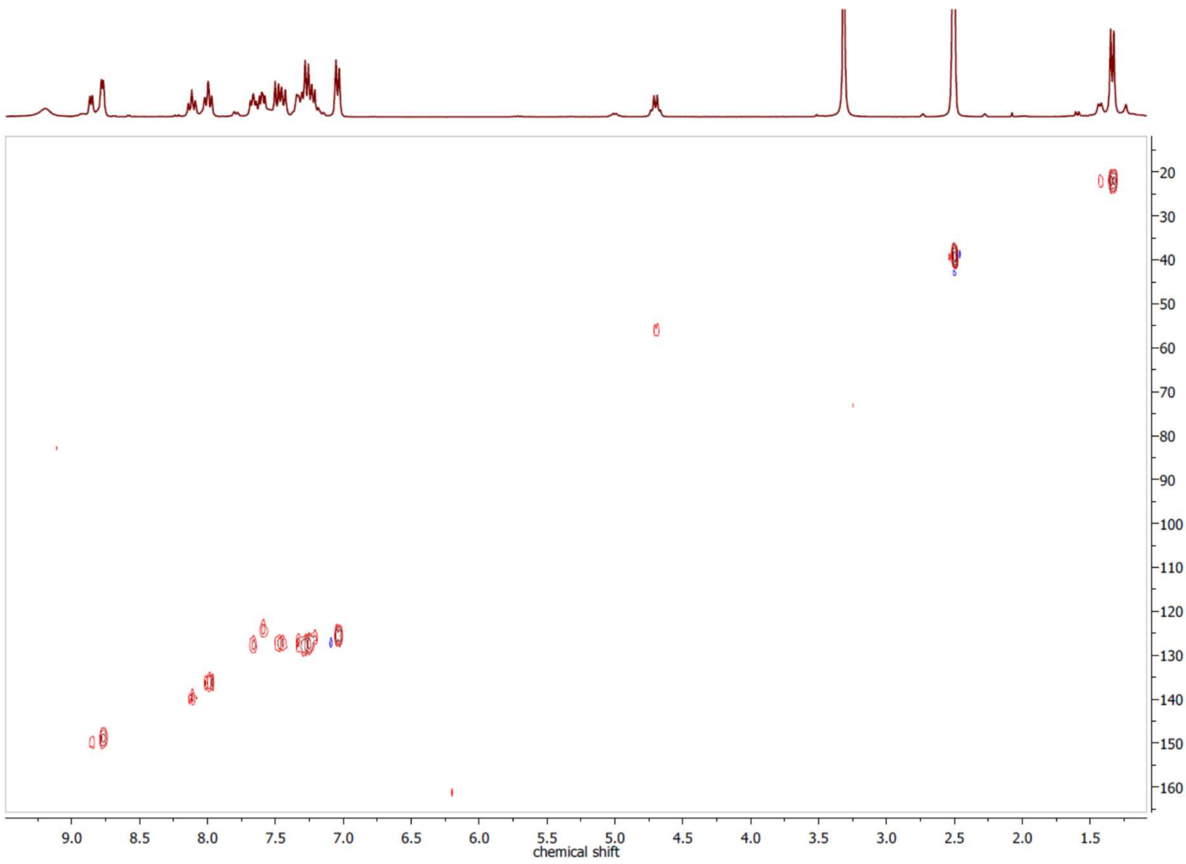

Fig. X73. $^{1}H,^{13}C$ HSQC NMR spectrum of [Pt(dpyTSCmB)(CN)] in DMSO-d_6.

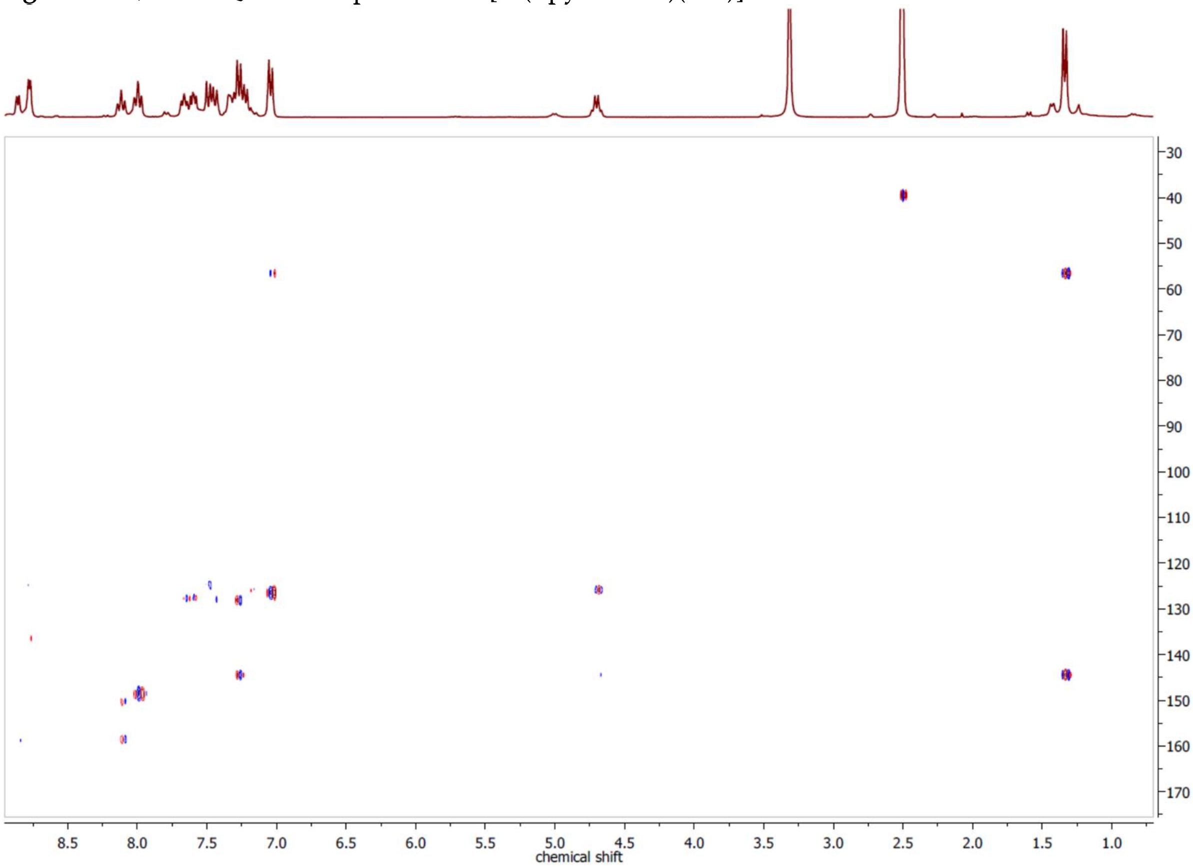

Fig. X74. $^{1}H,^{13}C$ HMBC NMR spectrum of [Pt(dpyTSCmB)(CN)] in DMSO-d_6.

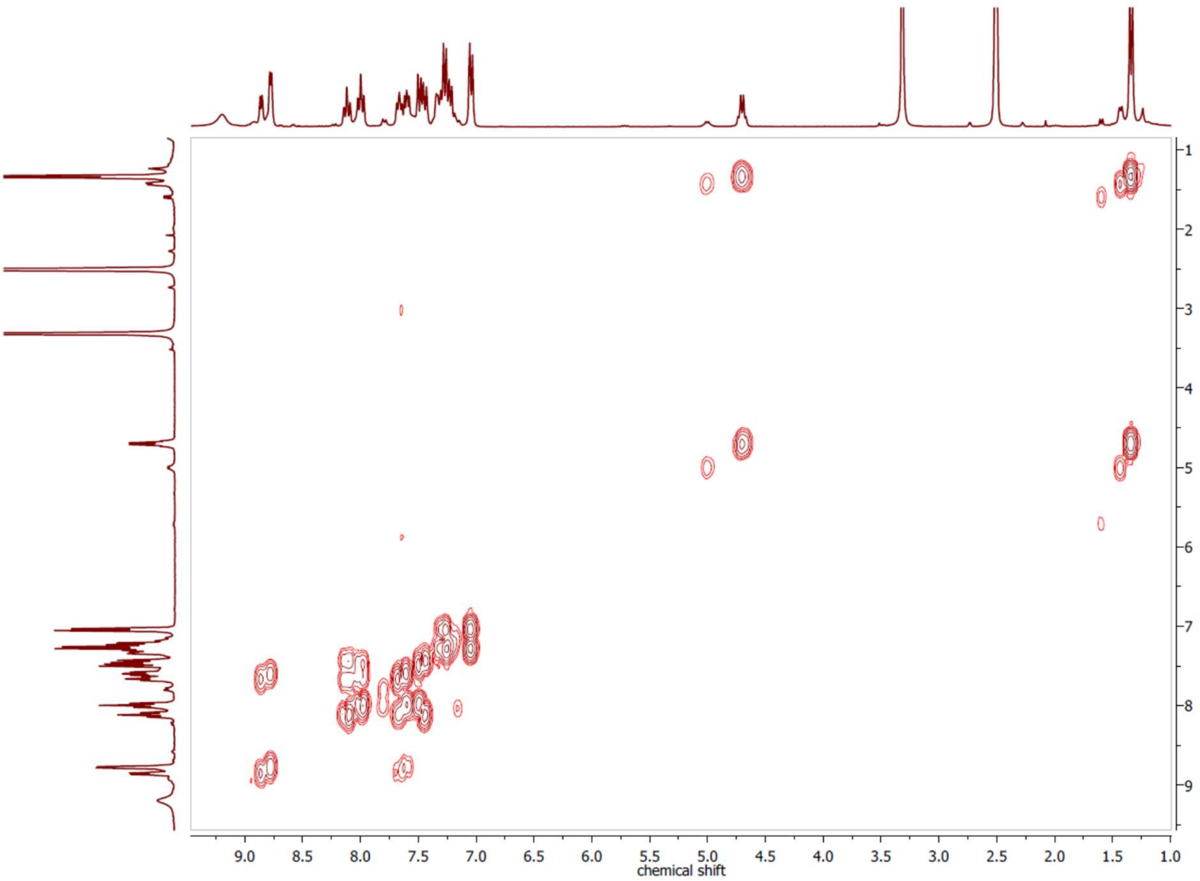

Fig. X75. $^{1}H,^{1}H$ COSY NMR spectrum of [Pt(dpyTSCmB)(CN)] in DMSO-d_6.

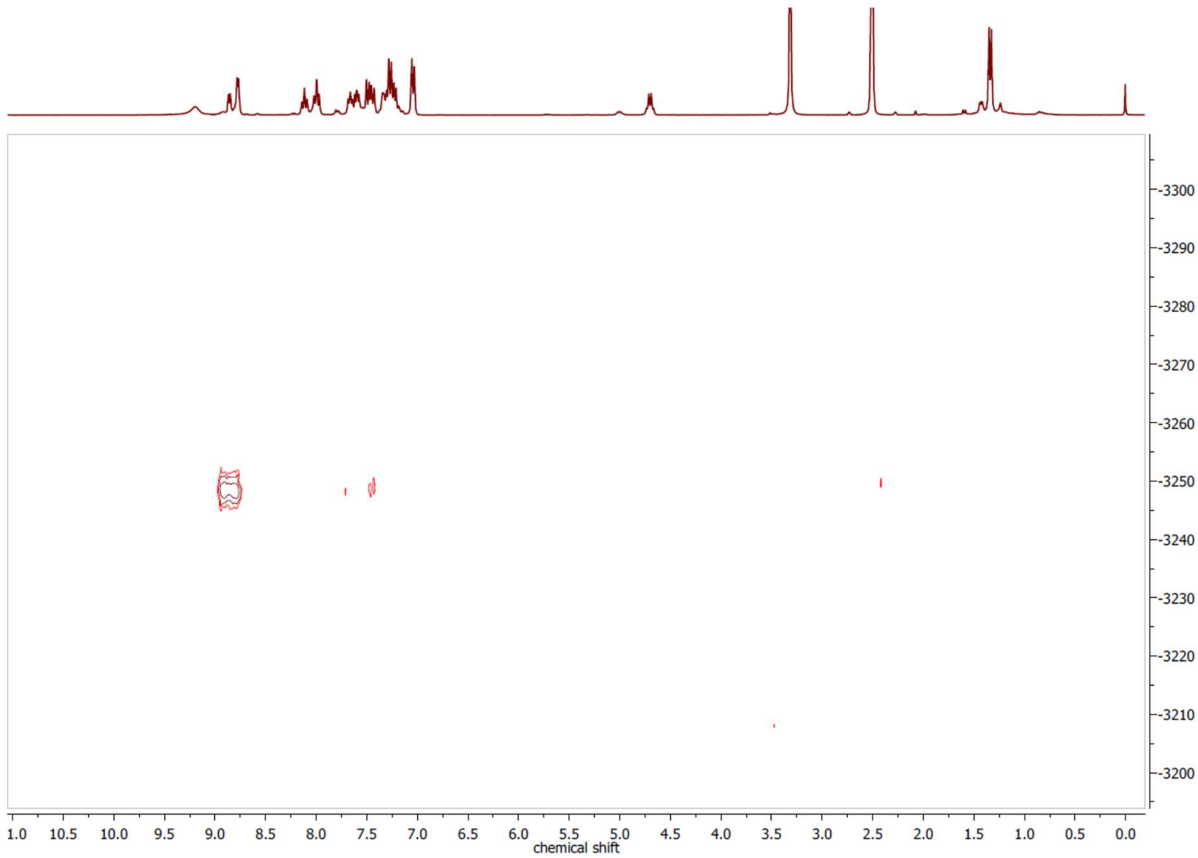

Fig. X76. $^{1}H,^{195}Pt$ HMBC NMR spectrum of [Pt(dpyTSCmB)(CN)] in DMSO-d_6.

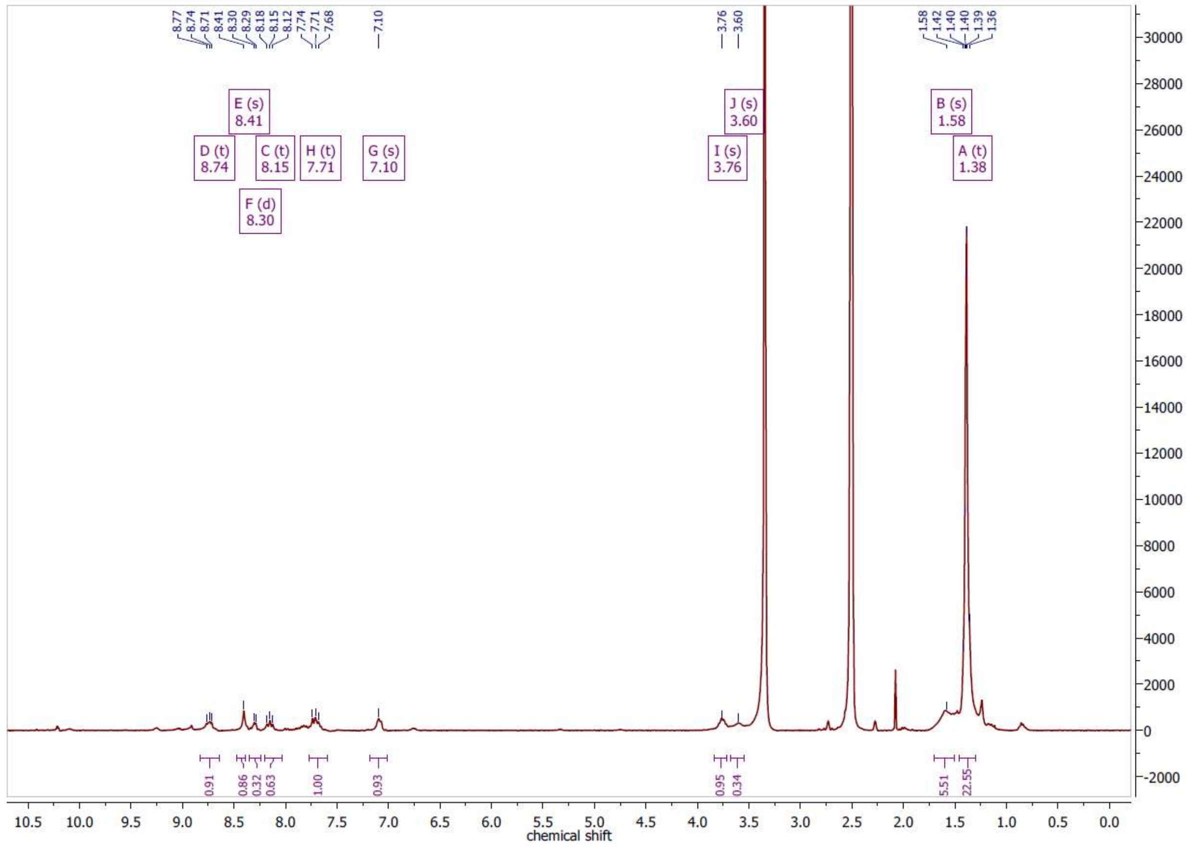

Fig. X77. ^{1}H NMR spectrum of [Pt(fpyTSCLp)Cl] in DMSO-d_6 at 300 MHz.

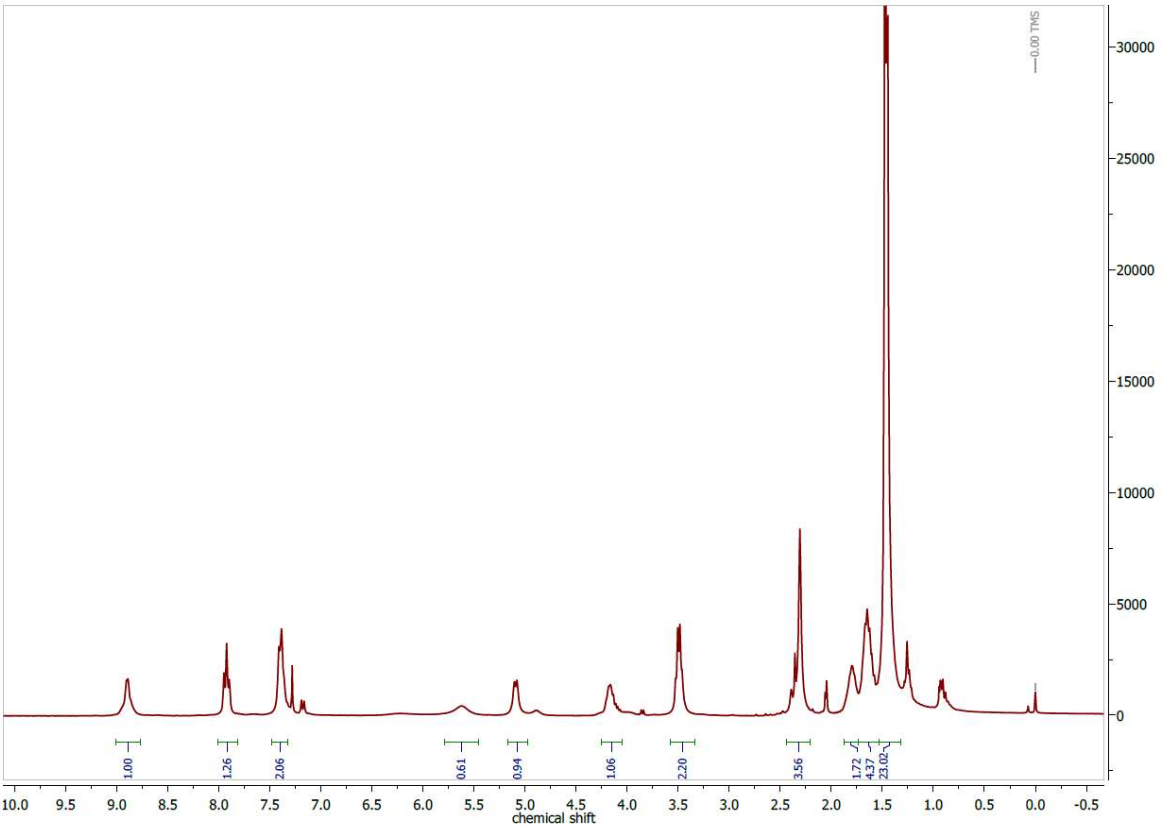

Fig. X78. ^{1}H NMR spectrum of [Pt(apyTSCLp)Cl] in DMSO-d_6 at 300 MHz.

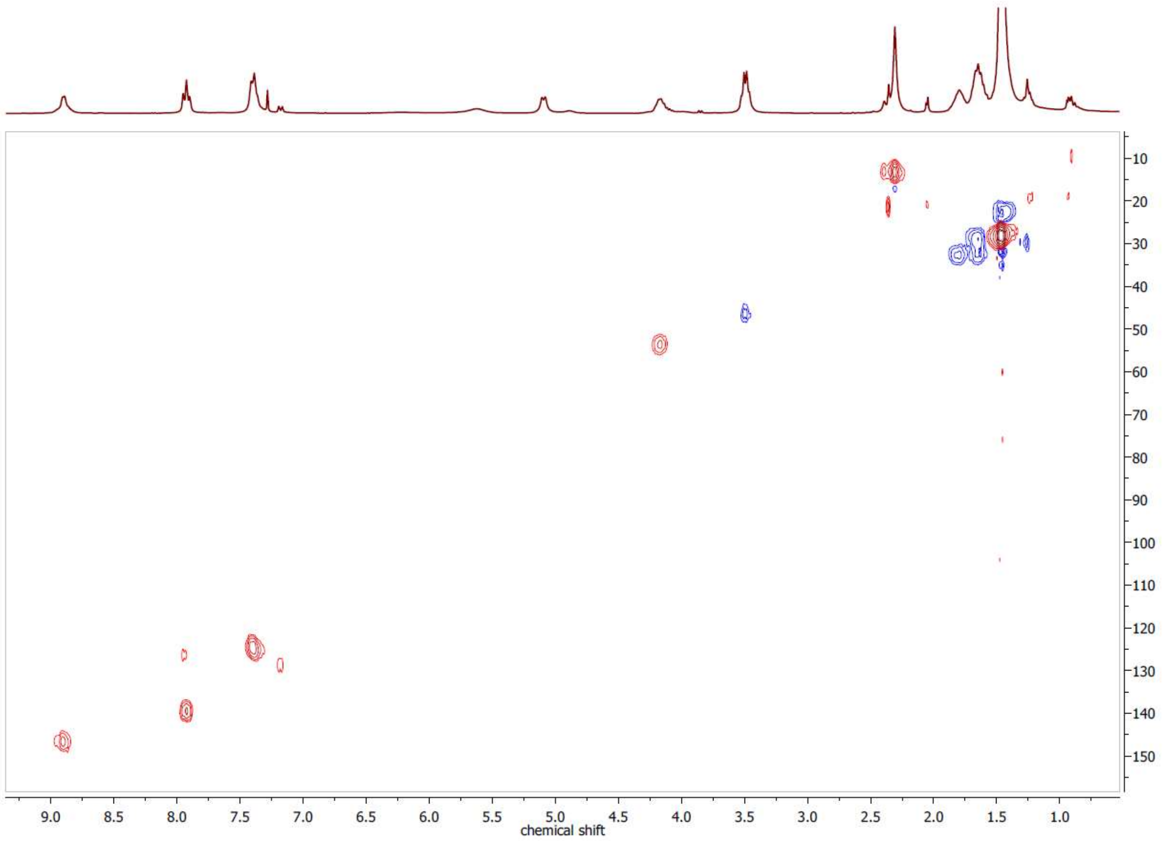

Fig. X79. $^{1}H,^{13}C$ HSQCed NMR spectrum of [Pt(apyTSCLp)Cl] in DMSO-d_6.

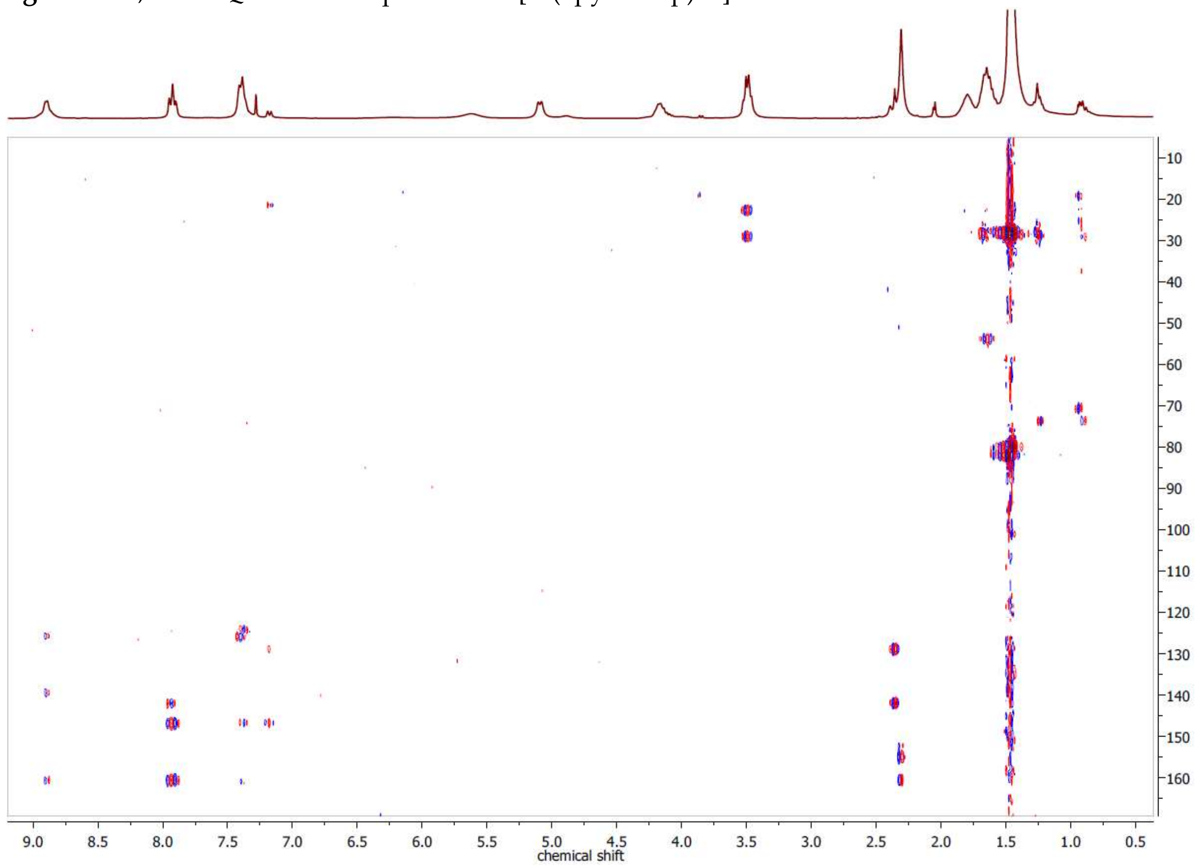

Fig. X80. $^{1}H,^{13}C$ HMBC NMR spectrum of [Pt(apyTSCLp)Cl] in DMSO-d_6.

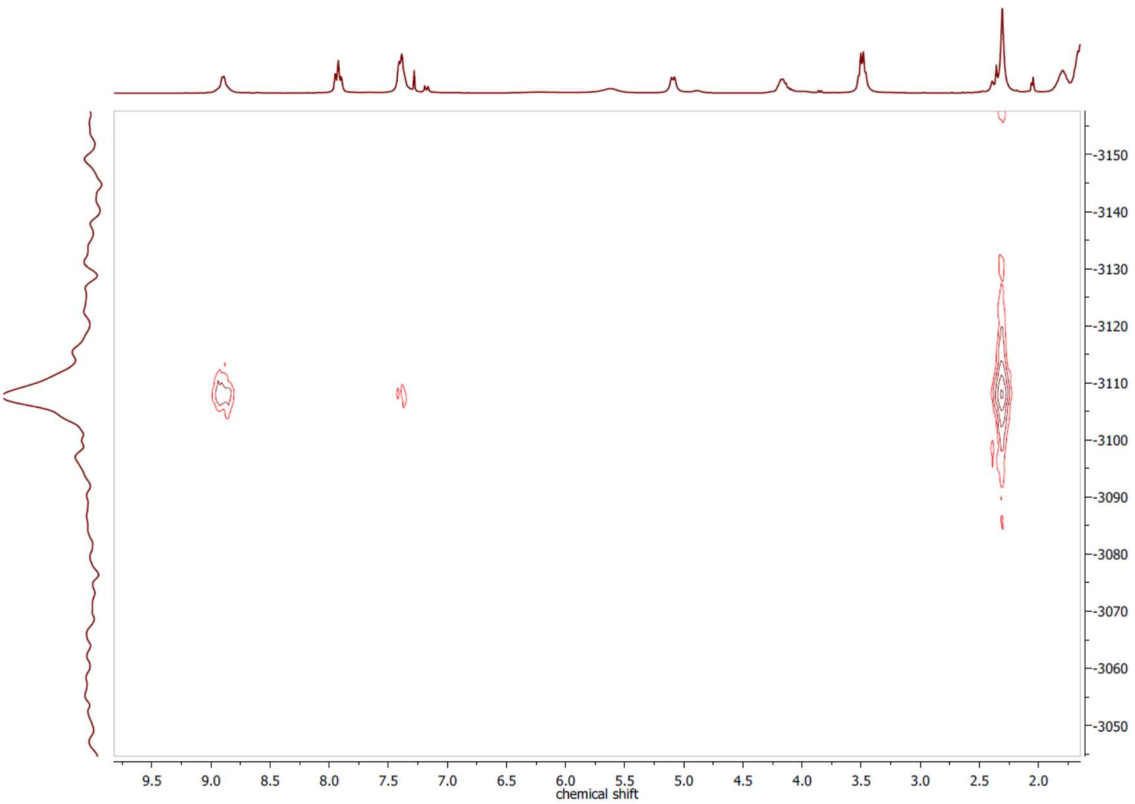

Fig. X81. $^{1}H,^{195}Pt$ HMBC NMR spectrum of [Pt(apyTSCLp)Cl] in DMSO-d_6.

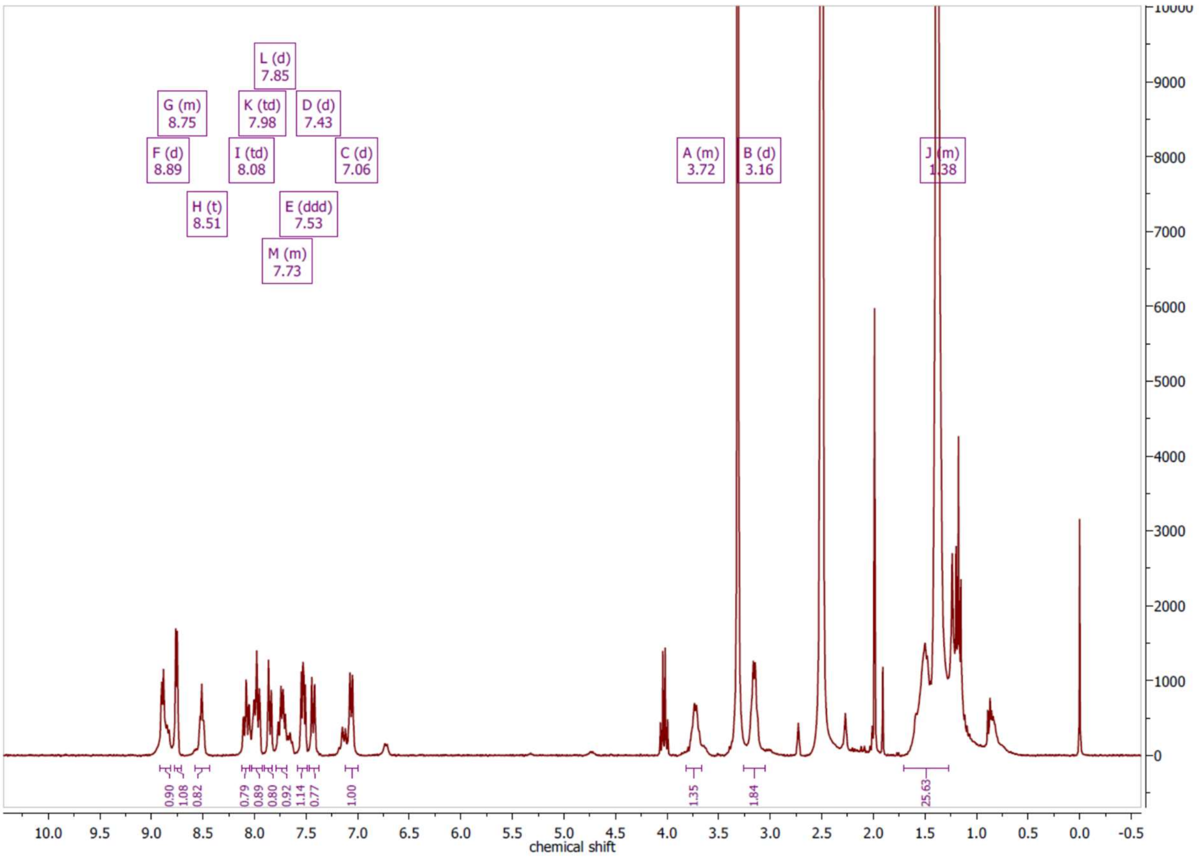

Fig. X82. ^{1}H NMR spectrum of [Pt(dpyTSCLp)Cl] in DMSO-d_6 at 300 MHz.
